# Temporal trends in prevalence of chronic liver disease among women of childbearing age from 1992 to 2021

**DOI:** 10.3389/fgwh.2026.1641073

**Published:** 2026-02-24

**Authors:** Hedan Chen, Hongwei Wu, Junyan Liu, Ali Li, Weiti Wu, Ling Lin, Ni Zhou, Yan Chen, Yonghui Lu, Yongzhi Tang, Hui Shao

**Affiliations:** 1 Department of Infectious Diseases, Taizhou Hospital of Zhejiang Province Affiliated to Wenzhou Medical University, Linhai, Zhejiang, China; 2 Department of Infectious Diseases, Taizhou Enze Medical Center (Group) Enze Hospital, Taizhou, Zhejiang, China

**Keywords:** age-period-cohort model, chronic liver disease, global burden of disease, sociodemographic index, temporal trend, women of childbearing age

## Abstract

**Background:**

Chronic liver disease (CLD) is a leading cause of global mortality. This study aimed to comprehensively analyze the temporal trends in CLD prevalence among women of childbearing age (WCBA) over a 30-year period.

**Methods:**

An age-period-cohort (APC) model was developed to assess the overall annual percentage change [net drift (ND), % per year] and the annual percentage change within distinct age groups (local drift, % per year) in CLD prevalence from 1992 to 2021. The APC model incorporated longitudinal age-specific rates, adjusting for deviations across time periods (age effects), as well as period/cohort relative risks (period/cohort effects).

**Results:**

From 1992 to 2021, the global ND in CLD prevalence among WCBA was 0.057% per year [95% confidence interval (CI): 0.029%–0.084%], with regional variation ranging from −0.27% to 0.66%. Local drift analysis indicated that age groups with increasing prevalence were more prominent in high sociodemographic index region (SDIR), while those with decreasing prevalence were more common in low SDIR. Age effects showed consistent patterns across SDIRs, with risk increasing progressively with age. Period risks were relatively lower in low SDIR, while more adverse period risks were observed in other regions. Additionally, improvements in prevalence were seen across birth cohorts in all regions.

**Conclusion:**

Over the past three decades, the global prevalence of CLD among WCBA has predominantly exhibited an adverse trend. Targeted advancements in prevention, management, and treatment of CLD are essential to mitigate relative risks for successive birth cohorts.

## Introduction

Cirrhosis and chronic liver disease (CLD) can result from various etiologies, with the most prominent being hepatitis B virus (HBV) infection, hepatitis C virus (HCV) infection, excessive alcohol consumption, and non-alcoholic fatty liver disease (NAFLD) (1). CLD is among the top ten leading causes of global mortality (2). Although the incidence of HBV has declined due to vaccination programs (3), the prevalence of NAFLD and alcoholic liver disease (ALD) continues to rise, contributing to an anticipated increase in the overall prevalence of CLD in the near future (4). The etiology of CLD exhibits remarkable variations across different sociodemographic index regions (SDIRs) (5). In high SDIR, lifestyle factors, such as obesity and excessive alcohol consumption, have emerged as the primary drivers of the rising incidence of CLD. Conversely, in low SDIR, viral infections, particularly HBV and HCV, remain the leading causes of CLD (3, 5). The impact of these regional disparities in etiology is reflected in the global burden of CLD, with each country undergoing distinct epidemiological shifts that align with evolving public health priorities, healthcare access, and economic development (6). Gaining a deeper understanding of these etiological variations across SDIRs and key countries is crucial for the development of targeted prevention, diagnosis, and treatment strategies for CLD at a global level.

The World Health Organization (WHO) characterizes women of childbearing age (WCBA) as women aged 15–49 years, a demographic that constitutes approximately one-quarter of the global population. With a global fertility rate of 2.4 children per woman, the health status of women within this age group is closely tied to the well-being of newborns (5). As the incidence of liver disease continues to rise among WCBA, a concomitant increase in pregnancies in this demographic is noteworthy. Previous research indicated that the delivery rate among women with cirrhosis has nearly doubled over the past two decades (6). CLD significantly and adversely impacts maternal morbidity and mortality (7). Women with CLD are at an increased risk of giving birth to infants with low birth weight or preterm birth, with up to two-thirds of pregnant women with CLD experiencing preterm birth. Notably, one-fifth of these pregnancies result in deliveries before 30 weeks (8). Pregnant women diagnosed with NAFLD are at elevated risk of developing gestational diabetes and preeclampsia (9).

While numerous studies have examined the prevalence of CLD in specific age groups, there is a notable gap in understanding the trends in CLD prevalence among WCBA, particularly with regard to age, period, and birth cohort effects. While the global burden of CLD is noteworthy, it is essential to acknowledge the growing importance of understanding the specific etiologies driving these trends. Remarkably, NAFLD has emerged as the leading cause of CLD, surpassing HBV and HCV in several regions, reflecting the global shift towards metabolic liver diseases. However, the complexities in the temporal evolution of CLD necessitate a more granular examination of etiology-specific trends, especially by age and period. For instance, the rise in NAFLD prevalence has been more remarkable in older age groups, particularly those over 30, where lifestyle factors, such as obesity, diabetes, and poor dietary habits have exacerbated the condition's prevalence. On the other hand, the decline in HBV prevalence, influenced by vaccination programs, is more apparent in younger cohorts, with notable reductions in incidence rates across successive generations. To address this gap, the present study also explored etiology-specific temporal trends by stratifying the data by age groups and examining period and birth cohort effects. By analyzing the evolving patterns of HBV, HCV, NAFLD, and ALD across different periods and age groups, this research aimed to provide a more comprehensive understanding of the shifting landscape of liver diseases. The study extracted data related to CLD among WCBA from the Global Burden of Disease (GBD) 2021 database, and an APC model was applied to analyze the temporal trends in CLD prevalence from 1992 to 2021, both globally and across regional and national levels. Specifically, the APC model allows for an in-depth assessment of how each etiology has contributed to the overall rise in CLD prevalence, concentrating on the differential impacts of age and period factors.

## Methods

### Data source

The GBD 2021 study, released in 2024, involves health and disease data from 1980 to 2021. It provides comprehensive epidemiological assessments of 371 diseases and injuries across 204 countries and regions, with continuous updates to the data (10). Detailed information on data inputs, processing methodologies, synthesis procedures, and final models is available in the associated GBD 2021 publications (10). The GBD network utilized standardized Bayesian tools to integrate a vast array of data across multiple time periods, age groups, geographical regions, and health conditions, enabling precise estimation of disease burden. This methodology promotes the estimation of disease burden even in countries lacking primary data by “borrowing” information from existing data sources, thus enabling comprehensive global estimates of chronic diseases.

The SDI, calculated using fertility rates for women under 25 years, average years of education for women aged 15 and above, and per capita income, serves as an indicator of a country's or region's development level. Based on the 2021 SDI value, countries were categorized into five SDI quintiles: low, low-middle, middle, high-middle, and high. The prevalence of CLD among WCBA was extracted for 204 countries and regions, categorized by SDI, with rates expressed per 100,000 population. The 95% uncertainty intervals (UIs) were derived from the 25th and 975th values of 1,000 ordered posterior distributions based on GBD algorithms (11).

### Analysis of temporal trends in CLD prevalence among WCBA

Temporal trends in CLD prevalence among WCBA from 1992 to 2021 were analyzed, concentrating on case numbers and age-standardized (AS) rates. AS prevalence rates were calculated through direct age standardization, assuming the rates follow the weighted sum of independent Poisson random variables. Additionally, the distribution of CLD prevalence was examined across seven distinct age groups (15–19, 20–24, 25–29, 30–34, 35–39, 40–44, and 45–49 years), and changes in the age distribution of prevalence over time were assessed.

The APC model is an epidemiological method based on demographic characteristics. It divides the population into different cohorts according to birth year and follow-up time (12). By analyzing the health status and disease incidence of each cohort at different time points, the model revealed trends in diseases over time and their relationships with age and period factors (13). This model helps researchers gain a deeper understanding of health status and disease development trends in various age groups, providing a scientific basis for the formulation of prevention and treatment strategies (14). We utilized the APC Web tool, a network analysis tool developed by the National Cancer Institute. The design of this tool is based on the “estimable functions” theory within the APC model, meaning that while individual age, period, and cohort coefficients cannot be uniquely identified, specific linear combinations yield statistically consistent and unique solutions. The backend of the tool employs algorithms such as the intrinsic estimator method to compute these estimable functions and directly outputs public health-relevant metrics, including period/cohort relative risk ratios, age-specific incidence curves, and local drifts. These results do not rely on arbitrary constraints imposed by researchers and represent standardized outputs derived after resolving the inherent collinearity issues of the model (15, 16). For this investigation, estimates of CLD prevalence among WCBA and corresponding population data from the GBD 2021 dataset were used as foundational inputs into the APC model.

In the analysis, concentrating on WCBA (15–49 years), seven distinct age groups were employed: 45–49, 40–44, 35–39, 30–34, 25–29, 20–24, and 15–19 years. This age segmentation aligns with the requisite five-year age intervals corresponding to five-year calendar periods, a fundamental requirement for the APC model. By adhering to these specifications, the aim was to capture comprehensive insights into how CLD prevalence evolves across different stages of reproductive age, thereby illuminating potential avenues for targeted intervention and policy formulation. The study period (1992–2021) was divided into six five-year periods: 1992–1996, 1997–2001, 2002–2006, 2007–2011, 2012–2016, and 2017–2021. Accordingly, 12 partially overlapping 10-year birth cohorts were used: 1942–1951, 1947–1956, 1952–1961, 1957–1966, 1962–1971, 1967–1976, 1972–1981, 1977–1986, 1982–1991, 1987–1996, 1992–2001, and 1997–2006.

The APC model could also estimate the overall annual percentage change in prevalence (ND, % per year) and the annual percentage change in prevalence in specific age groups (local drift, % per year), reflecting trends influenced by birth cohort effects (16). The significance of these annual percentage changes was determined through the Wald *χ*² test (16). In the APC model, age effects were represented by fitted longitudinal age-specific rates adjusted for period deviations. The influences of period and cohort were elucidated by calculating period/cohort relative risks, formulated as the ratios of prevalence rates associated with specific ages across each period/cohort relative to an arbitrarily selected reference period/cohort. Importantly, the utilization of this reference period/cohort was inherently arbitrary and exerted no influence on the analytical robustness of the outcomes. In this study, R 4.3.1 software was used to process and visualize data. P-value <0.05 indicated statistical significance.

## Results

### Trends in CLD prevalence in WCBA, 1992–2021

The global and regional prevalence rates of CLD, AS prevalence rates, and NDs are presented in Table 1. From 1992 to 2021, the global prevalence of CLD among WCBA increased by 53.42%, reaching 413.67 million cases (95% UI: 377.66–459.28) in 2021, reflecting the growing global population. The percentage variation in CLD prevalence was elevated across all SDIRs. In 2021, the global AS prevalence rate of CLD among WCBA was 20,877.84 per 100,000 (95% UI: 17,250.33–25,044.54), marking a 4.4% increase from 1992. Relative increases in AS prevalence rates were found in low-middle, high-middle, and high SDIRs. The APC model further estimated a global ND in CLD prevalence among WCBA at 0.057% per year (95% CI: 0.029%–0.084%), with variations ranging from −0.27% (95% CI: −0.32% to −0.23%) in middle SDIR to 0.66% (95% CI: 0.62%–0.70%) in high SDIR.

**Table 1:** Global and regional prevalence rates of CLD among WCBA, age-standardized prevalence rates, and NDs from 1992 to 2021.

Traits	Global		High SDI		High-middle SDI		Middle SDI		Low-middle SDI		Low SDI	
	1992	2021	1992	2021	1992	2021	1992	2021	1992	2021	1992	2021
Population												
Number in millions (95% UI)	5,497.21 (5,379.11, 5,624.27)	7,891.35 (7,666.73, 8,131.22)	893.58	1,094.05	1,086.54	1,304.03	1,779.77	2,448.54	1,205.82	1,921.11	526.36	1,117.38
Percentage of global level (%)	100	100	16.26	13.86	19.77	16.52	32.38	31.03	21.94	24.34	9.85	14.16
Prevalence												
Number in millions (95% UI)	269.63 (248.01, 296.81)	413.67 (377.66, 459.28)	23.63 (21.56, 26.18)	31.62 (28.60, 35.32)	56.27 (51.83, 62.03)	67.42 (61.22, 75.25)	104.81 (96.56, 115.35)	147.55 (134. 76, 164.38)	58.11 (53.32, 63.97)	107.17 (97.56, 118.74)	26.57 (24.52, 28.92)	59.59 (54.65, 65.15)
Percentage of global level (%)	100	100	8.76	7.64	20.87	16.30	38.87	35.67	21.55	25.91	9.85	14.41
Percentage change of prevalence, 1992–2021 (%)	53.42		33.81		19.82		40.78		84.43		124.28	
Age-standardized prevalence rate												
Rate per 100,000 (95% UI)	19,997.38 (16,964.05, 23,501.98)	20,877.84 (17,250.33, 25,044.54)	10,028.67 (8,281.08, 12,137.16)	12,172.40 (9,786.56, 14,962.89)	19,868.63 (16,875.18, 2,3384.03)	20,109.07 (16,441.92, 24,339.41)	23,655.89 (20,119.54, 27,770.50)	22,896.70 (18,828.96, 27,605.91)	21,587.73 (18,228.68, 25,551.42)	21,669.57 (17,910.79, 26,002.33)	23,916.18 (20,679.64, 27,711.04)	23,217.47 (19,732.88, 27,227.2)
APC model estimates												
Net drift of prevalence (% per year, 95% CI)	0.057 (0.029, 0.084)		0.66 (0.62, 0.70)		0.19 (−0.27, −0.12)		0.27 (−0.32, −0.23)		0.0074 (−0.0050, 0.020)		0.14 (−0.15, −0.12)	

APC, age-period-cohort; CLD, chronic liver disease; ND, net drift; SDI, sociodemographic index; WCBA, women of childbearing age.

**Figure 1:**
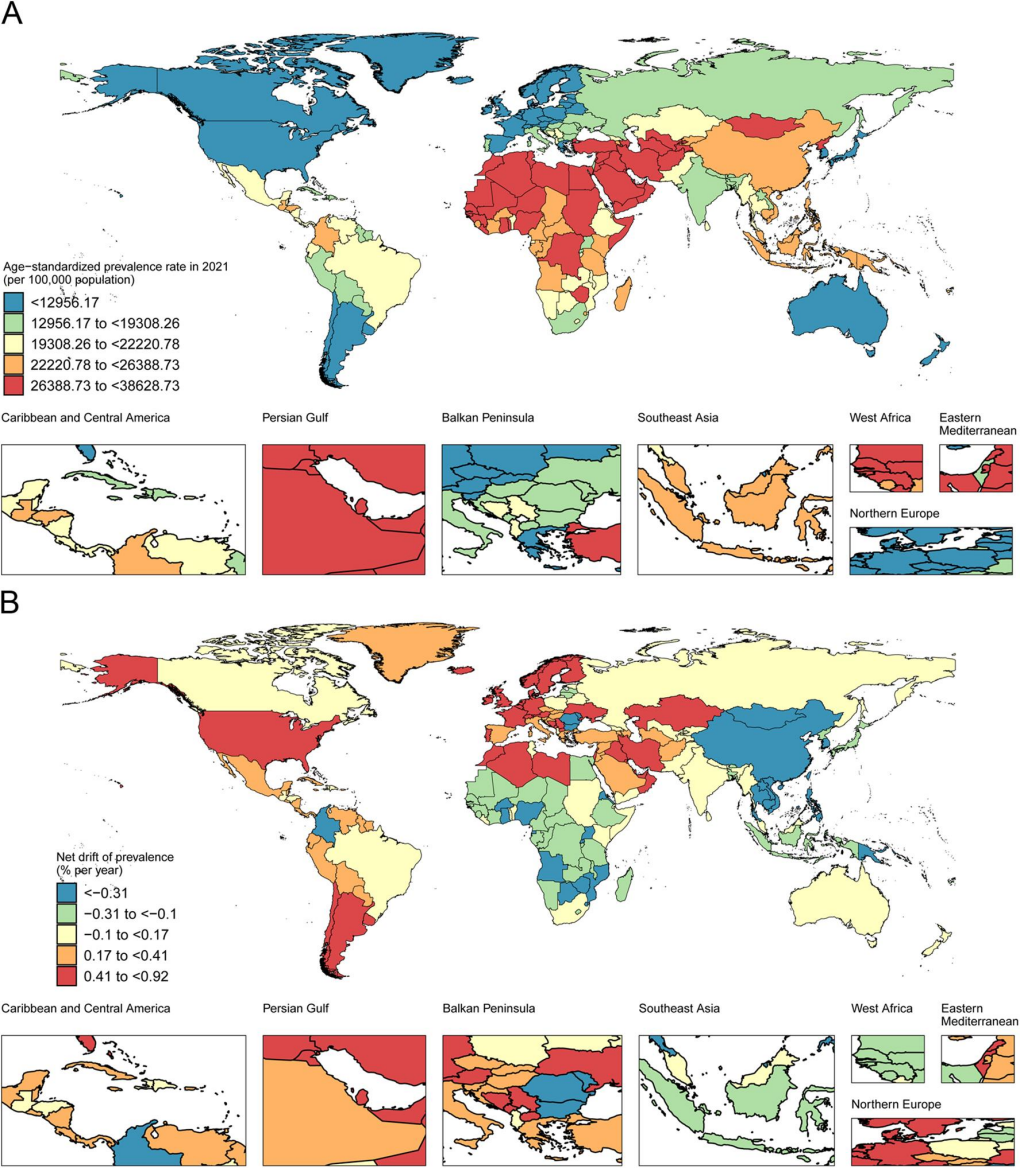
AS prevalence rates and ND of prevalence for CLD among WCBA in 204 countries and regions in 2021. **(A)** AS prevalence rates of CLD among WCBA in 2021 worldwide. **(B)** NDs of CLD prevalence among WCBA from 1992 to 2021 worldwide.

National prevalence rates and AS prevalence rates of CLD among WCBA in 2021, along with NDs of prevalence trends from 1992 to 2021, are illustrated in Figure 1 and online Supplementary Table 1. In 2021, 66 countries and regions reported a CLD prevalence of at least 1 million, involving China, India, Indonesia, Nigeria, and Brazil, accounting for 47.35% of the global CLD prevalence among WCBA. In 103 countries, AS prevalence rates met or exceeded the global average, with 21 countries, including Egypt, Qatar, and Kuwait, exceeding 1.5 times the global average, most of which were high and high-middle SDI countries. From 1992 to 2021, the United Arab Emirates yielded the highest increase in AS prevalence (25.91%), with an annual ND of 0.92% (95% CI: 0.89%–0.94%). AS prevalence rates declined in 90 countries. Although China and India had the highest CLD prevalence due to their large populations, their AS prevalence rates decreased, with relatively minor changes in ND. Among 204 countries and regions, the APC model estimated ND trends indicating an upward trend in 99 countries, a downward trend in 86 countries, and a relatively stable trend in 19 countries, demonstrating strong heterogeneity in global CLD prevalence trends.

**Figure 2:**
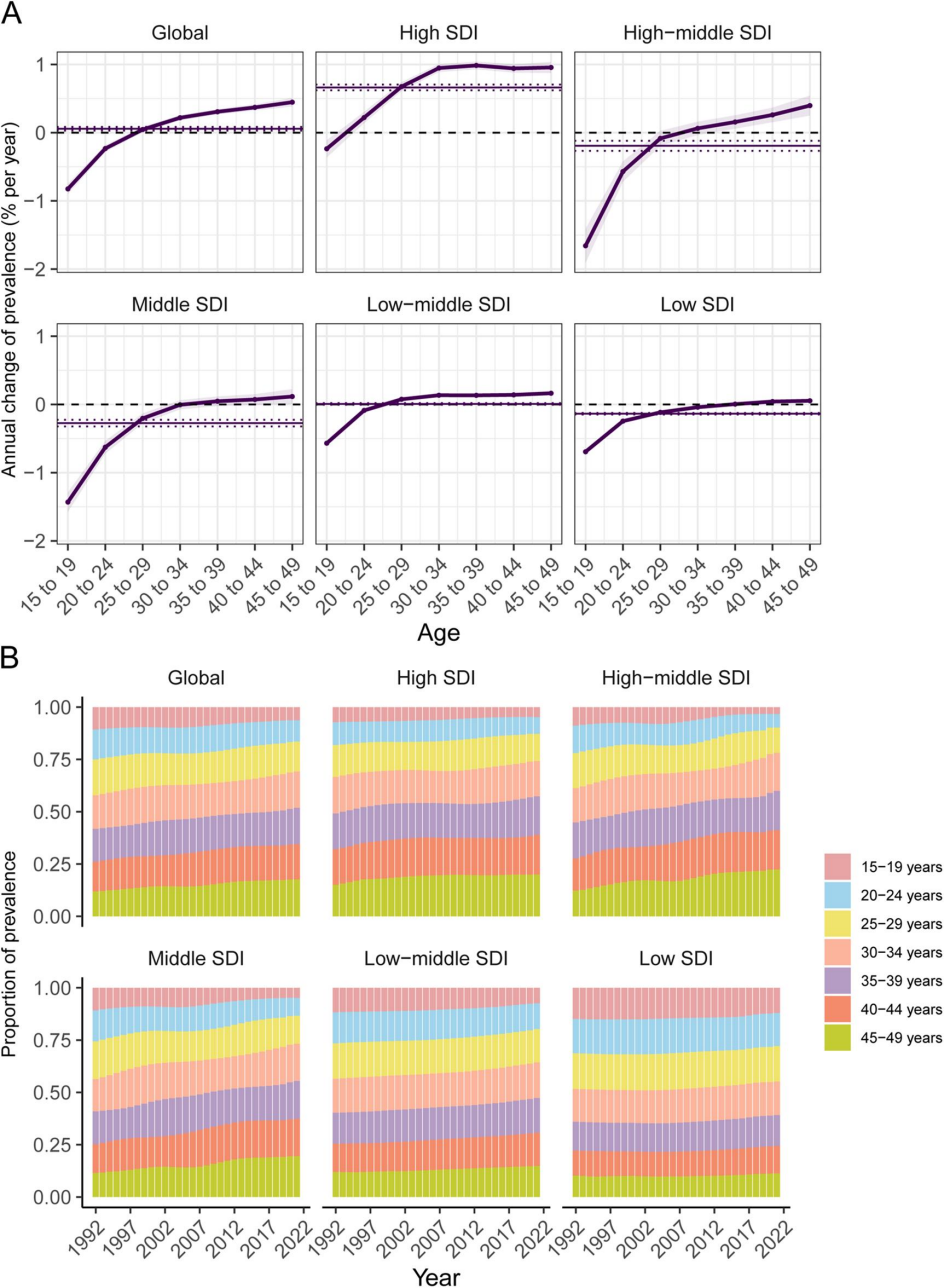
Local drifts and temporal changes in age distribution of CLD prevalence among WCBA from 1992 to 2021 across sDIRs quintiles. **(A)** Local drifts in CLD prevalence among WCBA across seven age groups. **(B)** Temporal changes in the age distribution of CLD prevalence among WCBA from 1992 to 2021.

The annual percentage change in age-specific prevalence of CLD among WCBA, calculated as local drifts using the APC model, is presented in Figure 2A and online Supplementary Table 2. On a global scale, the prevalence of CLD among WCBA decreased in the 15–19 and 20–24 age groups, while it increased in the 30–34 to 45–49 age groups. The decline in the 15–19 and 20–24 age groups diminished with advancing age. For adolescents (15–19 years), a downward trend in CLD prevalence was found across all SDIRs, and the most significant decline was identified in the high-middle SDIR (−1.66%, 95% CI: −1.41% to −1.91%), while the least significant decline was noted in the high SDIR (−0.24%, 95% CI: −0.11% to −0.36%). Furthermore, in high SDIR countries, prevalence increased across all age groups, except for the 15–19 years group. The local drifts in CLD prevalence for each age group across different countries are detailed in online Supplementary Table 3.

The temporal changes in age distribution of CLD prevalence among WCBA were shown in Figure 2B. Globally, CLD prevalence transitioned from 15–19 years to 20–49 years, a trend evident across all SDIR except low SDI. Additionally, older age groups represented a larger share of the prevalence, with over 50% of CLD prevalence in 2021 concentrated among women aged 35 years and older in all regions except low and low-middle SDIRs.

### Composition of CLD causes among WCBA from 1992 to 2021 across different SDI regions

**Figure 3:**
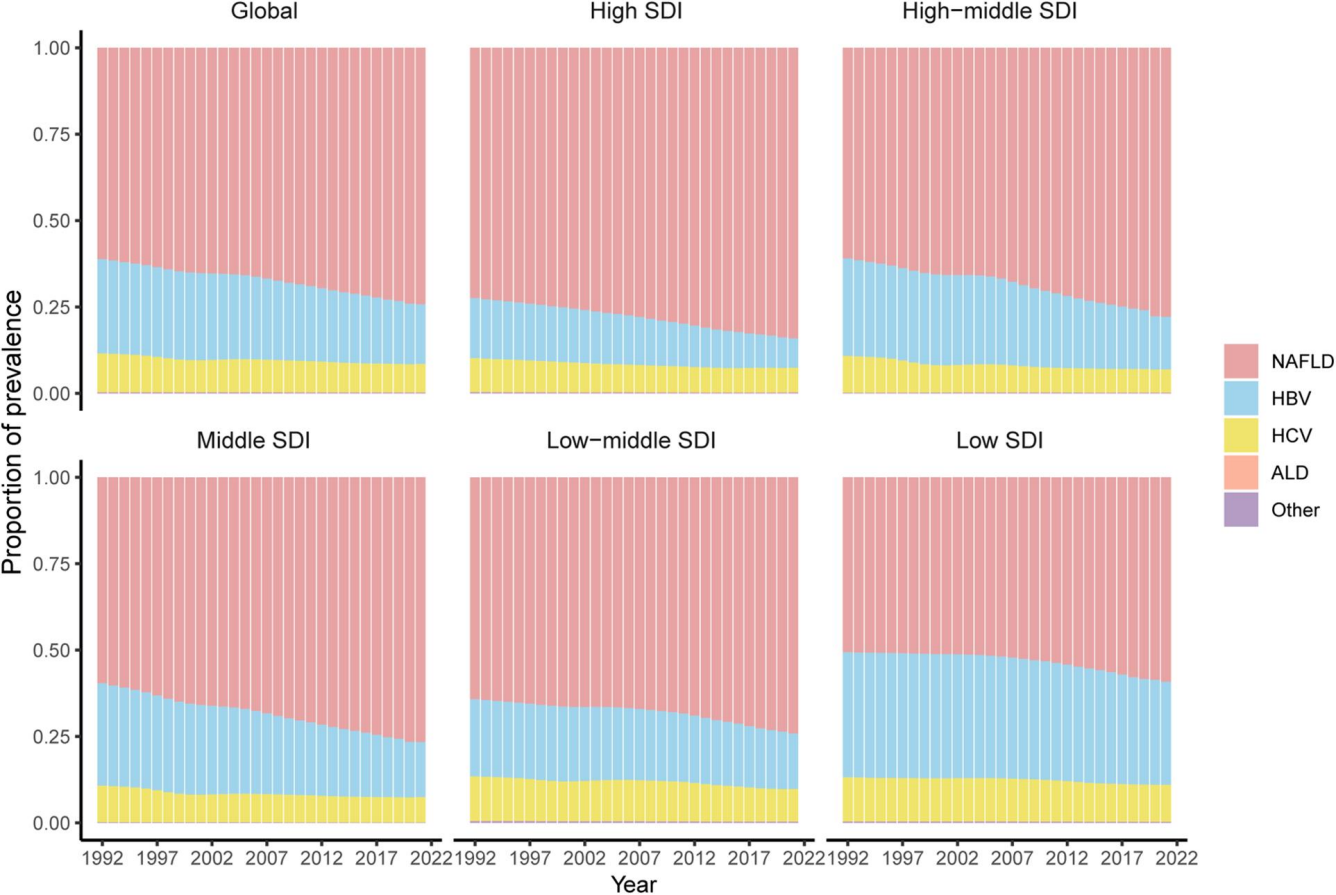
Composition of HBV, HCV, ALD, NAFLD, and other CLD among WCBA in different SDIRs from 1992 to 2021.

In all five SDIRs, NAFLD consistently constituted the largest proportion of CLD, accounting for over 50% of all liver diseases (Figure 3). Globally, the prevalence of HBV declined, whereas NAFLD significantly increased, rising from 61.11% in 1992 to 74.24% in 2021, while HBV decreased from 27.34% to 17.26%. This trend was consistent across all SDIRs. HCV showed a slow decline globally and across SDIRs, but in low SDIR, the decrease in HBV and increase in NAFLD were relatively moderate (HBV from 32.63% in 1992 to 29.81% in 2021, and NAFLD from 50.60% in 1992 to 59.19% in 2021). The proportion of ALD cases showed relatively minor changes, decreasing from 0.057% in 1992 to 0.052% in 2021 (online Supplementary Table 4).

### Age, period and birth cohort effects on CLD prevalence in WCBA

**Figure 4:**
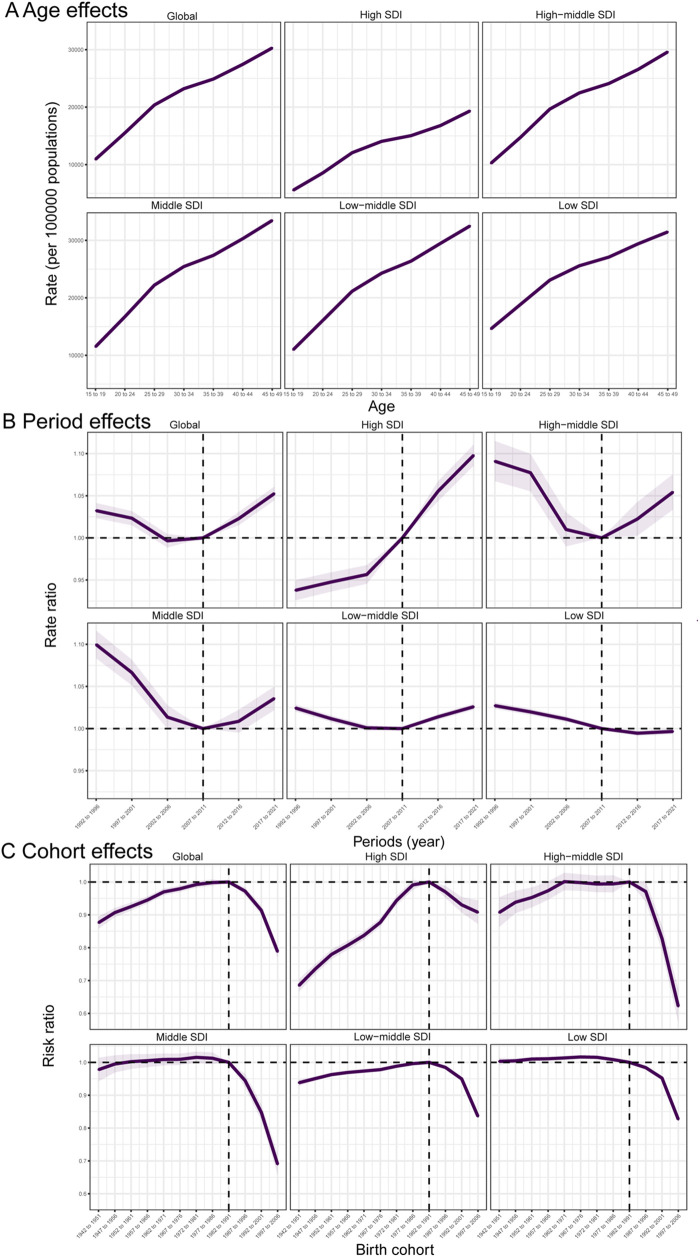
Effects of age, period, and birth cohort on CLD prevalence among WCBA across SDI quintiles. **(A)** Age effects; **(B)** period effects; **(C)** birth cohort effects.

Age, period, and birth cohort effects derived from the APC model were shown in Figure 4 and online Supplementary Tables 5–7. Age effects demonstrated uniform patterns across diverse SDIRs, exhibiting a minimal risk in the 15–19-year group that progressively elevated with advancing age. Specifically, high SDIR consistently reported lower overall prevalence rates among all age-based groups relative to their lower SDI counterparts. Period effects revealed a remarkable trend: the prevalence initially attenuated before rising across most SDIRs, with notable exceptions in high and low SDIRs. During the study period, all regions except high and low SDIRs had more adverse period risks in most periods. In high SDIR, the risk increased gradually over time, while in low SDIR, the risk decreased gradually in later periods and stabilized thereafter. When comparing the 2017–2021 period to the reference period of 2007–2011, the relative period risk was 1.098 (95% CI: 1.086–1.110) in high SDIR, 1.054 (95% CI: 1.034–1.075) in high-middle SDIR, 1.036 (95% CI: 1.022–1.050) in middle SDIR, and 0.997 (95% CI: 0.993–1.000) in low SDIR. Regarding birth cohort effects, the prevalence risk for successive birth cohorts globally initially increased and then decreased, with overall improvements in prevalence across all SDIRs for successive birth cohorts. Relative to individuals born in the 1982–1991 cohort, the relative cohort risk for those from the 1997–2006 cohort was 0.836 (95% CI: 0.828–0.844) in low-middle SDIR and 0.908 (95% CI: 0.872–0.945) in high SDIR.

**Figure 5:**
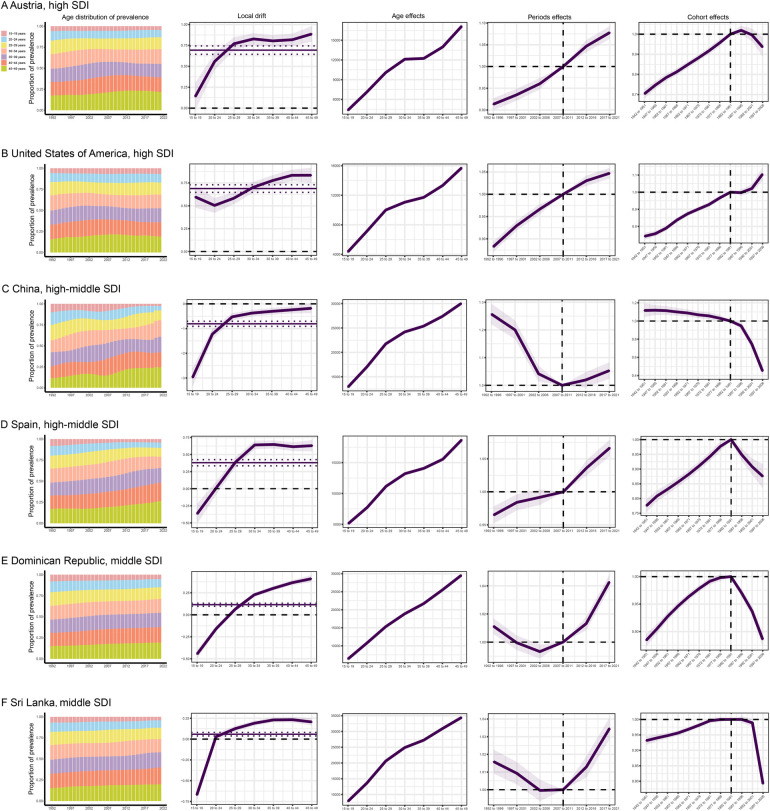
The effects of age, period, and birth cohort on prevalence of CLD among WCBA in the typical countries.

The influences of age, period, and birth cohort on CLD prevalence among WCBA in each country are detailed in online Supplementary Tables 8–10. Temporal trends in global CLD prevalence, highlighting several representative countries with relatively favorable and unfavorable age, period, and birth cohort effects across various SDI quintiles, are illustrated in Figure 5. The United States, a high SDI country, demonstrated unfavorable trends, with no decline in prevalence across all age groups in recent years and a worsening of period and cohort risks. In contrast, Australia exhibited more favorable trends in cohort risks among high SDI countries. China and Spain, representing high-middle SDI countries, exhibited a shift in prevalence from adolescence to adulthood. In both countries, prevalence rates among the elderly population increased. China exhibited an initial decline in risk, followed by an increase in later years, whereas Spain showed a sustained rise in prevalence, with birth cohort risks tending to decrease in the later stages. Among low SDI countries, Sierra Leone and the Central African Republic showed a decline in prevalence across all age groups, with period risks initially decreasing, followed by an increase, and cohort risks gradually decreasing.

**Figure:**
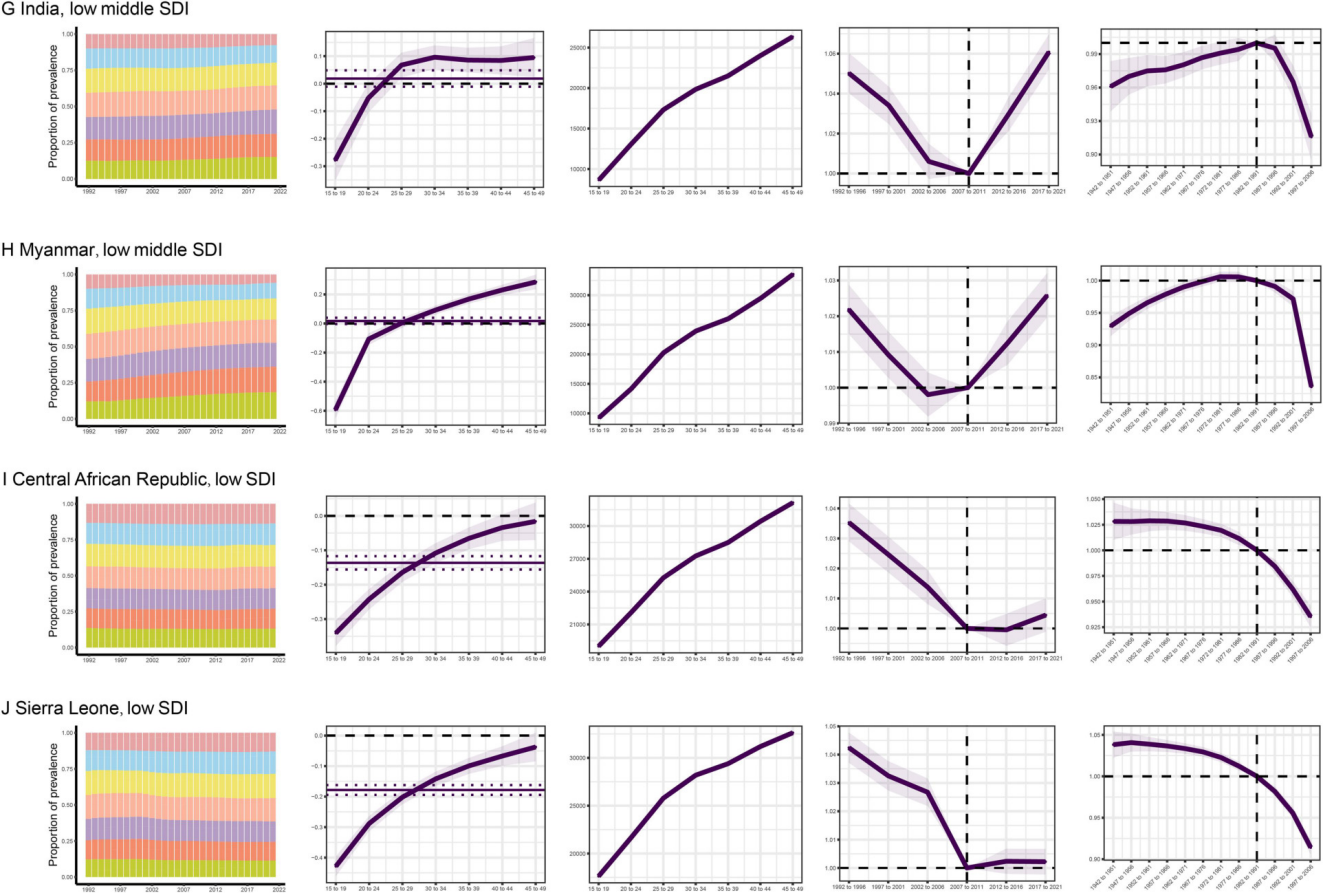


## Discussion

Notably, CLD can lead to severe complications and death, significantly impacting quality of life and imposing a substantial economic burden (17). Despite various conditions leading to CLD, HBV, HCV, NAFLD, and ALD are the most common culprits (18). In 2015, the United Nations established defined objectives under Sustainable Development Goal 3, with a target to decrease the global maternal mortality ratio to less than 70 per 100,000 live births by 2030 (19). These include gestational hypertension, gestational diabetes, preeclampsia, preterm birth, and low birth weight (6, 7). Such severe complications not only pose significant challenges to maternal survival but also impede progress towards achieving the United Nations' Sustainable Development Goals. This study explored the relative influence of age, period, and birth cohort on the prevalence trends of CLD among WCBA. The increasing global prevalence of CLD, particularly driven by NAFLD, underscores the need for public health initiatives focusing on metabolic health. Given the strong association between NAFLD and obesity, type 2 diabetes, and poor dietary habits, it is critical that preventive strategies target lifestyle factors, particularly in high-middle and high SDI countries where the trends are more pronounced. Furthermore, the rising prevalence of CLD in younger populations, coupled with the slow decline in HBV cases, suggests that while vaccine programs have been successful in curbing viral infections, the focus must now shift to managing the growing burden of metabolic liver diseases.

Globally, we observed adverse period effects and favorable birth cohort effects. CLD period effects have been rising over the past decade. Period effects reflect changes in incidence or mortality rates due to societal, economic, cultural, or environmental changes (20). Over time, with improvements in socio-economic conditions, education, and living standards, the overall incidence of CLD has increased. Recent studies indicated that the rising prevalence of CLD is mainly driven by the increasing prevalence of NAFLD (21). Over the past decade, the incidence of CLD has continued to increase, attributable primarily to the growth of metabolic-related risk factors driven by societal, economic, and environmental changes, rather than population expansion alone (22). These trends, intertwined with factors such as reduced physical activity and altered gut microbiota, underscore the pivotal role of metabolic and lifestyle factors in liver disease risk (23). The shift towards more sedentary lifestyles, accompanied by the proliferation of calorie-dense, nutrient-poor foods, has exhibited a remarkable impact on health outcomes. The increase in the period effect, particularly in the later years of the study, cannot solely be attributed to general social development. Instead, more specific factors may be influential, such as the increase in the consumption of ultra-processed foods. These dietary changes may significantly contribute to the rise in conditions, such as obesity and type 2 diabetes, which are key drivers of diseases like NAFLD (21). Given the escalating global prevalence of NAFLD, particularly in low-middle and high-middle SDI countries, public health strategies require adaptation. It is recommended to enhance promotion of healthy lifestyles, emphasize dietary and physical activity interventions, and implement early screening for metabolic risk factors in individuals over 30 years of age. Furthermore, air pollution deserves attention as a potential risk factor for liver disease (24). Moving forward, targeted health education should be advanced to address the continued rise in the burden of CLD (4).

With improved access to HBV vaccines globally, implementation of mother-to-child transmission prevention programs, and approval of antiviral medications for children and adolescents, the incidence of chronic viral hepatitis has significantly reduced (25, 26). This is consistent with our findings, although the decline was slower in low SDIR. In low SDIR, limited access to antiviral medications (27), challenges in standardizing screening and diagnosis, and low HBV vaccine coverage exacerbate the prevention and treatment burden of liver diseases among WCBA (28). As of 2016, nearly half of children aged 1 to 6 in India had not been administered the hepatitis B vaccine (29). Expanding screening for HBV, increasing vaccine coverage, and improving access to antiviral drugs are crucial in these regions. In the present study, the improvements in birth cohort effects, particularly for those born after 1980–1989, can be attributed to the widespread introduction of HBV vaccines, which have successfully reduced the risk of chronic viral hepatitis in subsequent generations (30). The WHO has recommended routine HBV immunization for newborns worldwide and has been advocating for the inclusion of HBV vaccines in national immunization programs since 1992. Surveys indicated that between 1990 and 2019, the prevalence of HBV surface antigen among infants and children under the age of five decreased by 77% (3). Additionally, significant improvements in healthcare services for newborns have occurred alongside social progress. However, this positive trend contrasts with the growing burden of metabolic CLD, especially NAFLD, which has increasingly affected younger populations. The growing prevalence of NAFLD highlights the need for concentration on preventive strategies, including promoting healthier dietary habits and lifestyles, especially in the younger population.

This study has several limitations. Firstly, while this study outlined the prevalence trends of CLD among WCBA, remarkable socio-economic and clinical changes over the past three decades might influence the impact of different types of CLD, a factor that was not explored in this study. Secondly, in certain underdeveloped countries with limited healthcare infrastructure, misdiagnosis and underdiagnosis might contribute to an underestimation of CLD prevalence. The extensive statistical modeling employed by the GBD collaborators might also influence the precision of the age, period, and birth cohort effect estimates. Thirdly, the delayed nature of the GBD data should be considered. In conclusion, nearly half of the countries globally are experiencing an upward trend in CLD, with concurrent increases in associated risks, highlighting a critical shortage of resources allocated to CLD healthcare for WCBA. Notably, while HBV is declining, NAFLD is rapidly increasing. The consistent pattern of increasing CLD risk with age is not surprising, as the development of CLD is closely linked to cumulative exposure to risk factors. However, the divergent trends found across SDIRs, with high SDI countries experiencing more rapid increases in prevalence, demonstrate that socio-economic factors, including lifestyle and access to healthcare, play a pivotal role in shaping these patterns. While the APC model presents valuable insights into the trends of CLD prevalence, it is important to recognize its limitations. One known limitation is the recognition problem, where the model may not always accurately capture complex relationships between age, period, and cohort effects, especially in regions with incomplete or inconsistent data. Additionally, boundary effects may arise, particularly when the model does not adequately handle data at the extremes of age or time periods, potentially skewing results. The sensitivity of the model to input data quality is another critical factor, as inaccuracies in the underlying data can significantly influence the model's output. Critically, the model did not incorporate several important covariates that could significantly influence these trends. Factors, such as obesity rates, dietary patterns, alcohol consumption, and HBV vaccine coverage are well-established drivers of CLD and could provide a more detailed understanding of the observed prevalence trends. For example, the rise in NAFLD has been closely linked to increasing obesity and poor dietary habits, while the decline in HBV prevalence could highly be attributed to successful vaccination programs, varying in coverage across regions and time periods. Furthermore, the APC model did not account for pregnancy status, parity, or gestational age, which are critical factors in understanding the impact of CLD on maternal and fetal health. Given the significant effect of these factors on the prevalence and outcomes of CLD in women, future studies may benefit from incorporating them into more detailed models to better capture the unique dynamics of liver disease among pregnant women and in different reproductive stages. The absence of these covariates in the current model represents a limitation, as it may overlook important contributors to CLD trends, especially in the context of rapidly changing socio-economic and health conditions. Future research incorporating these factors will provide deeper insights into the interaction among lifestyle, public health interventions, and disease progression, potentially informing more targeted prevention and intervention strategies for WCBA globally.

## Data Availability

Publicly available datasets were analyzed in this study. This data can be found here: data of the study are accessible via the Institute for Health Metrics and Evaluation (IHME)'s online platform, found at: https://vizhub.healthdata.org/gbd-results/.
