# Supplementary material for: Temporal trends in prevalence of chronic liver disease among women of childbearing age from 1992 to 2021
Source: Front Glob Womens Health. 2026 Feb 24;7:1641073. doi: 10.3389/fgwh.2026.1641073 (PMC12971455; doi:10.3389/fgwh.2026.1641073)
Supplement: Supplementary file 1 [file Datasheet1.pdf]

Supplementary Materials

Temporal Trends in Prevalence of Chronic Liver Disease among Women of Childbearing Age from 1992 to 2021

Within the framework of the GBD 2021 study, the population with CLD was identified by querying "Cirrhosis and other chronic liver diseases". The etiology codes associated with CLD encompass "Chronic hepatitis B including cirrhosis", "Chronic hepatitis C including cirrhosis", "Nonalcoholic fatty liver disease including cirrhosis", "Cirrhosis due to alcohol", and "Cirrhosis due to other causes".

Table S1 The prevalence number and AS prevalence rates in 2021, as well as NDs of prevalence from 1992 to 2021 for CLD in WCBA across countries.

Location	Prevalence number	Age-standardized prevalence rate	Net Drift (%/year)
Afghanistan	1784142.98 (1600256.19, 1972078.59)	27903.03 (22419.73, 34059.39)	0.37 (0.36, 0.38)
Albania	126946.33 (112857.77, 144732.15)	19886.83 (15397.27, 25190.73)	-0.04 (-0.09, 0.01)
Algeria	3858768.40 (3471738.62, 4350160.35)	33007.76 (26354.32, 40734.70)	0.63 (0.61, 0.64)
American Samoa	2969.99 (2663.07, 3344.26)	25378.18 (20172.28, 31345.90)	-0.07 (-0.24, 0.11)
Andorra	2259.95 (1982.40, 2568.65)	10136.32 (7857.51, 12887.23)	0.31 (0.07, 0.55)
Angola	1829218.83 (1679670.94, 2002791.93)	25243.67 (21644.92, 29527.81)	-0.56 (-0.58, -0.55)
Antigua and Barbuda	4545.14 (3968.17, 5182.77)	17770.81 (13478.60, 22885.18)	-0.10 (-0.26, 0.06)
Argentina	1071287.35 (929872.24, 1235221.99)	8780.76 (6490.35, 11521.08)	0.75 (0.73, 0.77)
Armenia	192317.29 (170099.12, 220946.55)	24303.53 (19414.90, 30214.63)	0.30 (0.28, 0.33)
Australia	599444.09 (536001.54, 677770.76)	9393.21 (7457.44, 11703.10)	0.06 (0.01, 0.11)
Austria	213526.35 (186905.83, 245423.98)	10047.14 (7648.98, 12900.10)	0.69 (0.64, 0.74)
Azerbaijan	819196.96 (735211.02, 923285.67)	28565.88 (23420.73, 34385.55)	0.26 (0.24, 0.28)
Bahamas	19862.92 (17626.09, 22504.25)	17942.32 (13599.47, 23088.20)	0.44 (0.36, 0.52)
Bahrain	119948.00 (107618.31, 134132.73)	35714.37 (28298.58, 44035.26)	0.58 (0.54, 0.62)
Bangladesh	7742130.14 (6986952.52, 8634233.90)	17084.14 (13651.51, 21107.51)	-0.12 (-0.15, -0.09)
Barbados	12996.50 (11320.10, 14822.26)	17056.44 (13059.34, 21847.02)	0.15 (0.06, 0.25)
Belarus	302799.88 (269406.12, 337810.22)	12949.43 (10252.24, 16176.51)	-0.01 (-0.03, 0.01)
Belgium	247574.69 (218355.52, 280665.41)	9428.21 (7192.11, 12084.81)	0.66 (0.63, 0.69)
Belize	25147.32 (22388.19, 28406.20)	21729.91 (16919.44, 27242.31)	0.34 (0.26, 0.43)
Benin	631582.76 (566133.35, 711978.93)	21325.91 (17268.65, 25906.09)	0.12 (0.10, 0.14)

Bermuda	2890.98 (2519.66, 3314.64)	19179.56 (14433.59, 24862.84)	0.31 (0.11, 0.50)
Bhutan	39470.14 (35742.35, 44151.16)	19257.16 (15542.98, 23722.44)	-0.22 (-0.27, -0.16)
Bolivia (Plurinational State of)	422089.84 (376091.81, 480791.63)	13750.91 (10561.21, 17500.59)	0.36 (0.34, 0.39)
Bosnia and Herzegovina	170837.29 (150895.08, 194729.11)	21445.15 (16508.57, 27227.48)	0.47 (0.45, 0.50)
Botswana	138793.06 (124635.59, 154903.73)	20381.34 (16458.04, 24909.47)	-0.48 (-0.59, -0.38)
Brazil	13004184.21 (11669172.08, 14655487.20)	21066.78 (16896.36, 26055.28)	0.15 (0.12, 0.18)
Brunei Darussalam	14960.73 (13267.28, 16754.70)	11567.11 (9090.38, 14439.09)	-0.34 (-0.47, -0.21)
Bulgaria	233272.36 (205551.28, 265550.78)	14519.26 (11181.51, 18488.88)	-0.34 (-0.42, -0.27)
Burkina Faso	1248151.04 (1142793.72, 1361040.11)	24307.49 (21024.05, 28155.47)	-0.38 (-0.40, -0.37)
Burundi	597717.04 (533488.12, 665276.79)	21078.55 (17274.92, 25526.58)	-0.15 (-0.17, -0.14)
Cabo Verde	37534.00 (33979.88, 41840.88)	25167.00 (20472.12, 30705.18)	-0.21 (-0.27, -0.15)
Cambodia	1026426.53 (932031.03, 1142796.56)	22916.42 (19442.11, 26900.27)	-0.71 (-0.72, -0.70)
Cameroon	1849649.85 (1692904.21, 2029515.42)	25306.58 (21151.35, 30121.15)	-0.26 (-0.27, -0.24)
Canada	772087.18 (695046.07, 876677.23)	8926.95 (7089.95, 11151.69)	-0.01 (-0.04, 0.02)
Central African Republic	344051.82 (317726.24, 374304.81)	25968.84 (22522.61, 30124.76)	-0.14 (-0.16, -0.12)
Chad	936871.31 (830049.60, 1048316.53)	25667.83 (21673.92, 30131.15)	-0.21 (-0.23, -0.20)
Chile	498279.64 (432717.76, 571998.89)	10126.95 (7565.92, 13349.23)	0.66 (0.64, 0.68)
China	80111725.57 (72607010.97, 89318900.94)	22441.55 (18542.32, 26993.95)	-0.81 (-0.91, -0.70)
Colombia	3123454.18 (2804478.22, 3494184.25)	23538.02 (18846.13, 29027.09)	-0.40 (-0.48, -0.32)
Comoros	45731.07 (41132.79, 51129.32)	24253.67 (19843.05, 29477.75)	-0.16 (-0.21, -0.11)
Congo	316104.88 (286755.65, 347810.28)	22568.55 (19033.58, 26600.63)	-0.28 (-0.30, -0.25)
Cook Islands	850.82 (752.60, 971.41)	19432.78 (14951.14, 24882.23)	-0.61 (-0.94, -0.29)

Costa Rica	270769.54 (237231.83, 306749.50)	20288.51 (15572.26, 25758.60)	0.22 (0.18, 0.26)
Croatia	147673.38 (129432.30, 168869.15)	22625.06 (19047.91, 26905.96)	-0.27 (-0.28, -0.25)
Cuba	499883.13 (436365.62, 571274.40)	15020.50 (11355.10, 19517.85)	0.18 (0.13, 0.22)
Cyprus	44410.34 (38425.75, 51546.71)	18544.44 (13997.35, 23974.90)	0.26 (0.21, 0.30)
Czechia	312685.17 (276662.90, 357234.68)	11072.97 (8289.05, 14590.51)	0.22 (0.16, 0.29)
Côte d'Ivoire	1432655.98 (1308139.13, 1583371.43)	12019.16 (9351.37, 15287.51)	0.29 (0.26, 0.32)
Democratic People's Republic of Korea	1832310.72 (1661294.35, 2038985.58)	26676.21 (22734.26, 31298.62)	-0.24 (-0.26, -0.22)
Democratic Republic of the Congo	6210899.29 (5683246.22, 6803514.74)	30450.08 (26489.22, 35116.88)	-0.19 (-0.20, -0.18)
Denmark	101808.70 (90901.55, 116431.23)	7733.35 (6103.94, 9688.94)	0.63 (0.60, 0.67)
Djibouti	72944.74 (65884.73, 81351.03)	22647.67 (18515.89, 27458.71)	0.17 (0.12, 0.22)
Dominica	3107.14 (2746.35, 3527.55)	18868.07 (14335.56, 24228.32)	0.18 (0.00, 0.36)
Dominican Republic	501437.34 (444043.44, 565487.13)	17510.37 (13803.64, 21759.73)	0.11 (0.10, 0.13)
Ecuador	1023215.58 (914074.81, 1159285.31)	21838.29 (16750.46, 27668.90)	0.31 (0.30, 0.33)
Egypt	9772939.94 (8779273.02, 10921338.48)	38628.73 (30913.40, 46866.11)	-0.14 (-0.22, -0.06)
El Salvador	381968.83 (341087.52, 430838.72)	21610.01 (16941.30, 27100.57)	0.20 (0.16, 0.24)
Equatorial Guinea	66809.99 (59829.41, 74363.15)	19491.68 (15660.88, 23971.96)	-0.45 (-0.51, -0.38)
Eritrea	328759.52 (295787.94, 366528.59)	21003.97 (17334.20, 25385.11)	-0.33 (-0.35, -0.31)
Estonia	36064.67 (32067.61, 40775.07)	11811.08 (9474.40, 14545.94)	-0.09 (-0.14, -0.04)
Eswatini	66701.59 (59777.68, 74567.43)	22540.21 (18332.02, 27611.16)	-0.50 (-0.57, -0.44)
Ethiopia	5057385.36 (4639841.02, 5582806.91)	19714.19 (16499.72, 23579.12)	-0.29 (-0.31, -0.26)
Fiji	56114.30 (50160.45, 63796.27)	24529.90 (19513.11, 30547.65)	-0.25 (-0.39, -0.10)
Finland	93295.03 (82903.90, 106256.97)	7842.53 (6123.33, 9901.13)	0.49 (0.45, 0.52)

France	1357401.71 (1195909.24, 1548401.63)	9175.27 (6986.48, 11841.03)	0.76 (0.71, 0.81)
Gabon	109882.84 (100111.94, 120477.47)	23521.70 (19568.85, 28310.82)	-0.24 (-0.28, -0.20)
Gambia	111545.17 (100359.45, 124897.54)	20373.08 (16461.42, 25121.88)	-0.77 (-0.95, -0.59)
Georgia	177522.60 (155081.64, 203123.73)	20897.63 (16188.08, 26358.37)	-0.08 (-0.10, -0.06)
Germany	1555599.59 (1362682.83, 1772851.73)	8515.83 (6451.22, 11034.58)	0.81 (0.79, 0.84)
Ghana	2345209.04 (2162403.45, 2564306.96)	26845.29 (22685.12, 31589.00)	-0.51 (-0.54, -0.48)
Greece	248583.19 (220691.67, 282361.20)	10259.20 (7917.49, 13101.43)	0.29 (0.25, 0.32)
Greenland	1303.44 (1157.62, 1466.03)	10126.36 (8038.82, 12795.89)	0.20 (-0.06, 0.46)
Grenada	4453.67 (3966.32, 5095.34)	17264.77 (13143.88, 22256.25)	0.09 (-0.06, 0.25)
Guam	7137.70 (6404.03, 8027.85)	19265.79 (15379.63, 23892.23)	-0.31 (-0.43, -0.20)
Guatemala	971406.53 (866666.02, 1085285.81)	23303.52 (18670.05, 28802.90)	0.04 (0.00, 0.08)
Guinea	947394.44 (854859.77, 1047718.68)	30138.08 (25464.82, 35250.41)	-0.19 (-0.21, -0.17)
Guinea-Bissau	140016.52 (125816. 24, 155909.58)	27830.40 (23438.55, 32656.46)	-0.13 (-0.16, -0.10)
Guyana	36257.54 (32427.23, 41169.05)	18213.83 (14288.92, 22831.31)	0.23 (0.17, 0.28)
Haiti	654639.78 (583472.58, 738090.71)	18912.48 (15105.79, 23304.90)	0.02 (0.01, 0.04)
Honduras	659128.97 (591499.44, 733431.29)	24476.96 (19560.04, 30178.79)	-0.02 (-0.09, 0.06)
Hungary	364758.24 (321707.35, 418431.63)	15294.98 (11635.41, 19698.60)	0.34 (0.31, 0.37)
Iceland	7233.12 (6370.66, 8177.62)	8778.20 (6701.62, 11306.65)	0.51 (0.38, 0.63)
India	68086361.70 (61837792.44, 75603981.91)	18208.67 (14882.59, 22127.19)	0.02 (-0.01, 0.05)
Indonesia	18341447.54 (16661533.41, 20463741.69)	23657.19 (19478.40, 28557.08)	-0.23 (-0.28, -0.19)
Iran (Islamic Republic of)	8997396.18 (8081677.82, 10120893.11)	35632.69 (28333.97, 43688.29)	0.68 (0.63, 0.74)
Iraq	3273819.29 (2945522.33, 3676896.18)	32364.84 (25597.67, 39765.46)	0.59 (0.57, 0.60)

Ireland	120148.22 (105711.74, 135937.21)	9688.88 (7452.35, 12413.09)	0.57 (0.53, 0.60)
Israel	297673.52 (259920.51, 340268.40)	13133.34 (9734.63, 17197.35)	0.67 (0.64, 0.70)
Italy	2077850.68 (1833019.70, 2351436.43)	15466.07 (11962.37, 19690.31)	0.37 (0.27, 0.47)
Jamaica	147044.57 (130343.61, 168221.18)	18927.95 (14469.87, 24186.90)	0.30 (0.27, 0.33)
Japan	1829328.26 (1634392.10, 2044517.73)	6874.37 (5579.04, 8473.07)	-0.27 (-0.30, -0.23)
Jordan	1079057.61 (971503.44, 1209522.59)	36443.10 (29061.31, 44654.03)	0.35 (0.30, 0.40)
Kazakhstan	972545.02 (862562.04, 1108632.74)	19607.54 (15629.37, 24407.04)	0.43 (0.41, 0.45)
Kenya	2822213.42 (2570987.74, 3140741.80)	23377.87 (19314.36, 28300.61)	-0.03 (-0.04, -0.01)
Kiribati	8662.46 (7810.59, 9679.35)	27980.45 (23214.90, 33542.03)	-0.35 (-0.47, -0.24)
Kuwait	619736.85 (551869.76, 703069.98)	37916.73 (29896.85, 46571.81)	0.72 (0.70, 0.74)
Kyrgyzstan	447583.54 (400228.85, 500761.28)	25943.83 (21371.33, 31197.95)	0.03 (0.01, 0.05)
Lao People's Democratic Republic	374930.21 (335950.53, 418591.41)	19294.22 (16002.41, 23131.30)	-0.46 (-0.48, -0.44)
Latvia	49685.73 (43802.48, 56407.19)	11494.67 (9178.03, 14311.72)	-0.12 (-0.16, -0.08)
Lebanon	523589.09 (467274.34, 589881.64)	33310.35 (26173.60, 41399.86)	0.60 (0.58, 0.62)
Lesotho	98783.35 (89161.32, 110069.47)	20765.50 (17244.57, 24895.00)	-0.13 (-0.16, -0.10)
Liberia	389936.61 (349760.75, 435302.92)	29211.62 (24630.08, 34560.32)	-0.02 (-0.04, 0.00)
Libya	698135.80 (627073.20, 788736.29)	33817.37 (26705.55, 41736.15)	0.55 (0.53, 0.58)
Lithuania	68627.52 (61396.25, 76997.97)	11095.84 (8956.01, 13631.87)	-0.10 (-0.14, -0.07)
Luxembourg	15519.79 (13648.94, 17688.28)	9214.47 (7038.65, 11915.54)	0.51 (0.41, 0.60)
Madagascar	1568149.62 (1428170.21, 1709479.30)	23371.42 (19550.93, 27770.23)	-0.22 (-0.23, -0.20)
Malawi	956993.82 (864544.77, 1060666.99)	21361.43 (17619.70, 25973.47)	-0.31 (-0.33, -0.29)
Malaysia	2062347.27 (1844437.48, 2314181.31)	24498.24 (19640.03, 30249.75)	-0.04 (-0.09, 0.00)

Maldives	27293.07 (24274.90, 30624.86)	22406.81 (18132.99, 27372.76)	-0.56 (-0.66, -0.47)
Mali	1310648.72 (1186544.26, 1444489.11)	26560.53 (22211.65, 31646.63)	-0.18 (-0.20, -0.17)
Malta	11293.98 (9845.55, 12907.26)	10817.35 (8126.74, 13932.99)	0.45 (0.34, 0.55)
Marshall Islands	3073.15 (2759.17, 3435.13)	21236.83 (17223.77, 26110.57)	-0.30 (-0.49, -0.12)
Mauritania	337314.66 (310332.41, 368646.51)	33922.88 (28821.95, 39895.91)	-0.14 (-0.15, -0.12)
Mauritius	68531.49 (60669.34, 76943.87)	20740.44 (16356.85, 25609.14)	-0.02 (-0.06, 0.01)
Mexico	6971490.99 (6210429.28, 7861572.82)	19566.52 (15130.74, 24662.47)	0.29 (0.26, 0.32)
Micronesia (Federated States of)	5323.73 (4768.33, 5976.19)	21468.06 (17210.14, 26592.86)	-0.52 (-0.65, -0.39)
Monaco	773.76 (669.50, 892.11)	9958.01 (7482.32, 12966.97)	0.57 (0.16, 0.97)
Mongolia	241371.78 (208078.87, 275879.25)	27518.38 (22576.39, 32999.89)	-0.32 (-0.34, -0.30)
Montenegro	22560.19 (19787.23, 25821.67)	14708.26 (11014.32, 19191.21)	0.54 (0.47, 0.61)
Morocco	3144192.89 (2810428.53, 3523464.87)	31988.99 (25226.00, 39657.49)	0.66 (0.65, 0.67)
Mozambique	1504084.84 (1360625.77, 1671926.37)	22032.53 (18476.02, 26230.39)	-0.38 (-0.40, -0.37)
Myanmar	3207030.41 (2888306.06, 3592472.51)	21294.30 (17232.29, 26009.18)	0.02 (0.00, 0.04)
Namibia	123870.18 (113304.91, 137570.45)	19364.41 (16022.26, 23309.40)	-0.22 (-0.25, -0.19)
Nauru	530.98 (475.21, 599.74)	19833.51 (15804.30, 24484.35)	-0.68 (-1.08, -0.27)
Nepal	1267545.89 (1134217.97, 1435288.78)	14482.16 (11380.03, 18272.05)	0.11 (0.08, 0.14)
Netherlands	335139.36 (294737.39, 379201.17)	8745.05 (6632.68, 11373.96)	0.65 (0.62, 0.67)
New Zealand	124538.27 (111108.94, 139340.01)	9983.69 (8081.73, 12181.50)	0.00 (-0.09, 0.08)
Nicaragua	382625.53 (338926.98, 433718.72)	21375.71 (16670.45, 26967.58)	0.20 (0.17, 0.24)
Niger	1609210.48 (1464813.15, 1764042.67)	32635.59 (28128.92, 37708.01)	-0.15 (-0.16, -0.14)
Nigeria	16185672.24 (14784895.03, 17727980.94)	30479.14 (26078.33, 35702.67)	-0.40 (-0.42, -0.39)

Niue	77.83 (69.19, 87.97)	19519.60 (15001.12, 24977.44)	-0.58 (-1.64, 0.50)
North Macedonia	108449.83 (95282.44, 124188.64)	18723.70 (14363.57, 23789.26)	0.60 (0.57, 0.63)
Northern Mariana Islands	2867.64 (2540.18, 3232.33)	23243.41 (18425.34, 28849.38)	-0.46 (-0.64, -0.29)
Norway	97318.64 (86433.74, 109809.52)	7633.17 (5911.16, 9674.96)	0.54 (0.51, 0.58)
Oman	363553.64 (325266.30, 409686.34)	34477.53 (27218.12, 42559.44)	0.59 (0.52, 0.67)
Pakistan	11973520.99 (10834901.95, 13405119.82)	20589.96 (16973.43, 24855.91)	0.09 (0.07, 0.11)
Palau	898.76 (803.77, 1009.55)	21373.06 (16838.38, 26618.05)	-0.43 (-0.76, -0.10)
Palestine	396935.74 (358686.54, 447435.67)	32798.25 (26023.72, 40613.41)	0.29 (0.26, 0.33)
Panama	213325.70 (189155.81, 241951.18)	19943.38 (15360.58, 25447.69)	0.34 (0.32, 0.37)
Papua New Guinea	630978.71 (576098.07, 698772.29)	24930.65 (21241.10, 29390.60)	-0.50 (-0.56, -0.44)
Paraguay	334748.64 (298042.16, 376779.52)	18020.73 (14058.89, 22679.25)	0.24 (0.22, 0.26)
Peru	1504748.64 (1346462.89, 1698814.99)	15415.99 (12169.20, 19197.79)	0.33 (0.31, 0.35)
Philippines	6841306.55 (6236112.29, 7552840.52)	24073.14 (20302.92, 28520.43)	-0.66 (-0.72, -0.61)
Poland	1263113.54 (1111851.15, 1429086.24)	12898.68 (10037.57, 16357.39)	0.16 (0.15, 0.18)
Portugal	331104.71 (290105.58, 385832.67)	12960.67 (9982.43, 16715.20)	0.70 (0.68, 0.73)
Puerto Rico	163985.33 (146159.75, 186596.50)	20498.11 (16011.43, 25776.11)	0.18 (0.16, 0.20)
Qatar	234694.86 (209237.88, 265370.38)	38587.44 (30656.13, 47234.67)	0.70 (0.66, 0.75)
Republic of Korea	1208158.94 (1075886.05, 1354722.04)	9386.35 (7329.36, 11983.76)	-0.74 (-0.86, -0.61)
Republic of Moldova	178800.86 (160512.06, 197944.06)	18033.71 (14960.09, 21710.50)	-0.52 (-0.57, -0.46)
Romania	707763.97 (624040.29, 800603.67)	15686.88 (12431.69, 19463.89)	-0.45 (-0.56, -0.33)
Russian Federation	5563472.11 (4961401.43, 6235240.38)	14887.09 (12002.47, 18240.15)	0.08 (0.05, 0.10)
Rwanda	567935.14 (507578.16, 640274.48)	17194.09 (14015.15, 20863.40)	-0.49 (-0.51, -0.47)

Saint Kitts and Nevis	3102.06 (2708.43, 3522.58)	18878.91 (14348.09, 24048.74)	0.08 (-0.12, 0.29)
Saint Lucia	7337.26 (6436.08, 8302.58)	15140.18 (11640.60, 19306.57)	0.03 (-0.09, 0.16)
Saint Vincent and the Grenadines	5007.95 (4433.44, 5686.52)	17506.54 (13355.04, 22471.81)	0.20 (0.06, 0.35)
Samoa	10295.35 (9226.52, 11536.42)	22482.44 (18082.73, 27628.49)	-0.30 (-0.39, -0.20)
San Marino	750.56 (653.08, 862.50)	9696.80 (7325.38, 12604.91)	0.50 (0.10, 0.89)
Sao Tome and Principe	13213.34 (12010.40, 14735.71)	24901.37 (20551.71, 30120.52)	-0.35 (-0.45, -0.25)
Saudi Arabia	3951894.52 (3540366.96, 4413091.99)	36716.63 (29148.13, 44825.45)	0.40 (0.31, 0.49)
Senegal	1011765.15 (916865.81, 1132216.04)	27956.40 (23082.85, 33495.35)	-0.10 (-0.11, -0.09)
Serbia	456756.09 (404170.16, 514960.20)	20952.08 (16250.79, 26501.28)	0.55 (0.53, 0.56)
Seychelles	5633.12 (4991.56, 6385.02)	21980.33 (17361.39, 27489.08)	-0.26 (-0.40, -0.11)
Sierra Leone	560933.99 (493361.21, 625791.20)	26110.05 (21606.27, 31026.93)	-0.18 (-0.19, -0.16)
Singapore	198198.42 (176303.01, 223140.04)	12063.48 (9515.22, 15117.28)	-0.45 (-0.53, -0.38)
Slovakia	167978.23 (148538.65, 191593.54)	11967.41 (9327.49, 15169.32)	0.21 (0.18, 0.25)
Slovenia	60798.22 (53636.94, 69157.52)	12880.81 (9917.96, 16469.91)	0.24 (0.20, 0.28)
Solomon Islands	36915.80 (33275.02, 41140.72)	22742.55 (18802.13, 27405.32)	-0.81 (-0.95, -0.68)
Somalia	1445956.65 (1330344.40, 1571771.91)	31826.48 (27456.34, 36838.08)	-0.03 (-0.04, -0.02)
South Africa	2854962.22 (2566594.26, 3221977.48)	18116.01 (14407.76, 22343.43)	-0.07 (-0.13, -0.01)
South Sudan	570773.38 (512975.46, 634404.91)	26274.20 (21824.51, 31482.03)	0.11 (0.09, 0.13)
Spain	1245983.06 (1091263.12, 1419004.95)	11274.77 (8631.95, 14447.25)	0.38 (0.33, 0.43)
Sri Lanka	1263914.15 (1128829.46, 1427803.49)	21729.67 (17137.41, 27037.27)	0.06 (0.04, 0.08)
Sudan	3021439.03 (2731805.09, 3377691.28)	28446.63 (23246.03, 34350.68)	0.13 (0.12, 0.14)
Suriname	26655.31 (23671.18, 30285.41)	17990.89 (13856.49, 22842.46)	0.32 (0.25, 0.39)

Sweden	188129.90 (166953.34, 213655.31)	8140.30 (6264.34, 10387.47)	0.66 (0.63, 0.68)
Switzerland	190272.12 (167484.60, 219969.52)	9068.94 (7005.37, 11598.73)	0.65 (0.63, 0.68)
Syrian Arab Republic	1256550.95 (1138258.09, 1407638.58)	33571.59 (26670.53, 41227.14)	0.38 (0.35, 0.42)
Taiwan (Province of China)	1510196.54 (1356437.21, 1695484.47)	23389.78 (19119.37, 28406.75)	-1.01 (-1.15, -0.86)
Tajikistan	666992.38 (593871.84, 747893.46)	26685.75 (21984.37, 31992.85)	0.06 (0.04, 0.08)
Thailand	3789540.45 (3379059.81, 4274259.97)	20993.25 (16778.08, 25979.33)	-0.44 (-0.55, -0.32)
Timor-Leste	74168.60 (66814.89, 82510.21)	23375.48 (19440.36, 28056.08)	-0.15 (-0.19, -0.11)
Togo	566784.96 (517004.63, 632153.57)	26998.38 (22785.20, 31945.88)	-0.03 (-0.05, -0.01)
Tokelau	67.67 (60.30, 75.85)	21007.40 (16708.96, 26100.53)	-0.31 (-1.48, 0.88)
Tonga	5417.69 (4856.62, 6130.57)	22590.53 (17958.04, 28251.63)	-0.83 (-0.98, -0.68)
Trinidad and Tobago	61801.23 (53999.21, 71151.06)	17075.50 (12940.82, 22108.89)	0.31 (0.27, 0.35)
Tunisia	1067282.08 (967055.38, 1200672.30)	32497.61 (25926.31, 39975.91)	0.17 (0.08, 0.25)
Turkmenistan	342371.71 (308260.21, 380510.89)	30112.40 (23736.01, 37315.32)	0.34 (0.31, 0.38)
Tuvalu	634.94 (572.00, 709.24)	27314.87 (22339.52, 32965.31)	0.21 (0.19, 0.23)
Turkey	6810093.59 (6148534.22, 7679191.92)	22914.15 (18845.15, 27824.90)	-0.21 (-0.6, 0.19)
Uganda	1788037.45 (1633775.87, 1971126.74)	19099.27 (15869.40, 23050.28)	-0.47 (-0.50, -0.44)
Ukraine	1618629.23 (1438475.58, 1822137.42)	14446.41 (11592.45, 17885.22)	0.43 (0.41, 0.45)
United Arab Emirates	668190.95 (586983.59, 758449.13)	33846.96 (26590.33, 41681.68)	0.92 (0.89, 0.94)
United Kingdom	1591271.81 (1420611.06, 1778328.04)	9884.88 (7865.35, 12248.36)	0.72 (0.68, 0.76)
United Republic of Tanzania	3055154.40 (2776014.82, 3400467.51)	22267.84 (18413.39, 26863.72)	-0.21 (-0.23, -0.19)
United States of America	7542841.36 (6772031.54, 8455505.17)	9647.10 (7579.64, 12123.75)	0.69 (0.65, 0.73)
United States Virgin Islands	3132.57 (2760.14, 3560.41)	16895.99 (13100.25, 21500.88)	0.27 (0.10, 0.44)

Uruguay	82428.16 (72213.56, 94610.70)	9527.56 (7119.81, 12482.65)	0.49 (0.46, 0.53)
Uzbekistan	2773203.44 (2511638.24, 3075189.14)	30436.13 (25503.42, 36179.01)	0.05 (0.03, 0.07)
Vanuatu	19372.02 (17506.82, 21554.54)	26095.34 (21530.85, 31357.66)	-0.28 (-0.37, -0.18)
Venezuela (Bolivarian Republic of)	1417869.37 (1244802.63, 1618409.42)	19757.37 (15120.83, 25118.87)	0.30 (0.28, 0.32)
Viet Nam	6704206.74 (6075306.09, 7350914.13)	24810.04 (20734.11, 29502.55)	-0.52 (-0.55, -0.49)
Yemen	2511710.83 (2293896.06, 2763055.97)	32141.87 (26598.11, 38489.50)	-0.08 (-0.11, -0.04)
Zambia	918710.70 (834454.22, 1021640.04)	20541.22 (16814.51, 24918.94)	-0.25 (-0.27, -0.23)
Zimbabwe	1153914.22 (1056327.39, 1266324.33)	30274.39 (26085.05, 34909.65)	-0.34 (-0.40, -0.28)

AS, age-standardized; CLD, chronic liver disease; ND, net drift; WCBA, women of childbearing age.

Table S2 The local drifts of prevalence from 1992 to 2021 for CLD in WCBA for seven age groups across SDI quintiles.

Location	Age	Local drift (%/year)
Global	15 to 19	-0.82 (-0.90, -0.75)
Global	20 to 24	-0.23 (-0.28, -0.18)
Global	25 to 29	0.05 (0.00, 0.09)
Global	30 to 34	0.22 (0.18, 0.26)
Global	35 to 39	0.31 (0.27, 0.35)
Global	40 to 44	0.37 (0.32, 0.42)
Global	45 to 49	0.45 (0.38, 0.51)
High SDI	15 to 19	-0.24 (-0.36, -0.11)
High SDI	20 to 24	0.22 (0.14, 0.30)
High SDI	25 to 29	0.67 (0.61, 0.74)
High SDI	30 to 34	0.95 (0.89, 1.00)
High SDI	35 to 39	0.99 (0.93, 1.04)
High SDI	40 to 44	0.94 (0.88, 1.00)
High SDI	45 to 49	0.96 (0.87, 1.04)
High-middle SDI	15 to 19	-1.66 (-1.91, -1.41)
High-middle SDI	20 to 24	-0.57 (-0.72, -0.42)
High-middle SDI	25 to 29	-0.08 (-0.19, 0.03)
High-middle SDI	30 to 34	0.06 (-0.04, 0.16)
High-middle SDI	35 to 39	0.16 (0.06, 0.25)
High-middle SDI	40 to 44	0.26 (0.15, 0.37)
High-middle SDI	45 to 49	0.40 (0.25, 0.54)
Middle SDI	15 to 19	-1.43 (-1.57, -1.29)

Middle SDI	20 to 24	-0.63 (-0.72, -0.53)
Middle SDI	25 to 29	-0.20 (-0.28, -0.13)
Middle SDI	30 to 34	-0.01 (-0.07, 0.06)
Middle SDI	35 to 39	0.05 (-0.02, 0.12)
Middle SDI	40 to 44	0.07 (-0.01, 0.15)
Middle SDI	45 to 49	0.12 (0.01, 0.22)
Low-middle SDI	15 to 19	-0.57 (-0.60, -0.54)
Low-middle SDI	20 to 24	-0.09 (-0.11, -0.07)
Low-middle SDI	25 to 29	0.08 (0.06, 0.09)
Low-middle SDI	30 to 34	0.13 (0.12, 0.15)
Low-middle SDI	35 to 39	0.13 (0.11, 0.15)
Low-middle SDI	40 to 44	0.14 (0.12, 0.16)
Low-middle SDI	45 to 49	0.16 (0.13, 0.19)
Low SDI	15 to 19	-0.69 (-0.72, -0.67)
Low SDI	20 to 24	-0.24 (-0.26, -0.23)
Low SDI	25 to 29	-0.12 (-0.13, -0.10)
Low SDI	30 to 34	-0.04 (-0.06, -0.02)
Low SDI	35 to 39	0.00 (-0.01, 0.02)
Low SDI	40 to 44	0.04 (0.02, 0.06)
Low SDI	45 to 49	0.05 (0.02, 0.08)

CLD, chronic liver disease; SDI, sociodemographic index; WCBA, women of childbearing age.

Table S3 The local drifts of prevalence from 1992 to 2021 for CLD in WCBA for seven age groups across countries.

Location	Age	Local drift (%/year)
Afghanistan	15 to 19	0.20 (0.18, 0.23)
Afghanistan	20 to 24	0.30 (0.28, 0.32)
Afghanistan	25 to 29	0.36 (0.35, 0.38)
Afghanistan	30 to 34	0.40 (0.38, 0.42)
Afghanistan	35 to 39	0.43 (0.41, 0.45)
Afghanistan	40 to 44	0.44 (0.42, 0.47)
Afghanistan	45 to 49	0.45 (0.42, 0.48)
Albania	15 to 19	-0.66 (-0.83, -0.49)
Albania	20 to 24	-0.37 (-0.47, -0.27)
Albania	25 to 29	-0.07 (-0.15, 0.01)
Albania	30 to 34	0.16 (0.09, 0.23)
Albania	35 to 39	0.18 (0.11, 0.25)
Albania	40 to 44	0.20 (0.13, 0.27)
Albania	45 to 49	0.23 (0.13, 0.32)
Algeria	15 to 19	0.21 (0.18, 0.24)
Algeria	20 to 24	0.60 (0.58, 0.62)
Algeria	25 to 29	0.66 (0.64, 0.68)
Algeria	30 to 34	0.69 (0.68, 0.71)
Algeria	35 to 39	0.71 (0.70, 0.73)
Algeria	40 to 44	0.73 (0.71, 0.74)
Algeria	45 to 49	0.74 (0.71, 0.77)
American Samoa	15 to 19	-1.27 (-1.77, -0.76)

American Samoa	20 to 24	-0.72 (-1.07, -0.37)
American Samoa	25 to 29	-0.03 (-0.30, 0.24)
American Samoa	30 to 34	0.32 (0.08, 0.56)
American Samoa	35 to 39	0.36 (0.12, 0.59)
American Samoa	40 to 44	0.37 (0.11, 0.63)
American Samoa	45 to 49	0.37 (0.01, 0.73)
Andorra	15 to 19	-0.79 (-1.56, -0.02)
Andorra	20 to 24	-0.09 (-0.60, 0.42)
Andorra	25 to 29	0.50 (0.12, 0.88)
Andorra	30 to 34	0.56 (0.24, 0.88)
Andorra	35 to 39	0.59 (0.29, 0.90)
Andorra	40 to 44	0.62 (0.28, 0.96)
Andorra	45 to 49	0.66 (0.20, 1.12)
Angola	15 to 19	-0.94 (-0.97, -0.91)
Angola	20 to 24	-0.68 (-0.70, -0.66)
Angola	25 to 29	-0.58 (-0.60, -0.56)
Angola	30 to 34	-0.51 (-0.53, -0.49)
Angola	35 to 39	-0.46 (-0.49, -0.43)
Angola	40 to 44	-0.42 (-0.45, -0.39)
Angola	45 to 49	-0.39 (-0.44, -0.35)
Antigua and Barbuda	15 to 19	-0.49 (-1.00, 0.02)
Antigua and Barbuda	20 to 24	-0.22 (-0.54, 0.10)
Antigua and Barbuda	25 to 29	-0.01 (-0.26, 0.23)
Antigua and Barbuda	30 to 34	0.00 (-0.22, 0.21)

Antigua and Barbuda	35 to 39	0.00 (-0.21, 0.21)
Antigua and Barbuda	40 to 44	0.00 (-0.24, 0.23)
Antigua and Barbuda	45 to 49	0.00 (-0.33, 0.33)
Argentina	15 to 19	0.41 (0.35, 0.48)
Argentina	20 to 24	0.67 (0.63, 0.72)
Argentina	25 to 29	0.78 (0.74, 0.81)
Argentina	30 to 34	0.80 (0.77, 0.83)
Argentina	35 to 39	0.83 (0.80, 0.86)
Argentina	40 to 44	0.86 (0.82, 0.89)
Argentina	45 to 49	0.88 (0.84, 0.93)
Armenia	15 to 19	-0.10 (-0.17, -0.03)
Armenia	20 to 24	0.22 (0.18, 0.27)
Armenia	25 to 29	0.36 (0.33, 0.39)
Armenia	30 to 34	0.39 (0.36, 0.42)
Armenia	35 to 39	0.40 (0.38, 0.43)
Armenia	40 to 44	0.41 (0.37, 0.44)
Armenia	45 to 49	0.40 (0.35, 0.44)
Australia	15 to 19	-1.15 (-1.31, -1.00)
Australia	20 to 24	-0.34 (-0.44, -0.24)
Australia	25 to 29	0.31 (0.23, 0.39)
Australia	30 to 34	0.34 (0.27, 0.41)
Australia	35 to 39	0.35 (0.27, 0.42)
Australia	40 to 44	0.36 (0.29, 0.44)
Australia	45 to 49	0.41 (0.31, 0.51)

Austria	15 to 19	0.14 (-0.02, 0.30)
Austria	20 to 24	0.56 (0.46, 0.66)
Austria	25 to 29	0.77 (0.69, 0.85)
Austria	30 to 34	0.83 (0.76, 0.90)
Austria	35 to 39	0.80 (0.74, 0.87)
Austria	40 to 44	0.82 (0.74, 0.89)
Austria	45 to 49	0.89 (0.79, 0.98)
Azerbaijan	15 to 19	-0.20 (-0.26, -0.14)
Azerbaijan	20 to 24	0.19 (0.15, 0.22)
Azerbaijan	25 to 29	0.29 (0.26, 0.32)
Azerbaijan	30 to 34	0.35 (0.32, 0.38)
Azerbaijan	35 to 39	0.38 (0.35, 0.41)
Azerbaijan	40 to 44	0.39 (0.35, 0.42)
Azerbaijan	45 to 49	0.36 (0.30, 0.42)
Bahamas	15 to 19	0.13 (-0.11, 0.37)
Bahamas	20 to 24	0.45 (0.30, 0.61)
Bahamas	25 to 29	0.50 (0.38, 0.63)
Bahamas	30 to 34	0.51 (0.40, 0.62)
Bahamas	35 to 39	0.50 (0.39, 0.61)
Bahamas	40 to 44	0.49 (0.37, 0.62)
Bahamas	45 to 49	0.48 (0.30, 0.65)
Bahrain	15 to 19	0.21 (0.11, 0.32)
Bahrain	20 to 24	0.36 (0.29, 0.43)
Bahrain	25 to 29	0.56 (0.50, 0.62)

Bahrain	30 to 34	0.72 (0.67, 0.78)
Bahrain	35 to 39	0.73 (0.68, 0.79)
Bahrain	40 to 44	0.72 (0.66, 0.79)
Bahrain	45 to 49	0.70 (0.59, 0.81)
Bangladesh	15 to 19	-0.77 (-0.84, -0.69)
Bangladesh	20 to 24	-0.19 (-0.24, -0.14)
Bangladesh	25 to 29	-0.09 (-0.13, -0.04)
Bangladesh	30 to 34	-0.02 (-0.07, 0.02)
Bangladesh	35 to 39	0.02 (-0.03, 0.07)
Bangladesh	40 to 44	0.05 (0.00, 0.11)
Bangladesh	45 to 49	0.08 (0.00, 0.17)
Barbados	15 to 19	-0.08 (-0.38, 0.22)
Barbados	20 to 24	0.12 (-0.07, 0.31)
Barbados	25 to 29	0.17 (0.02, 0.32)
Barbados	30 to 34	0.2 (0.07, 0.33)
Barbados	35 to 39	0.21 (0.09, 0.33)
Barbados	40 to 44	0.23 (0.10, 0.36)
Barbados	45 to 49	0.21 (0.04, 0.38)
Belarus	15 to 19	-0.82 (-0.87, -0.76)
Belarus	20 to 24	-0.23 (-0.27, -0.20)
Belarus	25 to 29	0.15 (0.12, 0.18)
Belarus	30 to 34	0.18 (0.15, 0.20)
Belarus	35 to 39	0.18 (0.16, 0.20)
Belarus	40 to 44	0.18 (0.16, 0.21)

Belarus	45 to 49	0.20 (0.16, 0.23)
Belgium	15 to 19	0.23 (0.13, 0.32)
Belgium	20 to 24	0.58 (0.52, 0.64)
Belgium	25 to 29	0.75 (0.70, 0.80)
Belgium	30 to 34	0.76 (0.72, 0.80)
Belgium	35 to 39	0.75 (0.71, 0.79)
Belgium	40 to 44	0.74 (0.70, 0.79)
Belgium	45 to 49	0.76 (0.70, 0.81)
Belize	15 to 19	-0.01 (-0.20, 0.19)
Belize	20 to 24	0.27 (0.13, 0.40)
Belize	25 to 29	0.41 (0.28, 0.53)
Belize	30 to 34	0.43 (0.31, 0.54)
Belize	35 to 39	0.44 (0.31, 0.56)
Belize	40 to 44	0.43 (0.29, 0.58)
Belize	45 to 49	0.41 (0.19, 0.63)
Benin	15 to 19	-0.48 (-0.52, -0.44)
Benin	20 to 24	0.15 (0.12, 0.18)
Benin	25 to 29	0.20 (0.18, 0.23)
Benin	30 to 34	0.22 (0.20, 0.25)
Benin	35 to 39	0.23 (0.20, 0.26)
Benin	40 to 44	0.23 (0.20, 0.27)
Benin	45 to 49	0.22 (0.17, 0.27)
Bermuda	15 to 19	0.10 (-0.60, 0.81)
Bermuda	20 to 24	0.32 (-0.11, 0.76)

Bermuda	25 to 29	0.34 (0.03, 0.66)
Bermuda	30 to 34	0.35 (0.09, 0.61)
Bermuda	35 to 39	0.35 (0.11, 0.58)
Bermuda	40 to 44	0.34 (0.10, 0.59)
Bermuda	45 to 49	0.32 (0.00, 0.63)
Bhutan	15 to 19	-1.72 (-1.86, -1.57)
Bhutan	20 to 24	-0.68 (-0.78, -0.58)
Bhutan	25 to 29	0.06 (-0.02, 0.14)
Bhutan	30 to 34	0.12 (0.04, 0.20)
Bhutan	35 to 39	0.15 (0.07, 0.24)
Bhutan	40 to 44	0.19 (0.09, 0.29)
Bhutan	45 to 49	0.22 (0.08, 0.36)
Bolivia (Plurinational State of)	15 to 19	0.30 (0.23, 0.37)
Bolivia (Plurinational State of)	20 to 24	0.38 (0.33, 0.43)
Bolivia (Plurinational State of)	25 to 29	0.40 (0.35, 0.44)
Bolivia (Plurinational State of)	30 to 34	0.38 (0.34, 0.42)
Bolivia (Plurinational State of)	35 to 39	0.38 (0.33, 0.42)
Bolivia (Plurinational State of)	40 to 44	0.36 (0.31, 0.41)
Bolivia (Plurinational State of)	45 to 49	0.35 (0.28, 0.42)
Bosnia and Herzegovina	15 to 19	0.25 (0.17, 0.34)
Bosnia and Herzegovina	20 to 24	0.40 (0.36, 0.45)
Bosnia and Herzegovina	25 to 29	0.47 (0.44, 0.51)
Bosnia and Herzegovina	30 to 34	0.52 (0.49, 0.55)
Bosnia and Herzegovina	35 to 39	0.54 (0.51, 0.57)

Bosnia and Herzegovina	40 to 44	0.55 (0.52, 0.58)
Bosnia and Herzegovina	45 to 49	0.56 (0.51, 0.60)
Botswana	15 to 19	-2.02 (-2.30, -1.75)
Botswana	20 to 24	-1.26 (-1.44, -1.07)
Botswana	25 to 29	-0.68 (-0.83, -0.52)
Botswana	30 to 34	0.01 (-0.14, 0.15)
Botswana	35 to 39	0.09 (-0.07, 0.24)
Botswana	40 to 44	0.13 (-0.05, 0.32)
Botswana	45 to 49	0.19 (-0.08, 0.46)
Brazil	15 to 19	-0.53 (-0.62, -0.43)
Brazil	20 to 24	-0.03 (-0.09, 0.03)
Brazil	25 to 29	0.25 (0.20, 0.30)
Brazil	30 to 34	0.30 (0.26, 0.34)
Brazil	35 to 39	0.32 (0.28, 0.37)
Brazil	40 to 44	0.34 (0.29, 0.39)
Brazil	45 to 49	0.35 (0.28, 0.41)
Brunei Darussalam	15 to 19	-0.99 (-1.37, -0.62)
Brunei Darussalam	20 to 24	-1.15 (-1.40, -0.90)
Brunei Darussalam	25 to 29	-1.01 (-1.21, -0.81)
Brunei Darussalam	30 to 34	-0.59 (-0.78, -0.41)
Brunei Darussalam	35 to 39	0.06 (-0.14, 0.25)
Brunei Darussalam	40 to 44	0.66 (0.44, 0.89)
Brunei Darussalam	45 to 49	0.69 (0.34, 1.03)
Bulgaria	15 to 19	-1.56 (-1.83, -1.29)

Bulgaria	20 to 24	-1.03 (-1.20, -0.85)
Bulgaria	25 to 29	-0.42 (-0.54, -0.30)
Bulgaria	30 to 34	0.08 (-0.02, 0.18)
Bulgaria	35 to 39	0.14 (0.04, 0.23)
Bulgaria	40 to 44	0.13 (0.03, 0.23)
Bulgaria	45 to 49	0.10 (-0.02, 0.23)
Burkina Faso	15 to 19	-0.82 (-0.85, -0.79)
Burkina Faso	20 to 24	-0.42 (-0.44, -0.40)
Burkina Faso	25 to 29	-0.36 (-0.38, -0.34)
Burkina Faso	30 to 34	-0.31 (-0.33, -0.29)
Burkina Faso	35 to 39	-0.28 (-0.30, -0.26)
Burkina Faso	40 to 44	-0.26 (-0.29, -0.23)
Burkina Faso	45 to 49	-0.25 (-0.29, -0.21)
Burundi	15 to 19	-0.94 (-0.97, -0.90)
Burundi	20 to 24	-0.14 (-0.16, -0.11)
Burundi	25 to 29	-0.06 (-0.08, -0.03)
Burundi	30 to 34	-0.02 (-0.04, 0.01)
Burundi	35 to 39	0.00 (-0.03, 0.02)
Burundi	40 to 44	0.00 (-0.03, 0.03)
Burundi	45 to 49	0.00 (-0.04, 0.05)
Cabo Verde	15 to 19	-1.34 (-1.48, -1.20)
Cabo Verde	20 to 24	-0.21 (-0.30, -0.12)
Cabo Verde	25 to 29	-0.09 (-0.17, -0.01)
Cabo Verde	30 to 34	-0.03 (-0.11, 0.05)

Cabo Verde	35 to 39	0.01 (-0.07, 0.10)
Cabo Verde	40 to 44	0.04 (-0.06, 0.15)
Cabo Verde	45 to 49	0.05 (-0.11, 0.21)
Cambodia	15 to 19	-1.91 (-1.94, -1.87)
Cambodia	20 to 24	-0.79 (-0.81, -0.77)
Cambodia	25 to 29	-0.63 (-0.65, -0.61)
Cambodia	30 to 34	-0.55 (-0.57, -0.53)
Cambodia	35 to 39	-0.47 (-0.49, -0.45)
Cambodia	40 to 44	-0.38 (-0.41, -0.36)
Cambodia	45 to 49	-0.33 (-0.36, -0.29)
Cameroon	15 to 19	-0.96 (-1.00, -0.93)
Cameroon	20 to 24	-0.35 (-0.38, -0.32)
Cameroon	25 to 29	-0.22 (-0.25, -0.20)
Cameroon	30 to 34	-0.15 (-0.17, -0.12)
Cameroon	35 to 39	-0.09 (-0.12, -0.06)
Cameroon	40 to 44	-0.05 (-0.09, -0.01)
Cameroon	45 to 49	-0.03 (-0.09, 0.02)
Canada	15 to 19	-0.25 (-0.34, -0.16)
Canada	20 to 24	-0.09 (-0.15, -0.03)
Canada	25 to 29	0.02 (-0.03, 0.07)
Canada	30 to 34	0.03 (-0.01, 0.08)
Canada	35 to 39	0.02 (-0.03, 0.06)
Canada	40 to 44	0.04 (-0.01, 0.09)
Canada	45 to 49	0.13 (0.06, 0.19)

Central African Republic	15 to 19	-0.34 (-0.38, -0.30)
Central African Republic	20 to 24	-0.24 (-0.27, -0.21)
Central African Republic	25 to 29	-0.16 (-0.19, -0.14)
Central African Republic	30 to 34	-0.11 (-0.14, -0.08)
Central African Republic	35 to 39	-0.07 (-0.10, -0.03)
Central African Republic	40 to 44	-0.03 (-0.07, 0.00)
Central African Republic	45 to 49	-0.02 (-0.07, 0.04)
Chad	15 to 19	-0.37 (-0.40, -0.34)
Chad	20 to 24	-0.31 (-0.33, -0.28)
Chad	25 to 29	-0.24 (-0.27, -0.22)
Chad	30 to 34	-0.19 (-0.22, -0.17)
Chad	35 to 39	-0.15 (-0.18, -0.12)
Chad	40 to 44	-0.12 (-0.16, -0.09)
Chad	45 to 49	-0.12 (-0.17, -0.07)
Chile	15 to 19	0.41 (0.35, 0.47)
Chile	20 to 24	0.61 (0.57, 0.65)
Chile	25 to 29	0.68 (0.65, 0.71)
Chile	30 to 34	0.71 (0.68, 0.74)
Chile	35 to 39	0.72 (0.70, 0.75)
Chile	40 to 44	0.73 (0.69, 0.76)
Chile	45 to 49	0.73 (0.69, 0.77)
China	15 to 19	-2.98 (-3.36, -2.60)
China	20 to 24	-1.22 (-1.43, -1.01)
China	25 to 29	-0.53 (-0.67, -0.38)

China	30 to 34	-0.37 (-0.51, -0.24)
China	35 to 39	-0.30 (-0.44, -0.17)
China	40 to 44	-0.24 (-0.39, -0.10)
China	45 to 49	-0.18 (-0.38, 0.02)
Colombia	15 to 19	-2.16 (-2.38, -1.94)
Colombia	20 to 24	-1.24 (-1.38, -1.09)
Colombia	25 to 29	-0.48 (-0.60, -0.36)
Colombia	30 to 34	0.14 (0.03, 0.25)
Colombia	35 to 39	0.21 (0.10, 0.32)
Colombia	40 to 44	0.26 (0.13, 0.39)
Colombia	45 to 49	0.30 (0.11, 0.49)
Comoros	15 to 19	-1.06 (-1.19, -0.93)
Comoros	20 to 24	-0.14 (-0.23, -0.05)
Comoros	25 to 29	-0.07 (-0.14, 0.01)
Comoros	30 to 34	-0.02 (-0.10, 0.05)
Comoros	35 to 39	0.02 (-0.06, 0.10)
Comoros	40 to 44	0.04 (-0.05, 0.13)
Comoros	45 to 49	0.06 (-0.07, 0.19)
Congo	15 to 19	-0.60 (-0.65, -0.56)
Congo	20 to 24	-0.43 (-0.47, -0.40)
Congo	25 to 29	-0.31 (-0.34, -0.28)
Congo	30 to 34	-0.23 (-0.26, -0.19)
Congo	35 to 39	-0.16 (-0.20, -0.13)
Congo	40 to 44	-0.12 (-0.16, -0.08)

Congo	45 to 49	-0.10 (-0.16, -0.05)
Cook Islands	15 to 19	-1.85 (-2.88, -0.81)
Cook Islands	20 to 24	-1.38 (-2.03, -0.72)
Cook Islands	25 to 29	-0.87 (-1.38, -0.36)
Cook Islands	30 to 34	-0.41 (-0.86, 0.04)
Cook Islands	35 to 39	-0.03 (-0.46, 0.41)
Cook Islands	40 to 44	0.04 (-0.42, 0.50)
Cook Islands	45 to 49	0.08 (-0.56, 0.71)
Costa Rica	15 to 19	-0.23 (-0.34, -0.12)
Costa Rica	20 to 24	0.08 (0.01, 0.15)
Costa Rica	25 to 29	0.27 (0.22, 0.33)
Costa Rica	30 to 34	0.31 (0.25, 0.36)
Costa Rica	35 to 39	0.33 (0.27, 0.38)
Costa Rica	40 to 44	0.35 (0.29, 0.41)
Costa Rica	45 to 49	0.37 (0.29, 0.46)
Côte d'Ivoire	15 to 19	-1.33 (-1.36, -1.30)
Côte d'Ivoire	20 to 24	-0.23 (-0.25, -0.20)
Côte d'Ivoire	25 to 29	-0.12 (-0.14, -0.11)
Côte d'Ivoire	30 to 34	-0.09 (-0.11, -0.07)
Côte d'Ivoire	35 to 39	-0.08 (-0.10, -0.05)
Côte d'Ivoire	40 to 44	-0.06 (-0.08, -0.03)
Côte d'Ivoire	45 to 49	-0.04 (-0.08, 0.00)
Croatia	15 to 19	-0.62 (-0.78, -0.46)
Croatia	20 to 24	-0.08 (-0.17, 0.02)

Croatia	25 to 29	0.30 (0.23, 0.37)
Croatia	30 to 34	0.37 (0.31, 0.43)
Croatia	35 to 39	0.38 (0.33, 0.44)
Croatia	40 to 44	0.41 (0.35, 0.46)
Croatia	45 to 49	0.39 (0.32, 0.47)
Cuba	15 to 19	-0.05 (-0.20, 0.11)
Cuba	20 to 24	0.06 (-0.03, 0.16)
Cuba	25 to 29	0.19 (0.12, 0.26)
Cuba	30 to 34	0.33 (0.26, 0.39)
Cuba	35 to 39	0.42 (0.36, 0.48)
Cuba	40 to 44	0.42 (0.35, 0.48)
Cuba	45 to 49	0.40 (0.32, 0.48)
Cyprus	15 to 19	-0.59 (-0.80, -0.39)
Cyprus	20 to 24	-0.27 (-0.39, -0.14)
Cyprus	25 to 29	0.09 (-0.01, 0.18)
Cyprus	30 to 34	0.40 (0.32, 0.49)
Cyprus	35 to 39	0.58 (0.50, 0.67)
Cyprus	40 to 44	0.62 (0.53, 0.71)
Cyprus	45 to 49	0.64 (0.52, 0.76)
Czechia	15 to 19	-0.24 (-0.34, -0.13)
Czechia	20 to 24	0.29 (0.22, 0.35)
Czechia	25 to 29	0.38 (0.33, 0.43)
Czechia	30 to 34	0.38 (0.34, 0.42)
Czechia	35 to 39	0.38 (0.34, 0.42)

Czechia	40 to 44	0.40 (0.36, 0.44)
Czechia	45 to 49	0.43 (0.38, 0.48)
Democratic People's Republic of Korea	15 to 19	-1.68 (-1.74, -1.62)
Democratic People's Republic of Korea	20 to 24	-0.19 (-0.23, -0.15)
Democratic People's Republic of Korea	25 to 29	-0.1 (-0.13, -0.07)
Democratic People's Republic of Korea	30 to 34	-0.03 (-0.06, 0.00)
Democratic People's Republic of Korea	35 to 39	0.03 (0.00, 0.06)
Democratic People's Republic of Korea	40 to 44	0.09 (0.05, 0.12)
Democratic People's Republic of Korea	45 to 49	0.13 (0.08, 0.17)
Democratic Republic of the Congo	15 to 19	-0.45 (-0.48, -0.42)
Democratic Republic of the Congo	20 to 24	-0.29 (-0.31, -0.27)
Democratic Republic of the Congo	25 to 29	-0.19 (-0.21, -0.17)
Democratic Republic of the Congo	30 to 34	-0.13 (-0.16, -0.11)
Democratic Republic of the Congo	35 to 39	-0.11 (-0.13, -0.08)
Democratic Republic of the Congo	40 to 44	-0.09 (-0.12, -0.06)
Democratic Republic of the Congo	45 to 49	-0.09 (-0.13, -0.05)
Denmark	15 to 19	0.50 (0.40, 0.61)
Denmark	20 to 24	0.58 (0.51, 0.65)
Denmark	25 to 29	0.65 (0.59, 0.70)
Denmark	30 to 34	0.68 (0.63, 0.73)
Denmark	35 to 39	0.68 (0.63, 0.73)
Denmark	40 to 44	0.65 (0.60, 0.70)
Denmark	45 to 49	0.68 (0.62, 0.74)
Djibouti	15 to 19	-0.09 (-0.20, 0.02)

Djibouti	20 to 24	0.06 (-0.02, 0.14)
Djibouti	25 to 29	0.15 (0.08, 0.22)
Djibouti	30 to 34	0.20 (0.13, 0.28)
Djibouti	35 to 39	0.25 (0.17, 0.32)
Djibouti	40 to 44	0.28 (0.18, 0.37)
Djibouti	45 to 49	0.30 (0.16, 0.43)
Dominica	15 to 19	0.06 (-0.47, 0.59)
Dominica	20 to 24	0.15 (-0.20, 0.51)
Dominica	25 to 29	0.20 (-0.08, 0.48)
Dominica	30 to 34	0.20 (-0.05, 0.46)
Dominica	35 to 39	0.20 (-0.05, 0.46)
Dominica	40 to 44	0.20 (-0.07, 0.48)
Dominica	45 to 49	0.20 (-0.16, 0.57)
Dominican Republic	15 to 19	-0.45 (-0.50, -0.40)
Dominican Republic	20 to 24	-0.16 (-0.19, -0.13)
Dominican Republic	25 to 29	0.06 (0.04, 0.09)
Dominican Republic	30 to 34	0.23 (0.20, 0.25)
Dominican Republic	35 to 39	0.30 (0.28, 0.33)
Dominican Republic	40 to 44	0.36 (0.34, 0.39)
Dominican Republic	45 to 49	0.41 (0.37, 0.45)
Ecuador	15 to 19	0.14 (0.09, 0.18)
Ecuador	20 to 24	0.32 (0.30, 0.35)
Ecuador	25 to 29	0.36 (0.33, 0.38)
Ecuador	30 to 34	0.34 (0.32, 0.37)

Ecuador	35 to 39	0.33 (0.31, 0.35)
Ecuador	40 to 44	0.32 (0.30, 0.35)
Ecuador	45 to 49	0.34 (0.30, 0.37)
Egypt	15 to 19	-0.68 (-0.89, -0.47)
Egypt	20 to 24	-0.38 (-0.53, -0.23)
Egypt	25 to 29	-0.14 (-0.26, -0.01)
Egypt	30 to 34	0.04 (-0.08, 0.15)
Egypt	35 to 39	0.02 (-0.10, 0.14)
Egypt	40 to 44	0.03 (-0.10, 0.17)
Egypt	45 to 49	0.04 (-0.15, 0.23)
El Salvador	15 to 19	-0.43 (-0.55, -0.31)
El Salvador	20 to 24	0.03 (-0.05, 0.11)
El Salvador	25 to 29	0.28 (0.21, 0.35)
El Salvador	30 to 34	0.32 (0.26, 0.38)
El Salvador	35 to 39	0.36 (0.29, 0.42)
El Salvador	40 to 44	0.38 (0.31, 0.46)
El Salvador	45 to 49	0.40 (0.30, 0.50)
Equatorial Guinea	15 to 19	-0.95 (-1.07, -0.82)
Equatorial Guinea	20 to 24	-0.69 (-0.79, -0.60)
Equatorial Guinea	25 to 29	-0.51 (-0.60, -0.42)
Equatorial Guinea	30 to 34	-0.38 (-0.47, -0.29)
Equatorial Guinea	35 to 39	-0.28 (-0.38, -0.18)
Equatorial Guinea	40 to 44	-0.20 (-0.32, -0.08)
Equatorial Guinea	45 to 49	-0.15 (-0.31, 0.02)

Eritrea	15 to 19	-1.73 (-1.78, -1.68)
Eritrea	20 to 24	-0.23 (-0.26, -0.19)
Eritrea	25 to 29	-0.16 (-0.19, -0.13)
Eritrea	30 to 34	-0.12 (-0.15, -0.09)
Eritrea	35 to 39	-0.09 (-0.12, -0.05)
Eritrea	40 to 44	-0.06 (-0.10, -0.02)
Eritrea	45 to 49	-0.04 (-0.10, 0.01)
Estonia	15 to 19	-0.98 (-1.15, -0.81)
Estonia	20 to 24	-0.42 (-0.53, -0.31)
Estonia	25 to 29	0.08 (0.00, 0.16)
Estonia	30 to 34	0.13 (0.06, 0.20)
Estonia	35 to 39	0.16 (0.09, 0.22)
Estonia	40 to 44	0.15 (0.08, 0.22)
Estonia	45 to 49	0.13 (0.04, 0.22)
Eswatini	15 to 19	-2.25 (-2.42, -2.09)
Eswatini	20 to 24	-1.05 (-1.15, -0.94)
Eswatini	25 to 29	-0.21 (-0.30, -0.12)
Eswatini	30 to 34	-0.10 (-0.19, -0.01)
Eswatini	35 to 39	-0.06 (-0.16, 0.04)
Eswatini	40 to 44	-0.03 (-0.14, 0.09)
Eswatini	45 to 49	0.01 (-0.16, 0.17)
Ethiopia	15 to 19	-0.61 (-0.66, -0.56)
Ethiopia	20 to 24	-0.42 (-0.46, -0.38)
Ethiopia	25 to 29	-0.29 (-0.32, -0.25)

Ethiopia	30 to 34	-0.21 (-0.25, -0.17)
Ethiopia	35 to 39	-0.18 (-0.22, -0.14)
Ethiopia	40 to 44	-0.16 (-0.21, -0.11)
Ethiopia	45 to 49	-0.18 (-0.25, -0.11)
Fiji	15 to 19	-1.49 (-1.96, -1.02)
Fiji	20 to 24	-1.05 (-1.35, -0.76)
Fiji	25 to 29	-0.54 (-0.76, -0.31)
Fiji	30 to 34	-0.02 (-0.22, 0.18)
Fiji	35 to 39	0.37 (0.17, 0.57)
Fiji	40 to 44	0.42 (0.20, 0.64)
Fiji	45 to 49	0.44 (0.14, 0.75)
Finland	15 to 19	0.35 (0.26, 0.45)
Finland	20 to 24	0.44 (0.37, 0.51)
Finland	25 to 29	0.51 (0.45, 0.56)
Finland	30 to 34	0.53 (0.48, 0.58)
Finland	35 to 39	0.52 (0.47, 0.56)
Finland	40 to 44	0.51 (0.46, 0.55)
Finland	45 to 49	0.53 (0.47, 0.59)
France	15 to 19	0.43 (0.29, 0.58)
France	20 to 24	0.68 (0.58, 0.78)
France	25 to 29	0.83 (0.75, 0.91)
France	30 to 34	0.89 (0.82, 0.96)
France	35 to 39	0.84 (0.77, 0.91)
France	40 to 44	0.81 (0.73, 0.88)

France	45 to 49	0.81 (0.72, 0.91)
Gabon	15 to 19	-0.91 (-1.00, -0.83)
Gabon	20 to 24	-0.35 (-0.41, -0.28)
Gabon	25 to 29	-0.22 (-0.28, -0.16)
Gabon	30 to 34	-0.15 (-0.21, -0.09)
Gabon	35 to 39	-0.10 (-0.16, -0.03)
Gabon	40 to 44	-0.05 (-0.12, 0.03)
Gabon	45 to 49	0.02 (-0.09, 0.14)
Gambia	15 to 19	-2.33 (-2.74, -1.92)
Gambia	20 to 24	-1.62 (-1.91, -1.33)
Gambia	25 to 29	-1.13 (-1.38, -0.87)
Gambia	30 to 34	-0.6 (-0.86, -0.35)
Gambia	35 to 39	0.02 (-0.26, 0.30)
Gambia	40 to 44	0.07 (-0.28, 0.42)
Gambia	45 to 49	0.07 (-0.44, 0.58)
Georgia	15 to 19	-0.53 (-0.60, -0.46)
Georgia	20 to 24	-0.17 (-0.22, -0.13)
Georgia	25 to 29	-0.03 (-0.06, 0.00)
Georgia	30 to 34	0.01 (-0.02, 0.04)
Georgia	35 to 39	0.03 (0.01, 0.06)
Georgia	40 to 44	0.05 (0.02, 0.08)
Georgia	45 to 49	0.04 (0.00, 0.08)
Germany	15 to 19	0.39 (0.30, 0.48)
Germany	20 to 24	0.61 (0.55, 0.67)

Germany	25 to 29	0.84 (0.80, 0.88)
Germany	30 to 34	0.96 (0.92, 1.00)
Germany	35 to 39	0.97 (0.93, 1.00)
Germany	40 to 44	0.94 (0.90, 0.98)
Germany	45 to 49	0.92 (0.87, 0.97)
Ghana	15 to 19	-2.24 (-2.32, -2.17)
Ghana	20 to 24	-0.63 (-0.69, -0.58)
Ghana	25 to 29	-0.34 (-0.38, -0.29)
Ghana	30 to 34	-0.23 (-0.28, -0.19)
Ghana	35 to 39	-0.15 (-0.20, -0.09)
Ghana	40 to 44	-0.08 (-0.14, -0.01)
Ghana	45 to 49	-0.02 (-0.11, 0.07)
Greece	15 to 19	-0.89 (-1.01, -0.76)
Greece	20 to 24	-0.18 (-0.26, -0.10)
Greece	25 to 29	0.42 (0.36, 0.48)
Greece	30 to 34	0.53 (0.48, 0.58)
Greece	35 to 39	0.59 (0.54, 0.64)
Greece	40 to 44	0.66 (0.61, 0.71)
Greece	45 to 49	0.75 (0.69, 0.82)
Greenland	15 to 19	0.20 (-0.51, 0.92)
Greenland	20 to 24	0.18 (-0.31, 0.67)
Greenland	25 to 29	0.18 (-0.21, 0.57)
Greenland	30 to 34	0.18 (-0.19, 0.54)
Greenland	35 to 39	0.18 (-0.20, 0.55)

Greenland	40 to 44	0.21 (-0.22, 0.65)
Greenland	45 to 49	0.30 (-0.27, 0.88)
Grenada	15 to 19	-0.42 (-0.87, 0.03)
Grenada	20 to 24	-0.04 (-0.32, 0.25)
Grenada	25 to 29	0.17 (-0.06, 0.41)
Grenada	30 to 34	0.20 (-0.02, 0.42)
Grenada	35 to 39	0.22 (0.00, 0.44)
Grenada	40 to 44	0.23 (-0.01, 0.48)
Grenada	45 to 49	0.23 (-0.10, 0.57)
Guam	15 to 19	-1.52 (-1.88, -1.16)
Guam	20 to 24	-1.10 (-1.33, -0.86)
Guam	25 to 29	-0.50 (-0.68, -0.31)
Guam	30 to 34	-0.07 (-0.24, 0.09)
Guam	35 to 39	0.24 (0.08, 0.39)
Guam	40 to 44	0.30 (0.13, 0.46)
Guam	45 to 49	0.35 (0.13, 0.57)
Guatemala	15 to 19	-0.44 (-0.53, -0.35)
Guatemala	20 to 24	-0.08 (-0.14, -0.01)
Guatemala	25 to 29	0.03 (-0.03, 0.09)
Guatemala	30 to 34	0.11 (0.05, 0.17)
Guatemala	35 to 39	0.17 (0.11, 0.23)
Guatemala	40 to 44	0.21 (0.14, 0.28)
Guatemala	45 to 49	0.26 (0.16, 0.36)
Guinea	15 to 19	-0.38 (-0.42, -0.34)

Guinea	20 to 24	-0.28 (-0.31, -0.25)
Guinea	25 to 29	-0.22 (-0.24, -0.19)
Guinea	30 to 34	-0.16 (-0.19, -0.14)
Guinea	35 to 39	-0.13 (-0.15, -0.10)
Guinea	40 to 44	-0.10 (-0.14, -0.07)
Guinea	45 to 49	-0.09 (-0.14, -0.04)
Guinea-Bissau	15 to 19	-0.26 (-0.32, -0.20)
Guinea-Bissau	20 to 24	-0.17 (-0.21, -0.12)
Guinea-Bissau	25 to 29	-0.12 (-0.17, -0.08)
Guinea-Bissau	30 to 34	-0.10 (-0.15, -0.06)
Guinea-Bissau	35 to 39	-0.08 (-0.13, -0.03)
Guinea-Bissau	40 to 44	-0.08 (-0.14, -0.01)
Guinea-Bissau	45 to 49	-0.09 (-0.18, 0.00)
Guyana	15 to 19	-0.17 (-0.32, -0.03)
Guyana	20 to 24	0.22 (0.12, 0.31)
Guyana	25 to 29	0.28 (0.20, 0.36)
Guyana	30 to 34	0.30 (0.22, 0.37)
Guyana	35 to 39	0.30 (0.23, 0.38)
Guyana	40 to 44	0.30 (0.22, 0.39)
Guyana	45 to 49	0.32 (0.20, 0.43)
Haiti	15 to 19	-0.13 (-0.16, -0.09)
Haiti	20 to 24	-0.04 (-0.06, -0.01)
Haiti	25 to 29	0.02 (0.00, 0.04)
Haiti	30 to 34	0.04 (0.02, 0.07)

Haiti	35 to 39	0.06 (0.04, 0.08)
Haiti	40 to 44	0.08 (0.05, 0.10)
Haiti	45 to 49	0.09 (0.06, 0.13)
Honduras	15 to 19	-0.86 (-1.04, -0.68)
Honduras	20 to 24	-0.24 (-0.36, -0.11)
Honduras	25 to 29	0.10 (-0.01, 0.20)
Honduras	30 to 34	0.15 (0.04, 0.26)
Honduras	35 to 39	0.19 (0.07, 0.30)
Honduras	40 to 44	0.22 (0.09, 0.36)
Honduras	45 to 49	0.24 (0.05, 0.44)
Hungary	15 to 19	0.26 (0.16, 0.37)
Hungary	20 to 24	0.31 (0.25, 0.38)
Hungary	25 to 29	0.39 (0.34, 0.44)
Hungary	30 to 34	0.38 (0.34, 0.42)
Hungary	35 to 39	0.36 (0.33, 0.40)
Hungary	40 to 44	0.34 (0.30, 0.38)
Hungary	45 to 49	0.34 (0.29, 0.39)
Iceland	15 to 19	0.55 (0.21, 0.89)
Iceland	20 to 24	0.45 (0.21, 0.69)
Iceland	25 to 29	0.47 (0.28, 0.67)
Iceland	30 to 34	0.49 (0.32, 0.67)
Iceland	35 to 39	0.52 (0.34, 0.69)
Iceland	40 to 44	0.53 (0.34, 0.72)
Iceland	45 to 49	0.55 (0.29, 0.81)

India	15 to 19	-0.28 (-0.35, -0.20)
India	20 to 24	-0.05 (-0.10, 0.00)
India	25 to 29	0.07 (0.02, 0.11)
India	30 to 34	0.10 (0.05, 0.14)
India	35 to 39	0.09 (0.04, 0.13)
India	40 to 44	0.08 (0.03, 0.14)
India	45 to 49	0.09 (0.02, 0.17)
Indonesia	15 to 19	-1.25 (-1.38, -1.12)
Indonesia	20 to 24	-0.72 (-0.81, -0.64)
Indonesia	25 to 29	-0.28 (-0.35, -0.21)
Indonesia	30 to 34	0.02 (-0.05, 0.08)
Indonesia	35 to 39	0.10 (0.04, 0.17)
Indonesia	40 to 44	0.18 (0.10, 0.25)
Indonesia	45 to 49	0.23 (0.13, 0.34)
Iran (Islamic Republic of)	15 to 19	0.20 (0.04, 0.37)
Iran (Islamic Republic of)	20 to 24	0.43 (0.32, 0.53)
Iran (Islamic Republic of)	25 to 29	0.67 (0.60, 0.75)
Iran (Islamic Republic of)	30 to 34	0.84 (0.77, 0.91)
Iran (Islamic Republic of)	35 to 39	0.86 (0.79, 0.94)
Iran (Islamic Republic of)	40 to 44	0.85 (0.76, 0.94)
Iran (Islamic Republic of)	45 to 49	0.84 (0.71, 0.97)
Iraq	15 to 19	0.48 (0.45, 0.51)
Iraq	20 to 24	0.52 (0.50, 0.54)
Iraq	25 to 29	0.58 (0.56, 0.60)

Iraq	30 to 34	0.62 (0.60, 0.64)
Iraq	35 to 39	0.63 (0.61, 0.65)
Iraq	40 to 44	0.64 (0.62, 0.66)
Iraq	45 to 49	0.63 (0.60, 0.66)
Ireland	15 to 19	0.48 (0.39, 0.56)
Ireland	20 to 24	0.52 (0.46, 0.59)
Ireland	25 to 29	0.55 (0.50, 0.60)
Ireland	30 to 34	0.57 (0.53, 0.62)
Ireland	35 to 39	0.58 (0.54, 0.63)
Ireland	40 to 44	0.61 (0.56, 0.66)
Ireland	45 to 49	0.63 (0.57, 0.70)
Israel	15 to 19	0.30 (0.22, 0.37)
Israel	20 to 24	0.40 (0.34, 0.45)
Israel	25 to 29	0.58 (0.54, 0.63)
Israel	30 to 34	0.75 (0.71, 0.80)
Israel	35 to 39	0.82 (0.78, 0.86)
Israel	40 to 44	0.88 (0.83, 0.92)
Israel	45 to 49	0.95 (0.89, 1.01)
Italy	15 to 19	-0.29 (-0.62, 0.04)
Italy	20 to 24	-0.15 (-0.36, 0.06)
Italy	25 to 29	0.13 (-0.04, 0.29)
Italy	30 to 34	0.42 (0.28, 0.56)
Italy	35 to 39	0.67 (0.54, 0.80)
Italy	40 to 44	0.88 (0.74, 1.01)

Italy	45 to 49	0.88 (0.70, 1.05)
Jamaica	15 to 19	-0.07 (-0.15, 0.01)
Jamaica	20 to 24	0.32 (0.27, 0.37)
Jamaica	25 to 29	0.35 (0.31, 0.39)
Jamaica	30 to 34	0.36 (0.32, 0.40)
Jamaica	35 to 39	0.37 (0.33, 0.41)
Jamaica	40 to 44	0.37 (0.32, 0.41)
Jamaica	45 to 49	0.36 (0.30, 0.43)
Japan	15 to 19	-0.21 (-0.31, -0.10)
Japan	20 to 24	-0.15 (-0.23, -0.08)
Japan	25 to 29	-0.16 (-0.22, -0.10)
Japan	30 to 34	-0.21 (-0.26, -0.15)
Japan	35 to 39	-0.29 (-0.34, -0.23)
Japan	40 to 44	-0.39 (-0.45, -0.34)
Japan	45 to 49	-0.47 (-0.53, -0.41)
Jordan	15 to 19	-0.47 (-0.58, -0.36)
Jordan	20 to 24	0.00 (-0.08, 0.09)
Jordan	25 to 29	0.45 (0.37, 0.52)
Jordan	30 to 34	0.56 (0.49, 0.64)
Jordan	35 to 39	0.61 (0.53, 0.69)
Jordan	40 to 44	0.62 (0.52, 0.71)
Jordan	45 to 49	0.62 (0.47, 0.76)
Kazakhstan	15 to 19	-0.01 (-0.06, 0.05)
Kazakhstan	20 to 24	0.33 (0.29, 0.37)

Kazakhstan	25 to 29	0.55 (0.52, 0.58)
Kazakhstan	30 to 34	0.55 (0.52, 0.58)
Kazakhstan	35 to 39	0.54 (0.51, 0.57)
Kazakhstan	40 to 44	0.52 (0.49, 0.55)
Kazakhstan	45 to 49	0.5 (0.46, 0.55)
Kenya	15 to 19	-1.13 (-1.17, -1.09)
Kenya	20 to 24	-0.03 (-0.06, 0.00)
Kenya	25 to 29	0.13 (0.10, 0.15)
Kenya	30 to 34	0.16 (0.13, 0.19)
Kenya	35 to 39	0.18 (0.15, 0.21)
Kenya	40 to 44	0.20 (0.17, 0.24)
Kenya	45 to 49	0.21 (0.16, 0.26)
Kiribati	15 to 19	-1.57 (-1.88, -1.26)
Kiribati	20 to 24	-1.09 (-1.30, -0.88)
Kiribati	25 to 29	-0.44 (-0.61, -0.27)
Kiribati	30 to 34	-0.09 (-0.25, 0.08)
Kiribati	35 to 39	0.17 (0.00, 0.34)
Kiribati	40 to 44	0.21 (0.01, 0.40)
Kiribati	45 to 49	0.21 (-0.07, 0.49)
Kuwait	15 to 19	0.68 (0.62, 0.75)
Kuwait	20 to 24	0.68 (0.64, 0.72)
Kuwait	25 to 29	0.71 (0.68, 0.74)
Kuwait	30 to 34	0.74 (0.72, 0.77)
Kuwait	35 to 39	0.76 (0.73, 0.79)

Kuwait	40 to 44	0.74 (0.70, 0.78)
Kuwait	45 to 49	0.72 (0.66, 0.78)
Kyrgyzstan	15 to 19	-0.51 (-0.56, -0.46)
Kyrgyzstan	20 to 24	-0.01 (-0.04, 0.02)
Kyrgyzstan	25 to 29	0.13 (0.11, 0.16)
Kyrgyzstan	30 to 34	0.15 (0.13, 0.18)
Kyrgyzstan	35 to 39	0.15 (0.12, 0.18)
Kyrgyzstan	40 to 44	0.14 (0.11, 0.18)
Kyrgyzstan	45 to 49	0.11 (0.06, 0.16)
Lao People's Democratic Republic	15 to 19	-1.35 (-1.40, -1.30)
Lao People's Democratic Republic	20 to 24	-0.61 (-0.64, -0.57)
Lao People's Democratic Republic	25 to 29	-0.47 (-0.50, -0.44)
Lao People's Democratic Republic	30 to 34	-0.35 (-0.38, -0.32)
Lao People's Democratic Republic	35 to 39	-0.26 (-0.29, -0.23)
Lao People's Democratic Republic	40 to 44	-0.17 (-0.21, -0.13)
Lao People's Democratic Republic	45 to 49	-0.10 (-0.16, -0.05)
Latvia	15 to 19	-1.41 (-1.56, -1.26)
Latvia	20 to 24	-0.50 (-0.59, -0.41)
Latvia	25 to 29	0.11 (0.05, 0.17)
Latvia	30 to 34	0.16 (0.10, 0.21)
Latvia	35 to 39	0.19 (0.14, 0.25)
Latvia	40 to 44	0.23 (0.17, 0.28)
Latvia	45 to 49	0.26 (0.19, 0.33)
Lebanon	15 to 19	0.24 (0.19, 0.30)

Lebanon	20 to 24	0.47 (0.43, 0.50)
Lebanon	25 to 29	0.66 (0.63, 0.69)
Lebanon	30 to 34	0.69 (0.66, 0.71)
Lebanon	35 to 39	0.70 (0.68, 0.73)
Lebanon	40 to 44	0.71 (0.68, 0.74)
Lebanon	45 to 49	0.70 (0.66, 0.74)
Lesotho	15 to 19	-1.07 (-1.15, -1.00)
Lesotho	20 to 24	-0.13 (-0.18, -0.07)
Lesotho	25 to 29	-0.04 (-0.09, 0.01)
Lesotho	30 to 34	0.02 (-0.03, 0.07)
Lesotho	35 to 39	0.06 (0.01, 0.11)
Lesotho	40 to 44	0.09 (0.03, 0.15)
Lesotho	45 to 49	0.11 (0.04, 0.19)
Liberia	15 to 19	-0.31 (-0.35, -0.27)
Liberia	20 to 24	-0.14 (-0.17, -0.11)
Liberia	25 to 29	-0.04 (-0.07, -0.01)
Liberia	30 to 34	0.03 (0.00, 0.06)
Liberia	35 to 39	0.08 (0.04, 0.11)
Liberia	40 to 44	0.09 (0.05, 0.13)
Liberia	45 to 49	0.10 (0.04, 0.16)
Libya	15 to 19	0.21 (0.14, 0.27)
Libya	20 to 24	0.46 (0.41, 0.50)
Libya	25 to 29	0.61 (0.57, 0.64)
Libya	30 to 34	0.63 (0.60, 0.67)

Libya	35 to 39	0.65 (0.61, 0.69)
Libya	40 to 44	0.65 (0.60, 0.69)
Libya	45 to 49	0.63 (0.57, 0.70)
Lithuania	15 to 19	-0.97 (-1.08, -0.86)
Lithuania	20 to 24	-0.46 (-0.53, -0.39)
Lithuania	25 to 29	0.04 (-0.01, 0.09)
Lithuania	30 to 34	0.10 (0.06, 0.15)
Lithuania	35 to 39	0.14 (0.10, 0.19)
Lithuania	40 to 44	0.16 (0.11, 0.21)
Lithuania	45 to 49	0.16 (0.10, 0.22)
Luxembourg	15 to 19	-0.17 (-0.47, 0.12)
Luxembourg	20 to 24	0.27 (0.08, 0.47)
Luxembourg	25 to 29	0.62 (0.47, 0.77)
Luxembourg	30 to 34	0.66 (0.52, 0.79)
Luxembourg	35 to 39	0.65 (0.52, 0.78)
Luxembourg	40 to 44	0.70 (0.56, 0.84)
Luxembourg	45 to 49	0.75 (0.56, 0.94)
Madagascar	15 to 19	-1.77 (-1.80, -1.74)
Madagascar	20 to 24	-0.15 (-0.17, -0.12)
Madagascar	25 to 29	-0.03 (-0.05, -0.01)
Madagascar	30 to 34	0.04 (0.02, 0.06)
Madagascar	35 to 39	0.08 (0.06, 0.10)
Madagascar	40 to 44	0.10 (0.07, 0.13)
Madagascar	45 to 49	0.10 (0.06, 0.14)

Malawi	15 to 19	-1.63 (-1.68, -1.59)
Malawi	20 to 24	-0.27 (-0.31, -0.24)
Malawi	25 to 29	-0.16 (-0.19, -0.13)
Malawi	30 to 34	-0.1 (-0.13, -0.07)
Malawi	35 to 39	-0.06 (-0.10, -0.03)
Malawi	40 to 44	-0.04 (-0.08, 0.00)
Malawi	45 to 49	-0.02 (-0.08, 0.04)
Malaysia	15 to 19	-0.73 (-0.86, -0.6)
Malaysia	20 to 24	-0.46 (-0.55, -0.38)
Malaysia	25 to 29	-0.21 (-0.28, -0.14)
Malaysia	30 to 34	0.06 (0.00, 0.12)
Malaysia	35 to 39	0.29 (0.22, 0.35)
Malaysia	40 to 44	0.33 (0.26, 0.41)
Malaysia	45 to 49	0.37 (0.26, 0.47)
Maldives	15 to 19	-1.96 (-2.22, -1.71)
Maldives	20 to 24	-1.21 (-1.37, -1.04)
Maldives	25 to 29	-0.59 (-0.72, -0.46)
Maldives	30 to 34	-0.22 (-0.34, -0.10)
Maldives	35 to 39	-0.12 (-0.26, 0.01)
Maldives	40 to 44	-0.03 (-0.20, 0.14)
Maldives	45 to 49	0.06 (-0.18, 0.31)
Mali	15 to 19	-1.00 (-1.04, -0.97)
Mali	20 to 24	-0.20 (-0.22, -0.17)
Mali	25 to 29	-0.10 (-0.13, -0.08)

Mali	30 to 34	-0.05 (-0.08, -0.03)
Mali	35 to 39	-0.02 (-0.05, 0.00)
Mali	40 to 44	0.01 (-0.02, 0.04)
Mali	45 to 49	0.04 (-0.01, 0.08)
Malta	15 to 19	-0.31 (-0.66, 0.05)
Malta	20 to 24	0.14 (-0.07, 0.35)
Malta	25 to 29	0.46 (0.30, 0.62)
Malta	30 to 34	0.59 (0.44, 0.73)
Malta	35 to 39	0.67 (0.54, 0.80)
Malta	40 to 44	0.73 (0.59, 0.87)
Malta	45 to 49	0.79 (0.62, 0.96)
Marshall Islands	15 to 19	-1.43 (-1.90, -0.95)
Marshall Islands	20 to 24	-0.94 (-1.28, -0.61)
Marshall Islands	25 to 29	-0.47 (-0.75, -0.19)
Marshall Islands	30 to 34	-0.04 (-0.31, 0.22)
Marshall Islands	35 to 39	0.17 (-0.10, 0.44)
Marshall Islands	40 to 44	0.22 (-0.09, 0.53)
Marshall Islands	45 to 49	0.26 (-0.19, 0.71)
Mauritania	15 to 19	-0.70 (-0.74, -0.66)
Mauritania	20 to 24	-0.22 (-0.26, -0.19)
Mauritania	25 to 29	-0.12 (-0.15, -0.09)
Mauritania	30 to 34	-0.05 (-0.08, -0.02)
Mauritania	35 to 39	0.00 (-0.03, 0.03)
Mauritania	40 to 44	0.04 (0.00, 0.08)

Mauritania	45 to 49	0.07 (0.02, 0.12)
Mauritius	15 to 19	-1.23 (-1.34, -1.11)
Mauritius	20 to 24	-0.44 (-0.52, -0.37)
Mauritius	25 to 29	0.14 (0.08, 0.20)
Mauritius	30 to 34	0.22 (0.16, 0.27)
Mauritius	35 to 39	0.29 (0.24, 0.34)
Mauritius	40 to 44	0.36 (0.30, 0.41)
Mauritius	45 to 49	0.40 (0.32, 0.48)
Mexico	15 to 19	-0.13 (-0.21, -0.06)
Mexico	20 to 24	0.11 (0.06, 0.16)
Mexico	25 to 29	0.34 (0.30, 0.38)
Mexico	30 to 34	0.39 (0.36, 0.43)
Mexico	35 to 39	0.41 (0.37, 0.45)
Mexico	40 to 44	0.43 (0.39, 0.47)
Mexico	45 to 49	0.43 (0.37, 0.49)
Micronesia (Federated States of)	15 to 19	-1.86 (-2.20, -1.51)
Micronesia (Federated States of)	20 to 24	-1.57 (-1.81, -1.32)
Micronesia (Federated States of)	25 to 29	-0.99 (-1.20, -0.79)
Micronesia (Federated States of)	30 to 34	-0.26 (-0.45, -0.07)
Micronesia (Federated States of)	35 to 39	0.25 (0.06, 0.44)
Micronesia (Federated States of)	40 to 44	0.30 (0.09, 0.51)
Micronesia (Federated States of)	45 to 49	0.32 (0.03, 0.62)
Monaco	15 to 19	-0.01 (-1.27, 1.27)
Monaco	20 to 24	0.21 (-0.64, 1.06)

Monaco	25 to 29	0.50 (-0.16, 1.16)
Monaco	30 to 34	0.75 (0.19, 1.32)
Monaco	35 to 39	0.79 (0.27, 1.31)
Monaco	40 to 44	0.83 (0.30, 1.36)
Monaco	45 to 49	0.83 (0.20, 1.47)
Mongolia	15 to 19	-1.22 (-1.27, -1.17)
Mongolia	20 to 24	-0.83 (-0.87, -0.80)
Mongolia	25 to 29	-0.39 (-0.42, -0.37)
Mongolia	30 to 34	-0.03 (-0.05, 0.00)
Mongolia	35 to 39	0.05 (0.02, 0.08)
Mongolia	40 to 44	0.05 (0.01, 0.09)
Mongolia	45 to 49	0.05 (-0.01, 0.10)
Montenegro	15 to 19	0.38 (0.17, 0.60)
Montenegro	20 to 24	0.52 (0.38, 0.66)
Montenegro	25 to 29	0.56 (0.45, 0.67)
Montenegro	30 to 34	0.57 (0.47, 0.66)
Montenegro	35 to 39	0.57 (0.48, 0.67)
Montenegro	40 to 44	0.58 (0.48, 0.67)
Montenegro	45 to 49	0.57 (0.44, 0.70)
Morocco	15 to 19	0.37 (0.34, 0.40)
Morocco	20 to 24	0.59 (0.57, 0.61)
Morocco	25 to 29	0.68 (0.67, 0.7)
Morocco	30 to 34	0.72 (0.70, 0.73)
Morocco	35 to 39	0.74 (0.73, 0.76)

Morocco	40 to 44	0.76 (0.74, 0.77)
Morocco	45 to 49	0.75 (0.73, 0.78)
Mozambique	15 to 19	-1.80 (-1.82, -1.77)
Mozambique	20 to 24	-0.35 (-0.37, -0.33)
Mozambique	25 to 29	-0.20 (-0.22, -0.18)
Mozambique	30 to 34	-0.15 (-0.17, -0.13)
Mozambique	35 to 39	-0.12 (-0.14, -0.09)
Mozambique	40 to 44	-0.09 (-0.12, -0.07)
Mozambique	45 to 49	-0.08 (-0.12, -0.04)
Myanmar	15 to 19	-0.59 (-0.65, -0.53)
Myanmar	20 to 24	-0.11 (-0.15, -0.07)
Myanmar	25 to 29	0.01 (-0.03, 0.04)
Myanmar	30 to 34	0.09 (0.06, 0.12)
Myanmar	35 to 39	0.17 (0.14, 0.20)
Myanmar	40 to 44	0.23 (0.20, 0.27)
Myanmar	45 to 49	0.29 (0.24, 0.34)
Namibia	15 to 19	-0.55 (-0.62, -0.48)
Namibia	20 to 24	-0.36 (-0.41, -0.31)
Namibia	25 to 29	-0.24 (-0.29, -0.20)
Namibia	30 to 34	-0.17 (-0.22, -0.13)
Namibia	35 to 39	-0.12 (-0.17, -0.07)
Namibia	40 to 44	-0.09 (-0.14, -0.03)
Namibia	45 to 49	-0.06 (-0.14, 0.02)
Nauru	15 to 19	-1.26 (-2.39, -0.11)

Nauru	20 to 24	-1.21 (-1.98, -0.44)
Nauru	25 to 29	-1.21 (-1.84, -0.57)
Nauru	30 to 34	-1.02 (-1.61, -0.42)
Nauru	35 to 39	-0.38 (-0.97, 0.21)
Nauru	40 to 44	0.19 (-0.46, 0.85)
Nauru	45 to 49	0.21 (-0.69, 1.11)
Nepal	15 to 19	-0.05 (-0.12, 0.01)
Nepal	20 to 24	0.16 (0.12, 0.20)
Nepal	25 to 29	0.14 (0.11, 0.18)
Nepal	30 to 34	0.13 (0.09, 0.17)
Nepal	35 to 39	0.12 (0.08, 0.16)
Nepal	40 to 44	0.12 (0.07, 0.16)
Nepal	45 to 49	0.13 (0.07, 0.19)
Netherlands	15 to 19	0.44 (0.36, 0.51)
Netherlands	20 to 24	0.56 (0.51, 0.61)
Netherlands	25 to 29	0.65 (0.61, 0.69)
Netherlands	30 to 34	0.70 (0.67, 0.73)
Netherlands	35 to 39	0.72 (0.69, 0.75)
Netherlands	40 to 44	0.72 (0.68, 0.75)
Netherlands	45 to 49	0.71 (0.67, 0.75)
New Zealand	15 to 19	-0.92 (-1.18, -0.67)
New Zealand	20 to 24	-0.74 (-0.91, -0.57)
New Zealand	25 to 29	-0.32 (-0.46, -0.19)
New Zealand	30 to 34	0.11 (-0.01, 0.23)

New Zealand	35 to 39	0.44 (0.32, 0.56)
New Zealand	40 to 44	0.65 (0.52, 0.78)
New Zealand	45 to 49	0.69 (0.51, 0.86)
Nicaragua	15 to 19	-0.21 (-0.31, -0.12)
Nicaragua	20 to 24	0.05 (-0.02, 0.11)
Nicaragua	25 to 29	0.24 (0.18, 0.29)
Nicaragua	30 to 34	0.28 (0.22, 0.33)
Nicaragua	35 to 39	0.31 (0.26, 0.37)
Nicaragua	40 to 44	0.35 (0.28, 0.42)
Nicaragua	45 to 49	0.37 (0.28, 0.47)
Niger	15 to 19	-0.39 (-0.41, -0.37)
Niger	20 to 24	-0.26 (-0.28, -0.24)
Niger	25 to 29	-0.18 (-0.19, -0.16)
Niger	30 to 34	-0.11 (-0.13, -0.09)
Niger	35 to 39	-0.07 (-0.09, -0.04)
Niger	40 to 44	-0.03 (-0.06, -0.01)
Niger	45 to 49	-0.01 (-0.05, 0.02)
Nigeria	15 to 19	-1.08 (-1.11, -1.05)
Nigeria	20 to 24	-0.55 (-0.57, -0.53)
Nigeria	25 to 29	-0.40 (-0.42, -0.38)
Nigeria	30 to 34	-0.29 (-0.31, -0.27)
Nigeria	35 to 39	-0.22 (-0.24, -0.20)
Nigeria	40 to 44	-0.18 (-0.21, -0.15)
Nigeria	45 to 49	-0.17 (-0.22, -0.13)

Niue	15 to 19	-1.86 (-5.16, 1.56)
Niue	20 to 24	-1.87 (-4.16, 0.48)
Niue	25 to 29	-1.15 (-2.93, 0.67)
Niue	30 to 34	-0.28 (-1.83, 1.29)
Niue	35 to 39	0.25 (-1.17, 1.69)
Niue	40 to 44	0.34 (-1.12, 1.82)
Niue	45 to 49	0.36 (-1.45, 2.21)
North Macedonia	15 to 19	0.26 (0.15, 0.37)
North Macedonia	20 to 24	0.57 (0.50, 0.64)
North Macedonia	25 to 29	0.64 (0.58, 0.69)
North Macedonia	30 to 34	0.66 (0.61, 0.71)
North Macedonia	35 to 39	0.68 (0.64, 0.72)
North Macedonia	40 to 44	0.69 (0.64, 0.73)
North Macedonia	45 to 49	0.67 (0.61, 0.74)
Northern Mariana Islands	15 to 19	-1.70 (-2.26, -1.13)
Northern Mariana Islands	20 to 24	-1.36 (-1.75, -0.97)
Northern Mariana Islands	25 to 29	-0.65 (-0.93, -0.36)
Northern Mariana Islands	30 to 34	-0.04 (-0.28, 0.20)
Northern Mariana Islands	35 to 39	0.10 (-0.13, 0.32)
Northern Mariana Islands	40 to 44	0.12 (-0.11, 0.36)
Northern Mariana Islands	45 to 49	0.13 (-0.22, 0.49)
Norway	15 to 19	0.22 (0.12, 0.32)
Norway	20 to 24	0.50 (0.43, 0.57)
Norway	25 to 29	0.54 (0.49, 0.60)

Norway	30 to 34	0.58 (0.53, 0.63)
Norway	35 to 39	0.60 (0.55, 0.65)
Norway	40 to 44	0.64 (0.59, 0.69)
Norway	45 to 49	0.68 (0.62, 0.75)
Oman	15 to 19	0.14 (-0.05, 0.34)
Oman	20 to 24	0.25 (0.11, 0.38)
Oman	25 to 29	0.43 (0.32, 0.55)
Oman	30 to 34	0.68 (0.58, 0.79)
Oman	35 to 39	0.86 (0.75, 0.98)
Oman	40 to 44	0.86 (0.72, 1.00)
Oman	45 to 49	0.85 (0.65, 1.05)
Pakistan	15 to 19	-0.42 (-0.47, -0.37)
Pakistan	20 to 24	0.13 (0.10, 0.17)
Pakistan	25 to 29	0.16 (0.13, 0.20)
Pakistan	30 to 34	0.16 (0.12, 0.19)
Pakistan	35 to 39	0.17 (0.13, 0.20)
Pakistan	40 to 44	0.19 (0.14, 0.23)
Pakistan	45 to 49	0.20 (0.14, 0.26)
Palau	15 to 19	-1.70 (-2.82, -0.57)
Palau	20 to 24	-1.42 (-2.15, -0.67)
Palau	25 to 29	-0.84 (-1.39, -0.29)
Palau	30 to 34	-0.18 (-0.63, 0.26)
Palau	35 to 39	0.31 (-0.09, 0.71)
Palau	40 to 44	0.33 (-0.10, 0.76)

Palau	45 to 49	0.33 (-0.25, 0.92)
Palestine	15 to 19	-0.17 (-0.25, -0.08)
Palestine	20 to 24	0.01 (-0.05, 0.07)
Palestine	25 to 29	0.25 (0.19, 0.30)
Palestine	30 to 34	0.46 (0.40, 0.51)
Palestine	35 to 39	0.48 (0.42, 0.54)
Palestine	40 to 44	0.49 (0.41, 0.56)
Palestine	45 to 49	0.48 (0.38, 0.58)
Panama	15 to 19	-0.06 (-0.13, 0.00)
Panama	20 to 24	0.30 (0.26, 0.35)
Panama	25 to 29	0.39 (0.35, 0.42)
Panama	30 to 34	0.41 (0.38, 0.45)
Panama	35 to 39	0.43 (0.39, 0.47)
Panama	40 to 44	0.44 (0.40, 0.48)
Panama	45 to 49	0.45 (0.39, 0.51)
Papua New Guinea	15 to 19	-2.23 (-2.37, -2.10)
Papua New Guinea	20 to 24	-1.20 (-1.30, -1.11)
Papua New Guinea	25 to 29	-0.51 (-0.59, -0.42)
Papua New Guinea	30 to 34	-0.11 (-0.19, -0.02)
Papua New Guinea	35 to 39	0.07 (-0.02, 0.16)
Papua New Guinea	40 to 44	0.13 (0.02, 0.24)
Papua New Guinea	45 to 49	0.19 (0.03, 0.35)
Paraguay	15 to 19	-0.28 (-0.34, -0.23)
Paraguay	20 to 24	0.24 (0.20, 0.27)

Paraguay	25 to 29	0.28 (0.25, 0.31)
Paraguay	30 to 34	0.30 (0.27, 0.33)
Paraguay	35 to 39	0.33 (0.30, 0.36)
Paraguay	40 to 44	0.35 (0.32, 0.39)
Paraguay	45 to 49	0.39 (0.35, 0.44)
Peru	15 to 19	-0.07 (-0.13, 0.00)
Peru	20 to 24	0.30 (0.25, 0.34)
Peru	25 to 29	0.35 (0.31, 0.38)
Peru	30 to 34	0.39 (0.35, 0.42)
Peru	35 to 39	0.43 (0.39, 0.46)
Peru	40 to 44	0.44 (0.40, 0.48)
Peru	45 to 49	0.45 (0.39, 0.50)
Philippines	15 to 19	-2.28 (-2.42, -2.15)
Philippines	20 to 24	-1.43 (-1.53, -1.34)
Philippines	25 to 29	-0.81 (-0.89, -0.73)
Philippines	30 to 34	-0.18 (-0.26, -0.10)
Philippines	35 to 39	-0.10 (-0.18, -0.02)
Philippines	40 to 44	-0.02 (-0.11, 0.08)
Philippines	45 to 49	0.04 (-0.09, 0.18)
Poland	15 to 19	-0.12 (-0.18, -0.07)
Poland	20 to 24	0.07 (0.03, 0.10)
Poland	25 to 29	0.25 (0.22, 0.28)
Poland	30 to 34	0.24 (0.22, 0.27)
Poland	35 to 39	0.23 (0.21, 0.25)

Poland	40 to 44	0.22 (0.20, 0.24)
Poland	45 to 49	0.23 (0.20, 0.26)
Portugal	15 to 19	0.18 (0.11, 0.25)
Portugal	20 to 24	0.55 (0.51, 0.60)
Portugal	25 to 29	0.74 (0.70, 0.78)
Portugal	30 to 34	0.80 (0.77, 0.84)
Portugal	35 to 39	0.82 (0.79, 0.85)
Portugal	40 to 44	0.86 (0.83, 0.90)
Portugal	45 to 49	0.93 (0.89, 0.97)
Puerto Rico	15 to 19	0.00 (-0.08, 0.08)
Puerto Rico	20 to 24	0.00 (-0.05, 0.05)
Puerto Rico	25 to 29	0.10 (0.07, 0.14)
Puerto Rico	30 to 34	0.22 (0.19, 0.25)
Puerto Rico	35 to 39	0.30 (0.27, 0.33)
Puerto Rico	40 to 44	0.32 (0.28, 0.35)
Puerto Rico	45 to 49	0.29 (0.25, 0.33)
Qatar	15 to 19	0.63 (0.52, 0.74)
Qatar	20 to 24	0.61 (0.54, 0.69)
Qatar	25 to 29	0.65 (0.59, 0.71)
Qatar	30 to 34	0.72 (0.67, 0.78)
Qatar	35 to 39	0.79 (0.74, 0.85)
Qatar	40 to 44	0.77 (0.70, 0.84)
Qatar	45 to 49	0.74 (0.63, 0.85)
Republic of Korea	15 to 19	-1.64 (-2.05, -1.24)

Republic of Korea	20 to 24	-1.73 (-1.98, -1.49)
Republic of Korea	25 to 29	-1.47 (-1.66, -1.27)
Republic of Korea	30 to 34	-0.88 (-1.05, -0.70)
Republic of Korea	35 to 39	-0.23 (-0.39, -0.06)
Republic of Korea	40 to 44	0.40 (0.22, 0.58)
Republic of Korea	45 to 49	0.39 (0.14, 0.64)
Republic of Moldova	15 to 19	-2.62 (-2.81, -2.43)
Republic of Moldova	20 to 24	-1.40 (-1.52, -1.29)
Republic of Moldova	25 to 29	-0.20 (-0.28, -0.12)
Republic of Moldova	30 to 34	0.09 (0.02, 0.16)
Republic of Moldova	35 to 39	0.10 (0.03, 0.17)
Republic of Moldova	40 to 44	0.10 (0.03, 0.18)
Republic of Moldova	45 to 49	0.09 (-0.02, 0.19)
Romania	15 to 19	-2.08 (-2.48, -1.68)
Romania	20 to 24	-1.29 (-1.55, -1.03)
Romania	25 to 29	-0.46 (-0.65, -0.27)
Romania	30 to 34	0.07 (-0.09, 0.24)
Romania	35 to 39	0.12 (-0.03, 0.27)
Romania	40 to 44	0.15 (0.00, 0.31)
Romania	45 to 49	0.19 (-0.02, 0.40)
Russian Federation	15 to 19	-0.74 (-0.83, -0.66)
Russian Federation	20 to 24	0.05 (0.00, 0.11)
Russian Federation	25 to 29	0.19 (0.15, 0.24)
Russian Federation	30 to 34	0.21 (0.18, 0.25)

Russian Federation	35 to 39	0.23 (0.20, 0.27)
Russian Federation	40 to 44	0.26 (0.23, 0.30)
Russian Federation	45 to 49	0.25 (0.21, 0.30)
Rwanda	15 to 19	-2.10 (-2.14, -2.06)
Rwanda	20 to 24	-0.40 (-0.43, -0.37)
Rwanda	25 to 29	-0.27 (-0.30, -0.24)
Rwanda	30 to 34	-0.22 (-0.25, -0.20)
Rwanda	35 to 39	-0.20 (-0.23, -0.17)
Rwanda	40 to 44	-0.19 (-0.22, -0.15)
Rwanda	45 to 49	-0.18 (-0.23, -0.12)
Saint Kitts and Nevis	15 to 19	-0.49 (-1.10, 0.13)
Saint Kitts and Nevis	20 to 24	-0.09 (-0.49, 0.30)
Saint Kitts and Nevis	25 to 29	0.20 (-0.11, 0.51)
Saint Kitts and Nevis	30 to 34	0.22 (-0.06, 0.50)
Saint Kitts and Nevis	35 to 39	0.22 (-0.05, 0.49)
Saint Kitts and Nevis	40 to 44	0.23 (-0.09, 0.54)
Saint Kitts and Nevis	45 to 49	0.23 (-0.24, 0.71)
Saint Lucia	15 to 19	-0.33 (-0.72, 0.05)
Saint Lucia	20 to 24	0.02 (-0.22, 0.26)
Saint Lucia	25 to 29	0.06 (-0.13, 0.25)
Saint Lucia	30 to 34	0.10 (-0.08, 0.27)
Saint Lucia	35 to 39	0.12 (-0.05, 0.30)
Saint Lucia	40 to 44	0.12 (-0.08, 0.32)
Saint Lucia	45 to 49	0.11 (-0.16, 0.39)

Saint Vincent and the Grenadines	15 to 19	-0.14 (-0.58, 0.29)
Saint Vincent and the Grenadines	20 to 24	0.20 (-0.08, 0.47)
Saint Vincent and the Grenadines	25 to 29	0.25 (0.02, 0.47)
Saint Vincent and the Grenadines	30 to 34	0.27 (0.06, 0.47)
Saint Vincent and the Grenadines	35 to 39	0.27 (0.07, 0.47)
Saint Vincent and the Grenadines	40 to 44	0.27 (0.04, 0.50)
Saint Vincent and the Grenadines	45 to 49	0.27 (-0.05, 0.59)
Samoa	15 to 19	-0.88 (-1.13, -0.64)
Samoa	20 to 24	-0.81 (-0.99, -0.63)
Samoa	25 to 29	-0.47 (-0.62, -0.32)
Samoa	30 to 34	-0.09 (-0.24, 0.05)
Samoa	35 to 39	0.00 (-0.15, 0.15)
Samoa	40 to 44	0.04 (-0.13, 0.21)
Samoa	45 to 49	0.08 (-0.15, 0.31)
San Marino	15 to 19	-0.10 (-1.31, 1.13)
San Marino	20 to 24	0.13 (-0.68, 0.94)
San Marino	25 to 29	0.48 (-0.15, 1.12)
San Marino	30 to 34	0.70 (0.15, 1.26)
San Marino	35 to 39	0.72 (0.19, 1.26)
San Marino	40 to 44	0.74 (0.17, 1.30)
San Marino	45 to 49	0.73 (-0.01, 1.47)
Sao Tome and Principe	15 to 19	-1.60 (-1.82, -1.38)
Sao Tome and Principe	20 to 24	-0.29 (-0.45, -0.14)
Sao Tome and Principe	25 to 29	-0.19 (-0.33, -0.05)

Sao Tome and Principe	30 to 34	-0.14 (-0.28, 0.00)
Sao Tome and Principe	35 to 39	-0.12 (-0.27, 0.04)
Sao Tome and Principe	40 to 44	-0.11 (-0.30, 0.08)
Sao Tome and Principe	45 to 49	-0.10 (-0.38, 0.18)
Saudi Arabia	15 to 19	-0.25 (-0.48, -0.02)
Saudi Arabia	20 to 24	-0.06 (-0.21, 0.09)
Saudi Arabia	25 to 29	0.20 (0.08, 0.33)
Saudi Arabia	30 to 34	0.52 (0.40, 0.64)
Saudi Arabia	35 to 39	0.75 (0.62, 0.88)
Saudi Arabia	40 to 44	0.78 (0.63, 0.94)
Saudi Arabia	45 to 49	0.79 (0.56, 1.02)
Senegal	15 to 19	-1.22 (-1.25, -1.19)
Senegal	20 to 24	-0.04 (-0.06, -0.02)
Senegal	25 to 29	0.04 (0.02, 0.06)
Senegal	30 to 34	0.07 (0.05, 0.09)
Senegal	35 to 39	0.10 (0.08, 0.12)
Senegal	40 to 44	0.12 (0.10, 0.15)
Senegal	45 to 49	0.15 (0.11, 0.18)
Serbia	15 to 19	0.31 (0.25, 0.37)
Serbia	20 to 24	0.54 (0.50, 0.58)
Serbia	25 to 29	0.57 (0.55, 0.60)
Serbia	30 to 34	0.59 (0.57, 0.62)
Serbia	35 to 39	0.60 (0.58, 0.63)
Serbia	40 to 44	0.60 (0.57, 0.62)

Serbia	45 to 49	0.59 (0.56, 0.62)
Seychelles	15 to 19	-1.51 (-1.96, -1.06)
Seychelles	20 to 24	-0.92 (-1.20, -0.63)
Seychelles	25 to 29	-0.29 (-0.50, -0.07)
Seychelles	30 to 34	0.13 (-0.06, 0.32)
Seychelles	35 to 39	0.18 (-0.01, 0.37)
Seychelles	40 to 44	0.22 (0.00, 0.45)
Seychelles	45 to 49	0.26 (-0.06, 0.57)
Sierra Leone	15 to 19	-0.43 (-0.46, -0.40)
Sierra Leone	20 to 24	-0.29 (-0.31, -0.26)
Sierra Leone	25 to 29	-0.20 (-0.22, -0.18)
Sierra Leone	30 to 34	-0.14 (-0.17, -0.12)
Sierra Leone	35 to 39	-0.10 (-0.13, -0.07)
Sierra Leone	40 to 44	-0.07 (-0.10, -0.03)
Sierra Leone	45 to 49	-0.04 (-0.08, 0.01)
Singapore	15 to 19	-1.35 (-1.61, -1.08)
Singapore	20 to 24	-1.13 (-1.28, -0.97)
Singapore	25 to 29	-0.76 (-0.87, -0.65)
Singapore	30 to 34	-0.30 (-0.40, -0.20)
Singapore	35 to 39	0.06 (-0.04, 0.16)
Singapore	40 to 44	0.09 (-0.03, 0.20)
Singapore	45 to 49	0.11 (-0.05, 0.27)
Slovakia	15 to 19	-0.56 (-0.67, -0.44)
Slovakia	20 to 24	-0.05 (-0.13, 0.02)

Slovakia	25 to 29	0.41 (0.36, 0.46)
Slovakia	30 to 34	0.42 (0.37, 0.47)
Slovakia	35 to 39	0.40 (0.36, 0.45)
Slovakia	40 to 44	0.39 (0.35, 0.44)
Slovakia	45 to 49	0.38 (0.32, 0.44)
Slovenia	15 to 19	-0.67 (-0.83, -0.52)
Slovenia	20 to 24	-0.06 (-0.16, 0.03)
Slovenia	25 to 29	0.41 (0.34, 0.47)
Slovenia	30 to 34	0.45 (0.39, 0.50)
Slovenia	35 to 39	0.47 (0.42, 0.52)
Slovenia	40 to 44	0.50 (0.44, 0.55)
Slovenia	45 to 49	0.51 (0.44, 0.59)
Solomon Islands	15 to 19	-2.59 (-2.91, -2.26)
Solomon Islands	20 to 24	-2.13 (-2.36, -1.89)
Solomon Islands	25 to 29	-1.33 (-1.53, -1.13)
Solomon Islands	30 to 34	-0.40 (-0.59, -0.20)
Solomon Islands	35 to 39	0.14 (-0.07, 0.35)
Solomon Islands	40 to 44	0.20 (-0.06, 0.45)
Solomon Islands	45 to 49	0.23 (-0.12, 0.59)
Somalia	15 to 19	-0.14 (-0.16, -0.11)
Somalia	20 to 24	-0.09 (-0.10, -0.07)
Somalia	25 to 29	-0.05 (-0.07, -0.03)
Somalia	30 to 34	-0.02 (-0.04, 0.00)
Somalia	35 to 39	0.00 (-0.01, 0.02)

Somalia	40 to 44	0.02 (0.00, 0.04)
Somalia	45 to 49	0.03 (0.00, 0.06)
South Africa	15 to 19	-0.79 (-0.95, -0.62)
South Africa	20 to 24	-0.42 (-0.53, -0.31)
South Africa	25 to 29	-0.08 (-0.17, 0.01)
South Africa	30 to 34	0.14 (0.06, 0.22)
South Africa	35 to 39	0.18 (0.10, 0.26)
South Africa	40 to 44	0.19 (0.09, 0.28)
South Africa	45 to 49	0.19 (0.06, 0.33)
South Sudan	15 to 19	-0.06 (-0.10, -0.02)
South Sudan	20 to 24	0.03 (-0.01, 0.06)
South Sudan	25 to 29	0.09 (0.06, 0.12)
South Sudan	30 to 34	0.14 (0.12, 0.17)
South Sudan	35 to 39	0.18 (0.15, 0.21)
South Sudan	40 to 44	0.20 (0.16, 0.24)
South Sudan	45 to 49	0.21 (0.15, 0.26)
Spain	15 to 19	-0.37 (-0.51, -0.22)
Spain	20 to 24	0.01 (-0.08, 0.11)
Spain	25 to 29	0.39 (0.32, 0.46)
Spain	30 to 34	0.64 (0.58, 0.70)
Spain	35 to 39	0.65 (0.59, 0.71)
Spain	40 to 44	0.61 (0.55, 0.68)
Spain	45 to 49	0.63 (0.55, 0.71)
Sri Lanka	15 to 19	-0.67 (-0.74, -0.60)

Sri Lanka	20 to 24	0.03 (-0.01, 0.07)
Sri Lanka	25 to 29	0.13 (0.09, 0.16)
Sri Lanka	30 to 34	0.19 (0.16, 0.22)
Sri Lanka	35 to 39	0.23 (0.20, 0.26)
Sri Lanka	40 to 44	0.23 (0.20, 0.27)
Sri Lanka	45 to 49	0.21 (0.16, 0.25)
Sudan	15 to 19	-0.53 (-0.55, -0.51)
Sudan	20 to 24	-0.01 (-0.02, 0.01)
Sudan	25 to 29	0.15 (0.13, 0.16)
Sudan	30 to 34	0.24 (0.23, 0.25)
Sudan	35 to 39	0.30 (0.28, 0.31)
Sudan	40 to 44	0.34 (0.32, 0.36)
Sudan	45 to 49	0.39 (0.36, 0.42)
Suriname	15 to 19	-0.11 (-0.31, 0.08)
Suriname	20 to 24	0.32 (0.19, 0.45)
Suriname	25 to 29	0.37 (0.27, 0.48)
Suriname	30 to 34	0.39 (0.30, 0.49)
Suriname	35 to 39	0.40 (0.31, 0.50)
Suriname	40 to 44	0.41 (0.30, 0.52)
Suriname	45 to 49	0.41 (0.27, 0.56)
Sweden	15 to 19	0.66 (0.59, 0.73)
Sweden	20 to 24	0.59 (0.54, 0.64)
Sweden	25 to 29	0.64 (0.60, 0.67)
Sweden	30 to 34	0.66 (0.62, 0.69)

Sweden	35 to 39	0.67 (0.64, 0.71)
Sweden	40 to 44	0.69 (0.65, 0.73)
Sweden	45 to 49	0.69 (0.65, 0.74)
Switzerland	15 to 19	0.55 (0.47, 0.62)
Switzerland	20 to 24	0.62 (0.57, 0.67)
Switzerland	25 to 29	0.65 (0.61, 0.69)
Switzerland	30 to 34	0.66 (0.63, 0.70)
Switzerland	35 to 39	0.68 (0.64, 0.71)
Switzerland	40 to 44	0.69 (0.66, 0.73)
Switzerland	45 to 49	0.71 (0.66, 0.76)
Syrian Arab Republic	15 to 19	-0.17 (-0.26, -0.08)
Syrian Arab Republic	20 to 24	0.08 (0.02, 0.15)
Syrian Arab Republic	25 to 29	0.35 (0.29, 0.40)
Syrian Arab Republic	30 to 34	0.56 (0.51, 0.61)
Syrian Arab Republic	35 to 39	0.59 (0.54, 0.64)
Syrian Arab Republic	40 to 44	0.60 (0.54, 0.66)
Syrian Arab Republic	45 to 49	0.61 (0.52, 0.69)
Taiwan (Province of China)	15 to 19	-2.91 (-3.43, -2.38)
Taiwan (Province of China)	20 to 24	-2.46 (-2.77, -2.14)
Taiwan (Province of China)	25 to 29	-1.71 (-1.96, -1.47)
Taiwan (Province of China)	30 to 34	-0.77 (-0.97, -0.57)
Taiwan (Province of China)	35 to 39	0.09 (-0.09, 0.27)
Taiwan (Province of China)	40 to 44	0.24 (0.04, 0.43)
Taiwan (Province of China)	45 to 49	0.33 (0.04, 0.61)

Tajikistan	15 to 19	-0.45 (-0.48, -0.41)
Tajikistan	20 to 24	0.03 (0.00, 0.05)
Tajikistan	25 to 29	0.11 (0.08, 0.13)
Tajikistan	30 to 34	0.15 (0.13, 0.18)
Tajikistan	35 to 39	0.18 (0.15, 0.20)
Tajikistan	40 to 44	0.18 (0.15, 0.21)
Tajikistan	45 to 49	0.17 (0.12, 0.22)
Thailand	15 to 19	-2.22 (-2.60, -1.83)
Thailand	20 to 24	-1.43 (-1.67, -1.19)
Thailand	25 to 29	-0.55 (-0.73, -0.37)
Thailand	30 to 34	0.11 (-0.04, 0.27)
Thailand	35 to 39	0.21 (0.06, 0.35)
Thailand	40 to 44	0.28 (0.12, 0.44)
Thailand	45 to 49	0.36 (0.15, 0.58)
Timor-Leste	15 to 19	-0.41 (-0.50, -0.32)
Timor-Leste	20 to 24	-0.30 (-0.37, -0.24)
Timor-Leste	25 to 29	-0.21 (-0.27, -0.15)
Timor-Leste	30 to 34	-0.14 (-0.20, -0.08)
Timor-Leste	35 to 39	-0.06 (-0.12, 0.00)
Timor-Leste	40 to 44	0.01 (-0.06, 0.08)
Timor-Leste	45 to 49	0.05 (-0.05, 0.15)
Togo	15 to 19	-0.28 (-0.32, -0.25)
Togo	20 to 24	-0.14 (-0.17, -0.12)
Togo	25 to 29	-0.05 (-0.07, -0.03)

Togo	30 to 34	0.00 (-0.02, 0.02)
Togo	35 to 39	0.05 (0.02, 0.07)
Togo	40 to 44	0.09 (0.06, 0.12)
Togo	45 to 49	0.11 (0.07, 0.16)
Tokelau	15 to 19	-1.92 (-5.30, 1.57)
Tokelau	20 to 24	-1.40 (-3.83, 1.08)
Tokelau	25 to 29	-0.25 (-2.05, 1.58)
Tokelau	30 to 34	0.23 (-1.48, 1.98)
Tokelau	35 to 39	0.36 (-1.30, 2.05)
Tokelau	40 to 44	0.30 (-1.43, 2.05)
Tokelau	45 to 49	0.37 (-1.97, 2.76)
Tonga	15 to 19	-2.28 (-2.69, -1.87)
Tonga	20 to 24	-1.98 (-2.27, -1.70)
Tonga	25 to 29	-1.41 (-1.65, -1.18)
Tonga	30 to 34	-0.67 (-0.88, -0.45)
Tonga	35 to 39	0.08 (-0.14, 0.29)
Tonga	40 to 44	0.14 (-0.10, 0.38)
Tonga	45 to 49	0.16 (-0.15, 0.48)
Trinidad and Tobago	15 to 19	-0.03 (-0.16, 0.11)
Trinidad and Tobago	20 to 24	0.34 (0.25, 0.42)
Trinidad and Tobago	25 to 29	0.37 (0.31, 0.44)
Trinidad and Tobago	30 to 34	0.38 (0.32, 0.43)
Trinidad and Tobago	35 to 39	0.37 (0.32, 0.43)
Trinidad and Tobago	40 to 44	0.36 (0.30, 0.43)

Trinidad and Tobago	45 to 49	0.33 (0.24, 0.41)
Tunisia	15 to 19	-0.95 (-1.21, -0.70)
Tunisia	20 to 24	-0.42 (-0.59, -0.26)
Tunisia	25 to 29	0.15 (0.02, 0.28)
Tunisia	30 to 34	0.49 (0.38, 0.61)
Tunisia	35 to 39	0.55 (0.43, 0.67)
Tunisia	40 to 44	0.60 (0.46, 0.73)
Tunisia	45 to 49	0.63 (0.44, 0.83)
Turkey	15 to 19	-0.29 (-0.38, -0.20)
Turkey	20 to 24	0.14 (0.08, 0.20)
Turkey	25 to 29	0.40 (0.36, 0.45)
Turkey	30 to 34	0.47 (0.42, 0.51)
Turkey	35 to 39	0.52 (0.47, 0.56)
Turkey	40 to 44	0.55 (0.50, 0.60)
Turkey	45 to 49	0.57 (0.51, 0.64)
Turkmenistan	15 to 19	-0.26 (-0.31, -0.22)
Turkmenistan	20 to 24	0.26 (0.23, 0.29)
Turkmenistan	25 to 29	0.29 (0.26, 0.32)
Turkmenistan	30 to 34	0.29 (0.26, 0.31)
Turkmenistan	35 to 39	0.29 (0.26, 0.32)
Turkmenistan	40 to 44	0.29 (0.26, 0.32)
Turkmenistan	45 to 49	0.27 (0.23, 0.32)
Tuvalu	15 to 19	-1.47 (-2.52, -0.40)
Tuvalu	20 to 24	-0.84 (-1.58, -0.09)

Tuvalu	25 to 29	-0.23 (-0.85, 0.39)
Tuvalu	30 to 34	0.15 (-0.42, 0.73)
Tuvalu	35 to 39	0.23 (-0.34, 0.80)
Tuvalu	40 to 44	0.28 (-0.34, 0.89)
Tuvalu	45 to 49	0.30 (-0.50, 1.11)
Uganda	15 to 19	-1.59 (-1.65, -1.54)
Uganda	20 to 24	-0.56 (-0.60, -0.52)
Uganda	25 to 29	-0.41 (-0.44, -0.37)
Uganda	30 to 34	-0.30 (-0.33, -0.26)
Uganda	35 to 39	-0.22 (-0.27, -0.18)
Uganda	40 to 44	-0.17 (-0.22, -0.11)
Uganda	45 to 49	-0.13 (-0.20, -0.05)
Ukraine	15 to 19	0.00 (-0.06, 0.06)
Ukraine	20 to 24	0.49 (0.46, 0.53)
Ukraine	25 to 29	0.53 (0.50, 0.56)
Ukraine	30 to 34	0.52 (0.49, 0.54)
Ukraine	35 to 39	0.50 (0.48, 0.53)
Ukraine	40 to 44	0.49 (0.46, 0.51)
Ukraine	45 to 49	0.44 (0.41, 0.47)
United Arab Emirates	15 to 19	0.90 (0.84, 0.95)
United Arab Emirates	20 to 24	0.87 (0.83, 0.91)
United Arab Emirates	25 to 29	0.88 (0.85, 0.92)
United Arab Emirates	30 to 34	0.93 (0.90, 0.96)
United Arab Emirates	35 to 39	0.96 (0.93, 0.99)

United Arab Emirates	40 to 44	0.94 (0.90, 0.98)
United Arab Emirates	45 to 49	0.92 (0.85, 0.98)
United Kingdom	15 to 19	0.56 (0.44, 0.68)
United Kingdom	20 to 24	0.64 (0.56, 0.71)
United Kingdom	25 to 29	0.75 (0.69, 0.81)
United Kingdom	30 to 34	0.78 (0.72, 0.83)
United Kingdom	35 to 39	0.76 (0.70, 0.81)
United Kingdom	40 to 44	0.76 (0.70, 0.81)
United Kingdom	45 to 49	0.78 (0.71, 0.86)
United Republic of Tanzania	15 to 19	-1.54 (-1.58, -1.5)
United Republic of Tanzania	20 to 24	-0.21 (-0.24, -0.18)
United Republic of Tanzania	25 to 29	-0.08 (-0.11, -0.05)
United Republic of Tanzania	30 to 34	-0.01 (-0.04, 0.02)
United Republic of Tanzania	35 to 39	0.04 (0.01, 0.07)
United Republic of Tanzania	40 to 44	0.08 (0.04, 0.12)
United Republic of Tanzania	45 to 49	0.12 (0.07, 0.18)
United States of America	15 to 19	0.60 (0.48, 0.72)
United States of America	20 to 24	0.50 (0.42, 0.59)
United States of America	25 to 29	0.58 (0.52, 0.65)
United States of America	30 to 34	0.70 (0.64, 0.76)
United States of America	35 to 39	0.78 (0.72, 0.83)
United States of America	40 to 44	0.83 (0.77, 0.90)
United States of America	45 to 49	0.83 (0.75, 0.92)
United States Virgin Islands	15 to 19	0.08 (-0.47, 0.63)

United States Virgin Islands	20 to 24	0.13 (-0.23, 0.49)
United States Virgin Islands	25 to 29	0.21 (-0.06, 0.48)
United States Virgin Islands	30 to 34	0.28 (0.04, 0.51)
United States Virgin Islands	35 to 39	0.36 (0.15, 0.57)
United States Virgin Islands	40 to 44	0.42 (0.20, 0.63)
United States Virgin Islands	45 to 49	0.39 (0.13, 0.66)
Uruguay	15 to 19	0.14 (0.02, 0.26)
Uruguay	20 to 24	0.31 (0.24, 0.39)
Uruguay	25 to 29	0.48 (0.42, 0.53)
Uruguay	30 to 34	0.51 (0.46, 0.57)
Uruguay	35 to 39	0.59 (0.54, 0.64)
Uruguay	40 to 44	0.66 (0.61, 0.71)
Uruguay	45 to 49	0.77 (0.69, 0.84)
Uzbekistan	15 to 19	-0.82 (-0.86, -0.78)
Uzbekistan	20 to 24	0.09 (0.06, 0.11)
Uzbekistan	25 to 29	0.18 (0.15, 0.20)
Uzbekistan	30 to 34	0.20 (0.18, 0.22)
Uzbekistan	35 to 39	0.21 (0.18, 0.23)
Uzbekistan	40 to 44	0.21 (0.18, 0.24)
Uzbekistan	45 to 49	0.21 (0.16, 0.26)
Vanuatu	15 to 19	-1.45 (-1.67, -1.22)
Vanuatu	20 to 24	-1.01 (-1.17, -0.85)
Vanuatu	25 to 29	-0.54 (-0.67, -0.40)
Vanuatu	30 to 34	0.01 (-0.12, 0.14)

Vanuatu	35 to 39	0.27 (0.13, 0.41)
Vanuatu	40 to 44	0.31 (0.15, 0.48)
Vanuatu	45 to 49	0.34 (0.11, 0.58)
Venezuela (Bolivarian Republic of)	15 to 19	-0.08 (-0.14, -0.01)
Venezuela (Bolivarian Republic of)	20 to 24	0.23 (0.19, 0.27)
Venezuela (Bolivarian Republic of)	25 to 29	0.33 (0.30, 0.37)
Venezuela (Bolivarian Republic of)	30 to 34	0.38 (0.35, 0.41)
Venezuela (Bolivarian Republic of)	35 to 39	0.40 (0.37, 0.43)
Venezuela (Bolivarian Republic of)	40 to 44	0.40 (0.37, 0.44)
Venezuela (Bolivarian Republic of)	45 to 49	0.40 (0.35, 0.45)
Viet Nam	15 to 19	-2.05 (-2.14, -1.96)
Viet Nam	20 to 24	-0.77 (-0.82, -0.71)
Viet Nam	25 to 29	-0.41 (-0.45, -0.37)
Viet Nam	30 to 34	-0.28 (-0.33, -0.24)
Viet Nam	35 to 39	-0.18 (-0.22, -0.13)
Viet Nam	40 to 44	-0.08 (-0.13, -0.03)
Viet Nam	45 to 49	0.02 (-0.06, 0.09)
Yemen	15 to 19	-1.02 (-1.09, -0.95)
Yemen	20 to 24	-0.28 (-0.34, -0.23)
Yemen	25 to 29	-0.05 (-0.09, 0.00)
Yemen	30 to 34	0.07 (0.02, 0.12)
Yemen	35 to 39	0.16 (0.11, 0.21)
Yemen	40 to 44	0.23 (0.17, 0.29)
Yemen	45 to 49	0.30 (0.21, 0.39)

Zambia	15 to 19	-0.88 (-0.91, -0.84)
Zambia	20 to 24	-0.37 (-0.40, -0.34)
Zambia	25 to 29	-0.22 (-0.24, -0.19)
Zambia	30 to 34	-0.13 (-0.16, -0.10)
Zambia	35 to 39	-0.08 (-0.11, -0.05)
Zambia	40 to 44	-0.07 (-0.10, -0.03)
Zambia	45 to 49	-0.06 (-0.11, -0.01)
Zimbabwe	15 to 19	-2.09 (-2.22, -1.97)
Zimbabwe	20 to 24	-0.75 (-0.84, -0.66)
Zimbabwe	25 to 29	0.01 (-0.07, 0.09)
Zimbabwe	30 to 34	0.05 (-0.03, 0.14)
Zimbabwe	35 to 39	0.07 (-0.03, 0.16)
Zimbabwe	40 to 44	0.08 (-0.03, 0.19)
Zimbabwe	45 to 49	0.08 (-0.08, 0.24)

CLD, chronic liver disease; WCBA, women of childbearing age.

Table S4 The number and proportion of different CLDs among WCBA in different SDI regions from 1992 to 2021, and the AS prevalence rates of each CLD.

	Global		High SDI		High-middle SDI		Middle SDI		Low-middle SDI		Low SDI	
	1992	2021	1992	2021	1992	2021	1992	2021	1992	2021	1992	2021
**Prevalence number in millions (95% UI)**												
NAFLD	164.77 (142.20, 187.12)	307.11 (273.95, 347.80)	17.12 (15.15, 19.38)	26.60 (23.72, 30.14)	34.28 (30.41, 39.14)	52.46 (46.61, 59.79)	62.45 (55.43, 70.97)	113.09 (100.74, 128.55)	37.31 (33.07, 42.29)	79.45 (70.77, 89.52)	13.45 (11.88, 15.21)	35.27 (31.21, 39.92)
HBV	73.73 (66.55, 82.32)	71.40 (64.42, 79.19)	4.11 (3.77, 4.49)	2.69 (2.46, 2.97)	15.91 (14.31, 17.68)	10.28 (9.07, 11.62)	31.08 (27.93, 34.83)	23.43 (20.94, 26.16)	12.96 (11.59, 14.49)	17.19 (15.52, 19.11)	9.63 (8.69, 10.73)	17.76 (16.17, 19.65)
HCV	30.19 (24.12, 36.53)	34.00 (26.65, 41.85)	2.30 (1.82, 2.78)	2.23 (1.74, 2.73)	5.96 (4.73, 7.28)	4.54 (3.57, 5.59)	11.01 (8.78, 13.40)	10.77 (8.50, 13.25)	7.51 (6.03, 9.06)	10.10 (7.88, 12.46)	3.38 (2.66, 4.15)	6.35 (4.94, 7.84)
ALD	0.15 (0.11, 0.20)	0.21 (0.16, 0.29)	0.044 (0.032, 0.057)	0.038 (0.028, 0.051)	0.032 (0.023, 0.041)	0.055 (0.040, 0.074)	0.035 (0.026, 0.046)	0.047 (0.035, 0.063)	0.031 (0.023, 0.042)	0.054 (0.040, 0.073)	0.010 (0.007, 0.014)	0.020 (0.014, 0.027)
Other	0.79 (0.63, 0.97)	0.94 (0.76, 1.15)	0.065 (0.048, 0.084)	0.063 (0.048, 0.082)	0.085 (0.066, 0.106)	0.092 (0.072, 0.118)	0.23 (0.19, 0.29)	0.21 (0.17, 0.26)	0.30 (0.24, 0.36)	0.38 (0.31, 0.47)	0.11 (0.090, 0.14)	0.19 (0.16, 0.24)
Total CLD	269.63 (248.01, 296.81)	413.67 (377.66, 459.28)	23.63 (21.56, 26.18)	31.62 (28.60, 35.32)	56.27 (51.83, 62.03)	67.42 (61.22, 75.25)	104.81 (96.56, 115.35)	147.55 (134.76, 164.38)	58.11 (53.32, 63.97)	107.17 (97.56, 118.74)	26.57 (24.52, 28.92)	59.59 (54.65, 65.15)
**Proportion of CLD by different causes (%)**												
NAFLD	61.11	74.24	72.44	84.12	60.91	77.80	59.58	76.65	64.21	74.13	50.60	59.19

HBV	27.34	17.26	17.38	8.52	28.28	15.25	29.65	15.88	22.65	16.04	36.23	29.81
HCV	11.20	8.22	9.72	7.04	10.60	6.73	10.51	7.30	14.17	9.42	12.72	10.65
ALD	0.057	0.052	0.19	0.12	0.056	0.081	0.034	0.032	0.065	0.051	0.039	0.033
Other	0.29	0.23	0.27	0.20	0.15	0.14	0.22	0.14	0.56	0.36	0.42	0.33
**Age-standardised prevalence rate (per 100,000, 95% UI)**												
NAFLD	12429.34 (9581.26, 15758.12)	15446.14 (11952.90, 19522.04)	7215.03 (5517.46, 9277.45)	10245.11 (7892.90, 13045.53)	12177.26 (9335.15, 15509.21)	15574.98 (11996.44, 19733.20)	14500.49 (11163.80, 18405.19)	17459.05 (13505.55, 22131.56)	14257.69 (11001.28, 18080.30)	16151.31 (12498.80, 20452.12)	12737.08 (9816.23, 16187.70)	14205.52 (10926.74, 18068.06)
HBV	5294.79 (4746.61, 5930.48)	3636.02 (3253.44, 4069.24)	1786.93 (1623.56, 1973.51)	1014.44 (915.02, 1127.63)	5553.21 (4972.25, 6223.26)	3046.82 (2668.96, 3462.40)	6685.57 (5981.47, 7519.78)	3676.95 (3253.74, 4144.51)	4513.81 (4027.42, 5070.42)	3419.88 (3071.76, 3820.84)	8096.09 (7254.09, 9011.86)	6531.66 (5907.33, 7244.39)
HCV	2207 (1750.97, 2711.38)	1735.27 (1344.93, 2164.57)	980.01 (766.22, 1223.28)	872.22 (667.59, 1103.39)	2096.55 (1657.42, 2582.13)	1440.63 (1118.18, 1791.31)	2414.98 (1914.69, 2964.26)	1717.08 (1334.94, 2133.95)	2713.07 (2165.12, 3317.52)	2014.88 (1552.30, 2516.17)	2991.35 (2331.50, 3728.62)	2410.59 (1864.62, 3015.04)
ALD	12.41 (8.22, 17.96)	10.51 (6.96, 15.47)	17.64 (11.42, 25.10)	13.05 (8.40, 19.07)	11.94 (7.93, 17.02)	14.35 (9.48, 20.77)	8.99 (5.97, 13.23)	6.97 (4.53, 10.35)	13.05 (8.47, 19.53)	11.42 (7.47, 17.01)	11.05 (6.76, 17.09)	8.81 (5.37, 13.60)
Other	53.84 (38.71, 71.73)	49.90 (35.77, 66.30)	29.07 (18.36, 42.89)	27.59 (17.84, 39.70)	29.68 (20.12, 41.59)	32.27 (21.83, 45.42)	45.86 (32.72, 61.53)	36.64 (26.17, 49.09)	90.11 (66.43, 117.29)	72.09 (52.24, 94.70)	80.62 (58.12, 106.49)	60.88 (43.66, 80.77)
Total CLD	19997.38 (16964.05, 23501.98)	20877.84 (17250.33, 25044.54)	10028.67 (8281.08, 12137.16)	12172.40 (9786.56, 14962.89)	19868.63 (16875.18, 23384.03)	20109.07 (16441.92, 24339.41)	23655.89 (20119.54, 27770.50)	22896.70 (18828.96, 27605.91)	21587.73 (18228.68, 25551.42)	21669.57 (17910.79, 26002.33)	23916.18 (20679.64, 27711.04)	23217.47 (19732.88, 27227.29)

NAFLD, HBV, HCV, ALD, and Other are different causes of CLD.

ALD, alcoholic liver disease; AS, age-standardized; CLD, chronic liver disease; HBV, hepatitis B virus; HCV, hepatitis C virus; NAFLD, non-alcoholic fatty liver disease; SDI, sociodemographic index; WCBA, women of childbearing age.

Table S5 Age effects on CLD prevalence in WCBA across SDI quintiles.

Location	Age	Prevalence rate (per 100,000 population)
Global	15 to 19	10919.15 (10801.12, 11038.46)
Global	20 to 24	15523.80 (15378.02, 15670.96)
Global	25 to 29	20372.19 (20194.54, 20551.41)
Global	30 to 34	23206.69 (23005.54, 23409.59)
Global	35 to 39	24886.30 (24633.59, 25141.61)
Global	40 to 44	27444.71 (27154.76, 27737.75)
Global	45 to 49	30292.53 (29955.73, 30633.12)
High SDI	15 to 19	5551.82 (5451.69, 5653.79)
High SDI	20 to 24	8545.61 (8418.23, 8674.93)
High SDI	25 to 29	12068.96 (11907.71, 12232.39)
High SDI	30 to 34	14046.08 (13861.83, 14232.78)
High SDI	35 to 39	15040.06 (14811.28, 15272.38)
High SDI	40 to 44	16795.71 (16533.03, 17062.57)
High SDI	45 to 49	19340.83 (19028.36, 19658.43)
High-middle SDI	15 to 19	10239.28 (9939.28, 10548.34)
High-middle SDI	20 to 24	14737.62 (14369.06, 15115.64)
High-middle SDI	25 to 29	19672.87 (19224.46, 20131.74)
High-middle SDI	30 to 34	22477.39 (21973.06, 22993.29)
High-middle SDI	35 to 39	24106.05 (23482.27, 24746.39)
High-middle SDI	40 to 44	26574.96 (25868.82, 27300.36)
High-middle SDI	45 to 49	29604.03 (28789.20, 30441.93)
Middle SDI	15 to 19	11489.22 (11268.99, 11713.75)

Middle SDI	20 to 24	16727.14 (16449.75, 17009.21)
Middle SDI	25 to 29	22210.09 (21870.50, 22554.96)
Middle SDI	30 to 34	25437.57 (25053.21, 25827.83)
Middle SDI	35 to 39	27379.30 (26897.49, 27869.75)
Middle SDI	40 to 44	30285.46 (29733.46, 30847.70)
Middle SDI	45 to 49	33460.05 (32821.05, 34111.49)
Low-middle SDI	15 to 19	10980.20 (10928.96, 11031.68)
Low-middle SDI	20 to 24	16050.84 (15985.72, 16116.23)
Low-middle SDI	25 to 29	21150.74 (21070.19, 21231.61)
Low-middle SDI	30 to 34	24281.84 (24189.13, 24374.90)
Low-middle SDI	35 to 39	26383.59 (26264.08, 26503.64)
Low-middle SDI	40 to 44	29475.16 (29334.68, 29616.30)
Low-middle SDI	45 to 49	32499.66 (32333.94, 32666.22)
Low SDI	15 to 19	14604.11 (14547.41, 14661.04)
Low SDI	20 to 24	18850.10 (18784.10, 18916.32)
Low SDI	25 to 29	23091.44 (23013.58, 23169.55)
Low SDI	30 to 34	25576.45 (25488.54, 25664.67)
Low SDI	35 to 39	27079.94 (26967.65, 27192.69)
Low SDI	40 to 44	29406.99 (29275.94, 29538.63)
Low SDI	45 to 49	31461.55 (31307.41, 31616.45)

CLD, chronic liver disease; SDI, sociodemographic index; WCBA, women of childbearing age.

Table S6 Period effects on CLD prevalence in WCBA across SDI quintiles.

Location	Period	Prevalence rate ratio
Global	1992 to 1996	1.032 (1.023, 1.041)
Global	1997 to 2001	1.023 (1.015, 1.032)
Global	2002 to 2006	0.997 (0.989, 1.005)
Global	2007 to 2011	1.000 (1.000, 1.000)
Global	2012 to 2016	1.023 (1.015, 1.031)
Global	2017 to 2021	1.053 (1.044, 1.061)
High SDI	1992 to 1996	0.938 (0.926, 0.950)
High SDI	1997 to 2001	0.948 (0.936, 0.959)
High SDI	2002 to 2006	0.956 (0.945, 0.968)
High SDI	2007 to 2011	1.000 (1.000, 1.000)
High SDI	2012 to 2016	1.055 (1.044, 1.067)
High SDI	2017 to 2021	1.098 (1.086, 1.110)
High-middle SDI	1992 to 1996	1.091 (1.067, 1.115)
High-middle SDI	1997 to 2001	1.077 (1.055, 1.100)
High-middle SDI	2002 to 2006	1.010 (0.990, 1.030)
High-middle SDI	2007 to 2011	1.000 (1.000, 1.000)
High-middle SDI	2012 to 2016	1.022 (1.003, 1.043)
High-middle SDI	2017 to 2021	1.054 (1.034, 1.075)
Middle SDI	1992 to 1996	1.100 (1.083, 1.116)
Middle SDI	1997 to 2001	1.066 (1.051, 1.082)
Middle SDI	2002 to 2006	1.014 (1.000, 1.028)
Middle SDI	2007 to 2011	1.000 (1.000, 1.000)

Middle SDI	2012 to 2016	1.009 (0.995, 1.022)
Middle SDI	2017 to 2021	1.036 (1.022, 1.050)
Low-middle SDI	1992 to 1996	1.024 (1.020, 1.028)
Low-middle SDI	1997 to 2001	1.012 (1.008, 1.016)
Low-middle SDI	2002 to 2006	1.001 (0.997, 1.004)
Low-middle SDI	2007 to 2011	1.000 (1.000, 1.000)
Low-middle SDI	2012 to 2016	1.014 (1.010, 1.017)
Low-middle SDI	2017 to 2021	1.026 (1.022, 1.029)
Low SDI	1992 to 1996	1.027 (1.023, 1.031)
Low SDI	1997 to 2001	1.020 (1.016, 1.023)
Low SDI	2002 to 2006	1.011 (1.008, 1.015)
Low SDI	2007 to 2011	1.000 (1.000, 1.000)
Low SDI	2012 to 2016	0.995 (0.991, 0.998)
Low SDI	2017 to 2021	0.997 (0.993, 1.000)

CLD, chronic liver disease; SDI, sociodemographic index; WCBA, women of childbearing age.

Table S7 Cohort effects on CLD prevalence in WCBA across SDI quintiles.

Location	Cohort	Prevalence rate ratio
Global	1942 to 1951	0.876 (0.858, 0.895)
Global	1947 to 1956	0.907 (0.893, 0.921)
Global	1952 to 1961	0.925 (0.913, 0.937)
Global	1957 to 1966	0.945 (0.934, 0.956)
Global	1962 to 1971	0.970 (0.960, 0.980)
Global	1967 to 1976	0.979 (0.970, 0.989)
Global	1972 to 1981	0.992 (0.983, 1.002)
Global	1977 to 1986	0.998 (0.988, 1.008)
Global	1982 to 1991	1.000 (1.000, 1.000)
Global	1987 to 1996	0.972 (0.961, 0.984)
Global	1992 to 2001	0.914 (0.900, 0.927)
Global	1997 to 2006	0.788 (0.770, 0.806)
High SDI	1942 to 1951	0.685 (0.666, 0.704)
High SDI	1947 to 1956	0.735 (0.720, 0.750)
High SDI	1952 to 1961	0.779 (0.765, 0.794)
High SDI	1957 to 1966	0.807 (0.794, 0.821)
High SDI	1962 to 1971	0.837 (0.824, 0.851)
High SDI	1967 to 1976	0.877 (0.864, 0.890)
High SDI	1972 to 1981	0.944 (0.930, 0.958)
High SDI	1977 to 1986	0.991 (0.976, 1.006)
High SDI	1982 to 1991	1.000 (1.000, 1.000)
High SDI	1987 to 1996	0.970 (0.952, 0.988)

High SDI	1992 to 2001	0.930 (0.907, 0.954)
High SDI	1997 to 2006	0.908 (0.872, 0.945)
High-middle SDI	1942 to 1951	0.907 (0.863, 0.953)
High-middle SDI	1947 to 1956	0.938 (0.905, 0.973)
High-middle SDI	1952 to 1961	0.953 (0.923, 0.984)
High-middle SDI	1957 to 1966	0.973 (0.946, 1.002)
High-middle SDI	1962 to 1971	1.001 (0.975, 1.028)
High-middle SDI	1967 to 1976	0.998 (0.973, 1.024)
High-middle SDI	1972 to 1981	0.994 (0.969, 1.019)
High-middle SDI	1977 to 1986	0.994 (0.969, 1.021)
High-middle SDI	1982 to 1991	1.000 (1.000, 1.000)
High-middle SDI	1987 to 1996	0.971 (0.940, 1.003)
High-middle SDI	1992 to 2001	0.828 (0.790, 0.867)
High-middle SDI	1997 to 2006	0.622 (0.573, 0.674)
Middle SDI	1942 to 1951	0.978 (0.943, 1.014)
Middle SDI	1947 to 1956	0.995 (0.969, 1.022)
Middle SDI	1952 to 1961	1.002 (0.980, 1.025)
Middle SDI	1957 to 1966	1.005 (0.985, 1.025)
Middle SDI	1962 to 1971	1.009 (0.991, 1.027)
Middle SDI	1967 to 1976	1.009 (0.992, 1.027)
Middle SDI	1972 to 1981	1.015 (0.998, 1.033)
Middle SDI	1977 to 1986	1.013 (0.995, 1.030)
Middle SDI	1982 to 1991	1.000 (1.000, 1.000)
Middle SDI	1987 to 1996	0.945 (0.925, 0.965)

Middle SDI	1992 to 2001	0.848 (0.824, 0.872)
Middle SDI	1997 to 2006	0.690 (0.659, 0.722)
Low-middle SDI	1942 to 1951	0.938 (0.929, 0.947)
Low-middle SDI	1947 to 1956	0.951 (0.944, 0.957)
Low-middle SDI	1952 to 1961	0.963 (0.957, 0.969)
Low-middle SDI	1957 to 1966	0.969 (0.964, 0.974)
Low-middle SDI	1962 to 1971	0.974 (0.969, 0.978)
Low-middle SDI	1967 to 1976	0.978 (0.974, 0.982)
Low-middle SDI	1972 to 1981	0.988 (0.984, 0.992)
Low-middle SDI	1977 to 1986	0.996 (0.992, 1.000)
Low-middle SDI	1982 to 1991	1.000 (1.000, 1.000)
Low-middle SDI	1987 to 1996	0.985 (0.980, 0.990)
Low-middle SDI	1992 to 2001	0.950 (0.944, 0.956)
Low-middle SDI	1997 to 2006	0.836 (0.828, 0.844)
Low SDI	1942 to 1951	1.003 (0.993, 1.013)
Low SDI	1947 to 1956	1.005 (0.998, 1.012)
Low SDI	1952 to 1961	1.010 (1.004, 1.016)
Low SDI	1957 to 1966	1.011 (1.006, 1.016)
Low SDI	1962 to 1971	1.014 (1.009, 1.018)
Low SDI	1967 to 1976	1.016 (1.012, 1.021)
Low SDI	1972 to 1981	1.015 (1.012, 1.019)
Low SDI	1977 to 1986	1.008 (1.005, 1.012)
Low SDI	1982 to 1991	1.000 (1.000, 1.000)
Low SDI	1987 to 1996	0.984 (0.980, 0.988)

Low SDI	1992 to 2001	0.952 (0.947, 0.957)
Low SDI	1997 to 2006	0.826 (0.821, 0.832)

CLD, chronic liver disease; SDI, sociodemographic index; WCBA, women of childbearing age.

Table S8 Age effects on CLD prevalence in WCBA across countries.

Location	Age	Prevalence rate
Afghanistan	15 to 19	10838.67 (10788.28, 10889.30)
Afghanistan	20 to 24	17581.51 (17512.30, 17650.98)
Afghanistan	25 to 29	24876.62 (24785.06, 24968.51)
Afghanistan	30 to 34	31028.79 (30915.25, 31142.74)
Afghanistan	35 to 39	35180.58 (35025.92, 35335.93)
Afghanistan	40 to 44	40746.54 (40558.76, 40935.20)
Afghanistan	45 to 49	46548.34 (46318.70, 46779.12)
Albania	15 to 19	6981.36 (6834.02, 7131.89)
Albania	20 to 24	12723.12 (12491.61, 12958.92)
Albania	25 to 29	18771.73 (18449.54, 19099.55)
Albania	30 to 34	22492.48 (22107.73, 22883.93)
Albania	35 to 39	24615.70 (24143.23, 25097.41)
Albania	40 to 44	28294.02 (27740.57, 28858.52)
Albania	45 to 49	31617.90 (30977.71, 32271.32)
Algeria	15 to 19	12366.73 (12313.89, 12419.79)
Algeria	20 to 24	20808.28 (20735.20, 20881.61)
Algeria	25 to 29	29832.46 (29736.76, 29928.48)
Algeria	30 to 34	37262.64 (37145.10, 37380.56)
Algeria	35 to 39	42160.34 (41999.67, 42321.62)
Algeria	40 to 44	49050.03 (48853.00, 49247.84)
Algeria	45 to 49	56646.98 (56404.76, 56890.23)
American Samoa	15 to 19	10546.34 (9823.89, 11321.92)

American Samoa	20 to 24	17325.32 (16250.51, 18471.22)
American Samoa	25 to 29	25399.16 (23910.13, 26980.93)
American Samoa	30 to 34	30721.22 (28934.76, 32617.98)
American Samoa	35 to 39	34348.10 (32131.10, 36718.07)
American Samoa	40 to 44	39609.42 (36993.47, 42410.35)
American Samoa	45 to 49	45536.88 (42411.32, 48892.78)
Andorra	15 to 19	5217.32 (4651.10, 5852.49)
Andorra	20 to 24	7583.04 (6917.46, 8312.66)
Andorra	25 to 29	10046.36 (9249.94, 10911.34)
Andorra	30 to 34	11547.61 (10667.83, 12499.94)
Andorra	35 to 39	12227.81 (11184.92, 13367.95)
Andorra	40 to 44	13542.47 (12364.03, 14833.22)
Andorra	45 to 49	15686.88 (14284.52, 17226.92)
Angola	15 to 19	20105.52 (20006.92, 20204.61)
Angola	20 to 24	23049.32 (22946.01, 23153.09)
Angola	25 to 29	26124.66 (26010.97, 26238.86)
Angola	30 to 34	27861.42 (27736.60, 27986.81)
Angola	35 to 39	28684.55 (28528.77, 28841.17)
Angola	40 to 44	30196.56 (30019.81, 30374.36)
Angola	45 to 49	31497.37 (31294.34, 31701.73)
Antigua and Barbuda	15 to 19	6573.07 (6111.90, 7069.04)
Antigua and Barbuda	20 to 24	11709.61 (11047.79, 12411.08)
Antigua and Barbuda	25 to 29	17016.74 (16150.04, 17929.96)
Antigua and Barbuda	30 to 34	20399.39 (19377.78, 21474.85)

Antigua and Barbuda	35 to 39	22472.03 (21196.19, 23824.67)
Antigua and Barbuda	40 to 44	25519.21 (24033.58, 27096.67)
Antigua and Barbuda	45 to 49	28245.40 (26538.63, 30061.94)
Argentina	15 to 19	2978.04 (2949.26, 3007.11)
Argentina	20 to 24	5952.40 (5907.08, 5998.07)
Argentina	25 to 29	8571.51 (8512.19, 8631.25)
Argentina	30 to 34	10230.68 (10160.53, 10301.32)
Argentina	35 to 39	11067.12 (10977.10, 11157.87)
Argentina	40 to 44	12670.78 (12563.91, 12778.56)
Argentina	45 to 49	15017.12 (14884.77, 15150.65)
Armenia	15 to 19	12025.23 (11921.98, 12129.37)
Armenia	20 to 24	17518.01 (17387.43, 17649.56)
Armenia	25 to 29	23896.28 (23731.12, 24062.58)
Armenia	30 to 34	27935.79 (27742.01, 28130.93)
Armenia	35 to 39	30552.15 (30308.00, 30798.28)
Armenia	40 to 44	34060.23 (33777.69, 34345.13)
Armenia	45 to 49	37799.79 (37470.56, 38131.91)
Australia	15 to 19	5376.65 (5259.74, 5496.16)
Australia	20 to 24	7684.63 (7543.08, 7828.83)
Australia	25 to 29	10017.82 (9850.87, 10187.61)
Australia	30 to 34	11129.50 (10948.27, 11313.73)
Australia	35 to 39	11519.27 (11299.71, 11743.09)
Australia	40 to 44	12433.94 (12189.63, 12683.15)
Australia	45 to 49	14216.36 (13927.82, 14510.88)

Austria	15 to 19	4349.37 (4248.88, 4452.23)
Austria	20 to 24	7124.06 (6992.40, 7258.21)
Austria	25 to 29	10098.23 (9932.36, 10266.87)
Austria	30 to 34	12116.28 (11923.15, 12312.55)
Austria	35 to 39	12235.61 (12008.60, 12466.91)
Austria	40 to 44	13980.11 (13716.35, 14248.95)
Austria	45 to 49	17140.16 (16811.35, 17475.41)
Azerbaijan	15 to 19	15810.97 (15684.55, 15938.41)
Azerbaijan	20 to 24	21565.62 (21416.10, 21716.17)
Azerbaijan	25 to 29	27735.64 (27557.99, 27914.43)
Azerbaijan	30 to 34	32212.28 (32006.40, 32419.50)
Azerbaijan	35 to 39	35145.36 (34880.58, 35412.16)
Azerbaijan	40 to 44	39424.86 (39115.09, 39737.08)
Azerbaijan	45 to 49	42275.87 (41925.56, 42629.11)
Bahamas	15 to 19	6324.80 (6096.55, 6561.59)
Bahamas	20 to 24	11116.31 (10793.38, 11448.91)
Bahamas	25 to 29	16362.51 (15933.53, 16803.03)
Bahamas	30 to 34	20038.83 (19526.44, 20564.66)
Bahamas	35 to 39	22841.99 (22171.48, 23532.77)
Bahamas	40 to 44	26836.55 (26030.56, 27667.50)
Bahamas	45 to 49	30925.98 (29959.92, 31923.20)
Bahrain	15 to 19	12787.11 (12575.03, 13002.75)
Bahrain	20 to 24	22461.23 (22184.14, 22741.79)
Bahrain	25 to 29	33007.06 (32661.19, 33356.59)

Bahrain	30 to 34	41441.07 (41013.00, 41873.60)
Bahrain	35 to 39	47036.82 (46452.29, 47628.72)
Bahrain	40 to 44	54073.14 (53362.14, 54793.61)
Bahrain	45 to 49	61565.50 (60700.27, 62443.06)
Bangladesh	15 to 19	8427.37 (8329.58, 8526.30)
Bangladesh	20 to 24	12690.03 (12563.63, 12817.71)
Bangladesh	25 to 29	16911.04 (16753.48, 17070.08)
Bangladesh	30 to 34	19308.42 (19126.85, 19491.72)
Bangladesh	35 to 39	20626.52 (20394.90, 20860.76)
Bangladesh	40 to 44	22931.18 (22659.86, 23205.75)
Bangladesh	45 to 49	25314.26 (24992.33, 25640.33)
Barbados	15 to 19	6476.73 (6196.19, 6769.96)
Barbados	20 to 24	10830.24 (10446.86, 11227.70)
Barbados	25 to 29	15854.32 (15348.41, 16376.90)
Barbados	30 to 34	19870.77 (19254.49, 20506.77)
Barbados	35 to 39	21952.99 (21186.09, 22747.64)
Barbados	40 to 44	25713.39 (24802.25, 26658.00)
Barbados	45 to 49	28701.98 (27660.86, 29782.29)
Belarus	15 to 19	8120.57 (8065.55, 8175.96)
Belarus	20 to 24	9906.37 (9845.46, 9967.67)
Belarus	25 to 29	13199.25 (13124.69, 13274.24)
Belarus	30 to 34	15030.43 (14946.18, 15115.17)
Belarus	35 to 39	16221.70 (16118.55, 16325.52)
Belarus	40 to 44	17591.77 (17476.34, 17707.96)

Belarus	45 to 49	19916.63 (19781.22, 20052.96)
Belgium	15 to 19	3847.43 (3793.14, 3902.50)
Belgium	20 to 24	6709.47 (6634.42, 6785.36)
Belgium	25 to 29	9830.68 (9733.01, 9929.33)
Belgium	30 to 34	11256.50 (11145.75, 11368.36)
Belgium	35 to 39	11765.12 (11631.89, 11899.89)
Belgium	40 to 44	13098.96 (12947.32, 13252.37)
Belgium	45 to 49	15417.80 (15234.81, 15602.99)
Belize	15 to 19	8377.97 (8120.79, 8643.29)
Belize	20 to 24	14367.97 (14004.96, 14740.38)
Belize	25 to 29	20413.31 (19941.77, 20895.99)
Belize	30 to 34	25137.88 (24563.04, 25726.17)
Belize	35 to 39	28777.35 (27984.52, 29592.63)
Belize	40 to 44	33397.81 (32429.62, 34394.90)
Belize	45 to 49	37481.08 (36320.34, 38678.92)
Benin	15 to 19	10403.97 (10338.56, 10469.80)
Benin	20 to 24	15682.38 (15599.45, 15765.75)
Benin	25 to 29	21184.22 (21080.06, 21288.90)
Benin	30 to 34	24725.66 (24601.80, 24850.14)
Benin	35 to 39	26882.19 (26717.53, 27047.86)
Benin	40 to 44	29793.86 (29597.62, 29991.39)
Benin	45 to 49	32408.81 (32174.15, 32645.18)
Bermuda	15 to 19	6426.38 (5818.80, 7097.39)
Bermuda	20 to 24	11713.67 (10841.35, 12656.18)

Bermuda	25 to 29	17515.25 (16360.87, 18751.08)
Bermuda	30 to 34	21551.37 (20174.68, 23022.00)
Bermuda	35 to 39	24335.00 (22613.21, 26187.89)
Bermuda	40 to 44	27819.95 (25825.55, 29968.37)
Bermuda	45 to 49	31597.74 (29282.53, 34096.00)
Bhutan	15 to 19	10310.68 (10111.44, 10513.86)
Bhutan	20 to 24	15078.71 (14818.16, 15343.85)
Bhutan	25 to 29	19519.91 (19201.86, 19843.22)
Bhutan	30 to 34	22091.35 (21728.80, 22459.95)
Bhutan	35 to 39	23668.47 (23200.75, 24145.62)
Bhutan	40 to 44	26312.41 (25757.73, 26879.04)
Bhutan	45 to 49	29079.16 (28417.69, 29756.02)
Bolivia (Plurinational State of)	15 to 19	5751.83 (5691.68, 5812.61)
Bolivia (Plurinational State of)	20 to 24	8901.70 (8821.90, 8982.23)
Bolivia (Plurinational State of)	25 to 29	12930.03 (12823.21, 13037.73)
Bolivia (Plurinational State of)	30 to 34	15492.41 (15364.10, 15621.80)
Bolivia (Plurinational State of)	35 to 39	17733.59 (17559.28, 17909.64)
Bolivia (Plurinational State of)	40 to 44	20286.02 (20076.79, 20497.43)
Bolivia (Plurinational State of)	45 to 49	23168.88 (22914.91, 23425.66)
Bosnia and Herzegovina	15 to 19	7133.04 (7056.03, 7210.88)
Bosnia and Herzegovina	20 to 24	14327.04 (14204.10, 14451.04)
Bosnia and Herzegovina	25 to 29	21525.01 (21355.40, 21695.97)
Bosnia and Herzegovina	30 to 34	25693.16 (25490.16, 25897.78)
Bosnia and Herzegovina	35 to 39	28400.54 (28150.71, 28652.59)

Bosnia and Herzegovina	40 to 44	31554.56 (31271.81, 31839.88)
Bosnia and Herzegovina	45 to 49	35295.02 (34967.00, 35626.11)
Botswana	15 to 19	10379.35 (9996.20, 10777.19)
Botswana	20 to 24	15908.95 (15405.19, 16429.17)
Botswana	25 to 29	20856.93 (20247.44, 21484.77)
Botswana	30 to 34	23933.57 (23240.10, 24647.73)
Botswana	35 to 39	26248.70 (25330.15, 27200.57)
Botswana	40 to 44	28977.69 (27894.08, 30103.40)
Botswana	45 to 49	31839.34 (30550.60, 33182.45)
Brazil	15 to 19	8157.18 (8051.24, 8264.51)
Brazil	20 to 24	14106.10 (13955.66, 14258.15)
Brazil	25 to 29	20142.74 (19946.85, 20340.55)
Brazil	30 to 34	24566.41 (24332.03, 24803.05)
Brazil	35 to 39	27733.82 (27426.34, 28044.74)
Brazil	40 to 44	31637.41 (31274.30, 32004.73)
Brazil	45 to 49	35209.13 (34784.88, 35638.56)
Brunei Darussalam	15 to 19	5270.88 (4976.62, 5582.53)
Brunei Darussalam	20 to 24	7882.90 (7517.78, 8265.75)
Brunei Darussalam	25 to 29	10491.46 (10051.49, 10950.68)
Brunei Darussalam	30 to 34	11860.62 (11370.50, 12371.86)
Brunei Darussalam	35 to 39	11955.89 (11382.16, 12558.55)
Brunei Darussalam	40 to 44	13218.24 (12560.42, 13910.51)
Brunei Darussalam	45 to 49	15303.41 (14497.84, 16153.75)
Bulgaria	15 to 19	7511.04 (7274.61, 7755.15)

Bulgaria	20 to 24	11316.17 (11012.07, 11628.66)
Bulgaria	25 to 29	15483.27 (15098.48, 15877.88)
Bulgaria	30 to 34	17717.70 (17282.48, 18163.89)
Bulgaria	35 to 39	19248.55 (18720.69, 19791.30)
Bulgaria	40 to 44	20939.73 (20353.47, 21542.88)
Bulgaria	45 to 49	22641.32 (21982.96, 23319.40)
Burkina Faso	15 to 19	20936.03 (20844.96, 21027.49)
Burkina Faso	20 to 24	23906.06 (23809.49, 24003.02)
Burkina Faso	25 to 29	26437.08 (26331.97, 26542.61)
Burkina Faso	30 to 34	28042.80 (27927.51, 28158.56)
Burkina Faso	35 to 39	28133.72 (27992.05, 28276.10)
Burkina Faso	40 to 44	28958.49 (28798.38, 29119.49)
Burkina Faso	45 to 49	29597.06 (29414.43, 29780.82)
Burundi	15 to 19	11330.88 (11270.58, 11391.51)
Burundi	20 to 24	15788.45 (15714.47, 15862.78)
Burundi	25 to 29	20511.55 (20420.66, 20602.84)
Burundi	30 to 34	23392.42 (23287.11, 23498.20)
Burundi	35 to 39	25253.60 (25112.80, 25395.20)
Burundi	40 to 44	27943.43 (27775.04, 28112.85)
Burundi	45 to 49	30405.82 (30204.73, 30608.24)
Cabo Verde	15 to 19	15399.62 (15116.57, 15687.96)
Cabo Verde	20 to 24	20530.32 (20183.15, 20883.46)
Cabo Verde	25 to 29	25775.11 (25358.89, 26198.16)
Cabo Verde	30 to 34	28662.31 (28195.94, 29136.40)

Cabo Verde	35 to 39	30120.76 (29528.69, 30724.70)
Cabo Verde	40 to 44	32515.81 (31831.81, 33214.52)
Cabo Verde	45 to 49	34458.98 (33678.05, 35258.02)
Cambodia	15 to 19	17989.53 (17910.27, 18069.14)
Cambodia	20 to 24	20964.37 (20877.86, 21051.23)
Cambodia	25 to 29	24720.62 (24622.42, 24819.21)
Cambodia	30 to 34	26693.43 (26585.61, 26801.69)
Cambodia	35 to 39	27433.41 (27301.52, 27565.94)
Cambodia	40 to 44	29425.41 (29275.69, 29575.91)
Cambodia	45 to 49	31326.94 (31156.40, 31498.42)
Cameroon	15 to 19	17218.27 (17118.62, 17318.50)
Cameroon	20 to 24	21628.33 (21517.46, 21739.78)
Cameroon	25 to 29	26031.69 (25904.28, 26159.73)
Cameroon	30 to 34	28536.47 (28393.23, 28680.43)
Cameroon	35 to 39	29856.15 (29669.86, 30043.62)
Cameroon	40 to 44	31899.42 (31682.78, 32117.54)
Cameroon	45 to 49	33737.75 (33484.12, 33993.31)
Canada	15 to 19	5526.64 (5452.94, 5601.34)
Canada	20 to 24	7359.36 (7273.63, 7446.11)
Canada	25 to 29	9512.95 (9410.76, 9616.25)
Canada	30 to 34	10310.84 (10201.32, 10421.54)
Canada	35 to 39	9941.64 (9819.99, 10064.80)
Canada	40 to 44	10631.18 (10497.80, 10766.25)
Canada	45 to 49	11763.92 (11611.43, 11918.41)

Central African Republic	15 to 19	19007.23 (18889.44, 19125.76)
Central African Republic	20 to 24	22078.17 (21951.55, 22205.52)
Central African Republic	25 to 29	25267.67 (25124.83, 25411.33)
Central African Republic	30 to 34	27262.87 (27102.88, 27423.79)
Central African Republic	35 to 39	28496.68 (28296.30, 28698.47)
Central African Republic	40 to 44	30433.73 (30202.95, 30666.29)
Central African Republic	45 to 49	32138.86 (31870.33, 32409.65)
Chad	15 to 19	19296.16 (19191.05, 19401.84)
Chad	20 to 24	22666.34 (22552.16, 22781.10)
Chad	25 to 29	25946.27 (25818.08, 26075.10)
Chad	30 to 34	27729.07 (27587.61, 27871.25)
Chad	35 to 39	28653.24 (28475.08, 28832.52)
Chad	40 to 44	30190.16 (29985.72, 30395.98)
Chad	45 to 49	31252.83 (31015.89, 31491.57)
Chile	15 to 19	3803.72 (3771.42, 3836.30)
Chile	20 to 24	7009.60 (6962.33, 7057.19)
Chile	25 to 29	10072.16 (10010.46, 10134.24)
Chile	30 to 34	11925.86 (11853.47, 11998.69)
Chile	35 to 39	12814.57 (12723.81, 12905.97)
Chile	40 to 44	14558.25 (14452.08, 14665.19)
Chile	45 to 49	17114.56 (16985.20, 17244.90)
China	15 to 19	12892.29 (12393.31, 13411.37)
China	20 to 24	17020.53 (16441.12, 17620.37)
China	25 to 29	21742.69 (21066.82, 22440.24)

China	30 to 34	24180.21 (23438.49, 24945.41)
China	35 to 39	25368.32 (24472.22, 26297.23)
China	40 to 44	27377.70 (26386.65, 28405.97)
China	45 to 49	30031.38 (28909.70, 31196.58)
Colombia	15 to 19	14124.91 (13709.07, 14553.38)
Colombia	20 to 24	19251.96 (18745.95, 19771.64)
Colombia	25 to 29	24603.90 (23994.49, 25228.80)
Colombia	30 to 34	27955.67 (27269.13, 28659.50)
Colombia	35 to 39	30117.38 (29262.35, 30997.39)
Colombia	40 to 44	33099.00 (32120.08, 34107.75)
Colombia	45 to 49	35929.61 (34809.16, 37086.13)
Comoros	15 to 19	11999.93 (11772.58, 12231.68)
Comoros	20 to 24	18001.89 (17703.14, 18305.67)
Comoros	25 to 29	24095.23 (23719.10, 24477.33)
Comoros	30 to 34	27820.77 (27383.81, 28264.70)
Comoros	35 to 39	30203.92 (29642.24, 30776.24)
Comoros	40 to 44	33468.91 (32814.34, 34136.54)
Comoros	45 to 49	36112.77 (35353.83, 36888.01)
Congo	15 to 19	16460.86 (16349.10, 16573.38)
Congo	20 to 24	19508.42 (19386.97, 19630.64)
Congo	25 to 29	22582.88 (22446.85, 22719.74)
Congo	30 to 34	24494.15 (24344.46, 24644.75)
Congo	35 to 39	25705.88 (25517.72, 25895.43)
Congo	40 to 44	27844.65 (27626.28, 28064.74)

Congo	45 to 49	29406.08 (29151.29, 29663.10)
Cook Islands	15 to 19	7975.88 (6927.91, 9182.37)
Cook Islands	20 to 24	12963.78 (11492.15, 14623.85)
Cook Islands	25 to 29	18947.24 (16938.39, 21194.34)
Cook Islands	30 to 34	22427.79 (20041.22, 25098.57)
Cook Islands	35 to 39	24501.32 (21620.40, 27766.12)
Cook Islands	40 to 44	27885.88 (24555.42, 31668.04)
Cook Islands	45 to 49	31790.76 (27868.99, 36264.42)
Costa Rica	15 to 19	8524.00 (8395.46, 8654.51)
Costa Rica	20 to 24	13004.06 (12836.94, 13173.36)
Costa Rica	25 to 29	18343.24 (18128.20, 18560.82)
Costa Rica	30 to 34	22862.39 (22600.21, 23127.61)
Costa Rica	35 to 39	25144.44 (24804.50, 25489.04)
Costa Rica	40 to 44	28272.71 (27874.70, 28676.40)
Costa Rica	45 to 49	32413.39 (31935.12, 32898.82)
Côte d'Ivoire	15 to 19	15854.56 (15784.50, 15924.92)
Côte d'Ivoire	20 to 24	19733.84 (19655.98, 19812.01)
Côte d'Ivoire	25 to 29	23785.85 (23695.79, 23876.24)
Côte d'Ivoire	30 to 34	25828.02 (25727.10, 25929.34)
Côte d'Ivoire	35 to 39	26974.90 (26845.70, 27104.73)
Côte d'Ivoire	40 to 44	28681.88 (28530.77, 28833.78)
Côte d'Ivoire	45 to 49	30296.84 (30118.42, 30476.31)
Croatia	15 to 19	5229.21 (5122.92, 5337.70)
Croatia	20 to 24	9671.22 (9514.03, 9831.01)

Croatia	25 to 29	14463.94 (14252.04, 14678.98)
Croatia	30 to 34	17867.69 (17613.12, 18125.95)
Croatia	35 to 39	19097.03 (18789.03, 19410.08)
Croatia	40 to 44	21812.90 (21455.85, 22175.90)
Croatia	45 to 49	23592.80 (23194.27, 23998.18)
Cuba	15 to 19	6197.17 (6064.60, 6332.65)
Cuba	20 to 24	11346.68 (11154.78, 11541.89)
Cuba	25 to 29	16971.55 (16713.45, 17233.64)
Cuba	30 to 34	20819.43 (20509.41, 21134.15)
Cuba	35 to 39	23541.34 (23149.02, 23940.31)
Cuba	40 to 44	27094.42 (26637.07, 27559.62)
Cuba	45 to 49	30775.24 (30244.93, 31314.86)
Cyprus	15 to 19	4311.14 (4193.17, 4432.42)
Cyprus	20 to 24	7210.73 (7057.65, 7367.12)
Cyprus	25 to 29	10346.08 (10156.42, 10539.28)
Cyprus	30 to 34	12914.07 (12689.49, 13142.62)
Cyprus	35 to 39	13804.49 (13517.11, 14097.98)
Cyprus	40 to 44	15546.67 (15210.78, 15889.97)
Cyprus	45 to 49	17779.62 (17378.93, 18189.55)
Czechia	15 to 19	4717.86 (4653.08, 4783.54)
Czechia	20 to 24	7874.14 (7786.44, 7962.82)
Czechia	25 to 29	11784.23 (11666.35, 11903.30)
Czechia	30 to 34	13697.94 (13563.01, 13834.20)
Czechia	35 to 39	14955.42 (14788.27, 15124.45)

Czechia	40 to 44	17220.68 (17025.35, 17418.25)
Czechia	45 to 49	19267.34 (19041.21, 19496.16)
Democratic People's Republic of Korea	15 to 19	17459.57 (17320.48, 17599.78)
Democratic People's Republic of Korea	20 to 24	21695.51 (21539.39, 21852.76)
Democratic People's Republic of Korea	25 to 29	27149.49 (26965.89, 27334.35)
Democratic People's Republic of Korea	30 to 34	30163.21 (29959.15, 30368.66)
Democratic People's Republic of Korea	35 to 39	31905.40 (31660.90, 32151.78)
Democratic People's Republic of Korea	40 to 44	34554.18 (34281.70, 34828.83)
Democratic People's Republic of Korea	45 to 49	37684.98 (37375.30, 37997.22)
Democratic Republic of the Congo	15 to 19	24433.60 (24326.84, 24540.83)
Democratic Republic of the Congo	20 to 24	27408.44 (27296.34, 27521.00)
Democratic Republic of the Congo	25 to 29	30745.94 (30621.66, 30870.72)
Democratic Republic of the Congo	30 to 34	32746.51 (32609.56, 32884.03)
Democratic Republic of the Congo	35 to 39	33964.52 (33793.19, 34136.72)
Democratic Republic of the Congo	40 to 44	35994.94 (35799.12, 36191.84)
Democratic Republic of the Congo	45 to 49	37825.04 (37599, 38052.44)
Denmark	15 to 19	3288.37 (3235.33, 3342.28)
Denmark	20 to 24	5356.58 (5286.18, 5427.93)
Denmark	25 to 29	7762.73 (7670.75, 7855.81)
Denmark	30 to 34	9042.92 (8937.30, 9149.80)
Denmark	35 to 39	9628.64 (9500.44, 9758.58)
Denmark	40 to 44	10212.32 (10073.04, 10353.52)
Denmark	45 to 49	12263.01 (12092.09, 12436.34)
Djibouti	15 to 19	11584.70 (11386.68, 11786.17)

Djibouti	20 to 24	16258.85 (16025.16, 16495.96)
Djibouti	25 to 29	21336.89 (21056.80, 21620.71)
Djibouti	30 to 34	24465.89 (24146.33, 24789.69)
Djibouti	35 to 39	26515.65 (26088.16, 26950.14)
Djibouti	40 to 44	29413.56 (28899.76, 29936.50)
Djibouti	45 to 49	32123.91 (31507.39, 32752.48)
Dominica	15 to 19	6787.80 (6284.14, 7331.83)
Dominica	20 to 24	12063.16 (11294.65, 12883.97)
Dominica	25 to 29	17638.77 (16603.52, 18738.56)
Dominica	30 to 34	21364.35 (20130.82, 22673.45)
Dominica	35 to 39	23887.29 (22312.77, 25572.92)
Dominica	40 to 44	27211.30 (25365.86, 29191.01)
Dominica	45 to 49	30408.98 (28273.50, 32705.76)
Dominican Republic	15 to 19	6360.06 (6313.76, 6406.70)
Dominican Republic	20 to 24	10765.54 (10700.41, 10831.06)
Dominican Republic	25 to 29	15321.16 (15235.70, 15407.09)
Dominican Republic	30 to 34	18862.50 (18758.96, 18966.60)
Dominican Republic	35 to 39	21676.83 (21537.88, 21816.68)
Dominican Republic	40 to 44	25487.53 (25318.55, 25657.63)
Dominican Republic	45 to 49	29553.47 (29347.83, 29760.55)
Ecuador	15 to 19	5998.88 (5957.92, 6040.11)
Ecuador	20 to 24	13974.53 (13903.39, 14046.03)
Ecuador	25 to 29	21399.02 (21300.74, 21497.75)
Ecuador	30 to 34	25464.94 (25348.66, 25581.74)

Ecuador	35 to 39	28326.65 (28173.26, 28480.88)
Ecuador	40 to 44	32371.49 (32189.14, 32554.87)
Ecuador	45 to 49	37458.70 (37237.47, 37681.24)
Egypt	15 to 19	17161.10 (16631.53, 17707.53)
Egypt	20 to 24	27269.10 (26563.51, 27993.43)
Egypt	25 to 29	37339.62 (36439.83, 38261.64)
Egypt	30 to 34	45088.56 (44004.10, 46199.75)
Egypt	35 to 39	50091.48 (48660.26, 51564.79)
Egypt	40 to 44	56581.90 (54882.10, 58334.35)
Egypt	45 to 49	62450.45 (60453.67, 64513.18)
El Salvador	15 to 19	8729.89 (8577.04, 8885.47)
El Salvador	20 to 24	14217.15 (14002.97, 14434.61)
El Salvador	25 to 29	20286.58 (20000.67, 20576.59)
El Salvador	30 to 34	24333.62 (23990.78, 24681.36)
El Salvador	35 to 39	27063.84 (26621.79, 27513.23)
El Salvador	40 to 44	30746.84 (30225.61, 31277.05)
El Salvador	45 to 49	34226.49 (33611.68, 34852.54)
Equatorial Guinea	15 to 19	11518.16 (11279.21, 11762.16)
Equatorial Guinea	20 to 24	15154.60 (14879.34, 15434.94)
Equatorial Guinea	25 to 29	18793.62 (18473.63, 19119.16)
Equatorial Guinea	30 to 34	20857.15 (20499.18, 21221.37)
Equatorial Guinea	35 to 39	22062.95 (21594.96, 22541.08)
Equatorial Guinea	40 to 44	24136.53 (23584.96, 24701.01)
Equatorial Guinea	45 to 49	26106.20 (25452.79, 26776.38)

Eritrea	15 to 19	12204.20 (12119.90, 12289.08)
Eritrea	20 to 24	16419.69 (16318.80, 16521.20)
Eritrea	25 to 29	20827.67 (20705.75, 20950.30)
Eritrea	30 to 34	23473.93 (23334.73, 23613.95)
Eritrea	35 to 39	24998.80 (24820.20, 25178.68)
Eritrea	40 to 44	27202.54 (26995.26, 27411.40)
Eritrea	45 to 49	28965.14 (28724.75, 29207.54)
Estonia	15 to 19	6488.07 (6353.67, 6625.31)
Estonia	20 to 24	8697.40 (8538.78, 8858.97)
Estonia	25 to 29	11830.97 (11632.27, 12033.06)
Estonia	30 to 34	13756.51 (13527.84, 13989.04)
Estonia	35 to 39	15161.74 (14877.52, 15451.39)
Estonia	40 to 44	16621.03 (16301.85, 16946.46)
Estonia	45 to 49	18399.64 (18033.49, 18773.23)
Eswatini	15 to 19	12520.00 (12253.56, 12792.23)
Eswatini	20 to 24	18529.53 (18177.87, 18887.99)
Eswatini	25 to 29	24033.57 (23596.85, 24478.38)
Eswatini	30 to 34	27253.55 (26744.37, 27772.43)
Eswatini	35 to 39	29202.89 (28548.06, 29872.73)
Eswatini	40 to 44	31993.77 (31219.94, 32786.77)
Eswatini	45 to 49	34144.13 (33233.35, 35079.87)
Ethiopia	15 to 19	11206.72 (11109.17, 11305.12)
Ethiopia	20 to 24	15029.77 (14910.71, 15149.78)
Ethiopia	25 to 29	18857.91 (18713.00, 19003.94)

Ethiopia	30 to 34	21176.31 (21011.18, 21342.74)
Ethiopia	35 to 39	22586.32 (22374.20, 22800.46)
Ethiopia	40 to 44	24796.39 (24545.47, 25049.88)
Ethiopia	45 to 49	25659.58 (25367.12, 25955.41)
Fiji	15 to 19	7767.79 (7275.97, 8292.85)
Fiji	20 to 24	15324.24 (14541.84, 16148.74)
Fiji	25 to 29	22796.98 (21730.15, 23916.19)
Fiji	30 to 34	27098.92 (25842.92, 28415.95)
Fiji	35 to 39	29829.51 (28250.42, 31496.87)
Fiji	40 to 44	34286.21 (32409.76, 36271.29)
Fiji	45 to 49	38973.20 (36744.78, 41336.77)
Finland	15 to 19	3811.78 (3756.64, 3867.72)
Finland	20 to 24	5551.76 (5482.93, 5621.45)
Finland	25 to 29	7711.79 (7624.77, 7799.81)
Finland	30 to 34	8924.47 (8825.02, 9025.04)
Finland	35 to 39	9243.82 (9125.41, 9363.77)
Finland	40 to 44	10267.28 (10132.72, 10403.62)
Finland	45 to 49	11842.70 (11682.90, 12004.69)
France	15 to 19	4078.18 (3988.46, 4169.93)
France	20 to 24	6386.63 (6268.77, 6506.71)
France	25 to 29	9238.92 (9084.45, 9396.01)
France	30 to 34	11091.29 (10910.38, 11275.20)
France	35 to 39	11164.06 (10953.04, 11379.14)
France	40 to 44	12522.11 (12280.07, 12768.93)

France	45 to 49	14989.39 (14692.45, 15292.33)
Gabon	15 to 19	15544.69 (15330.03, 15762.36)
Gabon	20 to 24	19150.74 (18908.59, 19395.99)
Gabon	25 to 29	22987.98 (22708.00, 23271.42)
Gabon	30 to 34	25379.31 (25064.99, 25697.57)
Gabon	35 to 39	26797.06 (26399.46, 27200.64)
Gabon	40 to 44	29257.69 (28796.75, 29726.01)
Gabon	45 to 49	31698.71 (31159.51, 32247.24)
Gambia	15 to 19	9575.96 (8960.93, 10233.19)
Gambia	20 to 24	14343.76 (13549.66, 15184.40)
Gambia	25 to 29	18816.73 (17839.56, 19847.43)
Gambia	30 to 34	21529.55 (20422.60, 22696.49)
Gambia	35 to 39	22295.66 (20915.96, 23766.37)
Gambia	40 to 44	24353.59 (22733.81, 26088.78)
Gambia	45 to 49	25922.86 (24017.36, 27979.53)
Georgia	15 to 19	11150.87 (11056.83, 11245.72)
Georgia	20 to 24	16105.23 (15985.06, 16226.31)
Georgia	25 to 29	21685.55 (21533.52, 21838.66)
Georgia	30 to 34	25068.81 (24892.57, 25246.30)
Georgia	35 to 39	27044.97 (26833.32, 27258.28)
Georgia	40 to 44	29834.09 (29593.50, 30076.63)
Georgia	45 to 49	32446.67 (32172.78, 32722.88)
Germany	15 to 19	3556.88 (3511.20, 3603.15)
Germany	20 to 24	5728.92 (5669.31, 5789.15)

Germany	25 to 29	8196.95 (8120.96, 8273.66)
Germany	30 to 34	9882.75 (9793.90, 9972.41)
Germany	35 to 39	10805.25 (10693.97, 10917.69)
Germany	40 to 44	11949.40 (11823.68, 12076.45)
Germany	45 to 49	14345.89 (14192.11, 14501.34)
Ghana	15 to 19	20623.86 (20396.94, 20853.31)
Ghana	20 to 24	24651.44 (24408.82, 24896.47)
Ghana	25 to 29	29255.06 (28980.99, 29531.71)
Ghana	30 to 34	31102.21 (30804.85, 31402.44)
Ghana	35 to 39	32353.54 (31978.09, 32733.40)
Ghana	40 to 44	34165.40 (33738.80, 34597.39)
Ghana	45 to 49	36156.26 (35661.54, 36657.85)
Greece	15 to 19	4695.42 (4618.44, 4773.68)
Greece	20 to 24	7315.30 (7214.70, 7417.30)
Greece	25 to 29	10032.53 (9904.96, 10161.75)
Greece	30 to 34	11932.14 (11782.72, 12083.46)
Greece	35 to 39	13016.69 (12835.43, 13200.51)
Greece	40 to 44	14969.59 (14757.27, 15184.96)
Greece	45 to 49	18155.59 (17891.60, 18423.48)
Greenland	15 to 19	5957.91 (5354.78, 6628.97)
Greenland	20 to 24	7892.16 (7182.42, 8672.03)
Greenland	25 to 29	10301.72 (9444.82, 11236.37)
Greenland	30 to 34	11204.13 (10264.28, 12230.03)
Greenland	35 to 39	11407.38 (10316.07, 12614.15)

Greenland	40 to 44	12421.74 (11191.65, 13787.02)
Greenland	45 to 49	14005.10 (12577.93, 15594.21)
Grenada	15 to 19	6513.84 (6121.97, 6930.78)
Grenada	20 to 24	11228.02 (10669.49, 11815.78)
Grenada	25 to 29	16008.97 (15258.33, 16796.54)
Grenada	30 to 34	19311.73 (18375.65, 20295.50)
Grenada	35 to 39	21728.36 (20514.15, 23014.44)
Grenada	40 to 44	25114.01 (23680.24, 26634.60)
Grenada	45 to 49	28447.70 (26768.70, 30232.01)
Guam	15 to 19	7976.78 (7582.11, 8391.98)
Guam	20 to 24	12613.17 (12075.81, 13174.44)
Guam	25 to 29	18016.58 (17302.13, 18760.53)
Guam	30 to 34	21161.14 (20325.21, 22031.44)
Guam	35 to 39	23786.60 (22745.75, 24875.07)
Guam	40 to 44	28417.25 (27151.60, 29741.90)
Guam	45 to 49	32693.63 (31195.08, 34264.16)
Guatemala	15 to 19	10256.18 (10111.96, 10402.45)
Guatemala	20 to 24	15997.39 (15801.20, 16196.01)
Guatemala	25 to 29	22075.48 (21820.35, 22333.60)
Guatemala	30 to 34	25877.53 (25575.77, 26182.86)
Guatemala	35 to 39	28449.33 (28052.13, 28852.15)
Guatemala	40 to 44	32082.76 (31609.37, 32563.24)
Guatemala	45 to 49	35488.08 (34926.64, 36058.56)
Guinea	15 to 19	19610.01 (19496.27, 19724.41)

Guinea	20 to 24	24515.51 (24386.20, 24645.51)
Guinea	25 to 29	29540.14 (29390.12, 29690.92)
Guinea	30 to 34	32345.13 (32177.68, 32513.46)
Guinea	35 to 39	33933.97 (33719.50, 34149.80)
Guinea	40 to 44	36252.38 (36004.87, 36501.60)
Guinea	45 to 49	38151.79 (37863.28, 38442.49)
Guinea-Bissau	15 to 19	18717.75 (18531.51, 18905.88)
Guinea-Bissau	20 to 24	23193.76 (22983.31, 23406.13)
Guinea-Bissau	25 to 29	27724.95 (27482.47, 27969.56)
Guinea-Bissau	30 to 34	30171.31 (29899.11, 30445.99)
Guinea-Bissau	35 to 39	31627.46 (31276.55, 31982.31)
Guinea-Bissau	40 to 44	33634.53 (33226.67, 34047.40)
Guinea-Bissau	45 to 49	35048.72 (34572.29, 35531.71)
Guyana	15 to 19	7426.76 (7263.94, 7593.23)
Guyana	20 to 24	12003.26 (11773.36, 12237.66)
Guyana	25 to 29	16832.54 (16527.24, 17143.49)
Guyana	30 to 34	20525.23 (20156.51, 20900.69)
Guyana	35 to 39	23003.93 (22534.37, 23483.27)
Guyana	40 to 44	26060.33 (25511.17, 26621.31)
Guyana	45 to 49	29431.46 (28778.72, 30099.00)
Haiti	15 to 19	7973.06 (7927.25, 8019.12)
Haiti	20 to 24	12881.85 (12820.24, 12943.76)
Haiti	25 to 29	17907.36 (17828.61, 17986.46)
Haiti	30 to 34	21264.04 (21170.88, 21357.61)

Haiti	35 to 39	23655.30 (23530.00, 23781.27)
Haiti	40 to 44	26648.07 (26498.39, 26798.60)
Haiti	45 to 49	29146.43 (28969.60, 29324.34)
Honduras	15 to 19	10552.11 (10271.25, 10840.64)
Honduras	20 to 24	17307.03 (16916.38, 17706.71)
Honduras	25 to 29	23808.84 (23309.84, 24318.52)
Honduras	30 to 34	28105.43 (27513.14, 28710.48)
Honduras	35 to 39	30885.36 (30096.28, 31695.14)
Honduras	40 to 44	34712.45 (33774.12, 35676.85)
Honduras	45 to 49	38025.45 (36915.94, 39168.30)
Hungary	15 to 19	4921.23 (4848.74, 4994.79)
Hungary	20 to 24	9366.38 (9256.41, 9477.66)
Hungary	25 to 29	14394.31 (14240.86, 14549.41)
Hungary	30 to 34	16914.00 (16736.73, 17093.14)
Hungary	35 to 39	18748.66 (18528.38, 18971.56)
Hungary	40 to 44	20992.58 (20742.70, 21245.47)
Hungary	45 to 49	23695.56 (23404.94, 23989.79)
Iceland	15 to 19	4422.52 (4196.06, 4661.20)
Iceland	20 to 24	6040.84 (5773.02, 6321.08)
Iceland	25 to 29	8498.87 (8153.62, 8858.74)
Iceland	30 to 34	9870.81 (9472.27, 10286.12)
Iceland	35 to 39	10429.29 (9943.82, 10938.45)
Iceland	40 to 44	11623.89 (11067.60, 12208.13)
Iceland	45 to 49	13560.07 (12889.65, 14265.35)

India	15 to 19	8651.17 (8550.84, 8752.68)
India	20 to 24	13077.58 (12947.83, 13208.64)
India	25 to 29	17326.28 (17166.31, 17487.74)
India	30 to 34	19849.59 (19667.41, 20033.47)
India	35 to 39	21508.67 (21274.97, 21744.95)
India	40 to 44	24004.75 (23730.87, 24281.79)
India	45 to 49	26341.43 (26021.17, 26665.63)
Indonesia	15 to 19	11216.85 (11006.61, 11431.11)
Indonesia	20 to 24	16862.02 (16591.67, 17136.76)
Indonesia	25 to 29	23038.35 (22699.34, 23382.43)
Indonesia	30 to 34	26870.66 (26482.64, 27264.36)
Indonesia	35 to 39	29325.93 (28833.56, 29826.72)
Indonesia	40 to 44	32974.05 (32401.23, 33556.98)
Indonesia	45 to 49	36811.57 (36137.63, 37498.07)
Iran (Islamic Republic of)	15 to 19	11883.74 (11645.76, 12126.59)
Iran (Islamic Republic of)	20 to 24	23006.75 (22649.04, 23370.11)
Iran (Islamic Republic of)	25 to 29	33887.23 (33403.08, 34378.39)
Iran (Islamic Republic of)	30 to 34	42671.50 (42072.29, 43279.25)
Iran (Islamic Republic of)	35 to 39	49357.28 (48519.46, 50209.58)
Iran (Islamic Republic of)	40 to 44	57672.91 (56632.95, 58731.97)
Iran (Islamic Republic of)	45 to 49	66203.45 (64924.23, 67507.87)
Iraq	15 to 19	11337.02 (11284.66, 11389.62)
Iraq	20 to 24	19687.75 (19612.78, 19763.01)
Iraq	25 to 29	28750.81 (28651.65, 28850.31)

Iraq	30 to 34	36163.84 (36041.28, 36286.82)
Iraq	35 to 39	41079.03 (40909.80, 41248.97)
Iraq	40 to 44	47599.70 (47393.64, 47806.66)
Iraq	45 to 49	54459.92 (54207.92, 54713.10)
Ireland	15 to 19	4661.26 (4599.59, 4723.75)
Ireland	20 to 24	6717.19 (6640.95, 6794.30)
Ireland	25 to 29	8916.09 (8823.09, 9010.08)
Ireland	30 to 34	10882.49 (10771.74, 10994.38)
Ireland	35 to 39	11433.19 (11298.94, 11569.05)
Ireland	40 to 44	13258.65 (13098.17, 13421.09)
Ireland	45 to 49	15530.84 (15335.38, 15728.81)
Israel	15 to 19	5020.37 (4957.71, 5083.83)
Israel	20 to 24	8791.12 (8702.05, 8881.10)
Israel	25 to 29	12711.05 (12594.36, 12828.82)
Israel	30 to 34	15258.76 (15120.77, 15398.02)
Israel	35 to 39	16867.23 (16687.56, 17048.84)
Israel	40 to 44	19057.52 (18846.47, 19270.95)
Israel	45 to 49	24188.02 (23911.61, 24467.61)
Italy	15 to 19	6459.16 (6154.94, 6778.41)
Italy	20 to 24	10581.69 (10175.62, 11003.97)
Italy	25 to 29	14747.30 (14235.51, 15277.48)
Italy	30 to 34	16957.61 (16377.28, 17558.51)
Italy	35 to 39	19092.15 (18371.62, 19840.93)
Italy	40 to 44	21047.02 (20240.75, 21885.41)

Italy	45 to 49	24553.05 (23590.47, 25554.90)
Jamaica	15 to 19	7196.08 (7113.77, 7279.35)
Jamaica	20 to 24	12416.34 (12298.69, 12535.12)
Jamaica	25 to 29	17842.53 (17686.57, 17999.86)
Jamaica	30 to 34	21583.66 (21395.28, 21773.70)
Jamaica	35 to 39	24329.93 (24084.17, 24578.19)
Jamaica	40 to 44	28068.78 (27773.67, 28367.02)
Jamaica	45 to 49	31710.04 (31358.90, 32065.12)
Japan	15 to 19	4687.21 (4610.65, 4765.04)
Japan	20 to 24	4671.32 (4598.78, 4745.00)
Japan	25 to 29	5933.01 (5846.52, 6020.78)
Japan	30 to 34	6534.62 (6441.16, 6629.42)
Japan	35 to 39	6953.74 (6843.28, 7065.99)
Japan	40 to 44	7477.55 (7356.98, 7600.09)
Japan	45 to 49	8448.02 (8308.07, 8590.34)
Jordan	15 to 19	15014.30 (14733.57, 15300.37)
Jordan	20 to 24	24607.90 (24234.11, 24987.46)
Jordan	25 to 29	34273.34 (33803.39, 34749.83)
Jordan	30 to 34	41983.80 (41420.84, 42554.40)
Jordan	35 to 39	46920.66 (46141.85, 47712.61)
Jordan	40 to 44	53336.27 (52395.40, 54294.04)
Jordan	45 to 49	60388.42 (59240.82, 61558.24)
Kazakhstan	15 to 19	9465.93 (9391.45, 9541.01)
Kazakhstan	20 to 24	13034.14 (12944.48, 13124.42)

Kazakhstan	25 to 29	17888.53 (17776.33, 18001.44)
Kazakhstan	30 to 34	21059.90 (20929.14, 21191.47)
Kazakhstan	35 to 39	23508.03 (23336.31, 23681.01)
Kazakhstan	40 to 44	26457.52 (26257.43, 26659.13)
Kazakhstan	45 to 49	30240.28 (30000.97, 30481.50)
Kenya	15 to 19	11895.70 (11825.36, 11966.47)
Kenya	20 to 24	17679.72 (17589.25, 17770.66)
Kenya	25 to 29	23520.59 (23406.69, 23635.05)
Kenya	30 to 34	26982.42 (26848.80, 27116.70)
Kenya	35 to 39	29412.38 (29233.90, 29591.96)
Kenya	40 to 44	33226.64 (33009.62, 33445.09)
Kenya	45 to 49	35753.56 (35496.37, 36012.62)
Kiribati	15 to 19	13276.83 (12692.44, 13888.11)
Kiribati	20 to 24	20095.97 (19327.80, 20894.67)
Kiribati	25 to 29	27097.32 (26137.91, 28091.94)
Kiribati	30 to 34	31545.39 (30435.55, 32695.71)
Kiribati	35 to 39	34078.21 (32670.47, 35546.60)
Kiribati	40 to 44	38160.35 (36510.70, 39884.54)
Kiribati	45 to 49	42300.37 (40349.99, 44345.02)
Kuwait	15 to 19	12587.70 (12459.66, 12717.05)
Kuwait	20 to 24	23340.19 (23181.09, 23500.37)
Kuwait	25 to 29	34861.78 (34679.55, 35044.98)
Kuwait	30 to 34	43874.02 (43663.06, 44086.00)
Kuwait	35 to 39	49480.00 (49167.23, 49794.77)

Kuwait	40 to 44	56658.99 (56273.53, 57047.09)
Kuwait	45 to 49	64381.61 (63905.08, 64861.69)
Kyrgyzstan	15 to 19	15114.96 (15010.91, 15219.74)
Kyrgyzstan	20 to 24	20868.95 (20742.50, 20996.18)
Kyrgyzstan	25 to 29	26235.16 (26083.98, 26387.21)
Kyrgyzstan	30 to 34	29663.09 (29489.16, 29838.05)
Kyrgyzstan	35 to 39	31662.56 (31441.71, 31884.96)
Kyrgyzstan	40 to 44	34733.68 (34479.20, 34990.05)
Kyrgyzstan	45 to 49	37292.57 (37000.93, 37586.51)
Lao People's Democratic Republic	15 to 19	13480.94 (13386.48, 13576.08)
Lao People's Democratic Republic	20 to 24	15979.87 (15875.66, 16084.76)
Lao People's Democratic Republic	25 to 29	19356.96 (19235.75, 19478.95)
Lao People's Democratic Republic	30 to 34	21233.12 (21098.36, 21368.73)
Lao People's Democratic Republic	35 to 39	21855.84 (21689.20, 22023.77)
Lao People's Democratic Republic	40 to 44	23699.08 (23506.83, 23892.90)
Lao People's Democratic Republic	45 to 49	25672.88 (25448.34, 25899.41)
Latvia	15 to 19	6442.44 (6338.83, 6547.76)
Latvia	20 to 24	8929.01 (8801.61, 9058.26)
Latvia	25 to 29	11760.34 (11602.17, 11920.68)
Latvia	30 to 34	13596.58 (13413.87, 13781.78)
Latvia	35 to 39	14859.50 (14636.69, 15085.71)
Latvia	40 to 44	16262.60 (16012.40, 16516.71)
Latvia	45 to 49	18278.69 (17987.57, 18574.51)
Lebanon	15 to 19	11845.66 (11756.49, 11935.51)

Lebanon	20 to 24	20596.11 (20473.80, 20719.15)
Lebanon	25 to 29	30348.53 (30194.37, 30503.49)
Lebanon	30 to 34	38023.19 (37837.17, 38210.12)
Lebanon	35 to 39	42593.29 (42336.33, 42851.81)
Lebanon	40 to 44	48849.33 (48541.00, 49159.62)
Lebanon	45 to 49	55674.44 (55305.82, 56045.51)
Lesotho	15 to 19	12293.33 (12159.69, 12428.44)
Lesotho	20 to 24	16581.2 (16416.74, 16747.31)
Lesotho	25 to 29	20776.46 (20574.96, 20979.92)
Lesotho	30 to 34	23353.59 (23119.21, 23590.35)
Lesotho	35 to 39	25375.53 (25075.45, 25679.20)
Lesotho	40 to 44	27506.22 (27158.28, 27858.62)
Lesotho	45 to 49	29956.11 (29547.11, 30370.78)
Liberia	15 to 19	18892.64 (18767.51, 19018.62)
Liberia	20 to 24	23824.40 (23683.54, 23966.11)
Liberia	25 to 29	28725.92 (28564.45, 28888.31)
Liberia	30 to 34	31984.88 (31798.91, 32171.94)
Liberia	35 to 39	34036.85 (33798.20, 34277.18)
Liberia	40 to 44	36760.30 (36481.91, 37040.83)
Liberia	45 to 49	38954.67 (38619.94, 39292.30)
Libya	15 to 19	12233.70 (12115.96, 12352.59)
Libya	20 to 24	21647.01 (21477.66, 21817.70)
Libya	25 to 29	31365.22 (31142.32, 31589.72)
Libya	30 to 34	39045.71 (38773.10, 39320.23)

Libya	35 to 39	43928.02 (43561.38, 44297.74)
Libya	40 to 44	50669.94 (50224.14, 51119.70)
Libya	45 to 49	58011.17 (57462.07, 58565.52)
Lithuania	15 to 19	6790.41 (6700.09, 6881.96)
Lithuania	20 to 24	8159.88 (8058.22, 8262.83)
Lithuania	25 to 29	10952.98 (10823.79, 11083.71)
Lithuania	30 to 34	12819.72 (12669.01, 12972.22)
Lithuania	35 to 39	13788.47 (13608.95, 13970.36)
Lithuania	40 to 44	15339.74 (15135.66, 15546.57)
Lithuania	45 to 49	16671.02 (16441.07, 16904.18)
Luxembourg	15 to 19	4398.03 (4202.62, 4602.52)
Luxembourg	20 to 24	6631.42 (6393.02, 6878.71)
Luxembourg	25 to 29	9326.58 (9040.59, 9621.61)
Luxembourg	30 to 34	10749.31 (10434.60, 11073.50)
Luxembourg	35 to 39	11066.45 (10682.52, 11464.17)
Luxembourg	40 to 44	12726.42 (12275.35, 13194.07)
Luxembourg	45 to 49	14951.07 (14406.49, 15516.24)
Madagascar	15 to 19	16172.01 (16091.70, 16252.71)
Madagascar	20 to 24	19994.54 (19904.07, 20085.42)
Madagascar	25 to 29	24073.39 (23968.27, 24178.97)
Madagascar	30 to 34	26548.07 (26429.95, 26666.72)
Madagascar	35 to 39	27967.47 (27816.66, 28119.10)
Madagascar	40 to 44	30051.65 (29877.17, 30227.15)
Madagascar	45 to 49	31877.25 (31673.62, 32082.19)

Malawi	15 to 19	13285.82 (13191.59, 13380.72)
Malawi	20 to 24	17285.81 (17176.13, 17396.18)
Malawi	25 to 29	21485.67 (21354.45, 21617.68)
Malawi	30 to 34	23998.07 (23848.38, 24148.70)
Malawi	35 to 39	25494.65 (25299.67, 25691.13)
Malawi	40 to 44	27793.44 (27561.76, 28027.07)
Malawi	45 to 49	29887.57 (29610.75, 30166.97)
Malaysia	15 to 19	8956.23 (8787.49, 9128.22)
Malaysia	20 to 24	15670.32 (15432.16, 15912.16)
Malaysia	25 to 29	23176.56 (22858.71, 23498.82)
Malaysia	30 to 34	27470.82 (27096.90, 27849.90)
Malaysia	35 to 39	29802.16 (29322.29, 30289.88)
Malaysia	40 to 44	33572.42 (33008.04, 34146.45)
Malaysia	45 to 49	38044.89 (37369.71, 38732.28)
Maldives	15 to 19	11523.17 (11170.16, 11887.34)
Maldives	20 to 24	16748.79 (16304.61, 17205.08)
Maldives	25 to 29	22658.22 (22112.98, 23216.90)
Maldives	30 to 34	25721.02 (25112.20, 26344.59)
Maldives	35 to 39	27295.20 (26500.25, 28114.00)
Maldives	40 to 44	30173.34 (29234.93, 31141.87)
Maldives	45 to 49	33374.63 (32253.84, 34534.37)
Mali	15 to 19	16709.26 (16623.18, 16795.78)
Mali	20 to 24	21847.96 (21747.86, 21948.53)
Mali	25 to 29	26946.16 (26827.82, 27065.01)

Mali	30 to 34	29986.10 (29851.28, 30121.54)
Mali	35 to 39	31709.48 (31534.32, 31885.61)
Mali	40 to 44	34471.44 (34266.24, 34677.86)
Mali	45 to 49	36899.01 (36657.93, 37141.67)
Malta	15 to 19	4009.40 (3829.22, 4198.05)
Malta	20 to 24	7337.87 (7073.77, 7611.83)
Malta	25 to 29	10167.11 (9833.35, 10512.19)
Malta	30 to 34	12592.45 (12189.52, 13008.69)
Malta	35 to 39	13774.27 (13266.04, 14301.96)
Malta	40 to 44	15875.45 (15277.19, 16497.14)
Malta	45 to 49	18986.43 (18251.97, 19750.45)
Marshall Islands	15 to 19	10535.01 (9842.29, 11276.50)
Marshall Islands	20 to 24	14944.78 (14047.10, 15899.83)
Marshall Islands	25 to 29	20565.21 (19394.23, 21806.89)
Marshall Islands	30 to 34	24161.57 (22787.46, 25618.53)
Marshall Islands	35 to 39	26508.45 (24769.45, 28369.55)
Marshall Islands	40 to 44	30107.57 (28040.14, 32327.43)
Marshall Islands	45 to 49	34334.85 (31823.58, 37044.29)
Mauritania	15 to 19	21184.38 (21042.59, 21327.13)
Mauritania	20 to 24	27534.67 (27368.41, 27701.95)
Mauritania	25 to 29	34108.66 (33910.87, 34307.60)
Mauritania	30 to 34	38172.25 (37946.64, 38399.21)
Mauritania	35 to 39	40591.99 (40304.69, 40881.34)
Mauritania	40 to 44	43872.48 (43541.09, 44206.39)

Mauritania	45 to 49	46430.53 (46046.65, 46817.60)
Mauritius	15 to 19	10162.67 (10000.45, 10327.52)
Mauritius	20 to 24	15101.67 (14891.26, 15315.05)
Mauritius	25 to 29	20666.27 (20398.56, 20937.49)
Mauritius	30 to 34	23932.58 (23623.56, 24245.64)
Mauritius	35 to 39	25709.26 (25335.28, 26088.77)
Mauritius	40 to 44	28652.96 (28225.25, 29087.15)
Mauritius	45 to 49	32272.43 (31772.24, 32780.49)
Mexico	15 to 19	6900.96 (6823.91, 6978.88)
Mexico	20 to 24	12217.79 (12105.93, 12330.69)
Mexico	25 to 29	18303.13 (18150.25, 18457.30)
Mexico	30 to 34	22247.76 (22065.18, 22431.84)
Mexico	35 to 39	24823.87 (24585.43, 25064.63)
Mexico	40 to 44	28515.47 (28231.76, 28802.03)
Mexico	45 to 49	32019.50 (31683.86, 32358.71)
Micronesia (Federated States of)	15 to 19	9315.55 (8856.24, 9798.69)
Micronesia (Federated States of)	20 to 24	13871.98 (13232.92, 14541.91)
Micronesia (Federated States of)	25 to 29	19348.88 (18499.40, 20237.37)
Micronesia (Federated States of)	30 to 34	22768.44 (21769.43, 23813.29)
Micronesia (Federated States of)	35 to 39	24953.64 (23712.73, 26259.50)
Micronesia (Federated States of)	40 to 44	28497.91 (27036.09, 30038.78)
Micronesia (Federated States of)	45 to 49	32613.01 (30858.74, 34467.00)
Monaco	15 to 19	4255.42 (3480.88, 5202.29)
Monaco	20 to 24	6950.69 (5913.07, 8170.40)

Monaco	25 to 29	9810.06 (8510.80, 11307.67)
Monaco	30 to 34	11575.18 (10093.46, 13274.41)
Monaco	35 to 39	12393.72 (10621.91, 14461.08)
Monaco	40 to 44	14095.51 (12062.00, 16471.86)
Monaco	45 to 49	16604.92 (14175.26, 19451.04)
Mongolia	15 to 19	22420.99 (22267.95, 22575.08)
Mongolia	20 to 24	26867.12 (26700.76, 27034.51)
Mongolia	25 to 29	32175.72 (31986.18, 32366.38)
Mongolia	30 to 34	35662.06 (35447.88, 35877.54)
Mongolia	35 to 39	37857.79 (37591.41, 38126.05)
Mongolia	40 to 44	40634.55 (40331.87, 40939.49)
Mongolia	45 to 49	43276.23 (42928.17, 43627.11)
Montenegro	15 to 19	5532.78 (5366.20, 5704.54)
Montenegro	20 to 24	9448.03 (9210.16, 9692.04)
Montenegro	25 to 29	13839.44 (13520.21, 14166.20)
Montenegro	30 to 34	16769.51 (16388.74, 17159.12)
Montenegro	35 to 39	18623.01 (18142.44, 19116.30)
Montenegro	40 to 44	20840.03 (20289.58, 21405.41)
Montenegro	45 to 49	23391.60 (22752.28, 24048.88)
Morocco	15 to 19	11083.54 (11033.95, 11133.35)
Morocco	20 to 24	19374.34 (19303.06, 19445.89)
Morocco	25 to 29	28429.85 (28333.98, 28526.04)
Morocco	30 to 34	35858.54 (35739.58, 35977.90)
Morocco	35 to 39	40810.67 (40650.98, 40970.99)

Morocco	40 to 44	47582.50 (47388.99, 47776.80)
Morocco	45 to 49	54872.51 (54637.62, 55108.41)
Mozambique	15 to 19	15883.51 (15812.74, 15954.61)
Mozambique	20 to 24	19284.67 (19206.89, 19362.76)
Mozambique	25 to 29	23190.50 (23099.47, 23281.90)
Mozambique	30 to 34	25387.76 (25284.41, 25491.53)
Mozambique	35 to 39	26797.68 (26666.94, 26929.06)
Mozambique	40 to 44	28693.62 (28542.60, 28845.44)
Mozambique	45 to 49	30618.63 (30441.00, 30797.30)
Myanmar	15 to 19	9260.45 (9178.22, 9343.42)
Myanmar	20 to 24	14174.93 (14067.10, 14283.59)
Myanmar	25 to 29	20279.23 (20136.75, 20422.71)
Myanmar	30 to 34	23947.48 (23779.93, 24116.20)
Myanmar	35 to 39	26023.81 (25813.04, 26236.30)
Myanmar	40 to 44	29484.82 (29237.54, 29734.19)
Myanmar	45 to 49	33546.71 (33251.35, 33844.69)
Namibia	15 to 19	10736.59 (10620.13, 10854.32)
Namibia	20 to 24	14972.74 (14827.73, 15119.16)
Namibia	25 to 29	18863.41 (18687.88, 19040.59)
Namibia	30 to 34	21090.26 (20890.73, 21291.70)
Namibia	35 to 39	22393.29 (22140.32, 22649.16)
Namibia	40 to 44	24084.95 (23794.24, 24379.21)
Namibia	45 to 49	25873.94 (25533.95, 26218.46)
Nauru	15 to 19	7815.96 (6547.99, 9329.46)

Nauru	20 to 24	11850.49 (10154.70, 13829.46)
Nauru	25 to 29	16615.45 (14384.22, 19192.77)
Nauru	30 to 34	19618.35 (17002.39, 22636.81)
Nauru	35 to 39	21334.65 (18130.03, 25105.71)
Nauru	40 to 44	24058.33 (20319.48, 28485.13)
Nauru	45 to 49	27097.11 (22638.37, 32434.01)
Nepal	15 to 19	5659.21 (5602.13, 5716.87)
Nepal	20 to 24	9797.64 (9715.69, 9880.28)
Nepal	25 to 29	13947.87 (13840.25, 14056.33)
Nepal	30 to 34	16427.15 (16299.91, 16555.37)
Nepal	35 to 39	17888.40 (17722.15, 18056.21)
Nepal	40 to 44	20251.67 (20053.83, 20451.47)
Nepal	45 to 49	22667.31 (22430.87, 22906.25)
Netherlands	15 to 19	3475.79 (3435.95, 3516.09)
Netherlands	20 to 24	6108.65 (6053.16, 6164.64)
Netherlands	25 to 29	8868.51 (8796.20, 8941.42)
Netherlands	30 to 34	10376.60 (10293.01, 10460.88)
Netherlands	35 to 39	10940.43 (10840.03, 11041.75)
Netherlands	40 to 44	12076.19 (11963.17, 12190.29)
Netherlands	45 to 49	13718.01 (13586.36, 13850.93)
New Zealand	15 to 19	4755.39 (4577.84, 4939.83)
New Zealand	20 to 24	7121.94 (6897.54, 7353.64)
New Zealand	25 to 29	9648.04 (9370.17, 9934.16)
New Zealand	30 to 34	10964.78 (10659.35, 11278.96)

New Zealand	35 to 39	11518.40 (11147.83, 11901.28)
New Zealand	40 to 44	12492.18 (12080.65, 12917.74)
New Zealand	45 to 49	13977.70 (13501.86, 14470.30)
Nicaragua	15 to 19	8668.60 (8547.15, 8791.78)
Nicaragua	20 to 24	13917.98 (13751.51, 14086.46)
Nicaragua	25 to 29	19873.15 (19652.98, 20095.78)
Nicaragua	30 to 34	23903.15 (23639.16, 24170.08)
Nicaragua	35 to 39	26395.86 (26046.63, 26749.77)
Nicaragua	40 to 44	30070.24 (29651.99, 30494.39)
Nicaragua	45 to 49	33416.72 (32918.46, 33922.52)
Niger	15 to 19	23202.75 (23112.39, 23293.46)
Niger	20 to 24	27719.19 (27619.71, 27819.02)
Niger	25 to 29	32412.17 (32297.74, 32527.01)
Niger	30 to 34	35151.93 (35023.31, 35281.03)
Niger	35 to 39	36577.91 (36416.35, 36740.18)
Niger	40 to 44	38798.44 (38613.26, 38984.50)
Niger	45 to 49	40614.20 (40399.17, 40830.37)
Nigeria	15 to 19	21127.28 (21028.89, 21226.13)
Nigeria	20 to 24	26028.56 (25918.84, 26138.74)
Nigeria	25 to 29	30932.79 (30806.41, 31059.68)
Nigeria	30 to 34	33071.50 (32932.18, 33211.40)
Nigeria	35 to 39	35003.67 (34826.51, 35181.73)
Nigeria	40 to 44	36881.37 (36679.49, 37084.37)
Nigeria	45 to 49	37958.05 (37728.86, 38188.64)

Niue	15 to 19	7434.08 (4556.41, 12129.21)
Niue	20 to 24	12226.62 (7843.40, 19059.36)
Niue	25 to 29	17862.91 (11855.23, 26915.01)
Niue	30 to 34	21340.57 (14296.55, 31855.24)
Niue	35 to 39	23134.92 (14810.68, 36137.75)
Niue	40 to 44	26861.72 (17107.38, 42177.83)
Niue	45 to 49	31388.03 (19751.85, 49879.31)
North Macedonia	15 to 19	6386.94 (6290.79, 6484.55)
North Macedonia	20 to 24	11516.88 (11377.54, 11657.92)
North Macedonia	25 to 29	17400.50 (17212.50, 17590.56)
North Macedonia	30 to 34	21018.87 (20796.42, 21243.70)
North Macedonia	35 to 39	23548.32 (23260.27, 23839.94)
North Macedonia	40 to 44	27017.29 (26679.05, 27359.81)
North Macedonia	45 to 49	30138.06 (29746.89, 30534.38)
Northern Mariana Islands	15 to 19	9879.08 (9100.42, 10724.37)
Northern Mariana Islands	20 to 24	16000.06 (14935.27, 17140.75)
Northern Mariana Islands	25 to 29	23190.37 (21754.23, 24721.32)
Northern Mariana Islands	30 to 34	27614.11 (25914.67, 29424.99)
Northern Mariana Islands	35 to 39	30610.68 (28580.22, 32785.40)
Northern Mariana Islands	40 to 44	34816.16 (32466.68, 37335.67)
Northern Mariana Islands	45 to 49	39673.01 (36887.54, 42668.82)
Norway	15 to 19	3538.41 (3482.98, 3594.73)
Norway	20 to 24	5125.65 (5058.21, 5194.00)
Norway	25 to 29	7193.97 (7109.40, 7279.55)

Norway	30 to 34	8447.30 (8350.51, 8545.21)
Norway	35 to 39	9097.80 (8976.99, 9220.24)
Norway	40 to 44	10324.57 (10184.73, 10466.34)
Norway	45 to 49	12023.53 (11856.18, 12193.24)
Oman	15 to 19	11729.51 (11403.57, 12064.76)
Oman	20 to 24	20821.64 (20354.63, 21299.36)
Oman	25 to 29	30665.99 (30065.03, 31278.96)
Oman	30 to 34	38998.50 (38273.21, 39737.54)
Oman	35 to 39	44040.88 (42994.03, 45113.22)
Oman	40 to 44	51012.85 (49693.73, 52366.98)
Oman	45 to 49	58558.40 (56877.83, 60288.63)
Pakistan	15 to 19	10851.60 (10765.27, 10938.62)
Pakistan	20 to 24	15885.56 (15776.96, 15994.91)
Pakistan	25 to 29	20837.37 (20704.33, 20971.27)
Pakistan	30 to 34	23842.49 (23687.89, 23998.10)
Pakistan	35 to 39	25941.64 (25736.49, 26148.42)
Pakistan	40 to 44	28943.12 (28700.17, 29188.13)
Pakistan	45 to 49	31761.05 (31471.95, 32052.81)
Palau	15 to 19	7875.99 (6778.24, 9151.54)
Palau	20 to 24	12650.53 (11141.41, 14364.05)
Palau	25 to 29	19064.48 (16947.46, 21445.95)
Palau	30 to 34	23182.42 (20621.33, 26061.57)
Palau	35 to 39	25696.84 (22626.70, 29183.56)
Palau	40 to 44	29884.06 (26267.46, 33998.60)

Palau	45 to 49	34630.78 (30336.53, 39532.89)
Palestine	15 to 19	12426.01 (12256.91, 12597.44)
Palestine	20 to 24	21528.65 (21287.33, 21772.70)
Palestine	25 to 29	30729.43 (30410.87, 31051.33)
Palestine	30 to 34	37740.36 (37347.30, 38137.56)
Palestine	35 to 39	42145.11 (41608.59, 42688.56)
Palestine	40 to 44	48115.48 (47464.35, 48775.53)
Palestine	45 to 49	54456.38 (53662.60, 55261.90)
Panama	15 to 19	7849.10 (7766.87, 7932.20)
Panama	20 to 24	12719.48 (12608.44, 12831.51)
Panama	25 to 29	18514.92 (18367.96, 18663.05)
Panama	30 to 34	22494.40 (22319.50, 22670.66)
Panama	35 to 39	25070.80 (24842.25, 25301.46)
Panama	40 to 44	28802.63 (28530.90, 29076.95)
Panama	45 to 49	32387.74 (32067.30, 32711.38)
Papua New Guinea	15 to 19	17375.24 (17021.54, 17736.30)
Papua New Guinea	20 to 24	21335.18 (20947.01, 21730.55)
Papua New Guinea	25 to 29	26067.92 (25623.85, 26519.69)
Papua New Guinea	30 to 34	28951.24 (28458.67, 29452.33)
Papua New Guinea	35 to 39	30894.03 (30262.23, 31539.02)
Papua New Guinea	40 to 44	33891.70 (33154.99, 34644.77)
Papua New Guinea	45 to 49	37422.73 (36538.78, 38328.07)
Paraguay	15 to 19	6690.16 (6636.53, 6744.22)
Paraguay	20 to 24	11323.27 (11247.41, 11399.63)

Paraguay	25 to 29	16168.31 (16068.78, 16268.45)
Paraguay	30 to 34	19407.25 (19288.66, 19526.58)
Paraguay	35 to 39	22102.59 (21940.84, 22265.52)
Paraguay	40 to 44	25349.62 (25155.32, 25545.41)
Paraguay	45 to 49	29269.70 (29033.04, 29508.28)
Peru	15 to 19	6566.31 (6503.84, 6629.38)
Peru	20 to 24	10065.81 (9984.09, 10148.21)
Peru	25 to 29	14462.49 (14355.25, 14570.53)
Peru	30 to 34	17446.61 (17319.54, 17574.62)
Peru	35 to 39	19678.22 (19508.63, 19849.29)
Peru	40 to 44	22627.11 (22424.22, 22831.83)
Peru	45 to 49	25392.60 (25151.61, 25635.89)
Philippines	15 to 19	16222.93 (15908.80, 16543.26)
Philippines	20 to 24	20976.78 (20607.77, 21352.39)
Philippines	25 to 29	25906.95 (25473.44, 26347.84)
Philippines	30 to 34	28644.69 (28163.84, 29133.75)
Philippines	35 to 39	30375.90 (29778.21, 30985.58)
Philippines	40 to 44	33120.03 (32435.53, 33818.97)
Philippines	45 to 49	35958.05 (35164.87, 36769.12)
Poland	15 to 19	5918.80 (5877.87, 5960.02)
Poland	20 to 24	8603.08 (8552.03, 8654.42)
Poland	25 to 29	12752.55 (12684.43, 12821.04)
Poland	30 to 34	15069.30 (14989.89, 15149.13)
Poland	35 to 39	16455.74 (16356.39, 16555.69)

Poland	40 to 44	18410.21 (18296.14, 18525.00)
Poland	45 to 49	20601.39 (20468.89, 20734.75)
Portugal	15 to 19	5881.19 (5819.41, 5943.63)
Portugal	20 to 24	9067.32 (8986.32, 9149.05)
Portugal	25 to 29	12657.85 (12553.39, 12763.18)
Portugal	30 to 34	14595.35 (14476.31, 14715.38)
Portugal	35 to 39	15676.17 (15532.95, 15820.72)
Portugal	40 to 44	18331.68 (18161.62, 18503.34)
Portugal	45 to 49	22468.61 (22254.86, 22684.41)
Puerto Rico	15 to 19	6762.65 (6688.93, 6837.18)
Puerto Rico	20 to 24	12927.19 (12812.05, 13043.37)
Puerto Rico	25 to 29	19455.05 (19294.65, 19616.77)
Puerto Rico	30 to 34	23953.05 (23756.04, 24151.70)
Puerto Rico	35 to 39	26693.65 (26449.67, 26939.89)
Puerto Rico	40 to 44	30603.17 (30318.34, 30890.68)
Puerto Rico	45 to 49	35240.22 (34902.63, 35581.08)
Qatar	15 to 19	12677.54 (12442.26, 12917.28)
Qatar	20 to 24	23182.19 (22887.55, 23480.61)
Qatar	25 to 29	34959.81 (34650.42, 35271.97)
Qatar	30 to 34	44209.77 (43848.42, 44574.09)
Qatar	35 to 39	50221.24 (49657.70, 50791.17)
Qatar	40 to 44	57748.55 (57027.78, 58478.43)
Qatar	45 to 49	65625.37 (64715.74, 66547.78)
Republic of Korea	15 to 19	5281.17 (4991.34, 5587.81)

Republic of Korea	20 to 24	7228.96 (6880.28, 7595.31)
Republic of Korea	25 to 29	9268.07 (8851.49, 9704.26)
Republic of Korea	30 to 34	10216.31 (9758.27, 10695.85)
Republic of Korea	35 to 39	10106.31 (9610.20, 10628.04)
Republic of Korea	40 to 44	10880.36 (10337.87, 11451.32)
Republic of Korea	45 to 49	12330.76 (11700.10, 12995.42)
Republic of Moldova	15 to 19	13591.37 (13334.68, 13853.00)
Republic of Moldova	20 to 24	17022.70 (16727.83, 17322.76)
Republic of Moldova	25 to 29	20545.15 (20209.55, 20886.33)
Republic of Moldova	30 to 34	22772.04 (22401.19, 23149.02)
Republic of Moldova	35 to 39	24056.71 (23610.65, 24511.20)
Republic of Moldova	40 to 44	26112.83 (25611.15, 26624.34)
Republic of Moldova	45 to 49	27925.64 (27360.60, 28502.35)
Romania	15 to 19	9226.73 (8805.27, 9668.37)
Romania	20 to 24	12990.51 (12460.25, 13543.34)
Romania	25 to 29	17182.47 (16516.96, 17874.79)
Romania	30 to 34	19267.16 (18525.36, 20038.67)
Romania	35 to 39	20666.45 (19780.92, 21591.62)
Romania	40 to 44	22504.22 (21521.06, 23532.30)
Romania	45 to 49	25151.78 (24008.06, 26349.98)
Russian Federation	15 to 19	8286.73 (8202.47, 8371.86)
Russian Federation	20 to 24	11051.24 (10952.38, 11150.99)
Russian Federation	25 to 29	14676.08 (14555.09, 14798.08)
Russian Federation	30 to 34	16722.17 (16585.47, 16859.99)

Russian Federation	35 to 39	18382.27 (18210.76, 18555.40)
Russian Federation	40 to 44	21136.45 (20934.45, 21340.41)
Russian Federation	45 to 49	23359.16 (23126.79, 23593.88)
Rwanda	15 to 19	11489.94 (11422.01, 11558.28)
Rwanda	20 to 24	14421.38 (14343.13, 14500.05)
Rwanda	25 to 29	17422.39 (17330.76, 17514.49)
Rwanda	30 to 34	18990.66 (18888.55, 19093.33)
Rwanda	35 to 39	20433.95 (20301.32, 20567.45)
Rwanda	40 to 44	21923.42 (21769.82, 22078.10)
Rwanda	45 to 49	23724.86 (23543.30, 23907.82)
Saint Kitts and Nevis	15 to 19	6985.26 (6400.27, 7623.73)
Saint Kitts and Nevis	20 to 24	12288.39 (11436.96, 13203.19)
Saint Kitts and Nevis	25 to 29	17653.69 (16542.96, 18839.00)
Saint Kitts and Nevis	30 to 34	21255.98 (19949.22, 22648.35)
Saint Kitts and Nevis	35 to 39	23916.42 (22218.21, 25744.43)
Saint Kitts and Nevis	40 to 44	27326.39 (25335.95, 29473.21)
Saint Kitts and Nevis	45 to 49	30737.55 (28413.95, 33251.16)
Saint Lucia	15 to 19	5837.54 (5538.79, 6152.41)
Saint Lucia	20 to 24	9898.43 (9468.42, 10347.97)
Saint Lucia	25 to 29	14009.59 (13442.98, 14600.10)
Saint Lucia	30 to 34	17025.41 (16344.02, 17735.21)
Saint Lucia	35 to 39	19152.92 (18281.63, 20065.73)
Saint Lucia	40 to 44	21623.63 (20614.65, 22682.01)
Saint Lucia	45 to 49	24389.44 (23208.53, 25630.44)

Saint Vincent and the Grenadines	15 to 19	6473.97 (6088.68, 6883.63)
Saint Vincent and the Grenadines	20 to 24	11323.49 (10746.42, 11931.55)
Saint Vincent and the Grenadines	25 to 29	16357.95 (15577.70, 17177.29)
Saint Vincent and the Grenadines	30 to 34	19891.66 (18949.88, 20880.25)
Saint Vincent and the Grenadines	35 to 39	22401.13 (21202.14, 23667.91)
Saint Vincent and the Grenadines	40 to 44	25725.85 (24312.48, 27221.39)
Saint Vincent and the Grenadines	45 to 49	29051.09 (27382.74, 30821.09)
Samoa	15 to 19	10693.70 (10290.05, 11113.18)
Samoa	20 to 24	15580.88 (15045.37, 16135.45)
Samoa	25 to 29	21601.80 (20904.33, 22322.56)
Samoa	30 to 34	25438.38 (24624.51, 26279.16)
Samoa	35 to 39	27827.08 (26792.49, 28901.61)
Samoa	40 to 44	31347.38 (30134.39, 32609.20)
Samoa	45 to 49	35529.97 (34080.47, 37041.12)
San Marino	15 to 19	4328.58 (3577.51, 5237.34)
San Marino	20 to 24	6967.84 (5974.75, 8126.00)
San Marino	25 to 29	9809.67 (8538.10, 11270.63)
San Marino	30 to 34	11413.19 (9965.13, 13071.67)
San Marino	35 to 39	12157.61 (10445.27, 14150.66)
San Marino	40 to 44	13703.49 (11748.37, 15983.99)
San Marino	45 to 49	15958.89 (13632.23, 18682.66)
Sao Tome and Principe	15 to 19	16014.09 (15505.38, 16539.48)
Sao Tome and Principe	20 to 24	20948.23 (20334.68, 21580.29)
Sao Tome and Principe	25 to 29	25923.05 (25191.71, 26675.62)

Sao Tome and Principe	30 to 34	28813.49 (27988.84, 29662.44)
Sao Tome and Principe	35 to 39	30155.36 (29114.62, 31233.31)
Sao Tome and Principe	40 to 44	32004.91 (30819.16, 33236.28)
Sao Tome and Principe	45 to 49	33480.83 (32122.13, 34896.99)
Saudi Arabia	15 to 19	14090.21 (13632.81, 14562.96)
Saudi Arabia	20 to 24	23220.93 (22613.00, 23845.21)
Saudi Arabia	25 to 29	32875.43 (32117.32, 33651.44)
Saudi Arabia	30 to 34	40360.87 (39473.48, 41268.21)
Saudi Arabia	35 to 39	45331.31 (44093.73, 46603.63)
Saudi Arabia	40 to 44	52345.05 (50812.57, 53923.75)
Saudi Arabia	45 to 49	60198.84 (58280.21, 62180.63)
Senegal	15 to 19	15584.15 (15514.81, 15653.80)
Senegal	20 to 24	22003.99 (21917.72, 22090.59)
Senegal	25 to 29	28127.42 (28022.16, 28233.07)
Senegal	30 to 34	32255.23 (32132.10, 32378.83)
Senegal	35 to 39	34628.78 (34469.05, 34789.24)
Senegal	40 to 44	37921.79 (37734.25, 38110.26)
Senegal	45 to 49	40431.39 (40212.87, 40651.10)
Serbia	15 to 19	7764.18 (7700.93, 7827.94)
Serbia	20 to 24	13407.79 (13317.92, 13498.26)
Serbia	25 to 29	19934.08 (19812.37, 20056.54)
Serbia	30 to 34	24189.43 (24044.62, 24335.11)
Serbia	35 to 39	27161.19 (26976.86, 27346.77)
Serbia	40 to 44	30460.12 (30249.08, 30672.64)

Serbia	45 to 49	33961.32 (33716.85, 34207.57)
Seychelles	15 to 19	9521.11 (8962.88, 10114.11)
Seychelles	20 to 24	15655.29 (14887.64, 16462.52)
Seychelles	25 to 29	22838.31 (21821.43, 23902.58)
Seychelles	30 to 34	26413.15 (25254.68, 27624.76)
Seychelles	35 to 39	28625.57 (27196.44, 30129.79)
Seychelles	40 to 44	32165.22 (30517.29, 33902.15)
Seychelles	45 to 49	36256.93 (34323.69, 38299.06)
Sierra Leone	15 to 19	17624.96 (17529.68, 17720.76)
Sierra Leone	20 to 24	21647.04 (21542.25, 21752.35)
Sierra Leone	25 to 29	25782.44 (25663.22, 25902.23)
Sierra Leone	30 to 34	28189.54 (28056.45, 28323.26)
Sierra Leone	35 to 39	29390.11 (29220.93, 29560.27)
Sierra Leone	40 to 44	31183.47 (30988.13, 31380.04)
Sierra Leone	45 to 49	32625.27 (32395.85, 32856.31)
Singapore	15 to 19	6063.70 (5860.49, 6273.95)
Singapore	20 to 24	9329.49 (9089.39, 9575.94)
Singapore	25 to 29	12128.65 (11849.57, 12414.31)
Singapore	30 to 34	13359.51 (13054.55, 13671.61)
Singapore	35 to 39	13336.78 (12984.37, 13698.76)
Singapore	40 to 44	14393.66 (14000.05, 14798.33)
Singapore	45 to 49	16015.00 (15555.81, 16487.76)
Slovakia	15 to 19	5359.66 (5283.40, 5437.03)
Slovakia	20 to 24	8053.56 (7956.05, 8152.27)

Slovakia	25 to 29	11479.04 (11352.49, 11606.99)
Slovakia	30 to 34	13692.37 (13544.27, 13842.09)
Slovakia	35 to 39	14834.16 (14650.24, 15020.40)
Slovakia	40 to 44	16889.94 (16675.80, 17106.83)
Slovakia	45 to 49	18618.64 (18372.67, 18867.91)
Slovenia	15 to 19	4993.96 (4896.87, 5092.97)
Slovenia	20 to 24	8584.86 (8450.16, 8721.70)
Slovenia	25 to 29	12632.44 (12454.73, 12812.69)
Slovenia	30 to 34	14991.26 (14784.29, 15201.12)
Slovenia	35 to 39	16616.79 (16358.54, 16879.11)
Slovenia	40 to 44	18666.95 (18371.52, 18967.14)
Slovenia	45 to 49	20968.12 (20626.52, 21315.38)
Solomon Islands	15 to 19	12526.39 (11915.11, 13169.04)
Solomon Islands	20 to 24	17145.23 (16399.40, 17924.99)
Solomon Islands	25 to 29	22397.01 (21479.76, 23353.43)
Solomon Islands	30 to 34	25848.53 (24806.68, 26934.14)
Solomon Islands	35 to 39	27479.17 (26173.47, 28850.00)
Solomon Islands	40 to 44	30599.02 (29061.01, 32218.43)
Solomon Islands	45 to 49	34021.35 (32174.31, 35974.44)
Somalia	15 to 19	20573.78 (20496.21, 20651.65)
Somalia	20 to 24	25896.70 (25807.56, 25986.15)
Somalia	25 to 29	31397.93 (31292.51, 31503.70)
Somalia	30 to 34	34728.58 (34609.76, 34847.81)
Somalia	35 to 39	36727.72 (36575.01, 36881.06)

Somalia	40 to 44	39510.66 (39332.70, 39689.43)
Somalia	45 to 49	41808.45 (41597.80, 42020.17)
South Africa	15 to 19	7375.68 (7207.78, 7547.49)
South Africa	20 to 24	12653.89 (12417.28, 12895.02)
South Africa	25 to 29	17737.75 (17433.84, 18046.96)
South Africa	30 to 34	20825.28 (20470.39, 21186.32)
South Africa	35 to 39	23089.42 (22622.60, 23565.88)
South Africa	40 to 44	25922.40 (25372.82, 26483.89)
South Africa	45 to 49	28313.96 (27675.15, 28967.51)
South Sudan	15 to 19	14248.80 (14155.17, 14343.04)
South Sudan	20 to 24	19407.19 (19293.47, 19521.58)
South Sudan	25 to 29	24873.05 (24733.30, 25013.59)
South Sudan	30 to 34	28341.45 (28175.94, 28507.92)
South Sudan	35 to 39	30587.35 (30376.54, 30799.62)
South Sudan	40 to 44	33669.50 (33419.67, 33921.20)
South Sudan	45 to 49	36496.99 (36196.73, 36799.75)
Spain	15 to 19	5111.52 (5007.49, 5217.71)
Spain	20 to 24	7740.23 (7610.78, 7871.87)
Spain	25 to 29	11164.48 (10996.82, 11334.69)
Spain	30 to 34	13206.71 (13011.93, 13404.40)
Spain	35 to 39	14076.26 (13842.82, 14313.65)
Spain	40 to 44	15546.27 (15282.31, 15814.79)
Spain	45 to 49	18684.97 (18358.89, 19016.84)
Sri Lanka	15 to 19	7840.52 (7764.68, 7917.10)

Sri Lanka	20 to 24	13637.17 (13528.59, 13746.62)
Sri Lanka	25 to 29	20640.89 (20491.82, 20791.03)
Sri Lanka	30 to 34	24882.97 (24706.21, 25060.99)
Sri Lanka	35 to 39	27230.34 (27008.15, 27454.36)
Sri Lanka	40 to 44	30925.38 (30666.30, 31186.65)
Sri Lanka	45 to 49	34436.86 (34136.73, 34739.64)
Sudan	15 to 19	15297.90 (15244.73, 15351.25)
Sudan	20 to 24	20649.02 (20585.09, 20713.15)
Sudan	25 to 29	26382.89 (26305.48, 26460.53)
Sudan	30 to 34	31125.69 (31034.89, 31216.74)
Sudan	35 to 39	34149.06 (34028.19, 34270.37)
Sudan	40 to 44	38460.65 (38314.65, 38607.20)
Sudan	45 to 49	43241.69 (43061.42, 43422.71)
Suriname	15 to 19	6649.41 (6460.73, 6843.60)
Suriname	20 to 24	11365.68 (11101.38, 11636.26)
Suriname	25 to 29	16452.28 (16104.14, 16807.94)
Suriname	30 to 34	20269.77 (19845.04, 20703.60)
Suriname	35 to 39	23042.12 (22480.96, 23617.29)
Suriname	40 to 44	26769.22 (26097.76, 27457.96)
Suriname	45 to 49	30540.08 (29744.94, 31356.47)
Sweden	15 to 19	3621.85 (3581.87, 3662.28)
Sweden	20 to 24	5607.51 (5556.62, 5658.87)
Sweden	25 to 29	8040.61 (7975.61, 8106.15)
Sweden	30 to 34	9473.13 (9398.02, 9548.85)

Sweden	35 to 39	10032.09 (9939.28, 10125.76)
Sweden	40 to 44	11281.35 (11174.65, 11389.07)
Sweden	45 to 49	12799.41 (12674.59, 12925.46)
Switzerland	15 to 19	4218.97 (4170.34, 4268.17)
Switzerland	20 to 24	6212.02 (6153.29, 6271.31)
Switzerland	25 to 29	8610.12 (8538.19, 8682.66)
Switzerland	30 to 34	10132.34 (10050.26, 10215.08)
Switzerland	35 to 39	10864.16 (10762.31, 10966.98)
Switzerland	40 to 44	12135.30 (12018.98, 12252.74)
Switzerland	45 to 49	13998.30 (13860.53, 14137.45)
Syrian Arab Republic	15 to 19	12814.00 (12653.80, 12976.23)
Syrian Arab Republic	20 to 24	21782.59 (21554.05, 22013.55)
Syrian Arab Republic	25 to 29	31066.33 (30742.66, 31393.40)
Syrian Arab Republic	30 to 34	38629.25 (38212.53, 39050.53)
Syrian Arab Republic	35 to 39	43654.85 (43125.94, 44190.23)
Syrian Arab Republic	40 to 44	50303.72 (49661.29, 50954.46)
Syrian Arab Republic	45 to 49	57310.91 (56532.67, 58099.87)
Taiwan (Province of China)	15 to 19	11382.30 (10648.08, 12167.15)
Taiwan (Province of China)	20 to 24	15871.01 (14966.88, 16829.77)
Taiwan (Province of China)	25 to 29	21288.93 (20170.37, 22469.52)
Taiwan (Province of China)	30 to 34	23904.27 (22672.34, 25203.14)
Taiwan (Province of China)	35 to 39	25460.68 (24024.59, 26982.61)
Taiwan (Province of China)	40 to 44	28190.24 (26571.78, 29907.28)
Taiwan (Province of China)	45 to 49	31439.92 (29581.21, 33415.42)

Tajikistan	15 to 19	15911.79 (15824.33, 15999.73)
Tajikistan	20 to 24	20782.00 (20679.94, 20884.55)
Tajikistan	25 to 29	26089.35 (25968.37, 26210.89)
Tajikistan	30 to 34	29484.64 (29345.99, 29623.94)
Tajikistan	35 to 39	31714.05 (31532.02, 31897.13)
Tajikistan	40 to 44	34838.00 (34625.11, 35052.19)
Tajikistan	45 to 49	37748.99 (37500.73, 37998.89)
Thailand	15 to 19	10330.80 (9845.00, 10840.57)
Thailand	20 to 24	15622.36 (14985.91, 16285.83)
Thailand	25 to 29	21386.05 (20580.29, 22223.37)
Thailand	30 to 34	24588.92 (23680.40, 25532.31)
Thailand	35 to 39	26700.31 (25603.67, 27843.93)
Thailand	40 to 44	30103.86 (28845.28, 31417.35)
Thailand	45 to 49	34178.79 (32706.51, 35717.34)
Timor-Leste	15 to 19	12687.53 (12495.83, 12882.18)
Timor-Leste	20 to 24	17177.70 (16947.58, 17410.94)
Timor-Leste	25 to 29	22551.32 (22264.90, 22841.43)
Timor-Leste	30 to 34	25636.76 (25302.69, 25975.24)
Timor-Leste	35 to 39	27218.92 (26797.12, 27647.37)
Timor-Leste	40 to 44	29987.25 (29502.19, 30480.29)
Timor-Leste	45 to 49	33114.85 (32541.33, 33698.48)
Togo	15 to 19	17623.15 (17530.31, 17716.47)
Togo	20 to 24	22127.36 (22023.18, 22232.02)
Togo	25 to 29	26716.91 (26596.96, 26837.39)

Togo	30 to 34	29466.67 (29332.37, 29601.58)
Togo	35 to 39	31077.88 (30905.82, 31250.90)
Togo	40 to 44	33410.05 (33210.73, 33610.56)
Togo	45 to 49	35455.56 (35222.50, 35690.16)
Tokelau	15 to 19	10124.27 (6126.92, 16729.57)
Tokelau	20 to 24	15889.41 (10033.85, 25162.16)
Tokelau	25 to 29	21419.98 (13895.53, 33018.91)
Tokelau	30 to 34	26093.71 (16955.09, 40157.96)
Tokelau	35 to 39	29155.04 (18009.20, 47199.02)
Tokelau	40 to 44	33237.34 (20230.24, 54607.40)
Tokelau	45 to 49	37366.30 (22393.38, 62350.58)
Tonga	15 to 19	8916.24 (8389.18, 9476.40)
Tonga	20 to 24	13874.88 (13137.19, 14654.00)
Tonga	25 to 29	19531.08 (18546.79, 20567.61)
Tonga	30 to 34	22627.48 (21484.35, 23831.43)
Tonga	35 to 39	24592.91 (23195.96, 26073.99)
Tonga	40 to 44	27993.27 (26346.90, 29742.51)
Tonga	45 to 49	31707.95 (29745.70, 33799.63)
Trinidad and Tobago	15 to 19	6095.17 (5986.86, 6205.43)
Trinidad and Tobago	20 to 24	11143.40 (10982.08, 11307.08)
Trinidad and Tobago	25 to 29	16174.54 (15960.85, 16391.10)
Trinidad and Tobago	30 to 34	19599.06 (19343.53, 19857.97)
Trinidad and Tobago	35 to 39	21372.77 (21049.46, 21701.04)
Trinidad and Tobago	40 to 44	24734.37 (24348.10, 25126.78)

Trinidad and Tobago	45 to 49	27332.77 (26886.15, 27786.81)
Tunisia	15 to 19	14398.30 (13923.66, 14889.12)
Tunisia	20 to 24	22728.72 (22092.78, 23382.96)
Tunisia	25 to 29	31227.88 (30418.04, 32059.28)
Tunisia	30 to 34	37650.62 (36692.13, 38634.14)
Tunisia	35 to 39	42384.64 (41131.65, 43675.79)
Tunisia	40 to 44	48769.41 (47268.22, 50318.27)
Tunisia	45 to 49	55993.57 (54175.24, 57872.93)
Turkey	15 to 19	11360.93 (11213.71, 11510.09)
Turkey	20 to 24	19083.70 (18877.20, 19292.46)
Turkey	25 to 29	27442.56 (27170.38, 27717.47)
Turkey	30 to 34	33658.95 (33334.20, 33986.87)
Turkey	35 to 39	38392.55 (37961.43, 38828.57)
Turkey	40 to 44	43884.46 (43373.47, 44401.46)
Turkey	45 to 49	49301.22 (48700.94, 49908.90)
Turkmenistan	15 to 19	15223.40 (15124.32, 15323.13)
Turkmenistan	20 to 24	20926.17 (20803.90, 21049.15)
Turkmenistan	25 to 29	27162.07 (27011.57, 27313.41)
Turkmenistan	30 to 34	30909.25 (30736.09, 31083.38)
Turkmenistan	35 to 39	33461.18 (33242.83, 33680.97)
Turkmenistan	40 to 44	36843.06 (36591.68, 37096.17)
Turkmenistan	45 to 49	39715.21 (39426.97, 40005.55)
Tuvalu	15 to 19	12392.62 (10543.51, 14566.02)
Tuvalu	20 to 24	17100.88 (14816.67, 19737.23)

Tuvalu	25 to 29	23044.77 (20174.83, 26322.96)
Tuvalu	30 to 34	26939.29 (23604.39, 30745.36)
Tuvalu	35 to 39	29567.03 (25370.30, 34457.98)
Tuvalu	40 to 44	33806.10 (28875.84, 39578.16)
Tuvalu	45 to 49	38275.73 (32459.82, 45133.69)
Uganda	15 to 19	12987.50 (12884.82, 13091.00)
Uganda	20 to 24	16105.43 (15988.59, 16223.13)
Uganda	25 to 29	19218.81 (19081.94, 19356.66)
Uganda	30 to 34	21239.98 (21083.33, 21397.78)
Uganda	35 to 39	22415.95 (22213.81, 22619.93)
Uganda	40 to 44	24484.92 (24245.90, 24726.30)
Uganda	45 to 49	26091.41 (25808.62, 26377.29)
Ukraine	15 to 19	7152.77 (7102.74, 7203.16)
Ukraine	20 to 24	10320.39 (10258.48, 10382.66)
Ukraine	25 to 29	14090.88 (14013.08, 14169.11)
Ukraine	30 to 34	16733.89 (16642.37, 16825.91)
Ukraine	35 to 39	18761.39 (18644.92, 18878.58)
Ukraine	40 to 44	21475.73 (21339.15, 21613.19)
Ukraine	45 to 49	23956.00 (23798.08, 24114.96)
United Arab Emirates	15 to 19	11296.71 (11192.72, 11401.67)
United Arab Emirates	20 to 24	20024.15 (19898.74, 20150.35)
United Arab Emirates	25 to 29	29708.06 (29560.16, 29856.70)
United Arab Emirates	30 to 34	38457.31 (38270.08, 38645.46)
United Arab Emirates	35 to 39	43900.48 (43638.04, 44164.50)

United Arab Emirates	40 to 44	50134.52 (49797.59, 50473.73)
United Arab Emirates	45 to 49	57607.63 (57172.55, 58046.02)
United Kingdom	15 to 19	4225.86 (4153.06, 4299.94)
United Kingdom	20 to 24	6973.65 (6877.06, 7071.60)
United Kingdom	25 to 29	9628.94 (9509.31, 9750.09)
United Kingdom	30 to 34	11198.20 (11061.80, 11336.28)
United Kingdom	35 to 39	11861.46 (11692.98, 12032.36)
United Kingdom	40 to 44	13220.03 (13027.12, 13415.81)
United Kingdom	45 to 49	15453.75 (15222.08, 15688.94)
United Republic of Tanzania	15 to 19	13418.54 (13329.68, 13507.99)
United Republic of Tanzania	20 to 24	18074.65 (17968.12, 18181.81)
United Republic of Tanzania	25 to 29	22576.90 (22449.28, 22705.24)
United Republic of Tanzania	30 to 34	25114.04 (24968.35, 25260.59)
United Republic of Tanzania	35 to 39	26689.96 (26502.33, 26878.91)
United Republic of Tanzania	40 to 44	29039.60 (28820.14, 29260.73)
United Republic of Tanzania	45 to 49	31112.43 (30853.75, 31373.27)
United States of America	15 to 19	4338.26 (4259.83, 4418.13)
United States of America	20 to 24	7125.52 (7020.02, 7232.61)
United States of America	25 to 29	10004.84 (9870.04, 10141.48)
United States of America	30 to 34	11058.02 (10908.93, 11209.16)
United States of America	35 to 39	11714.96 (11532.64, 11900.17)
United States of America	40 to 44	13325.30 (13112.55, 13541.49)
United States of America	45 to 49	15716.39 (15458.67, 15978.41)
United States Virgin Islands	15 to 19	6130.97 (5704.37, 6589.47)

United States Virgin Islands	20 to 24	10482.48 (9838.01, 11169.17)
United States Virgin Islands	25 to 29	15051.56 (14187.56, 15968.17)
United States Virgin Islands	30 to 34	18579.68 (17522.59, 19700.54)
United States Virgin Islands	35 to 39	21100.13 (19786.18, 22501.33)
United States Virgin Islands	40 to 44	24761.37 (23198.21, 26429.85)
United States Virgin Islands	45 to 49	27582.73 (25793.07, 29496.56)
Uruguay	15 to 19	3038.38 (2986.54, 3091.13)
Uruguay	20 to 24	6387.94 (6302.87, 6474.16)
Uruguay	25 to 29	9349.27 (9234.92, 9465.03)
Uruguay	30 to 34	10770.83 (10639.71, 10903.56)
Uruguay	35 to 39	11931.79 (11765.53, 12100.40)
Uruguay	40 to 44	13554.64 (13360.52, 13751.58)
Uruguay	45 to 49	16498.16 (16254.94, 16745.02)
Uzbekistan	15 to 19	18318.59 (18214.75, 18423.02)
Uzbekistan	20 to 24	24234.62 (24112.06, 24357.80)
Uzbekistan	25 to 29	30177.67 (30033.37, 30322.67)
Uzbekistan	30 to 34	34023.22 (33859.38, 34187.85)
Uzbekistan	35 to 39	36532.55 (36322.88, 36743.43)
Uzbekistan	40 to 44	40328.96 (40085.17, 40574.23)
Uzbekistan	45 to 49	43481.43 (43200.03, 43764.67)
Vanuatu	15 to 19	11989.90 (11574.86, 12419.82)
Vanuatu	20 to 24	18633.70 (18086.76, 19197.18)
Vanuatu	25 to 29	25347.14 (24659.37, 26054.09)
Vanuatu	30 to 34	29510.64 (28708.99, 30334.68)

Vanuatu	35 to 39	32066.64 (31029.80, 33138.12)
Vanuatu	40 to 44	36260.16 (35025.22, 37538.64)
Vanuatu	45 to 49	40871.08 (39383.66, 42414.67)
Venezuela (Bolivarian Republic of)	15 to 19	7655.42 (7587.23, 7724.21)
Venezuela (Bolivarian Republic of)	20 to 24	12428.43 (12336.04, 12521.50)
Venezuela (Bolivarian Republic of)	25 to 29	18151.71 (18029.87, 18274.37)
Venezuela (Bolivarian Republic of)	30 to 34	21840.10 (21693.45, 21987.75)
Venezuela (Bolivarian Republic of)	35 to 39	24381.01 (24189.77, 24573.75)
Venezuela (Bolivarian Republic of)	40 to 44	27790.70 (27564.34, 28018.92)
Venezuela (Bolivarian Republic of)	45 to 49	31010.36 (30743.72, 31279.32)
Viet Nam	15 to 19	15145.87 (14980.76, 15312.79)
Viet Nam	20 to 24	19721.62 (19528.26, 19916.90)
Viet Nam	25 to 29	25372.91 (25141.48, 25606.48)
Viet Nam	30 to 34	28188.63 (27931.31, 28448.32)
Viet Nam	35 to 39	29769.81 (29453.45, 30089.57)
Viet Nam	40 to 44	32237.26 (31879.75, 32598.78)
Viet Nam	45 to 49	35348.93 (34934.97, 35767.79)
Yemen	15 to 19	17386.91 (17199.62, 17576.23)
Yemen	20 to 24	23631.52 (23403.86, 23861.39)
Yemen	25 to 29	30102.72 (29831.84, 30376.06)
Yemen	30 to 34	35275.23 (34960.52, 35592.77)
Yemen	35 to 39	38443.20 (38019.36, 38871.77)
Yemen	40 to 44	43058.54 (42543.24, 43580.09)
Yemen	45 to 49	47910.92 (47278.37, 48551.93)

Zambia	15 to 19	11529.40 (11461.08, 11598.13)
Zambia	20 to 24	15668.14 (15585.86, 15750.85)
Zambia	25 to 29	20028.88 (19928.61, 20129.65)
Zambia	30 to 34	22797.24 (22681.66, 22913.41)
Zambia	35 to 39	24537.85 (24383.88, 24692.79)
Zambia	40 to 44	26656.80 (26475.36, 26839.49)
Zambia	45 to 49	28559.00 (28342.84, 28776.81)
Zimbabwe	15 to 19	22237.05 (21840.91, 22640.37)
Zimbabwe	20 to 24	28137.26 (27670.72, 28611.66)
Zimbabwe	25 to 29	33020.06 (32480.03, 33569.07)
Zimbabwe	30 to 34	36098.11 (35485.59, 36721.21)
Zimbabwe	35 to 39	37923.90 (37152.06, 38711.78)
Zimbabwe	40 to 44	40680.73 (39778.12, 41603.82)
Zimbabwe	45 to 49	42496.94 (41440.05, 43580.78)

CLD, chronic liver disease; WCBA, women of childbearing age.

Table S9 Period effects on CLD prevalence in WCBA across countries.

Location	Period	Prevalence rate ratio
Afghanistan	1992 to 1996	0.957 (0.953, 0.961)
Afghanistan	1997 to 2001	0.968 (0.965, 0.972)
Afghanistan	2002 to 2006	0.982 (0.978, 0.985)
Afghanistan	2007 to 2011	1.000 (1.000, 1.000)
Afghanistan	2012 to 2016	1.024 (1.021, 1.028)
Afghanistan	2017 to 2021	1.049 (1.046, 1.053)
Albania	1992 to 1996	1.017 (1.002, 1.032)
Albania	1997 to 2001	1.012 (0.998, 1.026)
Albania	2002 to 2006	1.008 (0.994, 1.022)
Albania	2007 to 2011	1.000 (1.000, 1.000)
Albania	2012 to 2016	0.994 (0.980, 1.008)
Albania	2017 to 2021	1.016 (1.001, 1.031)
Algeria	1992 to 1996	0.923 (0.920, 0.926)
Algeria	1997 to 2001	0.937 (0.934, 0.940)
Algeria	2002 to 2006	0.963 (0.960, 0.966)
Algeria	2007 to 2011	1.000 (1.000, 1.000)
Algeria	2012 to 2016	1.037 (1.034, 1.040)
Algeria	2017 to 2021	1.072 (1.069, 1.075)
American Samoa	1992 to 1996	1.004 (0.953, 1.056)
American Samoa	1997 to 2001	1.015 (0.968, 1.065)
American Samoa	2002 to 2006	1.026 (0.979, 1.074)
American Samoa	2007 to 2011	1.000 (1.000, 1.000)

American Samoa	2012 to 2016	0.996 (0.949, 1.044)
American Samoa	2017 to 2021	0.997 (0.949, 1.047)
Andorra	1992 to 1996	0.972 (0.907, 1.041)
Andorra	1997 to 2001	0.979 (0.917, 1.044)
Andorra	2002 to 2006	0.985 (0.927, 1.046)
Andorra	2007 to 2011	1.000 (1.000, 1.000)
Andorra	2012 to 2016	1.019 (0.960, 1.082)
Andorra	2017 to 2021	1.053 (0.992, 1.118)
Angola	1992 to 1996	1.081 (1.076, 1.086)
Angola	1997 to 2001	1.054 (1.049, 1.059)
Angola	2002 to 2006	1.030 (1.025, 1.034)
Angola	2007 to 2011	1.000 (1.000, 1.000)
Angola	2012 to 2016	0.965 (0.961, 0.969)
Angola	2017 to 2021	0.940 (0.937, 0.944)
Antigua and Barbuda	1992 to 1996	1.018 (0.970, 1.069)
Antigua and Barbuda	1997 to 2001	1.007 (0.964, 1.053)
Antigua and Barbuda	2002 to 2006	1.002 (0.961, 1.044)
Antigua and Barbuda	2007 to 2011	1.000 (1.000, 1.000)
Antigua and Barbuda	2012 to 2016	0.996 (0.956, 1.037)
Antigua and Barbuda	2017 to 2021	0.991 (0.951, 1.033)
Argentina	1992 to 1996	0.896 (0.890, 0.903)
Argentina	1997 to 2001	0.931 (0.925, 0.937)
Argentina	2002 to 2006	0.965 (0.959, 0.971)
Argentina	2007 to 2011	1.000 (1.000, 1.000)

Argentina	2012 to 2016	1.045 (1.039, 1.051)
Argentina	2017 to 2021	1.079 (1.073, 1.085)
Armenia	1992 to 1996	0.945 (0.939, 0.951)
Armenia	1997 to 2001	0.952 (0.946, 0.958)
Armenia	2002 to 2006	0.971 (0.965, 0.977)
Armenia	2007 to 2011	1.000 (1.000, 1.000)
Armenia	2012 to 2016	1.009 (1.003, 1.015)
Armenia	2017 to 2021	1.008 (1.002, 1.015)
Australia	1992 to 1996	0.983 (0.967, 0.999)
Australia	1997 to 2001	0.980 (0.966, 0.996)
Australia	2002 to 2006	0.995 (0.981, 1.010)
Australia	2007 to 2011	1.000 (1.000, 1.000)
Australia	2012 to 2016	0.995 (0.981, 1.009)
Australia	2017 to 2021	0.993 (0.979, 1.007)
Austria	1992 to 1996	0.913 (0.899, 0.927)
Austria	1997 to 2001	0.935 (0.922, 0.948)
Austria	2002 to 2006	0.960 (0.947, 0.974)
Austria	2007 to 2011	1.000 (1.000, 1.000)
Austria	2012 to 2016	1.047 (1.033, 1.061)
Austria	2017 to 2021	1.078 (1.064, 1.093)
Azerbaijan	1992 to 1996	0.962 (0.955, 0.968)
Azerbaijan	1997 to 2001	0.979 (0.973, 0.985)
Azerbaijan	2002 to 2006	0.983 (0.977, 0.989)
Azerbaijan	2007 to 2011	1.000 (1.000, 1.000)

Azerbaijan	2012 to 2016	1.016 (1.011, 1.022)
Azerbaijan	2017 to 2021	1.026 (1.020, 1.032)
Bahamas	1992 to 1996	0.925 (0.902, 0.949)
Bahamas	1997 to 2001	0.941 (0.920, 0.963)
Bahamas	2002 to 2006	0.974 (0.953, 0.995)
Bahamas	2007 to 2011	1.000 (1.000, 1.000)
Bahamas	2012 to 2016	1.009 (0.988, 1.030)
Bahamas	2017 to 2021	1.031 (1.009, 1.052)
Bahrain	1992 to 1996	0.928 (0.916, 0.939)
Bahrain	1997 to 2001	0.946 (0.935, 0.957)
Bahrain	2002 to 2006	0.973 (0.964, 0.983)
Bahrain	2007 to 2011	1.000 (1.000, 1.000)
Bahrain	2012 to 2016	1.034 (1.025, 1.043)
Bahrain	2017 to 2021	1.071 (1.062, 1.081)
Bangladesh	1992 to 1996	1.048 (1.038, 1.058)
Bangladesh	1997 to 2001	1.026 (1.016, 1.035)
Bangladesh	2002 to 2006	1.006 (0.997, 1.015)
Bangladesh	2007 to 2011	1.000 (1.000, 1.000)
Bangladesh	2012 to 2016	1.007 (0.998, 1.016)
Bangladesh	2017 to 2021	1.017 (1.008, 1.025)
Barbados	1992 to 1996	0.969 (0.943, 0.997)
Barbados	1997 to 2001	0.977 (0.952, 1.003)
Barbados	2002 to 2006	0.987 (0.963, 1.012)
Barbados	2007 to 2011	1.000 (1.000, 1.000)

Barbados	2012 to 2016	1.008 (0.984, 1.033)
Barbados	2017 to 2021	1.002 (0.977, 1.027)
Belarus	1992 to 1996	0.991 (0.986, 0.996)
Belarus	1997 to 2001	1.000 (0.995, 1.005)
Belarus	2002 to 2006	0.996 (0.991, 1.001)
Belarus	2007 to 2011	1.000 (1.000, 1.000)
Belarus	2012 to 2016	0.998 (0.993, 1.003)
Belarus	2017 to 2021	0.988 (0.983, 0.993)
Belgium	1992 to 1996	0.891 (0.883, 0.900)
Belgium	1997 to 2001	0.930 (0.922, 0.938)
Belgium	2002 to 2006	0.966 (0.958, 0.974)
Belgium	2007 to 2011	1.000 (1.000, 1.000)
Belgium	2012 to 2016	1.030 (1.021, 1.038)
Belgium	2017 to 2021	1.048 (1.040, 1.057)
Belize	1992 to 1996	0.922 (0.898, 0.947)
Belize	1997 to 2001	0.953 (0.930, 0.977)
Belize	2002 to 2006	0.985 (0.963, 1.008)
Belize	2007 to 2011	1.000 (1.000, 1.000)
Belize	2012 to 2016	1.005 (0.985, 1.026)
Belize	2017 to 2021	1.005 (0.985, 1.026)
Benin	1992 to 1996	0.969 (0.963, 0.974)
Benin	1997 to 2001	0.987 (0.982, 0.993)
Benin	2002 to 2006	1.000 (0.995, 1.005)
Benin	2007 to 2011	1.000 (1.000, 1.000)

Benin	2012 to 2016	1.002 (0.998, 1.007)
Benin	2017 to 2021	1.001 (0.996, 1.006)
Bermuda	1992 to 1996	0.965 (0.913, 1.020)
Bermuda	1997 to 2001	0.968 (0.920, 1.019)
Bermuda	2002 to 2006	0.978 (0.931, 1.027)
Bermuda	2007 to 2011	1.000 (1.000, 1.000)
Bermuda	2012 to 2016	1.018 (0.969, 1.070)
Bermuda	2017 to 2021	1.037 (0.986, 1.091)
Bhutan	1992 to 1996	1.058 (1.040, 1.077)
Bhutan	1997 to 2001	1.031 (1.014, 1.048)
Bhutan	2002 to 2006	1.016 (1.000, 1.033)
Bhutan	2007 to 2011	1.000 (1.000, 1.000)
Bhutan	2012 to 2016	0.991 (0.976, 1.007)
Bhutan	2017 to 2021	1.008 (0.992, 1.024)
Bolivia (Plurinational State of)	1992 to 1996	0.929 (0.920, 0.937)
Bolivia (Plurinational State of)	1997 to 2001	0.952 (0.944, 0.959)
Bolivia (Plurinational State of)	2002 to 2006	0.981 (0.974, 0.989)
Bolivia (Plurinational State of)	2007 to 2011	1.000 (1.000, 1.000)
Bolivia (Plurinational State of)	2012 to 2016	1.009 (1.002, 1.016)
Bolivia (Plurinational State of)	2017 to 2021	1.014 (1.007, 1.022)
Bosnia and Herzegovina	1992 to 1996	0.917 (0.911, 0.923)
Bosnia and Herzegovina	1997 to 2001	0.943 (0.937, 0.949)
Bosnia and Herzegovina	2002 to 2006	0.973 (0.967, 0.979)
Bosnia and Herzegovina	2007 to 2011	1.000 (1.000, 1.000)

Bosnia and Herzegovina	2012 to 2016	1.020 (1.014, 1.027)
Bosnia and Herzegovina	2017 to 2021	1.027 (1.020, 1.033)
Botswana	1992 to 1996	1.116 (1.080, 1.153)
Botswana	1997 to 2001	1.068 (1.036, 1.102)
Botswana	2002 to 2006	1.033 (1.003, 1.064)
Botswana	2007 to 2011	1.000 (1.000, 1.000)
Botswana	2012 to 2016	0.984 (0.957, 1.012)
Botswana	2017 to 2021	0.996 (0.968, 1.024)
Brazil	1992 to 1996	0.983 (0.973, 0.992)
Brazil	1997 to 2001	0.959 (0.951, 0.968)
Brazil	2002 to 2006	0.981 (0.973, 0.989)
Brazil	2007 to 2011	1.000 (1.000, 1.000)
Brazil	2012 to 2016	1.003 (0.995, 1.011)
Brazil	2017 to 2021	1.005 (0.997, 1.014)
Brunei Darussalam	1992 to 1996	1.100 (1.055, 1.146)
Brunei Darussalam	1997 to 2001	1.037 (0.997, 1.078)
Brunei Darussalam	2002 to 2006	0.996 (0.959, 1.034)
Brunei Darussalam	2007 to 2011	1.000 (1.000, 1.000)
Brunei Darussalam	2012 to 2016	1.001 (0.965, 1.037)
Brunei Darussalam	2017 to 2021	0.996 (0.961, 1.033)
Bulgaria	1992 to 1996	1.082 (1.058, 1.105)
Bulgaria	1997 to 2001	1.054 (1.033, 1.076)
Bulgaria	2002 to 2006	1.020 (1.000, 1.041)
Bulgaria	2007 to 2011	1.000 (1.000, 1.000)

Bulgaria	2012 to 2016	0.998 (0.978, 1.019)
Bulgaria	2017 to 2021	0.994 (0.974, 1.015)
Burkina Faso	1992 to 1996	1.058 (1.054, 1.063)
Burkina Faso	1997 to 2001	1.038 (1.033, 1.042)
Burkina Faso	2002 to 2006	1.021 (1.017, 1.025)
Burkina Faso	2007 to 2011	1.000 (1.000, 1.000)
Burkina Faso	2012 to 2016	0.983 (0.979, 0.987)
Burkina Faso	2017 to 2021	0.961 (0.957, 0.964)
Burundi	1992 to 1996	1.028 (1.023, 1.033)
Burundi	1997 to 2001	1.024 (1.019, 1.029)
Burundi	2002 to 2006	1.016 (1.011, 1.020)
Burundi	2007 to 2011	1.000 (1.000, 1.000)
Burundi	2012 to 2016	0.987 (0.983, 0.991)
Burundi	2017 to 2021	0.999 (0.994, 1.003)
Cabo Verde	1992 to 1996	1.051 (1.032, 1.069)
Cabo Verde	1997 to 2001	1.029 (1.012, 1.046)
Cabo Verde	2002 to 2006	1.015 (0.999, 1.031)
Cabo Verde	2007 to 2011	1.000 (1.000, 1.000)
Cabo Verde	2012 to 2016	0.993 (0.978, 1.008)
Cabo Verde	2017 to 2021	1.000 (0.984, 1.015)
Cambodia	1992 to 1996	1.140 (1.135, 1.145)
Cambodia	1997 to 2001	1.092 (1.088, 1.096)
Cambodia	2002 to 2006	1.043 (1.039, 1.047)
Cambodia	2007 to 2011	1.000 (1.000, 1.000)

Cambodia	2012 to 2016	0.979 (0.976, 0.983)
Cambodia	2017 to 2021	0.956 (0.953, 0.960)
Cameroon	1992 to 1996	1.049 (1.042, 1.055)
Cameroon	1997 to 2001	1.023 (1.017, 1.029)
Cameroon	2002 to 2006	1.009 (1.004, 1.014)
Cameroon	2007 to 2011	1.000 (1.000, 1.000)
Cameroon	2012 to 2016	0.986 (0.981, 0.990)
Cameroon	2017 to 2021	0.982 (0.977, 0.987)
Canada	1992 to 1996	0.995 (0.985, 1.005)
Canada	1997 to 2001	0.993 (0.984, 1.002)
Canada	2002 to 2006	0.995 (0.986, 1.004)
Canada	2007 to 2011	1.000 (1.000, 1.000)
Canada	2012 to 2016	0.996 (0.987, 1.005)
Canada	2017 to 2021	0.988 (0.979, 0.997)
Central African Republic	1992 to 1996	1.035 (1.029, 1.042)
Central African Republic	1997 to 2001	1.025 (1.019, 1.031)
Central African Republic	2002 to 2006	1.014 (1.008, 1.019)
Central African Republic	2007 to 2011	1.000 (1.000, 1.000)
Central African Republic	2012 to 2016	1.000 (0.994, 1.005)
Central African Republic	2017 to 2021	1.004 (0.999, 1.010)
Chad	1992 to 1996	1.030 (1.025, 1.036)
Chad	1997 to 2001	1.031 (1.026, 1.037)
Chad	2002 to 2006	1.017 (1.012, 1.022)
Chad	2007 to 2011	1.000 (1.000, 1.000)

Chad	2012 to 2016	0.988 (0.983, 0.993)
Chad	2017 to 2021	0.984 (0.979, 0.989)
Chile	1992 to 1996	0.885 (0.879, 0.890)
Chile	1997 to 2001	0.928 (0.923, 0.933)
Chile	2002 to 2006	0.969 (0.964, 0.974)
Chile	2007 to 2011	1.000 (1.000, 1.000)
Chile	2012 to 2016	1.023 (1.018, 1.029)
Chile	2017 to 2021	1.043 (1.037, 1.048)
China	1992 to 1996	1.257 (1.220, 1.295)
China	1997 to 2001	1.200 (1.167, 1.234)
China	2002 to 2006	1.041 (1.013, 1.071)
China	2007 to 2011	1.000 (1.000, 1.000)
China	2012 to 2016	1.019 (0.992, 1.048)
China	2017 to 2021	1.053 (1.024, 1.083)
Colombia	1992 to 1996	1.093 (1.067, 1.120)
Colombia	1997 to 2001	1.048 (1.024, 1.072)
Colombia	2002 to 2006	1.025 (1.003, 1.048)
Colombia	2007 to 2011	1.000 (1.000, 1.000)
Colombia	2012 to 2016	0.985 (0.964, 1.007)
Colombia	2017 to 2021	0.991 (0.969, 1.013)
Comoros	1992 to 1996	1.039 (1.022, 1.056)
Comoros	1997 to 2001	1.022 (1.006, 1.038)
Comoros	2002 to 2006	1.011 (0.996, 1.026)
Comoros	2007 to 2011	1.000 (1.000, 1.000)

Comoros	2012 to 2016	1.002 (0.988, 1.017)
Comoros	2017 to 2021	0.997 (0.982, 1.011)
Congo	1992 to 1996	1.042 (1.035, 1.049)
Congo	1997 to 2001	1.028 (1.021, 1.034)
Congo	2002 to 2006	1.016 (1.010, 1.022)
Congo	2007 to 2011	1.000 (1.000, 1.000)
Congo	2012 to 2016	0.986 (0.980, 0.991)
Congo	2017 to 2021	0.973 (0.967, 0.978)
Cook Islands	1992 to 1996	1.078 (0.982, 1.183)
Cook Islands	1997 to 2001	1.061 (0.971, 1.159)
Cook Islands	2002 to 2006	1.031 (0.946, 1.123)
Cook Islands	2007 to 2011	1.000 (1.000, 1.000)
Cook Islands	2012 to 2016	0.968 (0.888, 1.056)
Cook Islands	2017 to 2021	0.923 (0.844, 1.011)
Costa Rica	1992 to 1996	0.991 (0.979, 1.003)
Costa Rica	1997 to 2001	0.986 (0.975, 0.997)
Costa Rica	2002 to 2006	0.989 (0.979, 1.000)
Costa Rica	2007 to 2011	1.000 (1.000, 1.000)
Costa Rica	2012 to 2016	1.017 (1.007, 1.028)
Costa Rica	2017 to 2021	1.047 (1.036, 1.057)
Côte d'Ivoire	1992 to 1996	1.051 (1.046, 1.055)
Côte d'Ivoire	1997 to 2001	1.025 (1.021, 1.029)
Côte d'Ivoire	2002 to 2006	1.013 (1.009, 1.017)
Côte d'Ivoire	2007 to 2011	1.000 (1.000, 1.000)

Côte d'Ivoire	2012 to 2016	0.991 (0.987, 0.994)
Côte d'Ivoire	2017 to 2021	0.980 (0.976, 0.984)
Croatia	1992 to 1996	0.990 (0.977, 1.003)
Croatia	1997 to 2001	0.988 (0.976, 0.999)
Croatia	2002 to 2006	0.988 (0.976, 0.999)
Croatia	2007 to 2011	1.000 (1.000, 1.000)
Croatia	2012 to 2016	1.009 (0.997, 1.020)
Croatia	2017 to 2021	1.037 (1.025, 1.050)
Cuba	1992 to 1996	0.967 (0.955, 0.980)
Cuba	1997 to 2001	0.967 (0.955, 0.978)
Cuba	2002 to 2006	0.983 (0.972, 0.994)
Cuba	2007 to 2011	1.000 (1.000, 1.000)
Cuba	2012 to 2016	1.013 (1.001, 1.025)
Cuba	2017 to 2021	1.026 (1.014, 1.039)
Cyprus	1992 to 1996	0.987 (0.968, 1.006)
Cyprus	1997 to 2001	0.981 (0.964, 0.999)
Cyprus	2002 to 2006	0.988 (0.972, 1.004)
Cyprus	2007 to 2011	1.000 (1.000, 1.000)
Cyprus	2012 to 2016	1.019 (1.003, 1.035)
Cyprus	2017 to 2021	1.041 (1.025, 1.057)
Czechia	1992 to 1996	0.975 (0.966, 0.984)
Czechia	1997 to 2001	0.976 (0.968, 0.984)
Czechia	2002 to 2006	0.978 (0.971, 0.986)
Czechia	2007 to 2011	1.000 (1.000, 1.000)

Czechia	2012 to 2016	1.020 (1.012, 1.028)
Czechia	2017 to 2021	1.047 (1.039, 1.055)
Democratic People's Republic of Korea	1992 to 1996	1.052 (1.046, 1.059)
Democratic People's Republic of Korea	1997 to 2001	1.047 (1.041, 1.053)
Democratic People's Republic of Korea	2002 to 2006	1.021 (1.016, 1.027)
Democratic People's Republic of Korea	2007 to 2011	1.000 (1.000, 1.000)
Democratic People's Republic of Korea	2012 to 2016	1.000 (0.995, 1.006)
Democratic People's Republic of Korea	2017 to 2021	1.000 (0.994, 1.005)
Democratic Republic of the Congo	1992 to 1996	1.003 (0.998, 1.007)
Democratic Republic of the Congo	1997 to 2001	1.011 (1.006, 1.015)
Democratic Republic of the Congo	2002 to 2006	1.011 (1.007, 1.015)
Democratic Republic of the Congo	2007 to 2011	1.000 (1.000, 1.000)
Democratic Republic of the Congo	2012 to 2016	0.978 (0.974, 0.982)
Democratic Republic of the Congo	2017 to 2021	0.959 (0.955, 0.963)
Denmark	1992 to 1996	0.896 (0.887, 0.906)
Denmark	1997 to 2001	0.923 (0.914, 0.933)
Denmark	2002 to 2006	0.968 (0.959, 0.977)
Denmark	2007 to 2011	1.000 (1.000, 1.000)
Denmark	2012 to 2016	1.025 (1.015, 1.035)
Denmark	2017 to 2021	1.043 (1.033, 1.053)
Djibouti	1992 to 1996	0.988 (0.972, 1.004)
Djibouti	1997 to 2001	0.991 (0.976, 1.005)
Djibouti	2002 to 2006	1.002 (0.988, 1.016)
Djibouti	2007 to 2011	1.000 (1.000, 1.000)

Djibouti	2012 to 2016	1.013 (1.000, 1.025)
Djibouti	2017 to 2021	1.033 (1.021, 1.046)
Dominica	1992 to 1996	0.982 (0.929, 1.037)
Dominica	1997 to 2001	0.980 (0.930, 1.033)
Dominica	2002 to 2006	0.992 (0.943, 1.043)
Dominica	2007 to 2011	1.000 (1.000, 1.000)
Dominica	2012 to 2016	1.013 (0.964, 1.065)
Dominica	2017 to 2021	1.022 (0.971, 1.076)
Dominican Republic	1992 to 1996	1.011 (1.006, 1.017)
Dominican Republic	1997 to 2001	1.000 (0.994, 1.005)
Dominican Republic	2002 to 2006	0.993 (0.988, 0.998)
Dominican Republic	2007 to 2011	1.000 (1.000, 1.000)
Dominican Republic	2012 to 2016	1.013 (1.008, 1.018)
Dominican Republic	2017 to 2021	1.043 (1.038, 1.048)
Ecuador	1992 to 1996	0.946 (0.941, 0.950)
Ecuador	1997 to 2001	0.964 (0.960, 0.968)
Ecuador	2002 to 2006	0.992 (0.988, 0.996)
Ecuador	2007 to 2011	1.000 (1.000, 1.000)
Ecuador	2012 to 2016	1.013 (1.009, 1.017)
Ecuador	2017 to 2021	1.021 (1.017, 1.025)
Egypt	1992 to 1996	1.015 (0.989, 1.041)
Egypt	1997 to 2001	0.995 (0.971, 1.019)
Egypt	2002 to 2006	1.004 (0.981, 1.028)
Egypt	2007 to 2011	1.000 (1.000, 1.000)

Egypt	2012 to 2016	0.983 (0.961, 1.005)
Egypt	2017 to 2021	0.973 (0.952, 0.995)
El Salvador	1992 to 1996	0.973 (0.960, 0.987)
El Salvador	1997 to 2001	0.980 (0.968, 0.993)
El Salvador	2002 to 2006	0.997 (0.985, 1.010)
El Salvador	2007 to 2011	1.000 (1.000, 1.000)
El Salvador	2012 to 2016	1.004 (0.992, 1.016)
El Salvador	2017 to 2021	1.028 (1.016, 1.041)
Equatorial Guinea	1992 to 1996	1.129 (1.106, 1.152)
Equatorial Guinea	1997 to 2001	1.061 (1.041, 1.081)
Equatorial Guinea	2002 to 2006	1.009 (0.991, 1.027)
Equatorial Guinea	2007 to 2011	1.000 (1.000, 1.000)
Equatorial Guinea	2012 to 2016	1.000 (0.984, 1.017)
Equatorial Guinea	2017 to 2021	1.002 (0.986, 1.019)
Eritrea	1992 to 1996	1.076 (1.069, 1.083)
Eritrea	1997 to 2001	1.047 (1.041, 1.053)
Eritrea	2002 to 2006	1.022 (1.016, 1.028)
Eritrea	2007 to 2011	1.000 (1.000, 1.000)
Eritrea	2012 to 2016	0.987 (0.982, 0.992)
Eritrea	2017 to 2021	0.997 (0.991, 1.002)
Estonia	1992 to 1996	0.994 (0.980, 1.009)
Estonia	1997 to 2001	0.999 (0.985, 1.013)
Estonia	2002 to 2006	0.998 (0.985, 1.012)
Estonia	2007 to 2011	1.000 (1.000, 1.000)

Estonia	2012 to 2016	0.988 (0.974, 1.002)
Estonia	2017 to 2021	0.969 (0.955, 0.983)
Eswatini	1992 to 1996	1.077 (1.056, 1.098)
Eswatini	1997 to 2001	1.056 (1.037, 1.076)
Eswatini	2002 to 2006	1.039 (1.020, 1.058)
Eswatini	2007 to 2011	1.000 (1.000, 1.000)
Eswatini	2012 to 2016	0.965 (0.948, 0.982)
Eswatini	2017 to 2021	0.960 (0.943, 0.978)
Ethiopia	1992 to 1996	1.061 (1.052, 1.070)
Ethiopia	1997 to 2001	1.052 (1.043, 1.060)
Ethiopia	2002 to 2006	1.031 (1.023, 1.039)
Ethiopia	2007 to 2011	1.000 (1.000, 1.000)
Ethiopia	2012 to 2016	0.984 (0.977, 0.991)
Ethiopia	2017 to 2021	1.005 (0.997, 1.012)
Fiji	1992 to 1996	1.061 (1.014, 1.109)
Fiji	1997 to 2001	1.047 (1.005, 1.092)
Fiji	2002 to 2006	1.026 (0.986, 1.068)
Fiji	2007 to 2011	1.000 (1.000, 1.000)
Fiji	2012 to 2016	0.993 (0.954, 1.033)
Fiji	2017 to 2021	1.010 (0.970, 1.051)
Finland	1992 to 1996	0.922 (0.913, 0.932)
Finland	1997 to 2001	0.951 (0.942, 0.960)
Finland	2002 to 2006	0.982 (0.973, 0.992)
Finland	2007 to 2011	1.000 (1.000, 1.000)

Finland	2012 to 2016	1.017 (1.008, 1.027)
Finland	2017 to 2021	1.046 (1.036, 1.056)
France	1992 to 1996	0.896 (0.882, 0.910)
France	1997 to 2001	0.918 (0.905, 0.932)
France	2002 to 2006	0.957 (0.943, 0.970)
France	2007 to 2011	1.000 (1.000, 1.000)
France	2012 to 2016	1.041 (1.026, 1.055)
France	2017 to 2021	1.075 (1.060, 1.090)
Gabon	1992 to 1996	1.062 (1.048, 1.076)
Gabon	1997 to 2001	1.041 (1.028, 1.054)
Gabon	2002 to 2006	1.028 (1.016, 1.041)
Gabon	2007 to 2011	1.000 (1.000, 1.000)
Gabon	2012 to 2016	1.001 (0.990, 1.013)
Gabon	2017 to 2021	1.004 (0.993, 1.016)
Gambia	1992 to 1996	1.177 (1.112, 1.245)
Gambia	1997 to 2001	1.115 (1.057, 1.177)
Gambia	2002 to 2006	1.042 (0.989, 1.097)
Gambia	2007 to 2011	1.000 (1.000, 1.000)
Gambia	2012 to 2016	0.972 (0.925, 1.021)
Gambia	2017 to 2021	0.984 (0.937, 1.034)
Georgia	1992 to 1996	0.988 (0.982, 0.994)
Georgia	1997 to 2001	0.992 (0.987, 0.998)
Georgia	2002 to 2006	0.990 (0.984, 0.995)
Georgia	2007 to 2011	1.000 (1.000, 1.000)

Georgia	2012 to 2016	0.998 (0.993, 1.004)
Georgia	2017 to 2021	0.956 (0.950, 0.961)
Germany	1992 to 1996	0.890 (0.883, 0.898)
Germany	1997 to 2001	0.914 (0.907, 0.921)
Germany	2002 to 2006	0.954 (0.947, 0.961)
Germany	2007 to 2011	1.000 (1.000, 1.000)
Germany	2012 to 2016	1.039 (1.032, 1.047)
Germany	2017 to 2021	1.084 (1.076, 1.092)
Ghana	1992 to 1996	1.102 (1.091, 1.114)
Ghana	1997 to 2001	1.065 (1.055, 1.076)
Ghana	2002 to 2006	1.020 (1.011, 1.030)
Ghana	2007 to 2011	1.000 (1.000, 1.000)
Ghana	2012 to 2016	0.983 (0.974, 0.991)
Ghana	2017 to 2021	0.971 (0.963, 0.980)
Greece	1992 to 1996	0.956 (0.946, 0.966)
Greece	1997 to 2001	0.963 (0.953, 0.972)
Greece	2002 to 2006	0.982 (0.973, 0.992)
Greece	2007 to 2011	1.000 (1.000, 1.000)
Greece	2012 to 2016	1.013 (1.004, 1.023)
Greece	2017 to 2021	1.021 (1.011, 1.031)
Greenland	1992 to 1996	0.973 (0.899, 1.053)
Greenland	1997 to 2001	0.984 (0.912, 1.062)
Greenland	2002 to 2006	0.988 (0.917, 1.063)
Greenland	2007 to 2011	1.000 (1.000, 1.000)

Greenland	2012 to 2016	1.012 (0.939, 1.091)
Greenland	2017 to 2021	1.025 (0.947, 1.108)
Grenada	1992 to 1996	0.990 (0.944, 1.039)
Grenada	1997 to 2001	0.990 (0.947, 1.034)
Grenada	2002 to 2006	0.991 (0.950, 1.033)
Grenada	2007 to 2011	1.000 (1.000, 1.000)
Grenada	2012 to 2016	0.997 (0.957, 1.040)
Grenada	2017 to 2021	1.016 (0.974, 1.060)
Guam	1992 to 1996	1.049 (1.014, 1.086)
Guam	1997 to 2001	1.034 (1.002, 1.067)
Guam	2002 to 2006	1.018 (0.987, 1.049)
Guam	2007 to 2011	1.000 (1.000, 1.000)
Guam	2012 to 2016	0.979 (0.949, 1.010)
Guam	2017 to 2021	0.976 (0.945, 1.007)
Guatemala	1992 to 1996	1.020 (1.007, 1.033)
Guatemala	1997 to 2001	1.001 (0.990, 1.013)
Guatemala	2002 to 2006	0.998 (0.987, 1.009)
Guatemala	2007 to 2011	1.000 (1.000, 1.000)
Guatemala	2012 to 2016	1.012 (1.001, 1.022)
Guatemala	2017 to 2021	1.028 (1.017, 1.039)
Guinea	1992 to 1996	1.050 (1.044, 1.056)
Guinea	1997 to 2001	1.034 (1.028, 1.040)
Guinea	2002 to 2006	1.026 (1.020, 1.031)
Guinea	2007 to 2011	1.000 (1.000, 1.000)

Guinea	2012 to 2016	1.004 (0.999, 1.009)
Guinea	2017 to 2021	1.004 (0.999, 1.009)
Guinea-Bissau	1992 to 1996	1.043 (1.033, 1.054)
Guinea-Bissau	1997 to 2001	1.026 (1.017, 1.036)
Guinea-Bissau	2002 to 2006	1.019 (1.010, 1.029)
Guinea-Bissau	2007 to 2011	1.000 (1.000, 1.000)
Guinea-Bissau	2012 to 2016	1.016 (1.008, 1.025)
Guinea-Bissau	2017 to 2021	1.008 (0.999, 1.017)
Guyana	1992 to 1996	0.982 (0.967, 0.998)
Guyana	1997 to 2001	0.978 (0.963, 0.993)
Guyana	2002 to 2006	0.984 (0.969, 0.999)
Guyana	2007 to 2011	1.000 (1.000, 1.000)
Guyana	2012 to 2016	1.017 (1.001, 1.032)
Guyana	2017 to 2021	1.035 (1.019, 1.051)
Haiti	1992 to 1996	1.004 (0.999, 1.009)
Haiti	1997 to 2001	1.000 (0.995, 1.005)
Haiti	2002 to 2006	0.996 (0.992, 1.001)
Haiti	2007 to 2011	1.000 (1.000, 1.000)
Haiti	2012 to 2016	1.006 (1.002, 1.010)
Haiti	2017 to 2021	1.007 (1.003, 1.011)
Honduras	1992 to 1996	1.007 (0.983, 1.031)
Honduras	1997 to 2001	0.999 (0.977, 1.022)
Honduras	2002 to 2006	1.004 (0.983, 1.025)
Honduras	2007 to 2011	1.000 (1.000, 1.000)

Honduras	2012 to 2016	0.991 (0.972, 1.010)
Honduras	2017 to 2021	1.006 (0.987, 1.026)
Hungary	1992 to 1996	0.966 (0.957, 0.975)
Hungary	1997 to 2001	0.975 (0.966, 0.983)
Hungary	2002 to 2006	0.985 (0.977, 0.993)
Hungary	2007 to 2011	1.000 (1.000, 1.000)
Hungary	2012 to 2016	1.012 (1.004, 1.021)
Hungary	2017 to 2021	1.061 (1.052, 1.070)
Iceland	1992 to 1996	0.914 (0.879, 0.950)
Iceland	1997 to 2001	0.936 (0.902, 0.971)
Iceland	2002 to 2006	0.969 (0.935, 1.004)
Iceland	2007 to 2011	1.000 (1.000, 1.000)
Iceland	2012 to 2016	1.021 (0.986, 1.056)
Iceland	2017 to 2021	1.029 (0.994, 1.066)
India	1992 to 1996	1.050 (1.040, 1.060)
India	1997 to 2001	1.034 (1.025, 1.044)
India	2002 to 2006	1.006 (0.997, 1.015)
India	2007 to 2011	1.000 (1.000, 1.000)
India	2012 to 2016	1.030 (1.021, 1.038)
India	2017 to 2021	1.061 (1.052, 1.070)
Indonesia	1992 to 1996	1.067 (1.052, 1.083)
Indonesia	1997 to 2001	1.043 (1.029, 1.057)
Indonesia	2002 to 2006	1.021 (1.008, 1.035)
Indonesia	2007 to 2011	1.000 (1.000, 1.000)

Indonesia	2012 to 2016	0.999 (0.987, 1.012)
Indonesia	2017 to 2021	1.014 (1.001, 1.026)
Iran (Islamic Republic of)	1992 to 1996	0.780 (0.768, 0.794)
Iran (Islamic Republic of)	1997 to 2001	0.801 (0.789, 0.813)
Iran (Islamic Republic of)	2002 to 2006	0.961 (0.948, 0.974)
Iran (Islamic Republic of)	2007 to 2011	1.000 (1.000, 1.000)
Iran (Islamic Republic of)	2012 to 2016	0.891 (0.880, 0.902)
Iran (Islamic Republic of)	2017 to 2021	0.921 (0.909, 0.933)
Iraq	1992 to 1996	0.953 (0.950, 0.957)
Iraq	1997 to 2001	0.944 (0.940, 0.947)
Iraq	2002 to 2006	0.968 (0.965, 0.971)
Iraq	2007 to 2011	1.000 (1.000, 1.000)
Iraq	2012 to 2016	1.052 (1.049, 1.056)
Iraq	2017 to 2021	1.089 (1.085, 1.092)
Ireland	1992 to 1996	0.913 (0.904, 0.922)
Ireland	1997 to 2001	0.942 (0.934, 0.951)
Ireland	2002 to 2006	0.974 (0.965, 0.982)
Ireland	2007 to 2011	1.000 (1.000, 1.000)
Ireland	2012 to 2016	1.027 (1.019, 1.036)
Ireland	2017 to 2021	1.051 (1.042, 1.060)
Israel	1992 to 1996	0.890 (0.882, 0.899)
Israel	1997 to 2001	0.931 (0.923, 0.940)
Israel	2002 to 2006	0.972 (0.964, 0.980)
Israel	2007 to 2011	1.000 (1.000, 1.000)

Israel	2012 to 2016	1.027 (1.019, 1.035)
Israel	2017 to 2021	1.056 (1.048, 1.064)
Italy	1992 to 1996	0.929 (0.902, 0.956)
Italy	1997 to 2001	0.943 (0.917, 0.969)
Italy	2002 to 2006	0.991 (0.966, 1.017)
Italy	2007 to 2011	1.000 (1.000, 1.000)
Italy	2012 to 2016	1.013 (0.988, 1.039)
Italy	2017 to 2021	1.009 (0.983, 1.036)
Jamaica	1992 to 1996	0.936 (0.928, 0.944)
Jamaica	1997 to 2001	0.961 (0.953, 0.969)
Jamaica	2002 to 2006	0.984 (0.977, 0.992)
Jamaica	2007 to 2011	1.000 (1.000, 1.000)
Jamaica	2012 to 2016	1.004 (0.996, 1.011)
Jamaica	2017 to 2021	1.009 (1.002, 1.017)
Japan	1992 to 1996	1.199 (1.186, 1.214)
Japan	1997 to 2001	1.096 (1.084, 1.109)
Japan	2002 to 2006	0.993 (0.982, 1.005)
Japan	2007 to 2011	1.000 (1.000, 1.000)
Japan	2012 to 2016	1.040 (1.028, 1.052)
Japan	2017 to 2021	1.126 (1.113, 1.139)
Jordan	1992 to 1996	0.968 (0.952, 0.984)
Jordan	1997 to 2001	0.977 (0.962, 0.993)
Jordan	2002 to 2006	0.983 (0.969, 0.998)
Jordan	2007 to 2011	1.000 (1.000, 1.000)

Jordan	2012 to 2016	1.024 (1.012, 1.037)
Jordan	2017 to 2021	1.060 (1.047, 1.074)
Kazakhstan	1992 to 1996	0.958 (0.952, 0.964)
Kazakhstan	1997 to 2001	0.968 (0.962, 0.974)
Kazakhstan	2002 to 2006	0.981 (0.975, 0.987)
Kazakhstan	2007 to 2011	1.000 (1.000, 1.000)
Kazakhstan	2012 to 2016	1.026 (1.020, 1.031)
Kazakhstan	2017 to 2021	1.073 (1.067, 1.079)
Kenya	1992 to 1996	1.011 (1.005, 1.017)
Kenya	1997 to 2001	1.007 (1.002, 1.012)
Kenya	2002 to 2006	1.003 (0.998, 1.008)
Kenya	2007 to 2011	1.000 (1.000, 1.000)
Kenya	2012 to 2016	1.003 (0.998, 1.007)
Kenya	2017 to 2021	1.004 (1.000, 1.009)
Kiribati	1992 to 1996	1.056 (1.018, 1.095)
Kiribati	1997 to 2001	1.058 (1.023, 1.095)
Kiribati	2002 to 2006	1.040 (1.006, 1.074)
Kiribati	2007 to 2011	1.000 (1.000, 1.000)
Kiribati	2012 to 2016	0.970 (0.940, 1.002)
Kiribati	2017 to 2021	0.991 (0.960, 1.023)
Kuwait	1992 to 1996	0.891 (0.885, 0.897)
Kuwait	1997 to 2001	0.925 (0.919, 0.932)
Kuwait	2002 to 2006	0.963 (0.958, 0.969)
Kuwait	2007 to 2011	1.000 (1.000, 1.000)

Kuwait	2012 to 2016	1.035 (1.030, 1.040)
Kuwait	2017 to 2021	1.063 (1.058, 1.069)
Kyrgyzstan	1992 to 1996	0.971 (0.965, 0.977)
Kyrgyzstan	1997 to 2001	0.984 (0.978, 0.990)
Kyrgyzstan	2002 to 2006	0.992 (0.987, 0.997)
Kyrgyzstan	2007 to 2011	1.000 (1.000, 1.000)
Kyrgyzstan	2012 to 2016	0.989 (0.984, 0.995)
Kyrgyzstan	2017 to 2021	0.977 (0.972, 0.983)
Lao People's Democratic Republic	1992 to 1996	1.116 (1.108, 1.123)
Lao People's Democratic Republic	1997 to 2001	1.067 (1.060, 1.074)
Lao People's Democratic Republic	2002 to 2006	1.026 (1.020, 1.032)
Lao People's Democratic Republic	2007 to 2011	1.000 (1.000, 1.000)
Lao People's Democratic Republic	2012 to 2016	0.994 (0.988, 1.000)
Lao People's Democratic Republic	2017 to 2021	0.994 (0.989, 1.000)
Latvia	1992 to 1996	1.010 (0.998, 1.022)
Latvia	1997 to 2001	1.020 (1.009, 1.031)
Latvia	2002 to 2006	1.003 (0.992, 1.014)
Latvia	2007 to 2011	1.000 (1.000, 1.000)
Latvia	2012 to 2016	0.996 (0.985, 1.007)
Latvia	2017 to 2021	0.983 (0.971, 0.994)
Lebanon	1992 to 1996	0.930 (0.924, 0.935)
Lebanon	1997 to 2001	0.942 (0.937, 0.947)
Lebanon	2002 to 2006	0.967 (0.962, 0.972)
Lebanon	2007 to 2011	1.000 (1.000, 1.000)

Lebanon	2012 to 2016	1.034 (1.029, 1.039)
Lebanon	2017 to 2021	1.077 (1.072, 1.082)
Lesotho	1992 to 1996	1.011 (1.001, 1.021)
Lesotho	1997 to 2001	1.010 (1.001, 1.020)
Lesotho	2002 to 2006	1.008 (0.999, 1.017)
Lesotho	2007 to 2011	1.000 (1.000, 1.000)
Lesotho	2012 to 2016	0.983 (0.974, 0.992)
Lesotho	2017 to 2021	0.985 (0.976, 0.994)
Liberia	1992 to 1996	1.016 (1.009, 1.023)
Liberia	1997 to 2001	0.991 (0.985, 0.997)
Liberia	2002 to 2006	1.001 (0.995, 1.007)
Liberia	2007 to 2011	1.000 (1.000, 1.000)
Liberia	2012 to 2016	1.004 (0.999, 1.010)
Liberia	2017 to 2021	1.000 (0.994, 1.005)
Libya	1992 to 1996	0.941 (0.933, 0.948)
Libya	1997 to 2001	0.938 (0.931, 0.945)
Libya	2002 to 2006	0.961 (0.955, 0.968)
Libya	2007 to 2011	1.000 (1.000, 1.000)
Libya	2012 to 2016	1.042 (1.036, 1.049)
Libya	2017 to 2021	1.063 (1.056, 1.069)
Lithuania	1992 to 1996	0.999 (0.989, 1.009)
Lithuania	1997 to 2001	1.007 (0.998, 1.016)
Lithuania	2002 to 2006	0.999 (0.990, 1.008)
Lithuania	2007 to 2011	1.000 (1.000, 1.000)

Lithuania	2012 to 2016	0.983 (0.974, 0.993)
Lithuania	2017 to 2021	0.977 (0.967, 0.987)
Luxembourg	1992 to 1996	0.930 (0.902, 0.959)
Luxembourg	1997 to 2001	0.953 (0.927, 0.981)
Luxembourg	2002 to 2006	0.977 (0.951, 1.004)
Luxembourg	2007 to 2011	1.000 (1.000, 1.000)
Luxembourg	2012 to 2016	1.024 (0.999, 1.051)
Luxembourg	2017 to 2021	1.058 (1.031, 1.084)
Madagascar	1992 to 1996	1.043 (1.038, 1.048)
Madagascar	1997 to 2001	1.021 (1.017, 1.026)
Madagascar	2002 to 2006	1.009 (1.004, 1.013)
Madagascar	2007 to 2011	1.000 (1.000, 1.000)
Madagascar	2012 to 2016	0.987 (0.983, 0.991)
Madagascar	2017 to 2021	0.989 (0.985, 0.993)
Malawi	1992 to 1996	1.048 (1.041, 1.056)
Malawi	1997 to 2001	1.039 (1.032, 1.046)
Malawi	2002 to 2006	1.029 (1.023, 1.036)
Malawi	2007 to 2011	1.000 (1.000, 1.000)
Malawi	2012 to 2016	0.979 (0.973, 0.984)
Malawi	2017 to 2021	0.980 (0.974, 0.986)
Malaysia	1992 to 1996	1.017 (1.002, 1.032)
Malaysia	1997 to 2001	1.024 (1.011, 1.038)
Malaysia	2002 to 2006	1.011 (0.998, 1.024)
Malaysia	2007 to 2011	1.000 (1.000, 1.000)

Malaysia	2012 to 2016	1.003 (0.990, 1.015)
Malaysia	2017 to 2021	1.017 (1.005, 1.030)
Maldives	1992 to 1996	1.147 (1.115, 1.180)
Maldives	1997 to 2001	1.089 (1.061, 1.118)
Maldives	2002 to 2006	1.042 (1.016, 1.068)
Maldives	2007 to 2011	1.000 (1.000, 1.000)
Maldives	2012 to 2016	0.989 (0.967, 1.013)
Maldives	2017 to 2021	1.005 (0.982, 1.028)
Mali	1992 to 1996	1.034 (1.029, 1.039)
Mali	1997 to 2001	1.014 (1.009, 1.019)
Mali	2002 to 2006	1.004 (1.000, 1.009)
Mali	2007 to 2011	1.000 (1.000, 1.000)
Mali	2012 to 2016	0.994 (0.990, 0.998)
Mali	2017 to 2021	0.982 (0.978, 0.987)
Malta	1992 to 1996	0.937 (0.908, 0.967)
Malta	1997 to 2001	0.955 (0.928, 0.983)
Malta	2002 to 2006	0.982 (0.955, 1.009)
Malta	2007 to 2011	1.000 (1.000, 1.000)
Malta	2012 to 2016	1.021 (0.994, 1.049)
Malta	2017 to 2021	1.048 (1.020, 1.077)
Marshall Islands	1992 to 1996	1.067 (1.008, 1.130)
Marshall Islands	1997 to 2001	1.043 (0.988, 1.101)
Marshall Islands	2002 to 2006	1.016 (0.964, 1.070)
Marshall Islands	2007 to 2011	1.000 (1.000, 1.000)

Marshall Islands	2012 to 2016	0.998 (0.948, 1.051)
Marshall Islands	2017 to 2021	0.988 (0.938, 1.041)
Mauritania	1992 to 1996	1.029 (1.023, 1.035)
Mauritania	1997 to 2001	1.016 (1.010, 1.022)
Mauritania	2002 to 2006	1.013 (1.007, 1.019)
Mauritania	2007 to 2011	1.000 (1.000, 1.000)
Mauritania	2012 to 2016	0.996 (0.990, 1.001)
Mauritania	2017 to 2021	0.996 (0.991, 1.001)
Mauritius	1992 to 1996	1.007 (0.995, 1.018)
Mauritius	1997 to 2001	1.004 (0.994, 1.015)
Mauritius	2002 to 2006	1.002 (0.992, 1.013)
Mauritius	2007 to 2011	1.000 (1.000, 1.000)
Mauritius	2012 to 2016	0.997 (0.987, 1.008)
Mauritius	2017 to 2021	1.003 (0.993, 1.014)
Mexico	1992 to 1996	0.984 (0.976, 0.992)
Mexico	1997 to 2001	0.970 (0.962, 0.977)
Mexico	2002 to 2006	0.974 (0.966, 0.981)
Mexico	2007 to 2011	1.000 (1.000, 1.000)
Mexico	2012 to 2016	1.022 (1.015, 1.030)
Mexico	2017 to 2021	1.050 (1.042, 1.057)
Micronesia (Federated States of)	1992 to 1996	1.109 (1.066, 1.154)
Micronesia (Federated States of)	1997 to 2001	1.084 (1.044, 1.126)
Micronesia (Federated States of)	2002 to 2006	1.046 (1.008, 1.086)
Micronesia (Federated States of)	2007 to 2011	1.000 (1.000, 1.000)

Micronesia (Federated States of)	2012 to 2016	0.981 (0.944, 1.019)
Micronesia (Federated States of)	2017 to 2021	0.990 (0.952, 1.029)
Monaco	1992 to 1996	0.920 (0.815, 1.039)
Monaco	1997 to 2001	0.946 (0.846, 1.058)
Monaco	2002 to 2006	0.975 (0.877, 1.083)
Monaco	2007 to 2011	1.000 (1.000, 1.000)
Monaco	2012 to 2016	1.024 (0.924, 1.134)
Monaco	2017 to 2021	1.064 (0.959, 1.180)
Mongolia	1992 to 1996	0.982 (0.976, 0.988)
Mongolia	1997 to 2001	0.974 (0.968, 0.980)
Mongolia	2002 to 2006	0.976 (0.970, 0.981)
Mongolia	2007 to 2011	1.000 (1.000, 1.000)
Mongolia	2012 to 2016	0.973 (0.968, 0.978)
Mongolia	2017 to 2021	0.875 (0.870, 0.880)
Montenegro	1992 to 1996	0.938 (0.919, 0.958)
Montenegro	1997 to 2001	0.949 (0.931, 0.968)
Montenegro	2002 to 2006	0.968 (0.950, 0.987)
Montenegro	2007 to 2011	1.000 (1.000, 1.000)
Montenegro	2012 to 2016	1.032 (1.013, 1.052)
Montenegro	2017 to 2021	1.069 (1.049, 1.089)
Morocco	1992 to 1996	0.940 (0.936, 0.943)
Morocco	1997 to 2001	0.946 (0.943, 0.949)
Morocco	2002 to 2006	0.966 (0.963, 0.969)
Morocco	2007 to 2011	1.000 (1.000, 1.000)

Morocco	2012 to 2016	1.056 (1.053, 1.059)
Morocco	2017 to 2021	1.101 (1.098, 1.104)
Mozambique	1992 to 1996	1.060 (1.055, 1.064)
Mozambique	1997 to 2001	1.034 (1.029, 1.038)
Mozambique	2002 to 2006	1.020 (1.016, 1.024)
Mozambique	2007 to 2011	1.000 (1.000, 1.000)
Mozambique	2012 to 2016	0.979 (0.976, 0.983)
Mozambique	2017 to 2021	0.961 (0.957, 0.965)
Myanmar	1992 to 1996	1.022 (1.015, 1.029)
Myanmar	1997 to 2001	1.009 (1.003, 1.016)
Myanmar	2002 to 2006	0.998 (0.992, 1.004)
Myanmar	2007 to 2011	1.000 (1.000, 1.000)
Myanmar	2012 to 2016	1.012 (1.006, 1.019)
Myanmar	2017 to 2021	1.026 (1.020, 1.032)
Namibia	1992 to 1996	1.041 (1.031, 1.051)
Namibia	1997 to 2001	1.030 (1.020, 1.039)
Namibia	2002 to 2006	1.020 (1.011, 1.030)
Namibia	2007 to 2011	1.000 (1.000, 1.000)
Namibia	2012 to 2016	0.983 (0.974, 0.992)
Namibia	2017 to 2021	0.994 (0.985, 1.003)
Nauru	1992 to 1996	1.144 (1.009, 1.296)
Nauru	1997 to 2001	1.093 (0.969, 1.233)
Nauru	2002 to 2006	1.040 (0.923, 1.171)
Nauru	2007 to 2011	1.000 (1.000, 1.000)

Nauru	2012 to 2016	0.977 (0.865, 1.103)
Nauru	2017 to 2021	0.973 (0.860, 1.100)
Nepal	1992 to 1996	1.015 (1.007, 1.024)
Nepal	1997 to 2001	1.008 (1.000, 1.016)
Nepal	2002 to 2006	0.995 (0.987, 1.002)
Nepal	2007 to 2011	1.000 (1.000, 1.000)
Nepal	2012 to 2016	1.031 (1.024, 1.039)
Nepal	2017 to 2021	1.039 (1.032, 1.047)
Netherlands	1992 to 1996	0.905 (0.898, 0.911)
Netherlands	1997 to 2001	0.941 (0.935, 0.948)
Netherlands	2002 to 2006	0.969 (0.963, 0.976)
Netherlands	2007 to 2011	1.000 (1.000, 1.000)
Netherlands	2012 to 2016	1.042 (1.035, 1.049)
Netherlands	2017 to 2021	1.060 (1.053, 1.067)
New Zealand	1992 to 1996	1.021 (0.994, 1.049)
New Zealand	1997 to 2001	1.010 (0.985, 1.036)
New Zealand	2002 to 2006	1.008 (0.984, 1.033)
New Zealand	2007 to 2011	1.000 (1.000, 1.000)
New Zealand	2012 to 2016	1.004 (0.980, 1.028)
New Zealand	2017 to 2021	1.025 (1.001, 1.050)
Nicaragua	1992 to 1996	0.988 (0.976, 1.000)
Nicaragua	1997 to 2001	0.975 (0.964, 0.986)
Nicaragua	2002 to 2006	0.989 (0.978, 1.000)
Nicaragua	2007 to 2011	1.000 (1.000, 1.000)

Nicaragua	2012 to 2016	1.011 (1.001, 1.021)
Nicaragua	2017 to 2021	1.035 (1.025, 1.046)
Niger	1992 to 1996	1.032 (1.028, 1.036)
Niger	1997 to 2001	1.019 (1.016, 1.023)
Niger	2002 to 2006	1.016 (1.012, 1.019)
Niger	2007 to 2011	1.000 (1.000, 1.000)
Niger	2012 to 2016	1.003 (1.000, 1.006)
Niger	2017 to 2021	0.993 (0.989, 0.996)
Nigeria	1992 to 1996	1.052 (1.048, 1.057)
Nigeria	1997 to 2001	1.051 (1.046, 1.055)
Nigeria	2002 to 2006	1.028 (1.024, 1.033)
Nigeria	2007 to 2011	1.000 (1.000, 1.000)
Nigeria	2012 to 2016	0.968 (0.965, 0.972)
Nigeria	2017 to 2021	0.964 (0.961, 0.968)
Niue	1992 to 1996	1.126 (0.814, 1.558)
Niue	1997 to 2001	1.082 (0.792, 1.479)
Niue	2002 to 2006	1.014 (0.737, 1.394)
Niue	2007 to 2011	1.000 (1.000, 1.000)
Niue	2012 to 2016	0.976 (0.710, 1.344)
Niue	2017 to 2021	0.980 (0.712, 1.350)
North Macedonia	1992 to 1996	0.934 (0.925, 0.944)
North Macedonia	1997 to 2001	0.953 (0.944, 0.962)
North Macedonia	2002 to 2006	0.968 (0.960, 0.977)
North Macedonia	2007 to 2011	1.000 (1.000, 1.000)

North Macedonia	2012 to 2016	1.040 (1.031, 1.050)
North Macedonia	2017 to 2021	1.085 (1.076, 1.095)
Northern Mariana Islands	1992 to 1996	1.061 (1.011, 1.113)
Northern Mariana Islands	1997 to 2001	1.046 (1.003, 1.090)
Northern Mariana Islands	2002 to 2006	1.027 (0.986, 1.070)
Northern Mariana Islands	2007 to 2011	1.000 (1.000, 1.000)
Northern Mariana Islands	2012 to 2016	0.965 (0.922, 1.009)
Northern Mariana Islands	2017 to 2021	0.951 (0.906, 0.998)
Norway	1992 to 1996	0.926 (0.916, 0.937)
Norway	1997 to 2001	0.946 (0.936, 0.956)
Norway	2002 to 2006	0.974 (0.964, 0.984)
Norway	2007 to 2011	1.000 (1.000, 1.000)
Norway	2012 to 2016	1.026 (1.017, 1.036)
Norway	2017 to 2021	1.060 (1.050, 1.070)
Oman	1992 to 1996	0.921 (0.898, 0.944)
Oman	1997 to 2001	0.960 (0.938, 0.982)
Oman	2002 to 2006	0.980 (0.960, 1.001)
Oman	2007 to 2011	1.000 (1.000, 1.000)
Oman	2012 to 2016	1.036 (1.017, 1.056)
Oman	2017 to 2021	1.077 (1.057, 1.097)
Pakistan	1992 to 1996	0.995 (0.988, 1.002)
Pakistan	1997 to 2001	0.986 (0.980, 0.993)
Pakistan	2002 to 2006	0.992 (0.986, 0.998)
Pakistan	2007 to 2011	1.000 (1.000, 1.000)

Pakistan	2012 to 2016	1.032 (1.026, 1.038)
Pakistan	2017 to 2021	0.997 (0.991, 1.003)
Palau	1992 to 1996	1.070 (0.976, 1.173)
Palau	1997 to 2001	1.057 (0.971, 1.150)
Palau	2002 to 2006	1.034 (0.953, 1.121)
Palau	2007 to 2011	1.000 (1.000, 1.000)
Palau	2012 to 2016	0.980 (0.901, 1.067)
Palau	2017 to 2021	0.968 (0.887, 1.057)
Palestine	1992 to 1996	0.970 (0.958, 0.982)
Palestine	1997 to 2001	0.979 (0.968, 0.991)
Palestine	2002 to 2006	0.989 (0.978, 0.999)
Palestine	2007 to 2011	1.000 (1.000, 1.000)
Palestine	2012 to 2016	1.019 (1.009, 1.028)
Palestine	2017 to 2021	1.047 (1.037, 1.057)
Panama	1992 to 1996	0.970 (0.962, 0.977)
Panama	1997 to 2001	0.971 (0.963, 0.978)
Panama	2002 to 2006	0.979 (0.972, 0.986)
Panama	2007 to 2011	1.000 (1.000, 1.000)
Panama	2012 to 2016	1.025 (1.018, 1.032)
Panama	2017 to 2021	1.053 (1.046, 1.060)
Papua New Guinea	1992 to 1996	1.099 (1.079, 1.120)
Papua New Guinea	1997 to 2001	1.058 (1.040, 1.077)
Papua New Guinea	2002 to 2006	1.028 (1.010, 1.045)
Papua New Guinea	2007 to 2011	1.000 (1.000, 1.000)

Papua New Guinea	2012 to 2016	0.978 (0.963, 0.994)
Papua New Guinea	2017 to 2021	0.972 (0.957, 0.988)
Paraguay	1992 to 1996	0.992 (0.986, 0.999)
Paraguay	1997 to 2001	0.984 (0.978, 0.991)
Paraguay	2002 to 2006	0.992 (0.986, 0.998)
Paraguay	2007 to 2011	1.000 (1.000, 1.000)
Paraguay	2012 to 2016	1.021 (1.016, 1.027)
Paraguay	2017 to 2021	1.052 (1.047, 1.058)
Peru	1992 to 1996	0.958 (0.951, 0.965)
Peru	1997 to 2001	0.960 (0.953, 0.967)
Peru	2002 to 2006	0.976 (0.969, 0.983)
Peru	2007 to 2011	1.000 (1.000, 1.000)
Peru	2012 to 2016	1.020 (1.013, 1.027)
Peru	2017 to 2021	1.032 (1.025, 1.038)
Philippines	1992 to 1996	1.137 (1.117, 1.156)
Philippines	1997 to 2001	1.090 (1.072, 1.108)
Philippines	2002 to 2006	1.039 (1.023, 1.055)
Philippines	2007 to 2011	1.000 (1.000, 1.000)
Philippines	2012 to 2016	0.979 (0.965, 0.994)
Philippines	2017 to 2021	0.968 (0.953, 0.983)
Poland	1992 to 1996	0.982 (0.977, 0.987)
Poland	1997 to 2001	0.978 (0.974, 0.983)
Poland	2002 to 2006	0.983 (0.978, 0.987)
Poland	2007 to 2011	1.000 (1.000, 1.000)

Poland	2012 to 2016	1.016 (1.012, 1.021)
Poland	2017 to 2021	1.013 (1.009, 1.018)
Portugal	1992 to 1996	0.892 (0.886, 0.898)
Portugal	1997 to 2001	0.922 (0.915, 0.928)
Portugal	2002 to 2006	0.960 (0.954, 0.966)
Portugal	2007 to 2011	1.000 (1.000, 1.000)
Portugal	2012 to 2016	1.031 (1.025, 1.037)
Portugal	2017 to 2021	1.057 (1.051, 1.064)
Puerto Rico	1992 to 1996	0.946 (0.940, 0.953)
Puerto Rico	1997 to 2001	0.965 (0.958, 0.971)
Puerto Rico	2002 to 2006	0.986 (0.980, 0.993)
Puerto Rico	2007 to 2011	1.000 (1.000, 1.000)
Puerto Rico	2012 to 2016	0.998 (0.992, 1.005)
Puerto Rico	2017 to 2021	0.985 (0.978, 0.991)
Qatar	1992 to 1996	0.894 (0.881, 0.906)
Qatar	1997 to 2001	0.927 (0.916, 0.938)
Qatar	2002 to 2006	0.966 (0.956, 0.977)
Qatar	2007 to 2011	1.000 (1.000, 1.000)
Qatar	2012 to 2016	1.036 (1.027, 1.045)
Qatar	2017 to 2021	1.062 (1.053, 1.070)
Republic of Korea	1992 to 1996	1.178 (1.137, 1.220)
Republic of Korea	1997 to 2001	1.040 (1.005, 1.077)
Republic of Korea	2002 to 2006	1.011 (0.978, 1.045)
Republic of Korea	2007 to 2011	1.000 (1.000, 1.000)

Republic of Korea	2012 to 2016	1.023 (0.989, 1.058)
Republic of Korea	2017 to 2021	0.920 (0.889, 0.953)
Republic of Moldova	1992 to 1996	1.084 (1.068, 1.101)
Republic of Moldova	1997 to 2001	1.063 (1.048, 1.078)
Republic of Moldova	2002 to 2006	1.032 (1.018, 1.047)
Republic of Moldova	2007 to 2011	1.000 (1.000, 1.000)
Republic of Moldova	2012 to 2016	0.980 (0.965, 0.994)
Republic of Moldova	2017 to 2021	0.956 (0.941, 0.970)
Romania	1992 to 1996	1.086 (1.050, 1.123)
Romania	1997 to 2001	1.054 (1.021, 1.089)
Romania	2002 to 2006	1.027 (0.996, 1.060)
Romania	2007 to 2011	1.000 (1.000, 1.000)
Romania	2012 to 2016	0.982 (0.951, 1.014)
Romania	2017 to 2021	0.974 (0.942, 1.007)
Russian Federation	1992 to 1996	0.957 (0.949, 0.964)
Russian Federation	1997 to 2001	0.993 (0.986, 1.000)
Russian Federation	2002 to 2006	1.003 (0.996, 1.010)
Russian Federation	2007 to 2011	1.000 (1.000, 1.000)
Russian Federation	2012 to 2016	0.991 (0.985, 0.998)
Russian Federation	2017 to 2021	0.984 (0.977, 0.991)
Rwanda	1992 to 1996	1.100 (1.093, 1.106)
Rwanda	1997 to 2001	1.065 (1.059, 1.071)
Rwanda	2002 to 2006	1.028 (1.023, 1.034)
Rwanda	2007 to 2011	1.000 (1.000, 1.000)

Rwanda	2012 to 2016	0.979 (0.974, 0.984)
Rwanda	2017 to 2021	0.979 (0.974, 0.984)
Saint Kitts and Nevis	1992 to 1996	1.009 (0.947, 1.075)
Saint Kitts and Nevis	1997 to 2001	1.002 (0.945, 1.063)
Saint Kitts and Nevis	2002 to 2006	0.999 (0.945, 1.056)
Saint Kitts and Nevis	2007 to 2011	1.000 (1.000, 1.000)
Saint Kitts and Nevis	2012 to 2016	1.013 (0.960, 1.068)
Saint Kitts and Nevis	2017 to 2021	1.033 (0.979, 1.089)
Saint Lucia	1992 to 1996	1.000 (0.962, 1.039)
Saint Lucia	1997 to 2001	0.995 (0.959, 1.031)
Saint Lucia	2002 to 2006	0.992 (0.958, 1.027)
Saint Lucia	2007 to 2011	1.000 (1.000, 1.000)
Saint Lucia	2012 to 2016	1.006 (0.973, 1.040)
Saint Lucia	2017 to 2021	1.004 (0.970, 1.038)
Saint Vincent and the Grenadines	1992 to 1996	0.984 (0.942, 1.028)
Saint Vincent and the Grenadines	1997 to 2001	0.981 (0.941, 1.023)
Saint Vincent and the Grenadines	2002 to 2006	0.983 (0.944, 1.024)
Saint Vincent and the Grenadines	2007 to 2011	1.000 (1.000, 1.000)
Saint Vincent and the Grenadines	2012 to 2016	1.022 (0.982, 1.064)
Saint Vincent and the Grenadines	2017 to 2021	1.027 (0.986, 1.069)
Samoa	1992 to 1996	1.058 (1.026, 1.092)
Samoa	1997 to 2001	1.039 (1.008, 1.070)
Samoa	2002 to 2006	1.020 (0.991, 1.050)
Samoa	2007 to 2011	1.000 (1.000, 1.000)

Samoa	2012 to 2016	0.987 (0.959, 1.015)
Samoa	2017 to 2021	0.988 (0.960, 1.017)
San Marino	1992 to 1996	0.929 (0.826, 1.045)
San Marino	1997 to 2001	0.955 (0.857, 1.065)
San Marino	2002 to 2006	0.976 (0.881, 1.082)
San Marino	2007 to 2011	1.000 (1.000, 1.000)
San Marino	2012 to 2016	1.026 (0.928, 1.135)
San Marino	2017 to 2021	1.053 (0.950, 1.167)
Sao Tome and Principe	1992 to 1996	1.060 (1.028, 1.094)
Sao Tome and Principe	1997 to 2001	1.041 (1.011, 1.073)
Sao Tome and Principe	2002 to 2006	1.036 (1.007, 1.066)
Sao Tome and Principe	2007 to 2011	1.000 (1.000, 1.000)
Sao Tome and Principe	2012 to 2016	0.984 (0.958, 1.011)
Sao Tome and Principe	2017 to 2021	0.977 (0.951, 1.005)
Saudi Arabia	1992 to 1996	0.961 (0.934, 0.988)
Saudi Arabia	1997 to 2001	0.976 (0.951, 1.002)
Saudi Arabia	2002 to 2006	0.985 (0.961, 1.009)
Saudi Arabia	2007 to 2011	1.000 (1.000, 1.000)
Saudi Arabia	2012 to 2016	1.032 (1.010, 1.054)
Saudi Arabia	2017 to 2021	1.066 (1.044, 1.088)
Senegal	1992 to 1996	1.020 (1.016, 1.025)
Senegal	1997 to 2001	0.998 (0.994, 1.001)
Senegal	2002 to 2006	0.994 (0.991, 0.998)
Senegal	2007 to 2011	1.000 (1.000, 1.000)

Senegal	2012 to 2016	0.996 (0.993, 1.000)
Senegal	2017 to 2021	0.985 (0.982, 0.989)
Serbia	1992 to 1996	0.935 (0.930, 0.940)
Serbia	1997 to 2001	0.954 (0.949, 0.959)
Serbia	2002 to 2006	0.971 (0.966, 0.976)
Serbia	2007 to 2011	1.000 (1.000, 1.000)
Serbia	2012 to 2016	1.037 (1.032, 1.042)
Serbia	2017 to 2021	1.070 (1.065, 1.076)
Seychelles	1992 to 1996	1.055 (1.011, 1.101)
Seychelles	1997 to 2001	1.044 (1.003, 1.086)
Seychelles	2002 to 2006	1.030 (0.992, 1.069)
Seychelles	2007 to 2011	1.000 (1.000, 1.000)
Seychelles	2012 to 2016	0.989 (0.953, 1.026)
Seychelles	2017 to 2021	1.002 (0.965, 1.040)
Sierra Leone	1992 to 1996	1.042 (1.037, 1.048)
Sierra Leone	1997 to 2001	1.033 (1.027, 1.038)
Sierra Leone	2002 to 2006	1.027 (1.022, 1.032)
Sierra Leone	2007 to 2011	1.000 (1.000, 1.000)
Sierra Leone	2012 to 2016	1.002 (0.998, 1.007)
Sierra Leone	2017 to 2021	1.002 (0.998, 1.007)
Singapore	1992 to 1996	1.092 (1.067, 1.117)
Singapore	1997 to 2001	1.066 (1.044, 1.089)
Singapore	2002 to 2006	1.033 (1.013, 1.054)
Singapore	2007 to 2011	1.000 (1.000, 1.000)

Singapore	2012 to 2016	0.985 (0.966, 1.004)
Singapore	2017 to 2021	0.982 (0.963, 1.002)
Slovakia	1992 to 1996	0.988 (0.978, 0.998)
Slovakia	1997 to 2001	0.992 (0.983, 1.002)
Slovakia	2002 to 2006	0.994 (0.984, 1.003)
Slovakia	2007 to 2011	1.000 (1.000, 1.000)
Slovakia	2012 to 2016	1.015 (1.006, 1.024)
Slovakia	2017 to 2021	1.049 (1.039, 1.058)
Slovenia	1992 to 1996	0.976 (0.964, 0.988)
Slovenia	1997 to 2001	0.979 (0.967, 0.990)
Slovenia	2002 to 2006	0.984 (0.973, 0.995)
Slovenia	2007 to 2011	1.000 (1.000, 1.000)
Slovenia	2012 to 2016	1.015 (1.004, 1.027)
Slovenia	2017 to 2021	1.035 (1.023, 1.047)
Solomon Islands	1992 to 1996	1.147 (1.099, 1.197)
Solomon Islands	1997 to 2001	1.103 (1.060, 1.149)
Solomon Islands	2002 to 2006	1.055 (1.015, 1.096)
Solomon Islands	2007 to 2011	1.000 (1.000, 1.000)
Solomon Islands	2012 to 2016	0.954 (0.920, 0.990)
Solomon Islands	2017 to 2021	0.950 (0.916, 0.986)
Somalia	1992 to 1996	1.004 (1.001, 1.008)
Somalia	1997 to 2001	1.006 (1.003, 1.010)
Somalia	2002 to 2006	1.004 (1.000, 1.007)
Somalia	2007 to 2011	1.000 (1.000, 1.000)

Somalia	2012 to 2016	1.001 (0.998, 1.004)
Somalia	2017 to 2021	0.997 (0.994, 1.000)
South Africa	1992 to 1996	1.018 (1.000, 1.037)
South Africa	1997 to 2001	1.020 (1.003, 1.037)
South Africa	2002 to 2006	1.017 (1.001, 1.033)
South Africa	2007 to 2011	1.000 (1.000, 1.000)
South Africa	2012 to 2016	0.990 (0.975, 1.006)
South Africa	2017 to 2021	1.013 (0.998, 1.030)
South Sudan	1992 to 1996	0.998 (0.992, 1.004)
South Sudan	1997 to 2001	1.005 (0.999, 1.011)
South Sudan	2002 to 2006	1.005 (1.000, 1.011)
South Sudan	2007 to 2011	1.000 (1.000, 1.000)
South Sudan	2012 to 2016	1.012 (1.007, 1.017)
South Sudan	2017 to 2021	1.035 (1.030, 1.041)
Spain	1992 to 1996	0.965 (0.952, 0.978)
Spain	1997 to 2001	0.984 (0.972, 0.996)
Spain	2002 to 2006	0.992 (0.980, 1.003)
Spain	2007 to 2011	1.000 (1.000, 1.000)
Spain	2012 to 2016	1.036 (1.024, 1.048)
Spain	2017 to 2021	1.067 (1.054, 1.079)
Sri Lanka	1992 to 1996	1.016 (1.009, 1.023)
Sri Lanka	1997 to 2001	1.009 (1.003, 1.016)
Sri Lanka	2002 to 2006	1.000 (0.994, 1.006)
Sri Lanka	2007 to 2011	1.000 (1.000, 1.000)

Sri Lanka	2012 to 2016	1.013 (1.007, 1.019)
Sri Lanka	2017 to 2021	1.035 (1.029, 1.041)
Sudan	1992 to 1996	1.006 (1.003, 1.010)
Sudan	1997 to 2001	0.999 (0.996, 1.002)
Sudan	2002 to 2006	1.001 (0.998, 1.004)
Sudan	2007 to 2011	1.000 (1.000, 1.000)
Sudan	2012 to 2016	1.017 (1.014, 1.020)
Sudan	2017 to 2021	1.043 (1.040, 1.046)
Suriname	1992 to 1996	0.957 (0.937, 0.977)
Suriname	1997 to 2001	0.968 (0.949, 0.987)
Suriname	2002 to 2006	0.982 (0.964, 1.000)
Suriname	2007 to 2011	1.000 (1.000, 1.000)
Suriname	2012 to 2016	1.020 (1.002, 1.038)
Suriname	2017 to 2021	1.033 (1.015, 1.052)
Sweden	1992 to 1996	0.897 (0.890, 0.904)
Sweden	1997 to 2001	0.933 (0.926, 0.940)
Sweden	2002 to 2006	0.964 (0.957, 0.971)
Sweden	2007 to 2011	1.000 (1.000, 1.000)
Sweden	2012 to 2016	1.034 (1.027, 1.041)
Sweden	2017 to 2021	1.052 (1.045, 1.060)
Switzerland	1992 to 1996	0.908 (0.901, 0.915)
Switzerland	1997 to 2001	0.933 (0.926, 0.940)
Switzerland	2002 to 2006	0.967 (0.960, 0.974)
Switzerland	2007 to 2011	1.000 (1.000, 1.000)

Switzerland	2012 to 2016	1.031 (1.024, 1.038)
Switzerland	2017 to 2021	1.067 (1.060, 1.075)
Syrian Arab Republic	1992 to 1996	0.960 (0.950, 0.970)
Syrian Arab Republic	1997 to 2001	0.972 (0.963, 0.982)
Syrian Arab Republic	2002 to 2006	0.984 (0.976, 0.993)
Syrian Arab Republic	2007 to 2011	1.000 (1.000, 1.000)
Syrian Arab Republic	2012 to 2016	1.034 (1.025, 1.043)
Syrian Arab Republic	2017 to 2021	1.055 (1.045, 1.064)
Taiwan (Province of China)	1992 to 1996	1.254 (1.202, 1.308)
Taiwan (Province of China)	1997 to 2001	1.160 (1.115, 1.207)
Taiwan (Province of China)	2002 to 2006	1.072 (1.031, 1.114)
Taiwan (Province of China)	2007 to 2011	1.000 (1.000, 1.000)
Taiwan (Province of China)	2012 to 2016	0.999 (0.961, 1.039)
Taiwan (Province of China)	2017 to 2021	0.976 (0.938, 1.016)
Tajikistan	1992 to 1996	1.006 (1.001, 1.011)
Tajikistan	1997 to 2001	1.012 (1.007, 1.017)
Tajikistan	2002 to 2006	1.005 (1.000, 1.009)
Tajikistan	2007 to 2011	1.000 (1.000, 1.000)
Tajikistan	2012 to 2016	1.016 (1.012, 1.021)
Tajikistan	2017 to 2021	1.025 (1.021, 1.030)
Thailand	1992 to 1996	1.110 (1.075, 1.147)
Thailand	1997 to 2001	1.062 (1.031, 1.095)
Thailand	2002 to 2006	1.029 (0.999, 1.059)
Thailand	2007 to 2011	1.000 (1.000, 1.000)

Thailand	2012 to 2016	0.990 (0.962, 1.020)
Thailand	2017 to 2021	0.999 (0.970, 1.030)
Timor-Leste	1992 to 1996	1.051 (1.038, 1.065)
Timor-Leste	1997 to 2001	1.028 (1.015, 1.041)
Timor-Leste	2002 to 2006	1.005 (0.992, 1.017)
Timor-Leste	2007 to 2011	1.000 (1.000, 1.000)
Timor-Leste	2012 to 2016	1.002 (0.990, 1.014)
Timor-Leste	2017 to 2021	1.014 (1.002, 1.026)
Togo	1992 to 1996	1.003 (0.998, 1.008)
Togo	1997 to 2001	1.001 (0.997, 1.006)
Togo	2002 to 2006	1.001 (0.996, 1.005)
Togo	2007 to 2011	1.000 (1.000, 1.000)
Togo	2012 to 2016	0.999 (0.995, 1.004)
Togo	2017 to 2021	0.994 (0.990, 0.999)
Tokelau	1992 to 1996	1.060 (0.734, 1.529)
Tokelau	1997 to 2001	1.037 (0.733, 1.467)
Tokelau	2002 to 2006	1.023 (0.718, 1.457)
Tokelau	2007 to 2011	1.000 (1.000, 1.000)
Tokelau	2012 to 2016	1.004 (0.704, 1.431)
Tokelau	2017 to 2021	0.975 (0.683, 1.391)
Tonga	1992 to 1996	1.153 (1.102, 1.206)
Tonga	1997 to 2001	1.110 (1.063, 1.159)
Tonga	2002 to 2006	1.058 (1.014, 1.104)
Tonga	2007 to 2011	1.000 (1.000, 1.000)

Tonga	2012 to 2016	0.963 (0.923, 1.005)
Tonga	2017 to 2021	0.948 (0.907, 0.990)
Trinidad and Tobago	1992 to 1996	0.957 (0.945, 0.969)
Trinidad and Tobago	1997 to 2001	0.964 (0.952, 0.976)
Trinidad and Tobago	2002 to 2006	0.979 (0.968, 0.991)
Trinidad and Tobago	2007 to 2011	1.000 (1.000, 1.000)
Trinidad and Tobago	2012 to 2016	1.014 (1.002, 1.025)
Trinidad and Tobago	2017 to 2021	1.030 (1.018, 1.042)
Tunisia	1992 to 1996	0.997 (0.971, 1.024)
Tunisia	1997 to 2001	0.987 (0.963, 1.011)
Tunisia	2002 to 2006	0.998 (0.975, 1.022)
Tunisia	2007 to 2011	1.000 (1.000, 1.000)
Tunisia	2012 to 2016	1.013 (0.990, 1.036)
Tunisia	2017 to 2021	1.040 (1.017, 1.064)
Turkey	1992 to 1996	0.977 (0.968, 0.987)
Turkey	1997 to 2001	0.976 (0.967, 0.984)
Turkey	2002 to 2006	0.982 (0.974, 0.991)
Turkey	2007 to 2011	1.000 (1.000, 1.000)
Turkey	2012 to 2016	1.026 (1.018, 1.035)
Turkey	2017 to 2021	1.066 (1.057, 1.075)
Turkmenistan	1992 to 1996	0.971 (0.966, 0.976)
Turkmenistan	1997 to 2001	0.975 (0.970, 0.980)
Turkmenistan	2002 to 2006	0.985 (0.980, 0.990)
Turkmenistan	2007 to 2011	1.000 (1.000, 1.000)

Turkmenistan	2012 to 2016	1.015 (1.010, 1.020)
Turkmenistan	2017 to 2021	1.017 (1.012, 1.022)
Tuvalu	1992 to 1996	1.042 (0.921, 1.180)
Tuvalu	1997 to 2001	1.027 (0.912, 1.157)
Tuvalu	2002 to 2006	1.014 (0.903, 1.139)
Tuvalu	2007 to 2011	1.000 (1.000, 1.000)
Tuvalu	2012 to 2016	0.988 (0.880, 1.108)
Tuvalu	2017 to 2021	0.995 (0.886, 1.118)
Uganda	1992 to 1996	1.077 (1.068, 1.085)
Uganda	1997 to 2001	1.048 (1.040, 1.056)
Uganda	2002 to 2006	1.029 (1.022, 1.037)
Uganda	2007 to 2011	1.000 (1.000, 1.000)
Uganda	2012 to 2016	0.969 (0.963, 0.976)
Uganda	2017 to 2021	0.962 (0.955, 0.969)
Ukraine	1992 to 1996	0.884 (0.879, 0.888)
Ukraine	1997 to 2001	0.918 (0.914, 0.923)
Ukraine	2002 to 2006	0.958 (0.954, 0.962)
Ukraine	2007 to 2011	1.000 (1.000, 1.000)
Ukraine	2012 to 2016	0.993 (0.988, 0.997)
Ukraine	2017 to 2021	0.972 (0.967, 0.976)
United Arab Emirates	1992 to 1996	0.893 (0.887, 0.899)
United Arab Emirates	1997 to 2001	0.905 (0.900, 0.911)
United Arab Emirates	2002 to 2006	0.949 (0.944, 0.954)
United Arab Emirates	2007 to 2011	1.000 (1.000, 1.000)

United Arab Emirates	2012 to 2016	1.057 (1.053, 1.062)
United Arab Emirates	2017 to 2021	1.107 (1.103, 1.112)
United Kingdom	1992 to 1996	0.882 (0.872, 0.893)
United Kingdom	1997 to 2001	0.918 (0.907, 0.928)
United Kingdom	2002 to 2006	0.966 (0.956, 0.977)
United Kingdom	2007 to 2011	1.000 (1.000, 1.000)
United Kingdom	2012 to 2016	1.023 (1.012, 1.033)
United Kingdom	2017 to 2021	1.056 (1.045, 1.067)
United Republic of Tanzania	1992 to 1996	1.042 (1.035, 1.048)
United Republic of Tanzania	1997 to 2001	1.030 (1.024, 1.037)
United Republic of Tanzania	2002 to 2006	1.023 (1.017, 1.029)
United Republic of Tanzania	2007 to 2011	1.000 (1.000, 1.000)
United Republic of Tanzania	2012 to 2016	0.989 (0.984, 0.994)
United Republic of Tanzania	2017 to 2021	0.995 (0.990, 1.000)
United States of America	1992 to 1996	0.882 (0.871, 0.894)
United States of America	1997 to 2001	0.929 (0.918, 0.941)
United States of America	2002 to 2006	0.967 (0.956, 0.978)
United States of America	2007 to 2011	1.000 (1.000, 1.000)
United States of America	2012 to 2016	1.030 (1.019, 1.042)
United States of America	2017 to 2021	1.047 (1.035, 1.059)
United States Virgin Islands	1992 to 1996	0.961 (0.917, 1.008)
United States Virgin Islands	1997 to 2001	0.974 (0.933, 1.018)
United States Virgin Islands	2002 to 2006	0.985 (0.945, 1.027)
United States Virgin Islands	2007 to 2011	1.000 (1.000, 1.000)

United States Virgin Islands	2012 to 2016	1.016 (0.973, 1.061)
United States Virgin Islands	2017 to 2021	1.026 (0.980, 1.074)
Uruguay	1992 to 1996	0.931 (0.920, 0.941)
Uruguay	1997 to 2001	0.954 (0.944, 0.964)
Uruguay	2002 to 2006	0.982 (0.972, 0.992)
Uruguay	2007 to 2011	1.000 (1.000, 1.000)
Uruguay	2012 to 2016	1.025 (1.015, 1.036)
Uruguay	2017 to 2021	1.055 (1.044, 1.066)
Uzbekistan	1992 to 1996	0.993 (0.988, 0.998)
Uzbekistan	1997 to 2001	0.999 (0.995, 1.004)
Uzbekistan	2002 to 2006	0.996 (0.992, 1.001)
Uzbekistan	2007 to 2011	1.000 (1.000, 1.000)
Uzbekistan	2012 to 2016	1.002 (0.998, 1.007)
Uzbekistan	2017 to 2021	1.008 (1.003, 1.012)
Vanuatu	1992 to 1996	1.060 (1.030, 1.092)
Vanuatu	1997 to 2001	1.045 (1.016, 1.074)
Vanuatu	2002 to 2006	1.015 (0.989, 1.042)
Vanuatu	2007 to 2011	1.000 (1.000, 1.000)
Vanuatu	2012 to 2016	0.986 (0.962, 1.011)
Vanuatu	2017 to 2021	1.000 (0.975, 1.025)
Venezuela (Bolivarian Republic of)	1992 to 1996	0.988 (0.982, 0.995)
Venezuela (Bolivarian Republic of)	1997 to 2001	0.983 (0.976, 0.989)
Venezuela (Bolivarian Republic of)	2002 to 2006	0.987 (0.981, 0.993)
Venezuela (Bolivarian Republic of)	2007 to 2011	1.000 (1.000, 1.000)

Venezuela (Bolivarian Republic of)	2012 to 2016	1.031 (1.025, 1.037)
Venezuela (Bolivarian Republic of)	2017 to 2021	1.064 (1.058, 1.070)
Viet Nam	1992 to 1996	1.127 (1.116, 1.137)
Viet Nam	1997 to 2001	1.063 (1.054, 1.072)
Viet Nam	2002 to 2006	1.026 (1.018, 1.035)
Viet Nam	2007 to 2011	1.000 (1.000, 1.000)
Viet Nam	2012 to 2016	0.992 (0.984, 1.000)
Viet Nam	2017 to 2021	0.983 (0.975, 0.991)
Yemen	1992 to 1996	1.049 (1.038, 1.061)
Yemen	1997 to 2001	1.022 (1.012, 1.033)
Yemen	2002 to 2006	1.003 (0.994, 1.012)
Yemen	2007 to 2011	1.000 (1.000, 1.000)
Yemen	2012 to 2016	1.007 (0.999, 1.016)
Yemen	2017 to 2021	1.032 (1.023, 1.041)
Zambia	1992 to 1996	1.052 (1.046, 1.058)
Zambia	1997 to 2001	1.043 (1.037, 1.049)
Zambia	2002 to 2006	1.027 (1.022, 1.033)
Zambia	2007 to 2011	1.000 (1.000, 1.000)
Zambia	2012 to 2016	0.986 (0.981, 0.991)
Zambia	2017 to 2021	1.003 (0.998, 1.008)
Zimbabwe	1992 to 1996	1.047 (1.029, 1.066)
Zimbabwe	1997 to 2001	1.021 (1.004, 1.038)
Zimbabwe	2002 to 2006	1.009 (0.992, 1.026)
Zimbabwe	2007 to 2011	1.000 (1.000, 1.000)

Zimbabwe	2012 to 2016	0.970 (0.955, 0.986)
Zimbabwe	2017 to 2021	0.959 (0.944, 0.975)

CLD, chronic liver disease; WCBA, women of childbearing age.

Table S10 Cohort effects on CLD prevalence in WCBA across countries.

Location	Cohort	Prevalence rate ratio
Afghanistan	1942 to 1951	0.847 (0.839, 0.855)
Afghanistan	1947 to 1956	0.863 (0.857, 0.869)
Afghanistan	1952 to 1961	0.883 (0.877, 0.889)
Afghanistan	1957 to 1966	0.905 (0.900, 0.911)
Afghanistan	1962 to 1971	0.925 (0.921, 0.929)
Afghanistan	1967 to 1976	0.945 (0.941, 0.949)
Afghanistan	1972 to 1981	0.963 (0.960, 0.967)
Afghanistan	1977 to 1986	0.984 (0.979, 0.988)
Afghanistan	1982 to 1991	1.000 (1.000, 1.000)
Afghanistan	1987 to 1996	1.010 (1.006, 1.015)
Afghanistan	1992 to 2001	1.017 (1.011, 1.022)
Afghanistan	1997 to 2006	1.012 (1.004, 1.020)
Albania	1942 to 1951	0.924 (0.893, 0.956)
Albania	1947 to 1956	0.937 (0.913, 0.962)
Albania	1952 to 1961	0.952 (0.931, 0.973)
Albania	1957 to 1966	0.962 (0.943, 0.982)
Albania	1962 to 1971	0.971 (0.953, 0.990)
Albania	1967 to 1976	0.977 (0.959, 0.995)
Albania	1972 to 1981	0.987 (0.969, 1.006)
Albania	1977 to 1986	0.996 (0.977, 1.016)
Albania	1982 to 1991	1.000 (1.000, 1.000)
Albania	1987 to 1996	0.932 (0.910, 0.954)

Albania	1992 to 2001	0.887 (0.859, 0.915)
Albania	1997 to 2006	0.852 (0.807, 0.899)
Algeria	1942 to 1951	0.752 (0.745, 0.759)
Algeria	1947 to 1956	0.783 (0.778, 0.788)
Algeria	1952 to 1961	0.813 (0.808, 0.817)
Algeria	1957 to 1966	0.842 (0.838, 0.846)
Algeria	1962 to 1971	0.873 (0.870, 0.877)
Algeria	1967 to 1976	0.906 (0.903, 0.909)
Algeria	1972 to 1981	0.938 (0.935, 0.941)
Algeria	1977 to 1986	0.970 (0.966, 0.973)
Algeria	1982 to 1991	1.000 (1.000, 1.000)
Algeria	1987 to 1996	1.029 (1.024, 1.033)
Algeria	1992 to 2001	1.052 (1.046, 1.058)
Algeria	1997 to 2006	0.957 (0.948, 0.966)
American Samoa	1942 to 1951	0.875 (0.773, 0.990)
American Samoa	1947 to 1956	0.891 (0.813, 0.977)
American Samoa	1952 to 1961	0.908 (0.838, 0.982)
American Samoa	1957 to 1966	0.925 (0.862, 0.994)
American Samoa	1962 to 1971	0.942 (0.882, 1.007)
American Samoa	1967 to 1976	0.958 (0.898, 1.022)
American Samoa	1972 to 1981	0.977 (0.917, 1.042)
American Samoa	1977 to 1986	0.991 (0.927, 1.060)
American Samoa	1982 to 1991	1.000 (1.000, 1.000)
American Samoa	1987 to 1996	0.906 (0.834, 0.985)

American Samoa	1992 to 2001	0.776 (0.696, 0.865)
American Samoa	1997 to 2006	0.739 (0.629, 0.869)
Andorra	1942 to 1951	0.783 (0.667, 0.919)
Andorra	1947 to 1956	0.814 (0.720, 0.921)
Andorra	1952 to 1961	0.844 (0.759, 0.939)
Andorra	1957 to 1966	0.871 (0.791, 0.958)
Andorra	1962 to 1971	0.898 (0.820, 0.983)
Andorra	1967 to 1976	0.923 (0.846, 1.007)
Andorra	1972 to 1981	0.952 (0.873, 1.039)
Andorra	1977 to 1986	0.978 (0.893, 1.070)
Andorra	1982 to 1991	1.000 (1.000, 1.000)
Andorra	1987 to 1996	1.014 (0.902, 1.140)
Andorra	1992 to 2001	0.857 (0.730, 1.004)
Andorra	1997 to 2006	0.778 (0.609, 0.995)
Angola	1942 to 1951	1.201 (1.183, 1.218)
Angola	1947 to 1956	1.181 (1.169, 1.192)
Angola	1952 to 1961	1.161 (1.151, 1.170)
Angola	1957 to 1966	1.137 (1.130, 1.145)
Angola	1962 to 1971	1.114 (1.107, 1.120)
Angola	1967 to 1976	1.088 (1.082, 1.093)
Angola	1972 to 1981	1.063 (1.058, 1.068)
Angola	1977 to 1986	1.033 (1.028, 1.039)
Angola	1982 to 1991	1.000 (1.000, 1.000)
Angola	1987 to 1996	0.962 (0.956, 0.967)

Angola	1992 to 2001	0.916 (0.910, 0.921)
Angola	1997 to 2006	0.828 (0.821, 0.835)
Antigua and Barbuda	1942 to 1951	1.001 (0.894, 1.120)
Antigua and Barbuda	1947 to 1956	1.002 (0.923, 1.088)
Antigua and Barbuda	1952 to 1961	1.001 (0.933, 1.075)
Antigua and Barbuda	1957 to 1966	1.001 (0.939, 1.067)
Antigua and Barbuda	1962 to 1971	1.001 (0.943, 1.062)
Antigua and Barbuda	1967 to 1976	1.000 (0.945, 1.059)
Antigua and Barbuda	1972 to 1981	1.001 (0.946, 1.060)
Antigua and Barbuda	1977 to 1986	1.001 (0.943, 1.061)
Antigua and Barbuda	1982 to 1991	1.000 (1.000, 1.000)
Antigua and Barbuda	1987 to 1996	0.997 (0.927, 1.072)
Antigua and Barbuda	1992 to 2001	0.930 (0.844, 1.025)
Antigua and Barbuda	1997 to 2006	0.882 (0.750, 1.036)
Argentina	1942 to 1951	0.715 (0.704, 0.726)
Argentina	1947 to 1956	0.749 (0.740, 0.758)
Argentina	1952 to 1961	0.784 (0.776, 0.792)
Argentina	1957 to 1966	0.820 (0.812, 0.828)
Argentina	1962 to 1971	0.856 (0.848, 0.863)
Argentina	1967 to 1976	0.890 (0.883, 0.897)
Argentina	1972 to 1981	0.928 (0.921, 0.935)
Argentina	1977 to 1986	0.964 (0.957, 0.972)
Argentina	1982 to 1991	1.000 (1.000, 1.000)
Argentina	1987 to 1996	1.038 (1.028, 1.048)

Argentina	1992 to 2001	1.045 (1.032, 1.057)
Argentina	1997 to 2006	1.014 (0.994, 1.035)
Armenia	1942 to 1951	0.858 (0.844, 0.872)
Armenia	1947 to 1956	0.873 (0.864, 0.883)
Armenia	1952 to 1961	0.891 (0.882, 0.899)
Armenia	1957 to 1966	0.909 (0.901, 0.917)
Armenia	1962 to 1971	0.927 (0.920, 0.935)
Armenia	1967 to 1976	0.946 (0.939, 0.954)
Armenia	1972 to 1981	0.967 (0.960, 0.975)
Armenia	1977 to 1986	0.984 (0.977, 0.992)
Armenia	1982 to 1991	1.000 (1.000, 1.000)
Armenia	1987 to 1996	1.013 (1.004, 1.023)
Armenia	1992 to 2001	0.992 (0.978, 1.006)
Armenia	1997 to 2006	0.927 (0.905, 0.948)
Australia	1942 to 1951	0.856 (0.827, 0.886)
Australia	1947 to 1956	0.882 (0.859, 0.906)
Australia	1952 to 1961	0.903 (0.882, 0.924)
Australia	1957 to 1966	0.919 (0.899, 0.938)
Australia	1962 to 1971	0.936 (0.918, 0.955)
Australia	1967 to 1976	0.950 (0.932, 0.968)
Australia	1972 to 1981	0.968 (0.950, 0.986)
Australia	1977 to 1986	0.985 (0.966, 1.003)
Australia	1982 to 1991	1.000 (1.000, 1.000)
Australia	1987 to 1996	1.008 (0.986, 1.031)

Australia	1992 to 2001	0.820 (0.795, 0.846)
Australia	1997 to 2006	0.718 (0.683, 0.755)
Austria	1942 to 1951	0.704 (0.681, 0.726)
Austria	1947 to 1956	0.746 (0.728, 0.765)
Austria	1952 to 1961	0.784 (0.766, 0.801)
Austria	1957 to 1966	0.812 (0.796, 0.829)
Austria	1962 to 1971	0.848 (0.832, 0.864)
Austria	1967 to 1976	0.881 (0.866, 0.898)
Austria	1972 to 1981	0.917 (0.900, 0.934)
Austria	1977 to 1986	0.957 (0.939, 0.975)
Austria	1982 to 1991	1.000 (1.000, 1.000)
Austria	1987 to 1996	1.019 (0.996, 1.043)
Austria	1992 to 2001	0.997 (0.967, 1.028)
Austria	1997 to 2006	0.936 (0.889, 0.985)
Azerbaijan	1942 to 1951	0.874 (0.857, 0.891)
Azerbaijan	1947 to 1956	0.882 (0.872, 0.893)
Azerbaijan	1952 to 1961	0.900 (0.891, 0.908)
Azerbaijan	1957 to 1966	0.917 (0.909, 0.925)
Azerbaijan	1962 to 1971	0.936 (0.929, 0.943)
Azerbaijan	1967 to 1976	0.953 (0.946, 0.960)
Azerbaijan	1972 to 1981	0.971 (0.964, 0.979)
Azerbaijan	1977 to 1986	0.989 (0.982, 0.996)
Azerbaijan	1982 to 1991	1.000 (1.000, 1.000)
Azerbaijan	1987 to 1996	1.003 (0.995, 1.012)

Azerbaijan	1992 to 2001	0.995 (0.984, 1.007)
Azerbaijan	1997 to 2006	0.902 (0.886, 0.919)
Bahamas	1942 to 1951	0.822 (0.775, 0.872)
Bahamas	1947 to 1956	0.839 (0.804, 0.876)
Bahamas	1952 to 1961	0.860 (0.830, 0.892)
Bahamas	1957 to 1966	0.881 (0.853, 0.910)
Bahamas	1962 to 1971	0.902 (0.875, 0.930)
Bahamas	1967 to 1976	0.926 (0.899, 0.953)
Bahamas	1972 to 1981	0.950 (0.923, 0.978)
Bahamas	1977 to 1986	0.974 (0.945, 1.004)
Bahamas	1982 to 1991	1.000 (1.000, 1.000)
Bahamas	1987 to 1996	1.020 (0.984, 1.057)
Bahamas	1992 to 2001	1.034 (0.986, 1.084)
Bahamas	1997 to 2006	0.955 (0.888, 1.028)
Bahrain	1942 to 1951	0.754 (0.727, 0.782)
Bahrain	1947 to 1956	0.779 (0.761, 0.797)
Bahrain	1952 to 1961	0.805 (0.791, 0.819)
Bahrain	1957 to 1966	0.836 (0.824, 0.849)
Bahrain	1962 to 1971	0.866 (0.855, 0.878)
Bahrain	1967 to 1976	0.898 (0.886, 0.909)
Bahrain	1972 to 1981	0.933 (0.922, 0.944)
Bahrain	1977 to 1986	0.967 (0.956, 0.979)
Bahrain	1982 to 1991	1.000 (1.000, 1.000)
Bahrain	1987 to 1996	0.979 (0.965, 0.994)

Bahrain	1992 to 2001	0.981 (0.962, 1.001)
Bahrain	1997 to 2006	1.001 (0.970, 1.033)
Bangladesh	1942 to 1951	0.989 (0.963, 1.016)
Bangladesh	1947 to 1956	0.998 (0.979, 1.017)
Bangladesh	1952 to 1961	1.005 (0.990, 1.021)
Bangladesh	1957 to 1966	1.007 (0.994, 1.021)
Bangladesh	1962 to 1971	1.009 (0.998, 1.021)
Bangladesh	1967 to 1976	1.011 (1.001, 1.022)
Bangladesh	1972 to 1981	1.013 (1.003, 1.024)
Bangladesh	1977 to 1986	1.008 (0.998, 1.019)
Bangladesh	1982 to 1991	1.000 (1.000, 1.000)
Bangladesh	1987 to 1996	0.987 (0.975, 0.999)
Bangladesh	1992 to 2001	0.963 (0.948, 0.978)
Bangladesh	1997 to 2006	0.798 (0.778, 0.818)
Barbados	1942 to 1951	0.925 (0.872, 0.983)
Barbados	1947 to 1956	0.929 (0.888, 0.973)
Barbados	1952 to 1961	0.942 (0.905, 0.981)
Barbados	1957 to 1966	0.953 (0.918, 0.990)
Barbados	1962 to 1971	0.962 (0.928, 0.997)
Barbados	1967 to 1976	0.973 (0.940, 1.008)
Barbados	1972 to 1981	0.986 (0.952, 1.021)
Barbados	1977 to 1986	0.992 (0.957, 1.029)
Barbados	1982 to 1991	1.000 (1.000, 1.000)
Barbados	1987 to 1996	1.003 (0.959, 1.049)

Barbados	1992 to 2001	1.002 (0.945, 1.063)
Barbados	1997 to 2006	0.952 (0.867, 1.045)
Belarus	1942 to 1951	0.926 (0.915, 0.937)
Belarus	1947 to 1956	0.937 (0.929, 0.945)
Belarus	1952 to 1961	0.948 (0.941, 0.955)
Belarus	1957 to 1966	0.958 (0.951, 0.964)
Belarus	1962 to 1971	0.965 (0.959, 0.972)
Belarus	1967 to 1976	0.972 (0.966, 0.978)
Belarus	1972 to 1981	0.982 (0.976, 0.988)
Belarus	1977 to 1986	0.993 (0.987, 0.999)
Belarus	1982 to 1991	1.000 (1.000, 1.000)
Belarus	1987 to 1996	0.998 (0.990, 1.006)
Belarus	1992 to 2001	0.886 (0.876, 0.896)
Belarus	1997 to 2006	0.789 (0.775, 0.804)
Belgium	1942 to 1951	0.737 (0.723, 0.751)
Belgium	1947 to 1956	0.769 (0.757, 0.780)
Belgium	1952 to 1961	0.800 (0.789, 0.811)
Belgium	1957 to 1966	0.829 (0.819, 0.840)
Belgium	1962 to 1971	0.860 (0.850, 0.870)
Belgium	1967 to 1976	0.891 (0.881, 0.901)
Belgium	1972 to 1981	0.927 (0.917, 0.938)
Belgium	1977 to 1986	0.965 (0.954, 0.976)
Belgium	1982 to 1991	1.000 (1.000, 1.000)
Belgium	1987 to 1996	1.034 (1.020, 1.048)

Belgium	1992 to 2001	1.013 (0.995, 1.032)
Belgium	1997 to 2006	0.968 (0.939, 0.998)
Belize	1942 to 1951	0.849 (0.791, 0.912)
Belize	1947 to 1956	0.864 (0.823, 0.908)
Belize	1952 to 1961	0.881 (0.847, 0.916)
Belize	1957 to 1966	0.899 (0.869, 0.930)
Belize	1962 to 1971	0.920 (0.893, 0.948)
Belize	1967 to 1976	0.940 (0.915, 0.967)
Belize	1972 to 1981	0.963 (0.938, 0.989)
Belize	1977 to 1986	0.980 (0.955, 1.007)
Belize	1982 to 1991	1.000 (1.000, 1.000)
Belize	1987 to 1996	1.018 (0.987, 1.049)
Belize	1992 to 2001	0.994 (0.957, 1.033)
Belize	1997 to 2006	0.949 (0.895, 1.006)
Benin	1942 to 1951	0.917 (0.903, 0.932)
Benin	1947 to 1956	0.926 (0.916, 0.936)
Benin	1952 to 1961	0.937 (0.929, 0.945)
Benin	1957 to 1966	0.947 (0.940, 0.954)
Benin	1962 to 1971	0.958 (0.951, 0.964)
Benin	1967 to 1976	0.970 (0.964, 0.975)
Benin	1972 to 1981	0.982 (0.976, 0.988)
Benin	1977 to 1986	0.992 (0.986, 0.997)
Benin	1982 to 1991	1.000 (1.000, 1.000)
Benin	1987 to 1996	1.007 (1.000, 1.013)

Benin	1992 to 2001	1.005 (0.998, 1.013)
Benin	1997 to 2006	0.823 (0.814, 0.833)
Bermuda	1942 to 1951	0.877 (0.783, 0.983)
Bermuda	1947 to 1956	0.887 (0.810, 0.972)
Bermuda	1952 to 1961	0.902 (0.831, 0.979)
Bermuda	1957 to 1966	0.919 (0.851, 0.992)
Bermuda	1962 to 1971	0.932 (0.866, 1.004)
Bermuda	1967 to 1976	0.948 (0.882, 1.019)
Bermuda	1972 to 1981	0.968 (0.899, 1.041)
Bermuda	1977 to 1986	0.984 (0.912, 1.062)
Bermuda	1982 to 1991	1.000 (1.000, 1.000)
Bermuda	1987 to 1996	1.015 (0.919, 1.121)
Bermuda	1992 to 2001	1.027 (0.897, 1.175)
Bermuda	1997 to 2006	0.975 (0.779, 1.221)
Bhutan	1942 to 1951	0.934 (0.893, 0.978)
Bhutan	1947 to 1956	0.949 (0.919, 0.981)
Bhutan	1952 to 1961	0.962 (0.937, 0.989)
Bhutan	1957 to 1966	0.972 (0.950, 0.995)
Bhutan	1962 to 1971	0.981 (0.960, 1.001)
Bhutan	1967 to 1976	0.987 (0.968, 1.006)
Bhutan	1972 to 1981	0.997 (0.979, 1.015)
Bhutan	1977 to 1986	0.999 (0.981, 1.018)
Bhutan	1982 to 1991	1.000 (1.000, 1.000)
Bhutan	1987 to 1996	0.993 (0.972, 1.014)

Bhutan	1992 to 2001	0.779 (0.757, 0.801)
Bhutan	1997 to 2006	0.632 (0.603, 0.663)
Bolivia (Plurinational State of)	1942 to 1951	0.864 (0.846, 0.883)
Bolivia (Plurinational State of)	1947 to 1956	0.879 (0.866, 0.893)
Bolivia (Plurinational State of)	1952 to 1961	0.893 (0.882, 0.905)
Bolivia (Plurinational State of)	1957 to 1966	0.910 (0.899, 0.920)
Bolivia (Plurinational State of)	1962 to 1971	0.926 (0.917, 0.936)
Bolivia (Plurinational State of)	1967 to 1976	0.943 (0.934, 0.952)
Bolivia (Plurinational State of)	1972 to 1981	0.962 (0.953, 0.971)
Bolivia (Plurinational State of)	1977 to 1986	0.982 (0.973, 0.991)
Bolivia (Plurinational State of)	1982 to 1991	1.000 (1.000, 1.000)
Bolivia (Plurinational State of)	1987 to 1996	1.023 (1.012, 1.034)
Bolivia (Plurinational State of)	1992 to 2001	1.033 (1.019, 1.048)
Bolivia (Plurinational State of)	1997 to 2006	1.031 (1.010, 1.053)
Bosnia and Herzegovina	1942 to 1951	0.809 (0.797, 0.821)
Bosnia and Herzegovina	1947 to 1956	0.833 (0.824, 0.843)
Bosnia and Herzegovina	1952 to 1961	0.855 (0.846, 0.863)
Bosnia and Herzegovina	1957 to 1966	0.880 (0.872, 0.888)
Bosnia and Herzegovina	1962 to 1971	0.905 (0.897, 0.913)
Bosnia and Herzegovina	1967 to 1976	0.930 (0.921, 0.938)
Bosnia and Herzegovina	1972 to 1981	0.955 (0.947, 0.963)
Bosnia and Herzegovina	1977 to 1986	0.978 (0.970, 0.987)
Bosnia and Herzegovina	1982 to 1991	1.000 (1.000, 1.000)
Bosnia and Herzegovina	1987 to 1996	1.016 (1.005, 1.028)

Bosnia and Herzegovina	1992 to 2001	1.027 (1.011, 1.043)
Bosnia and Herzegovina	1997 to 2006	1.011 (0.984, 1.038)
Botswana	1942 to 1951	0.962 (0.881, 1.051)
Botswana	1947 to 1956	0.982 (0.924, 1.043)
Botswana	1952 to 1961	0.990 (0.942, 1.040)
Botswana	1957 to 1966	1.001 (0.959, 1.044)
Botswana	1962 to 1971	1.008 (0.970, 1.047)
Botswana	1967 to 1976	1.009 (0.975, 1.045)
Botswana	1972 to 1981	1.015 (0.982, 1.049)
Botswana	1977 to 1986	1.011 (0.979, 1.045)
Botswana	1982 to 1991	1.000 (1.000, 1.000)
Botswana	1987 to 1996	0.800 (0.767, 0.834)
Botswana	1992 to 2001	0.750 (0.709, 0.792)
Botswana	1997 to 2006	0.621 (0.568, 0.679)
Brazil	1942 to 1951	0.880 (0.860, 0.900)
Brazil	1947 to 1956	0.897 (0.882, 0.912)
Brazil	1952 to 1961	0.913 (0.900, 0.926)
Brazil	1957 to 1966	0.928 (0.917, 0.940)
Brazil	1962 to 1971	0.944 (0.933, 0.955)
Brazil	1967 to 1976	0.959 (0.949, 0.970)
Brazil	1972 to 1981	0.977 (0.967, 0.988)
Brazil	1977 to 1986	0.988 (0.977, 0.999)
Brazil	1982 to 1991	1.000 (1.000, 1.000)
Brazil	1987 to 1996	1.003 (0.990, 1.017)

Brazil	1992 to 2001	0.932 (0.916, 0.949)
Brazil	1997 to 2006	0.840 (0.815, 0.866)
Brunei Darussalam	1942 to 1951	1.040 (0.927, 1.168)
Brunei Darussalam	1947 to 1956	1.084 (1.004, 1.172)
Brunei Darussalam	1952 to 1961	1.130 (1.061, 1.204)
Brunei Darussalam	1957 to 1966	1.165 (1.103, 1.231)
Brunei Darussalam	1962 to 1971	1.199 (1.140, 1.261)
Brunei Darussalam	1967 to 1976	1.237 (1.179, 1.297)
Brunei Darussalam	1972 to 1981	1.287 (1.229, 1.349)
Brunei Darussalam	1977 to 1986	1.078 (1.027, 1.132)
Brunei Darussalam	1982 to 1991	1.000 (1.000, 1.000)
Brunei Darussalam	1987 to 1996	0.988 (0.931, 1.048)
Brunei Darussalam	1992 to 2001	0.982 (0.911, 1.060)
Brunei Darussalam	1997 to 2006	0.962 (0.855, 1.084)
Bulgaria	1942 to 1951	0.973 (0.932, 1.016)
Bulgaria	1947 to 1956	0.974 (0.941, 1.008)
Bulgaria	1952 to 1961	0.977 (0.946, 1.008)
Bulgaria	1957 to 1966	0.986 (0.957, 1.016)
Bulgaria	1962 to 1971	0.993 (0.965, 1.021)
Bulgaria	1967 to 1976	0.996 (0.969, 1.023)
Bulgaria	1972 to 1981	1.006 (0.979, 1.033)
Bulgaria	1977 to 1986	1.012 (0.984, 1.041)
Bulgaria	1982 to 1991	1.000 (1.000, 1.000)
Bulgaria	1987 to 1996	0.853 (0.821, 0.886)

Bulgaria	1992 to 2001	0.768 (0.726, 0.812)
Bulgaria	1997 to 2006	0.706 (0.646, 0.772)
Burkina Faso	1942 to 1951	1.122 (1.107, 1.137)
Burkina Faso	1947 to 1956	1.108 (1.098, 1.118)
Burkina Faso	1952 to 1961	1.095 (1.087, 1.103)
Burkina Faso	1957 to 1966	1.082 (1.076, 1.089)
Burkina Faso	1962 to 1971	1.068 (1.062, 1.073)
Burkina Faso	1967 to 1976	1.053 (1.048, 1.058)
Burkina Faso	1972 to 1981	1.038 (1.034, 1.043)
Burkina Faso	1977 to 1986	1.021 (1.016, 1.025)
Burkina Faso	1982 to 1991	1.000 (1.000, 1.000)
Burkina Faso	1987 to 1996	0.975 (0.970, 0.979)
Burkina Faso	1992 to 2001	0.947 (0.942, 0.952)
Burkina Faso	1997 to 2006	0.818 (0.812, 0.825)
Burundi	1942 to 1951	1.006 (0.992, 1.020)
Burundi	1947 to 1956	1.004 (0.995, 1.014)
Burundi	1952 to 1961	1.006 (0.998, 1.013)
Burundi	1957 to 1966	1.005 (0.998, 1.012)
Burundi	1962 to 1971	1.006 (1.000, 1.012)
Burundi	1967 to 1976	1.006 (1.000, 1.012)
Burundi	1972 to 1981	1.005 (1.000, 1.010)
Burundi	1977 to 1986	1.004 (0.999, 1.009)
Burundi	1982 to 1991	1.000 (1.000, 1.000)
Burundi	1987 to 1996	0.990 (0.984, 0.995)

Burundi	1992 to 2001	0.969 (0.962, 0.975)
Burundi	1997 to 2006	0.741 (0.733, 0.748)
Cabo Verde	1942 to 1951	1.001 (0.949, 1.055)
Cabo Verde	1947 to 1956	1.002 (0.969, 1.038)
Cabo Verde	1952 to 1961	1.006 (0.979, 1.033)
Cabo Verde	1957 to 1966	1.009 (0.987, 1.033)
Cabo Verde	1962 to 1971	1.011 (0.990, 1.032)
Cabo Verde	1967 to 1976	1.012 (0.993, 1.032)
Cabo Verde	1972 to 1981	1.013 (0.995, 1.032)
Cabo Verde	1977 to 1986	1.008 (0.990, 1.026)
Cabo Verde	1982 to 1991	1.000 (1.000, 1.000)
Cabo Verde	1987 to 1996	0.986 (0.966, 1.007)
Cabo Verde	1992 to 2001	0.958 (0.933, 0.983)
Cabo Verde	1997 to 2006	0.653 (0.625, 0.682)
Cambodia	1942 to 1951	1.195 (1.182, 1.208)
Cambodia	1947 to 1956	1.179 (1.170, 1.188)
Cambodia	1952 to 1961	1.165 (1.157, 1.172)
Cambodia	1957 to 1966	1.147 (1.141, 1.153)
Cambodia	1962 to 1971	1.125 (1.120, 1.131)
Cambodia	1967 to 1976	1.099 (1.094, 1.104)
Cambodia	1972 to 1981	1.071 (1.066, 1.076)
Cambodia	1977 to 1986	1.036 (1.031, 1.040)
Cambodia	1982 to 1991	1.000 (1.000, 1.000)
Cambodia	1987 to 1996	0.960 (0.955, 0.965)

Cambodia	1992 to 2001	0.895 (0.889, 0.900)
Cambodia	1997 to 2006	0.601 (0.595, 0.607)
Cameroon	1942 to 1951	1.040 (1.023, 1.058)
Cameroon	1947 to 1956	1.040 (1.028, 1.053)
Cameroon	1952 to 1961	1.039 (1.029, 1.049)
Cameroon	1957 to 1966	1.039 (1.031, 1.047)
Cameroon	1962 to 1971	1.035 (1.028, 1.042)
Cameroon	1967 to 1976	1.032 (1.026, 1.038)
Cameroon	1972 to 1981	1.027 (1.022, 1.033)
Cameroon	1977 to 1986	1.014 (1.009, 1.020)
Cameroon	1982 to 1991	1.000 (1.000, 1.000)
Cameroon	1987 to 1996	0.978 (0.972, 0.984)
Cameroon	1992 to 2001	0.943 (0.936, 0.950)
Cameroon	1997 to 2006	0.769 (0.761, 0.777)
Canada	1942 to 1951	0.956 (0.936, 0.977)
Canada	1947 to 1956	0.981 (0.965, 0.998)
Canada	1952 to 1961	0.993 (0.979, 1.008)
Canada	1957 to 1966	0.994 (0.981, 1.007)
Canada	1962 to 1971	0.992 (0.979, 1.004)
Canada	1967 to 1976	0.993 (0.981, 1.005)
Canada	1972 to 1981	0.995 (0.983, 1.007)
Canada	1977 to 1986	0.998 (0.986, 1.010)
Canada	1982 to 1991	1.000 (1.000, 1.000)
Canada	1987 to 1996	0.993 (0.979, 1.007)

Canada	1992 to 2001	0.963 (0.945, 0.981)
Canada	1997 to 2006	0.934 (0.907, 0.960)
Central African Republic	1942 to 1951	1.028 (1.010, 1.047)
Central African Republic	1947 to 1956	1.028 (1.015, 1.041)
Central African Republic	1952 to 1961	1.029 (1.019, 1.039)
Central African Republic	1957 to 1966	1.029 (1.020, 1.037)
Central African Republic	1962 to 1971	1.027 (1.019, 1.034)
Central African Republic	1967 to 1976	1.023 (1.016, 1.030)
Central African Republic	1972 to 1981	1.019 (1.013, 1.026)
Central African Republic	1977 to 1986	1.012 (1.005, 1.018)
Central African Republic	1982 to 1991	1.000 (1.000, 1.000)
Central African Republic	1987 to 1996	0.984 (0.978, 0.991)
Central African Republic	1992 to 2001	0.962 (0.954, 0.970)
Central African Republic	1997 to 2006	0.935 (0.925, 0.946)
Chad	1942 to 1951	1.068 (1.051, 1.086)
Chad	1947 to 1956	1.060 (1.048, 1.072)
Chad	1952 to 1961	1.056 (1.046, 1.065)
Chad	1957 to 1966	1.051 (1.043, 1.059)
Chad	1962 to 1971	1.044 (1.037, 1.051)
Chad	1967 to 1976	1.036 (1.029, 1.042)
Chad	1972 to 1981	1.028 (1.022, 1.034)
Chad	1977 to 1986	1.015 (1.010, 1.021)
Chad	1982 to 1991	1.000 (1.000, 1.000)
Chad	1987 to 1996	0.981 (0.975, 0.987)

Chad	1992 to 2001	0.959 (0.953, 0.966)
Chad	1997 to 2006	0.939 (0.930, 0.947)
Chile	1942 to 1951	0.751 (0.741, 0.762)
Chile	1947 to 1956	0.780 (0.772, 0.788)
Chile	1952 to 1961	0.809 (0.802, 0.816)
Chile	1957 to 1966	0.839 (0.832, 0.845)
Chile	1962 to 1971	0.869 (0.863, 0.875)
Chile	1967 to 1976	0.901 (0.895, 0.907)
Chile	1972 to 1981	0.935 (0.929, 0.942)
Chile	1977 to 1986	0.968 (0.961, 0.975)
Chile	1982 to 1991	1.000 (1.000, 1.000)
Chile	1987 to 1996	1.026 (1.018, 1.035)
Chile	1992 to 2001	1.047 (1.035, 1.058)
Chile	1997 to 2006	1.024 (1.005, 1.043)
China	1942 to 1951	1.116 (1.043, 1.194)
China	1947 to 1956	1.119 (1.065, 1.175)
China	1952 to 1961	1.114 (1.066, 1.164)
China	1957 to 1966	1.100 (1.058, 1.144)
China	1962 to 1971	1.089 (1.051, 1.128)
China	1967 to 1976	1.068 (1.032, 1.105)
China	1972 to 1981	1.057 (1.020, 1.094)
China	1977 to 1986	1.031 (0.994, 1.069)
China	1982 to 1991	1.000 (1.000, 1.000)
China	1987 to 1996	0.947 (0.905, 0.990)

China	1992 to 2001	0.746 (0.697, 0.799)
China	1997 to 2006	0.450 (0.395, 0.511)
Colombia	1942 to 1951	0.918 (0.863, 0.976)
Colombia	1947 to 1956	0.937 (0.896, 0.979)
Colombia	1952 to 1961	0.952 (0.918, 0.988)
Colombia	1957 to 1966	0.969 (0.938, 1.000)
Colombia	1962 to 1971	0.981 (0.953, 1.011)
Colombia	1967 to 1976	0.988 (0.960, 1.016)
Colombia	1972 to 1981	1.000 (0.974, 1.028)
Colombia	1977 to 1986	1.004 (0.977, 1.032)
Colombia	1982 to 1991	1.000 (1.000, 1.000)
Colombia	1987 to 1996	0.823 (0.795, 0.851)
Colombia	1992 to 2001	0.718 (0.687, 0.751)
Colombia	1997 to 2006	0.591 (0.551, 0.635)
Comoros	1942 to 1951	0.993 (0.953, 1.036)
Comoros	1947 to 1956	0.998 (0.968, 1.029)
Comoros	1952 to 1961	1.001 (0.976, 1.027)
Comoros	1957 to 1966	1.007 (0.985, 1.029)
Comoros	1962 to 1971	1.008 (0.989, 1.028)
Comoros	1967 to 1976	1.007 (0.989, 1.026)
Comoros	1972 to 1981	1.009 (0.991, 1.026)
Comoros	1977 to 1986	1.006 (0.989, 1.024)
Comoros	1982 to 1991	1.000 (1.000, 1.000)
Comoros	1987 to 1996	0.989 (0.969, 1.010)

Comoros	1992 to 2001	0.972 (0.948, 0.997)
Comoros	1997 to 2006	0.710 (0.681, 0.740)
Congo	1942 to 1951	1.075 (1.054, 1.096)
Congo	1947 to 1956	1.065 (1.051, 1.080)
Congo	1952 to 1961	1.063 (1.052, 1.074)
Congo	1957 to 1966	1.059 (1.050, 1.069)
Congo	1962 to 1971	1.053 (1.045, 1.061)
Congo	1967 to 1976	1.044 (1.037, 1.052)
Congo	1972 to 1981	1.034 (1.028, 1.041)
Congo	1977 to 1986	1.020 (1.013, 1.027)
Congo	1982 to 1991	1.000 (1.000, 1.000)
Congo	1987 to 1996	0.973 (0.965, 0.980)
Congo	1992 to 2001	0.934 (0.926, 0.943)
Congo	1997 to 2006	0.887 (0.876, 0.898)
Cook Islands	1942 to 1951	1.123 (0.900, 1.402)
Cook Islands	1947 to 1956	1.131 (0.958, 1.335)
Cook Islands	1952 to 1961	1.144 (0.988, 1.326)
Cook Islands	1957 to 1966	1.147 (1.005, 1.310)
Cook Islands	1962 to 1971	1.147 (1.012, 1.299)
Cook Islands	1967 to 1976	1.144 (1.014, 1.290)
Cook Islands	1972 to 1981	1.147 (1.017, 1.293)
Cook Islands	1977 to 1986	1.134 (1.000, 1.285)
Cook Islands	1982 to 1991	1.000 (1.000, 1.000)
Cook Islands	1987 to 1996	0.918 (0.781, 1.079)

Cook Islands	1992 to 2001	0.824 (0.667, 1.018)
Cook Islands	1997 to 2006	0.734 (0.522, 1.033)
Costa Rica	1942 to 1951	0.872 (0.847, 0.898)
Costa Rica	1947 to 1956	0.892 (0.874, 0.911)
Costa Rica	1952 to 1961	0.910 (0.894, 0.926)
Costa Rica	1957 to 1966	0.927 (0.913, 0.941)
Costa Rica	1962 to 1971	0.944 (0.930, 0.957)
Costa Rica	1967 to 1976	0.957 (0.944, 0.970)
Costa Rica	1972 to 1981	0.973 (0.961, 0.986)
Costa Rica	1977 to 1986	0.988 (0.975, 1.001)
Costa Rica	1982 to 1991	1.000 (1.000, 1.000)
Costa Rica	1987 to 1996	1.008 (0.993, 1.024)
Costa Rica	1992 to 2001	0.962 (0.942, 0.982)
Costa Rica	1997 to 2006	0.911 (0.881, 0.941)
Côte d'Ivoire	1942 to 1951	1.026 (1.013, 1.040)
Côte d'Ivoire	1947 to 1956	1.028 (1.019, 1.038)
Côte d'Ivoire	1952 to 1961	1.028 (1.021, 1.035)
Côte d'Ivoire	1957 to 1966	1.024 (1.018, 1.030)
Côte d'Ivoire	1962 to 1971	1.020 (1.015, 1.025)
Côte d'Ivoire	1967 to 1976	1.018 (1.014, 1.023)
Côte d'Ivoire	1972 to 1981	1.015 (1.010, 1.019)
Côte d'Ivoire	1977 to 1986	1.007 (1.002, 1.011)
Côte d'Ivoire	1982 to 1991	1.000 (1.000, 1.000)
Côte d'Ivoire	1987 to 1996	0.989 (0.984, 0.994)

Côte d'Ivoire	1992 to 2001	0.957 (0.951, 0.963)
Côte d'Ivoire	1997 to 2006	0.657 (0.651, 0.663)
Croatia	1942 to 1951	0.863 (0.841, 0.885)
Croatia	1947 to 1956	0.873 (0.855, 0.890)
Croatia	1952 to 1961	0.893 (0.877, 0.909)
Croatia	1957 to 1966	0.910 (0.895, 0.926)
Croatia	1962 to 1971	0.930 (0.915, 0.945)
Croatia	1967 to 1976	0.948 (0.933, 0.963)
Croatia	1972 to 1981	0.967 (0.951, 0.982)
Croatia	1977 to 1986	0.981 (0.965, 0.997)
Croatia	1982 to 1991	1.000 (1.000, 1.000)
Croatia	1987 to 1996	0.997 (0.976, 1.019)
Croatia	1992 to 2001	0.903 (0.877, 0.930)
Croatia	1997 to 2006	0.817 (0.775, 0.860)
Cuba	1942 to 1951	0.878 (0.854, 0.902)
Cuba	1947 to 1956	0.892 (0.873, 0.912)
Cuba	1952 to 1961	0.912 (0.894, 0.931)
Cuba	1957 to 1966	0.932 (0.916, 0.948)
Cuba	1962 to 1971	0.950 (0.934, 0.966)
Cuba	1967 to 1976	0.969 (0.954, 0.985)
Cuba	1972 to 1981	0.992 (0.975, 1.009)
Cuba	1977 to 1986	1.013 (0.995, 1.031)
Cuba	1982 to 1991	1.000 (1.000, 1.000)
Cuba	1987 to 1996	0.992 (0.971, 1.014)

Cuba	1992 to 2001	0.993 (0.964, 1.023)
Cuba	1997 to 2006	0.989 (0.941, 1.038)
Cyprus	1942 to 1951	0.829 (0.796, 0.864)
Cyprus	1947 to 1956	0.858 (0.832, 0.885)
Cyprus	1952 to 1961	0.888 (0.865, 0.912)
Cyprus	1957 to 1966	0.917 (0.895, 0.939)
Cyprus	1962 to 1971	0.947 (0.926, 0.968)
Cyprus	1967 to 1976	0.972 (0.952, 0.993)
Cyprus	1972 to 1981	1.004 (0.984, 1.024)
Cyprus	1977 to 1986	1.026 (1.006, 1.047)
Cyprus	1982 to 1991	1.000 (1.000, 1.000)
Cyprus	1987 to 1996	0.956 (0.931, 0.981)
Cyprus	1992 to 2001	0.916 (0.882, 0.951)
Cyprus	1997 to 2006	0.881 (0.825, 0.940)
Czechia	1942 to 1951	0.848 (0.833, 0.863)
Czechia	1947 to 1956	0.870 (0.858, 0.882)
Czechia	1952 to 1961	0.890 (0.878, 0.902)
Czechia	1957 to 1966	0.909 (0.898, 0.920)
Czechia	1962 to 1971	0.925 (0.915, 0.936)
Czechia	1967 to 1976	0.944 (0.934, 0.955)
Czechia	1972 to 1981	0.961 (0.951, 0.971)
Czechia	1977 to 1986	0.978 (0.967, 0.989)
Czechia	1982 to 1991	1.000 (1.000, 1.000)
Czechia	1987 to 1996	1.018 (1.003, 1.032)

Czechia	1992 to 2001	1.004 (0.984, 1.025)
Czechia	1997 to 2006	0.867 (0.838, 0.898)
Democratic People's Republic of Korea	1942 to 1951	0.983 (0.969, 0.998)
Democratic People's Republic of Korea	1947 to 1956	0.990 (0.979, 1.001)
Democratic People's Republic of Korea	1952 to 1961	1.001 (0.991, 1.010)
Democratic People's Republic of Korea	1957 to 1966	1.010 (1.001, 1.018)
Democratic People's Republic of Korea	1962 to 1971	1.012 (1.004, 1.020)
Democratic People's Republic of Korea	1967 to 1976	1.013 (1.005, 1.020)
Democratic People's Republic of Korea	1972 to 1981	1.012 (1.005, 1.020)
Democratic People's Republic of Korea	1977 to 1986	1.009 (1.002, 1.017)
Democratic People's Republic of Korea	1982 to 1991	1.000 (1.000, 1.000)
Democratic People's Republic of Korea	1987 to 1996	0.986 (0.977, 0.994)
Democratic People's Republic of Korea	1992 to 2001	0.964 (0.954, 0.975)
Democratic People's Republic of Korea	1997 to 2006	0.577 (0.566, 0.589)
Democratic Republic of the Congo	1942 to 1951	1.052 (1.038, 1.066)
Democratic Republic of the Congo	1947 to 1956	1.043 (1.034, 1.052)
Democratic Republic of the Congo	1952 to 1961	1.039 (1.031, 1.046)
Democratic Republic of the Congo	1957 to 1966	1.035 (1.029, 1.041)
Democratic Republic of the Congo	1962 to 1971	1.031 (1.025, 1.036)
Democratic Republic of the Congo	1967 to 1976	1.026 (1.021, 1.031)
Democratic Republic of the Congo	1972 to 1981	1.019 (1.014, 1.023)
Democratic Republic of the Congo	1977 to 1986	1.011 (1.007, 1.016)
Democratic Republic of the Congo	1982 to 1991	1.000 (1.000, 1.000)
Democratic Republic of the Congo	1987 to 1996	0.981 (0.977, 0.986)

Democratic Republic of the Congo	1992 to 2001	0.950 (0.944, 0.955)
Democratic Republic of the Congo	1997 to 2006	0.906 (0.899, 0.913)
Denmark	1942 to 1951	0.757 (0.741, 0.774)
Denmark	1947 to 1956	0.796 (0.782, 0.810)
Denmark	1952 to 1961	0.818 (0.805, 0.831)
Denmark	1957 to 1966	0.845 (0.833, 0.858)
Denmark	1962 to 1971	0.873 (0.862, 0.885)
Denmark	1967 to 1976	0.902 (0.891, 0.914)
Denmark	1972 to 1981	0.935 (0.923, 0.948)
Denmark	1977 to 1986	0.969 (0.956, 0.982)
Denmark	1982 to 1991	1.000 (1.000, 1.000)
Denmark	1987 to 1996	1.021 (1.005, 1.038)
Denmark	1992 to 2001	1.042 (1.020, 1.063)
Denmark	1997 to 2006	1.063 (1.029, 1.098)
Djibouti	1942 to 1951	0.907 (0.867, 0.948)
Djibouti	1947 to 1956	0.921 (0.893, 0.951)
Djibouti	1952 to 1961	0.937 (0.914, 0.960)
Djibouti	1957 to 1966	0.952 (0.932, 0.972)
Djibouti	1962 to 1971	0.964 (0.947, 0.981)
Djibouti	1967 to 1976	0.975 (0.960, 0.991)
Djibouti	1972 to 1981	0.988 (0.973, 1.003)
Djibouti	1977 to 1986	0.996 (0.982, 1.011)
Djibouti	1982 to 1991	1.000 (1.000, 1.000)
Djibouti	1987 to 1996	0.999 (0.982, 1.017)

Djibouti	1992 to 2001	0.990 (0.968, 1.012)
Djibouti	1997 to 2006	0.961 (0.930, 0.993)
Dominica	1942 to 1951	0.921 (0.814, 1.043)
Dominica	1947 to 1956	0.932 (0.847, 1.025)
Dominica	1952 to 1961	0.942 (0.868, 1.023)
Dominica	1957 to 1966	0.951 (0.884, 1.024)
Dominica	1962 to 1971	0.960 (0.897, 1.028)
Dominica	1967 to 1976	0.969 (0.907, 1.036)
Dominica	1972 to 1981	0.982 (0.918, 1.050)
Dominica	1977 to 1986	0.991 (0.925, 1.061)
Dominica	1982 to 1991	1.000 (1.000, 1.000)
Dominica	1987 to 1996	1.008 (0.928, 1.094)
Dominica	1992 to 2001	1.006 (0.903, 1.120)
Dominica	1997 to 2006	0.992 (0.841, 1.170)
Dominican Republic	1942 to 1951	0.884 (0.873, 0.896)
Dominican Republic	1947 to 1956	0.905 (0.896, 0.913)
Dominican Republic	1952 to 1961	0.927 (0.920, 0.935)
Dominican Republic	1957 to 1966	0.946 (0.940, 0.953)
Dominican Republic	1962 to 1971	0.963 (0.957, 0.970)
Dominican Republic	1967 to 1976	0.978 (0.972, 0.984)
Dominican Republic	1972 to 1981	0.992 (0.985, 0.998)
Dominican Republic	1977 to 1986	0.998 (0.992, 1.004)
Dominican Republic	1982 to 1991	1.000 (1.000, 1.000)
Dominican Republic	1987 to 1996	0.971 (0.963, 0.978)

Dominican Republic	1992 to 2001	0.937 (0.928, 0.946)
Dominican Republic	1997 to 2006	0.886 (0.872, 0.899)
Ecuador	1942 to 1951	0.870 (0.861, 0.880)
Ecuador	1947 to 1956	0.890 (0.883, 0.898)
Ecuador	1952 to 1961	0.905 (0.898, 0.911)
Ecuador	1957 to 1966	0.918 (0.913, 0.924)
Ecuador	1962 to 1971	0.932 (0.927, 0.938)
Ecuador	1967 to 1976	0.949 (0.944, 0.954)
Ecuador	1972 to 1981	0.965 (0.960, 0.970)
Ecuador	1977 to 1986	0.983 (0.978, 0.988)
Ecuador	1982 to 1991	1.000 (1.000, 1.000)
Ecuador	1987 to 1996	1.020 (1.014, 1.026)
Ecuador	1992 to 2001	1.025 (1.017, 1.033)
Ecuador	1997 to 2006	0.984 (0.970, 0.998)
Egypt	1942 to 1951	0.977 (0.918, 1.039)
Egypt	1947 to 1956	0.975 (0.932, 1.019)
Egypt	1952 to 1961	0.983 (0.947, 1.022)
Egypt	1957 to 1966	0.991 (0.958, 1.025)
Egypt	1962 to 1971	0.988 (0.959, 1.019)
Egypt	1967 to 1976	0.981 (0.954, 1.010)
Egypt	1972 to 1981	0.987 (0.960, 1.014)
Egypt	1977 to 1986	0.994 (0.967, 1.021)
Egypt	1982 to 1991	1.000 (1.000, 1.000)
Egypt	1987 to 1996	0.931 (0.901, 0.962)

Egypt	1992 to 2001	0.889 (0.852, 0.927)
Egypt	1997 to 2006	0.842 (0.789, 0.899)
El Salvador	1942 to 1951	0.867 (0.838, 0.897)
El Salvador	1947 to 1956	0.886 (0.864, 0.908)
El Salvador	1952 to 1961	0.905 (0.886, 0.924)
El Salvador	1957 to 1966	0.924 (0.907, 0.941)
El Salvador	1962 to 1971	0.942 (0.926, 0.958)
El Salvador	1967 to 1976	0.957 (0.942, 0.972)
El Salvador	1972 to 1981	0.976 (0.961, 0.991)
El Salvador	1977 to 1986	0.989 (0.973, 1.005)
El Salvador	1982 to 1991	1.000 (1.000, 1.000)
El Salvador	1987 to 1996	1.009 (0.990, 1.027)
El Salvador	1992 to 2001	0.946 (0.924, 0.969)
El Salvador	1997 to 2006	0.860 (0.828, 0.894)
Equatorial Guinea	1942 to 1951	1.117 (1.057, 1.180)
Equatorial Guinea	1947 to 1956	1.112 (1.070, 1.155)
Equatorial Guinea	1952 to 1961	1.110 (1.076, 1.146)
Equatorial Guinea	1957 to 1966	1.104 (1.075, 1.134)
Equatorial Guinea	1962 to 1971	1.091 (1.066, 1.117)
Equatorial Guinea	1967 to 1976	1.075 (1.052, 1.097)
Equatorial Guinea	1972 to 1981	1.060 (1.039, 1.081)
Equatorial Guinea	1977 to 1986	1.035 (1.015, 1.056)
Equatorial Guinea	1982 to 1991	1.000 (1.000, 1.000)
Equatorial Guinea	1987 to 1996	0.957 (0.936, 0.978)

Equatorial Guinea	1992 to 2001	0.902 (0.878, 0.926)
Equatorial Guinea	1997 to 2006	0.833 (0.802, 0.865)
Eritrea	1942 to 1951	1.035 (1.017, 1.053)
Eritrea	1947 to 1956	1.034 (1.022, 1.047)
Eritrea	1952 to 1961	1.033 (1.023, 1.044)
Eritrea	1957 to 1966	1.032 (1.023, 1.040)
Eritrea	1962 to 1971	1.028 (1.020, 1.036)
Eritrea	1967 to 1976	1.023 (1.016, 1.030)
Eritrea	1972 to 1981	1.019 (1.013, 1.026)
Eritrea	1977 to 1986	1.011 (1.004, 1.018)
Eritrea	1982 to 1991	1.000 (1.000, 1.000)
Eritrea	1987 to 1996	0.988 (0.980, 0.995)
Eritrea	1992 to 2001	0.966 (0.957, 0.974)
Eritrea	1997 to 2006	0.570 (0.562, 0.579)
Estonia	1942 to 1951	0.954 (0.925, 0.984)
Estonia	1947 to 1956	0.958 (0.936, 0.982)
Estonia	1952 to 1961	0.963 (0.943, 0.985)
Estonia	1957 to 1966	0.970 (0.951, 0.990)
Estonia	1962 to 1971	0.978 (0.960, 0.997)
Estonia	1967 to 1976	0.986 (0.968, 1.004)
Estonia	1972 to 1981	0.995 (0.977, 1.013)
Estonia	1977 to 1986	1.000 (0.982, 1.020)
Estonia	1982 to 1991	1.000 (1.000, 1.000)
Estonia	1987 to 1996	0.997 (0.973, 1.021)

Estonia	1992 to 2001	0.851 (0.821, 0.882)
Estonia	1997 to 2006	0.777 (0.735, 0.821)
Eswatini	1942 to 1951	1.019 (0.966, 1.075)
Eswatini	1947 to 1956	1.026 (0.988, 1.065)
Eswatini	1952 to 1961	1.027 (0.996, 1.059)
Eswatini	1957 to 1966	1.027 (1.000, 1.054)
Eswatini	1962 to 1971	1.026 (1.002, 1.051)
Eswatini	1967 to 1976	1.022 (1.000, 1.044)
Eswatini	1972 to 1981	1.019 (0.999, 1.041)
Eswatini	1977 to 1986	1.012 (0.991, 1.033)
Eswatini	1982 to 1991	1.000 (1.000, 1.000)
Eswatini	1987 to 1996	0.968 (0.945, 0.991)
Eswatini	1992 to 2001	0.731 (0.707, 0.755)
Eswatini	1997 to 2006	0.562 (0.532, 0.593)
Ethiopia	1942 to 1951	1.088 (1.063, 1.115)
Ethiopia	1947 to 1956	1.073 (1.056, 1.091)
Ethiopia	1952 to 1961	1.066 (1.052, 1.080)
Ethiopia	1957 to 1966	1.057 (1.045, 1.069)
Ethiopia	1962 to 1971	1.048 (1.038, 1.059)
Ethiopia	1967 to 1976	1.039 (1.029, 1.048)
Ethiopia	1972 to 1981	1.031 (1.022, 1.040)
Ethiopia	1977 to 1986	1.018 (1.010, 1.027)
Ethiopia	1982 to 1991	1.000 (1.000, 1.000)
Ethiopia	1987 to 1996	0.972 (0.963, 0.982)

Ethiopia	1992 to 2001	0.932 (0.922, 0.943)
Ethiopia	1997 to 2006	0.882 (0.868, 0.896)
Fiji	1942 to 1951	0.967 (0.874, 1.071)
Fiji	1947 to 1956	0.992 (0.919, 1.070)
Fiji	1952 to 1961	1.019 (0.954, 1.089)
Fiji	1957 to 1966	1.043 (0.983, 1.107)
Fiji	1962 to 1971	1.062 (1.005, 1.123)
Fiji	1967 to 1976	1.078 (1.022, 1.137)
Fiji	1972 to 1981	1.106 (1.050, 1.166)
Fiji	1977 to 1986	1.117 (1.059, 1.178)
Fiji	1982 to 1991	1.000 (1.000, 1.000)
Fiji	1987 to 1996	0.919 (0.858, 0.984)
Fiji	1992 to 2001	0.851 (0.776, 0.933)
Fiji	1997 to 2006	0.783 (0.671, 0.913)
Finland	1942 to 1951	0.806 (0.790, 0.822)
Finland	1947 to 1956	0.834 (0.820, 0.847)
Finland	1952 to 1961	0.858 (0.845, 0.871)
Finland	1957 to 1966	0.879 (0.867, 0.891)
Finland	1962 to 1971	0.899 (0.888, 0.911)
Finland	1967 to 1976	0.921 (0.909, 0.933)
Finland	1972 to 1981	0.949 (0.937, 0.961)
Finland	1977 to 1986	0.977 (0.964, 0.989)
Finland	1982 to 1991	1.000 (1.000, 1.000)
Finland	1987 to 1996	1.016 (1.001, 1.032)

Finland	1992 to 2001	1.027 (1.006, 1.047)
Finland	1997 to 2006	1.039 (1.008, 1.071)
France	1942 to 1951	0.707 (0.685, 0.731)
France	1947 to 1956	0.742 (0.724, 0.761)
France	1952 to 1961	0.774 (0.757, 0.792)
France	1957 to 1966	0.802 (0.786, 0.819)
France	1962 to 1971	0.834 (0.818, 0.850)
France	1967 to 1976	0.870 (0.854, 0.886)
France	1972 to 1981	0.910 (0.893, 0.927)
France	1977 to 1986	0.952 (0.935, 0.971)
France	1982 to 1991	1.000 (1.000, 1.000)
France	1987 to 1996	1.015 (0.992, 1.039)
France	1992 to 2001	1.022 (0.992, 1.053)
France	1997 to 2006	1.012 (0.967, 1.058)
Gabon	1942 to 1951	1.021 (0.984, 1.060)
Gabon	1947 to 1956	1.035 (1.009, 1.062)
Gabon	1952 to 1961	1.041 (1.020, 1.063)
Gabon	1957 to 1966	1.040 (1.022, 1.059)
Gabon	1962 to 1971	1.035 (1.019, 1.051)
Gabon	1967 to 1976	1.030 (1.015, 1.044)
Gabon	1972 to 1981	1.027 (1.013, 1.041)
Gabon	1977 to 1986	1.016 (1.002, 1.030)
Gabon	1982 to 1991	1.000 (1.000, 1.000)
Gabon	1987 to 1996	0.978 (0.963, 0.993)

Gabon	1992 to 2001	0.942 (0.925, 0.959)
Gabon	1997 to 2006	0.783 (0.762, 0.804)
Gambia	1942 to 1951	1.220 (1.031, 1.442)
Gambia	1947 to 1956	1.221 (1.090, 1.368)
Gambia	1952 to 1961	1.238 (1.131, 1.356)
Gambia	1957 to 1966	1.239 (1.148, 1.337)
Gambia	1962 to 1971	1.239 (1.159, 1.325)
Gambia	1967 to 1976	1.238 (1.164, 1.317)
Gambia	1972 to 1981	1.250 (1.179, 1.326)
Gambia	1977 to 1986	1.241 (1.170, 1.317)
Gambia	1982 to 1991	1.000 (1.000, 1.000)
Gambia	1987 to 1996	0.949 (0.885, 1.017)
Gambia	1992 to 2001	0.861 (0.790, 0.938)
Gambia	1997 to 2006	0.690 (0.606, 0.785)
Georgia	1942 to 1951	0.993 (0.980, 1.006)
Georgia	1947 to 1956	0.991 (0.981, 1.001)
Georgia	1952 to 1961	0.996 (0.987, 1.004)
Georgia	1957 to 1966	0.999 (0.991, 1.007)
Georgia	1962 to 1971	1.000 (0.992, 1.008)
Georgia	1967 to 1976	1.001 (0.994, 1.009)
Georgia	1972 to 1981	1.004 (0.996, 1.011)
Georgia	1977 to 1986	1.004 (0.996, 1.012)
Georgia	1982 to 1991	1.000 (1.000, 1.000)
Georgia	1987 to 1996	0.991 (0.982, 1.001)

Georgia	1992 to 2001	0.950 (0.937, 0.963)
Georgia	1997 to 2006	0.864 (0.845, 0.884)
Germany	1942 to 1951	0.688 (0.676, 0.701)
Germany	1947 to 1956	0.724 (0.714, 0.734)
Germany	1952 to 1961	0.756 (0.747, 0.765)
Germany	1957 to 1966	0.791 (0.782, 0.800)
Germany	1962 to 1971	0.829 (0.820, 0.837)
Germany	1967 to 1976	0.868 (0.859, 0.877)
Germany	1972 to 1981	0.916 (0.907, 0.926)
Germany	1977 to 1986	0.961 (0.951, 0.971)
Germany	1982 to 1991	1.000 (1.000, 1.000)
Germany	1987 to 1996	1.011 (0.998, 1.024)
Germany	1992 to 2001	1.003 (0.986, 1.021)
Germany	1997 to 2006	1.021 (0.992, 1.050)
Ghana	1942 to 1951	1.056 (1.024, 1.088)
Ghana	1947 to 1956	1.061 (1.040, 1.084)
Ghana	1952 to 1961	1.065 (1.048, 1.083)
Ghana	1957 to 1966	1.064 (1.049, 1.079)
Ghana	1962 to 1971	1.056 (1.043, 1.069)
Ghana	1967 to 1976	1.052 (1.040, 1.064)
Ghana	1972 to 1981	1.043 (1.032, 1.054)
Ghana	1977 to 1986	1.024 (1.013, 1.035)
Ghana	1982 to 1991	1.000 (1.000, 1.000)
Ghana	1987 to 1996	0.971 (0.959, 0.982)

Ghana	1992 to 2001	0.883 (0.871, 0.896)
Ghana	1997 to 2006	0.518 (0.506, 0.531)
Greece	1942 to 1951	0.770 (0.753, 0.787)
Greece	1947 to 1956	0.809 (0.795, 0.824)
Greece	1952 to 1961	0.846 (0.833, 0.860)
Greece	1957 to 1966	0.877 (0.864, 0.891)
Greece	1962 to 1971	0.905 (0.892, 0.918)
Greece	1967 to 1976	0.929 (0.917, 0.942)
Greece	1972 to 1981	0.958 (0.945, 0.971)
Greece	1977 to 1986	0.982 (0.968, 0.996)
Greece	1982 to 1991	1.000 (1.000, 1.000)
Greece	1987 to 1996	0.998 (0.979, 1.016)
Greece	1992 to 2001	0.848 (0.827, 0.870)
Greece	1997 to 2006	0.766 (0.735, 0.798)
Greenland	1942 to 1951	0.899 (0.742, 1.089)
Greenland	1947 to 1956	0.926 (0.802, 1.070)
Greenland	1952 to 1961	0.947 (0.840, 1.068)
Greenland	1957 to 1966	0.958 (0.863, 1.063)
Greenland	1962 to 1971	0.964 (0.874, 1.062)
Greenland	1967 to 1976	0.972 (0.881, 1.073)
Greenland	1972 to 1981	0.981 (0.888, 1.085)
Greenland	1977 to 1986	0.991 (0.897, 1.095)
Greenland	1982 to 1991	1.000 (1.000, 1.000)
Greenland	1987 to 1996	1.007 (0.899, 1.129)

Greenland	1992 to 2001	1.017 (0.879, 1.178)
Greenland	1997 to 2006	1.034 (0.830, 1.288)
Grenada	1942 to 1951	0.920 (0.822, 1.030)
Grenada	1947 to 1956	0.928 (0.855, 1.009)
Grenada	1952 to 1961	0.939 (0.875, 1.008)
Grenada	1957 to 1966	0.952 (0.894, 1.014)
Grenada	1962 to 1971	0.963 (0.909, 1.021)
Grenada	1967 to 1976	0.973 (0.921, 1.027)
Grenada	1972 to 1981	0.984 (0.933, 1.038)
Grenada	1977 to 1986	0.992 (0.939, 1.048)
Grenada	1982 to 1991	1.000 (1.000, 1.000)
Grenada	1987 to 1996	1.005 (0.942, 1.073)
Grenada	1992 to 2001	0.947 (0.868, 1.032)
Grenada	1997 to 2006	0.872 (0.754, 1.008)
Guam	1942 to 1951	0.983 (0.910, 1.061)
Guam	1947 to 1956	1.003 (0.946, 1.063)
Guam	1952 to 1961	1.027 (0.975, 1.082)
Guam	1957 to 1966	1.047 (0.998, 1.098)
Guam	1962 to 1971	1.060 (1.013, 1.109)
Guam	1967 to 1976	1.070 (1.025, 1.117)
Guam	1972 to 1981	1.082 (1.036, 1.130)
Guam	1977 to 1986	1.091 (1.042, 1.141)
Guam	1982 to 1991	1.000 (1.000, 1.000)
Guam	1987 to 1996	0.926 (0.876, 0.980)

Guam	1992 to 2001	0.813 (0.755, 0.875)
Guam	1997 to 2006	0.767 (0.683, 0.861)
Guatemala	1942 to 1951	0.930 (0.900, 0.961)
Guatemala	1947 to 1956	0.949 (0.927, 0.971)
Guatemala	1952 to 1961	0.964 (0.946, 0.983)
Guatemala	1957 to 1966	0.977 (0.960, 0.993)
Guatemala	1962 to 1971	0.985 (0.970, 1.000)
Guatemala	1967 to 1976	0.992 (0.979, 1.006)
Guatemala	1972 to 1981	1.002 (0.990, 1.016)
Guatemala	1977 to 1986	1.004 (0.992, 1.018)
Guatemala	1982 to 1991	1.000 (1.000, 1.000)
Guatemala	1987 to 1996	0.991 (0.976, 1.006)
Guatemala	1992 to 2001	0.974 (0.956, 0.992)
Guatemala	1997 to 2006	0.877 (0.853, 0.902)
Guinea	1942 to 1951	1.054 (1.038, 1.070)
Guinea	1947 to 1956	1.050 (1.039, 1.062)
Guinea	1952 to 1961	1.046 (1.037, 1.055)
Guinea	1957 to 1966	1.043 (1.035, 1.051)
Guinea	1962 to 1971	1.036 (1.029, 1.043)
Guinea	1967 to 1976	1.030 (1.024, 1.037)
Guinea	1972 to 1981	1.024 (1.018, 1.030)
Guinea	1977 to 1986	1.013 (1.007, 1.019)
Guinea	1982 to 1991	1.000 (1.000, 1.000)
Guinea	1987 to 1996	0.981 (0.974, 0.987)

Guinea	1992 to 2001	0.961 (0.954, 0.969)
Guinea	1997 to 2006	0.928 (0.919, 0.938)
Guinea-Bissau	1942 to 1951	1.042 (1.013, 1.072)
Guinea-Bissau	1947 to 1956	1.035 (1.015, 1.056)
Guinea-Bissau	1952 to 1961	1.030 (1.014, 1.047)
Guinea-Bissau	1957 to 1966	1.027 (1.013, 1.041)
Guinea-Bissau	1962 to 1971	1.023 (1.010, 1.035)
Guinea-Bissau	1967 to 1976	1.019 (1.008, 1.030)
Guinea-Bissau	1972 to 1981	1.016 (1.006, 1.026)
Guinea-Bissau	1977 to 1986	1.008 (0.998, 1.018)
Guinea-Bissau	1982 to 1991	1.000 (1.000, 1.000)
Guinea-Bissau	1987 to 1996	0.991 (0.981, 1.002)
Guinea-Bissau	1992 to 2001	0.977 (0.964, 0.990)
Guinea-Bissau	1997 to 2006	0.946 (0.929, 0.963)
Guyana	1942 to 1951	0.883 (0.849, 0.919)
Guyana	1947 to 1956	0.900 (0.874, 0.926)
Guyana	1952 to 1961	0.914 (0.891, 0.937)
Guyana	1957 to 1966	0.929 (0.909, 0.950)
Guyana	1962 to 1971	0.943 (0.923, 0.963)
Guyana	1967 to 1976	0.956 (0.937, 0.975)
Guyana	1972 to 1981	0.971 (0.953, 0.991)
Guyana	1977 to 1986	0.986 (0.966, 1.007)
Guyana	1982 to 1991	1.000 (1.000, 1.000)
Guyana	1987 to 1996	1.008 (0.984, 1.032)

Guyana	1992 to 2001	1.006 (0.978, 1.036)
Guyana	1997 to 2006	0.902 (0.861, 0.945)
Haiti	1942 to 1951	0.972 (0.960, 0.984)
Haiti	1947 to 1956	0.979 (0.971, 0.988)
Haiti	1952 to 1961	0.986 (0.979, 0.993)
Haiti	1957 to 1966	0.990 (0.984, 0.996)
Haiti	1962 to 1971	0.992 (0.987, 0.998)
Haiti	1967 to 1976	0.996 (0.991, 1.001)
Haiti	1972 to 1981	0.999 (0.994, 1.004)
Haiti	1977 to 1986	1.001 (0.996, 1.006)
Haiti	1982 to 1991	1.000 (1.000, 1.000)
Haiti	1987 to 1996	0.996 (0.991, 1.002)
Haiti	1992 to 2001	0.985 (0.978, 0.993)
Haiti	1997 to 2006	0.965 (0.954, 0.976)
Honduras	1942 to 1951	0.926 (0.869, 0.986)
Honduras	1947 to 1956	0.939 (0.898, 0.981)
Honduras	1952 to 1961	0.954 (0.92, 0.989)
Honduras	1957 to 1966	0.966 (0.936, 0.996)
Honduras	1962 to 1971	0.975 (0.948, 1.003)
Honduras	1967 to 1976	0.983 (0.959, 1.009)
Honduras	1972 to 1981	0.995 (0.971, 1.019)
Honduras	1977 to 1986	0.999 (0.975, 1.023)
Honduras	1982 to 1991	1.000 (1.000, 1.000)
Honduras	1987 to 1996	0.997 (0.971, 1.025)

Honduras	1992 to 2001	0.904 (0.873, 0.936)
Honduras	1997 to 2006	0.781 (0.740, 0.825)
Hungary	1942 to 1951	0.864 (0.849, 0.880)
Hungary	1947 to 1956	0.882 (0.869, 0.895)
Hungary	1952 to 1961	0.895 (0.883, 0.907)
Hungary	1957 to 1966	0.910 (0.899, 0.922)
Hungary	1962 to 1971	0.925 (0.914, 0.936)
Hungary	1967 to 1976	0.942 (0.931, 0.953)
Hungary	1972 to 1981	0.960 (0.949, 0.971)
Hungary	1977 to 1986	0.981 (0.969, 0.993)
Hungary	1982 to 1991	1.000 (1.000, 1.000)
Hungary	1987 to 1996	1.018 (1.003, 1.033)
Hungary	1992 to 2001	1.010 (0.989, 1.031)
Hungary	1997 to 2006	1.030 (0.996, 1.067)
Iceland	1942 to 1951	0.811 (0.743, 0.886)
Iceland	1947 to 1956	0.836 (0.783, 0.893)
Iceland	1952 to 1961	0.860 (0.812, 0.911)
Iceland	1957 to 1966	0.885 (0.840, 0.932)
Iceland	1962 to 1971	0.908 (0.865, 0.953)
Iceland	1967 to 1976	0.931 (0.889, 0.975)
Iceland	1972 to 1981	0.955 (0.912, 1.000)
Iceland	1977 to 1986	0.979 (0.934, 1.025)
Iceland	1982 to 1991	1.000 (1.000, 1.000)
Iceland	1987 to 1996	1.021 (0.967, 1.079)

Iceland	1992 to 2001	1.041 (0.971, 1.117)
Iceland	1997 to 2006	1.110 (1.001, 1.231)
India	1942 to 1951	0.961 (0.939, 0.984)
India	1947 to 1956	0.970 (0.954, 0.987)
India	1952 to 1961	0.975 (0.961, 0.989)
India	1957 to 1966	0.976 (0.964, 0.988)
India	1962 to 1971	0.980 (0.969, 0.992)
India	1967 to 1976	0.987 (0.976, 0.997)
India	1972 to 1981	0.991 (0.981, 1.001)
India	1977 to 1986	0.994 (0.984, 1.005)
India	1982 to 1991	1.000 (1.000, 1.000)
India	1987 to 1996	0.995 (0.983, 1.007)
India	1992 to 2001	0.965 (0.951, 0.980)
India	1997 to 2006	0.916 (0.895, 0.937)
Indonesia	1942 to 1951	0.954 (0.920, 0.988)
Indonesia	1947 to 1956	0.969 (0.944, 0.994)
Indonesia	1952 to 1961	0.983 (0.963, 1.005)
Indonesia	1957 to 1966	0.997 (0.978, 1.016)
Indonesia	1962 to 1971	1.006 (0.989, 1.024)
Indonesia	1967 to 1976	1.008 (0.992, 1.025)
Indonesia	1972 to 1981	1.013 (0.997, 1.030)
Indonesia	1977 to 1986	1.009 (0.993, 1.026)
Indonesia	1982 to 1991	1.000 (1.000, 1.000)
Indonesia	1987 to 1996	0.919 (0.901, 0.938)

Indonesia	1992 to 2001	0.831 (0.809, 0.853)
Indonesia	1997 to 2006	0.746 (0.715, 0.778)
Iran (Islamic Republic of)	1942 to 1951	0.720 (0.690, 0.752)
Iran (Islamic Republic of)	1947 to 1956	0.753 (0.731, 0.776)
Iran (Islamic Republic of)	1952 to 1961	0.784 (0.765, 0.803)
Iran (Islamic Republic of)	1957 to 1966	0.814 (0.797, 0.831)
Iran (Islamic Republic of)	1962 to 1971	0.850 (0.834, 0.866)
Iran (Islamic Republic of)	1967 to 1976	0.890 (0.875, 0.905)
Iran (Islamic Republic of)	1972 to 1981	0.932 (0.917, 0.947)
Iran (Islamic Republic of)	1977 to 1986	0.967 (0.952, 0.983)
Iran (Islamic Republic of)	1982 to 1991	1.000 (1.000, 1.000)
Iran (Islamic Republic of)	1987 to 1996	0.995 (0.975, 1.016)
Iran (Islamic Republic of)	1992 to 2001	0.986 (0.956, 1.016)
Iran (Islamic Republic of)	1997 to 2006	0.990 (0.941, 1.041)
Iraq	1942 to 1951	0.781 (0.773, 0.789)
Iraq	1947 to 1956	0.803 (0.798, 0.809)
Iraq	1952 to 1961	0.831 (0.826, 0.836)
Iraq	1957 to 1966	0.858 (0.854, 0.862)
Iraq	1962 to 1971	0.884 (0.880, 0.888)
Iraq	1967 to 1976	0.913 (0.909, 0.917)
Iraq	1972 to 1981	0.943 (0.939, 0.947)
Iraq	1977 to 1986	0.972 (0.968, 0.975)
Iraq	1982 to 1991	1.000 (1.000, 1.000)
Iraq	1987 to 1996	1.018 (1.014, 1.023)

Iraq	1992 to 2001	1.039 (1.033, 1.045)
Iraq	1997 to 2006	1.067 (1.058, 1.075)
Ireland	1942 to 1951	0.786 (0.769, 0.803)
Ireland	1947 to 1956	0.810 (0.797, 0.824)
Ireland	1952 to 1961	0.840 (0.828, 0.852)
Ireland	1957 to 1966	0.865 (0.854, 0.877)
Ireland	1962 to 1971	0.894 (0.883, 0.905)
Ireland	1967 to 1976	0.918 (0.907, 0.929)
Ireland	1972 to 1981	0.945 (0.935, 0.956)
Ireland	1977 to 1986	0.971 (0.960, 0.982)
Ireland	1982 to 1991	1.000 (1.000, 1.000)
Ireland	1987 to 1996	1.024 (1.009, 1.039)
Ireland	1992 to 2001	1.045 (1.026, 1.064)
Ireland	1997 to 2006	1.064 (1.036, 1.092)
Israel	1942 to 1951	0.709 (0.695, 0.724)
Israel	1947 to 1956	0.754 (0.742, 0.765)
Israel	1952 to 1961	0.792 (0.781, 0.803)
Israel	1957 to 1966	0.830 (0.820, 0.841)
Israel	1962 to 1971	0.866 (0.857, 0.876)
Israel	1967 to 1976	0.901 (0.891, 0.910)
Israel	1972 to 1981	0.939 (0.930, 0.949)
Israel	1977 to 1986	0.971 (0.961, 0.981)
Israel	1982 to 1991	1.000 (1.000, 1.000)
Israel	1987 to 1996	0.991 (0.979, 1.004)

Israel	1992 to 2001	0.996 (0.980, 1.012)
Israel	1997 to 2006	1.028 (1.003, 1.053)
Italy	1942 to 1951	0.789 (0.742, 0.839)
Italy	1947 to 1956	0.826 (0.786, 0.869)
Italy	1952 to 1961	0.864 (0.826, 0.904)
Italy	1957 to 1966	0.903 (0.867, 0.941)
Italy	1962 to 1971	0.943 (0.907, 0.980)
Italy	1967 to 1976	0.981 (0.945, 1.018)
Italy	1972 to 1981	1.031 (0.993, 1.070)
Italy	1977 to 1986	1.001 (0.962, 1.041)
Italy	1982 to 1991	1.000 (1.000, 1.000)
Italy	1987 to 1996	0.979 (0.931, 1.030)
Italy	1992 to 2001	0.960 (0.898, 1.027)
Italy	1997 to 2006	0.958 (0.862, 1.064)
Jamaica	1942 to 1951	0.866 (0.848, 0.885)
Jamaica	1947 to 1956	0.881 (0.868, 0.895)
Jamaica	1952 to 1961	0.896 (0.884, 0.907)
Jamaica	1957 to 1966	0.913 (0.903, 0.923)
Jamaica	1962 to 1971	0.931 (0.921, 0.941)
Jamaica	1967 to 1976	0.947 (0.938, 0.957)
Jamaica	1972 to 1981	0.965 (0.956, 0.975)
Jamaica	1977 to 1986	0.982 (0.973, 0.992)
Jamaica	1982 to 1991	1.000 (1.000, 1.000)
Jamaica	1987 to 1996	1.015 (1.003, 1.027)

Jamaica	1992 to 2001	1.025 (1.009, 1.040)
Jamaica	1997 to 2006	0.916 (0.893, 0.940)
Japan	1942 to 1951	1.147 (1.121, 1.174)
Japan	1947 to 1956	1.118 (1.096, 1.140)
Japan	1952 to 1961	1.077 (1.058, 1.097)
Japan	1957 to 1966	1.052 (1.034, 1.070)
Japan	1962 to 1971	1.035 (1.018, 1.051)
Japan	1967 to 1976	1.023 (1.008, 1.039)
Japan	1972 to 1981	1.007 (0.992, 1.023)
Japan	1977 to 1986	1.003 (0.987, 1.019)
Japan	1982 to 1991	1.000 (1.000, 1.000)
Japan	1987 to 1996	0.993 (0.973, 1.013)
Japan	1992 to 2001	0.979 (0.955, 1.003)
Japan	1997 to 2006	0.952 (0.920, 0.984)
Jordan	1942 to 1951	0.793 (0.757, 0.831)
Jordan	1947 to 1956	0.819 (0.793, 0.846)
Jordan	1952 to 1961	0.842 (0.821, 0.864)
Jordan	1957 to 1966	0.871 (0.852, 0.889)
Jordan	1962 to 1971	0.899 (0.883, 0.915)
Jordan	1967 to 1976	0.924 (0.909, 0.939)
Jordan	1972 to 1981	0.954 (0.939, 0.970)
Jordan	1977 to 1986	0.980 (0.964, 0.995)
Jordan	1982 to 1991	1.000 (1.000, 1.000)
Jordan	1987 to 1996	0.996 (0.979, 1.014)

Jordan	1992 to 2001	0.898 (0.878, 0.919)
Jordan	1997 to 2006	0.853 (0.825, 0.882)
Kazakhstan	1942 to 1951	0.811 (0.799, 0.823)
Kazakhstan	1947 to 1956	0.831 (0.823, 0.840)
Kazakhstan	1952 to 1961	0.851 (0.843, 0.858)
Kazakhstan	1957 to 1966	0.873 (0.866, 0.880)
Kazakhstan	1962 to 1971	0.895 (0.889, 0.902)
Kazakhstan	1967 to 1976	0.920 (0.913, 0.927)
Kazakhstan	1972 to 1981	0.946 (0.940, 0.953)
Kazakhstan	1977 to 1986	0.974 (0.967, 0.981)
Kazakhstan	1982 to 1991	1.000 (1.000, 1.000)
Kazakhstan	1987 to 1996	1.026 (1.017, 1.034)
Kazakhstan	1992 to 2001	0.978 (0.967, 0.990)
Kazakhstan	1997 to 2006	0.937 (0.920, 0.953)
Kenya	1942 to 1951	0.930 (0.915, 0.945)
Kenya	1947 to 1956	0.939 (0.928, 0.949)
Kenya	1952 to 1961	0.951 (0.943, 0.960)
Kenya	1957 to 1966	0.961 (0.954, 0.969)
Kenya	1962 to 1971	0.970 (0.964, 0.976)
Kenya	1967 to 1976	0.980 (0.974, 0.986)
Kenya	1972 to 1981	0.988 (0.982, 0.993)
Kenya	1977 to 1986	0.994 (0.989, 1.000)
Kenya	1982 to 1991	1.000 (1.000, 1.000)
Kenya	1987 to 1996	1.001 (0.995, 1.007)

Kenya	1992 to 2001	0.962 (0.954, 0.969)
Kenya	1997 to 2006	0.676 (0.668, 0.685)
Kiribati	1942 to 1951	1.011 (0.921, 1.109)
Kiribati	1947 to 1956	1.017 (0.952, 1.087)
Kiribati	1952 to 1961	1.033 (0.978, 1.091)
Kiribati	1957 to 1966	1.045 (0.996, 1.096)
Kiribati	1962 to 1971	1.056 (1.011, 1.103)
Kiribati	1967 to 1976	1.061 (1.018, 1.106)
Kiribati	1972 to 1981	1.073 (1.031, 1.117)
Kiribati	1977 to 1986	1.077 (1.034, 1.121)
Kiribati	1982 to 1991	1.000 (1.000, 1.000)
Kiribati	1987 to 1996	0.937 (0.893, 0.983)
Kiribati	1992 to 2001	0.795 (0.746, 0.847)
Kiribati	1997 to 2006	0.749 (0.680, 0.825)
Kuwait	1942 to 1951	0.751 (0.737, 0.766)
Kuwait	1947 to 1956	0.778 (0.768, 0.788)
Kuwait	1952 to 1961	0.806 (0.798, 0.814)
Kuwait	1957 to 1966	0.834 (0.827, 0.841)
Kuwait	1962 to 1971	0.866 (0.860, 0.872)
Kuwait	1967 to 1976	0.900 (0.894, 0.906)
Kuwait	1972 to 1981	0.935 (0.930, 0.941)
Kuwait	1977 to 1986	0.972 (0.967, 0.978)
Kuwait	1982 to 1991	1.000 (1.000, 1.000)
Kuwait	1987 to 1996	1.032 (1.025, 1.040)

Kuwait	1992 to 2001	1.070 (1.058, 1.082)
Kuwait	1997 to 2006	1.114 (1.093, 1.135)
Kyrgyzstan	1942 to 1951	0.953 (0.936, 0.970)
Kyrgyzstan	1947 to 1956	0.950 (0.940, 0.961)
Kyrgyzstan	1952 to 1961	0.957 (0.948, 0.966)
Kyrgyzstan	1957 to 1966	0.963 (0.956, 0.971)
Kyrgyzstan	1962 to 1971	0.969 (0.962, 0.976)
Kyrgyzstan	1967 to 1976	0.978 (0.971, 0.984)
Kyrgyzstan	1972 to 1981	0.985 (0.979, 0.992)
Kyrgyzstan	1977 to 1986	0.993 (0.987, 1.000)
Kyrgyzstan	1982 to 1991	1.000 (1.000, 1.000)
Kyrgyzstan	1987 to 1996	1.000 (0.993, 1.008)
Kyrgyzstan	1992 to 2001	0.965 (0.955, 0.974)
Kyrgyzstan	1997 to 2006	0.838 (0.826, 0.851)
Lao People's Democratic Republic	1942 to 1951	1.100 (1.080, 1.120)
Lao People's Democratic Republic	1947 to 1956	1.099 (1.085, 1.113)
Lao People's Democratic Republic	1952 to 1961	1.101 (1.090, 1.113)
Lao People's Democratic Republic	1957 to 1966	1.094 (1.084, 1.104)
Lao People's Democratic Republic	1962 to 1971	1.084 (1.076, 1.093)
Lao People's Democratic Republic	1967 to 1976	1.071 (1.063, 1.079)
Lao People's Democratic Republic	1972 to 1981	1.055 (1.048, 1.063)
Lao People's Democratic Republic	1977 to 1986	1.030 (1.023, 1.038)
Lao People's Democratic Republic	1982 to 1991	1.000 (1.000, 1.000)
Lao People's Democratic Republic	1987 to 1996	0.964 (0.956, 0.972)

Lao People's Democratic Republic	1992 to 2001	0.920 (0.911, 0.929)
Lao People's Democratic Republic	1997 to 2006	0.708 (0.697, 0.718)
Latvia	1942 to 1951	0.921 (0.899, 0.943)
Latvia	1947 to 1956	0.934 (0.916, 0.952)
Latvia	1952 to 1961	0.948 (0.932, 0.965)
Latvia	1957 to 1966	0.962 (0.947, 0.977)
Latvia	1962 to 1971	0.974 (0.959, 0.989)
Latvia	1967 to 1976	0.981 (0.967, 0.996)
Latvia	1972 to 1981	0.989 (0.974, 1.003)
Latvia	1977 to 1986	0.997 (0.982, 1.012)
Latvia	1982 to 1991	1.000 (1.000, 1.000)
Latvia	1987 to 1996	0.999 (0.980, 1.018)
Latvia	1992 to 2001	0.817 (0.793, 0.841)
Latvia	1997 to 2006	0.678 (0.646, 0.712)
Lebanon	1942 to 1951	0.761 (0.751, 0.771)
Lebanon	1947 to 1956	0.786 (0.779, 0.793)
Lebanon	1952 to 1961	0.815 (0.808, 0.821)
Lebanon	1957 to 1966	0.844 (0.838, 0.850)
Lebanon	1962 to 1971	0.874 (0.868, 0.880)
Lebanon	1967 to 1976	0.905 (0.900, 0.911)
Lebanon	1972 to 1981	0.938 (0.932, 0.943)
Lebanon	1977 to 1986	0.970 (0.965, 0.976)
Lebanon	1982 to 1991	1.000 (1.000, 1.000)
Lebanon	1987 to 1996	1.030 (1.023, 1.037)

Lebanon	1992 to 2001	1.001 (0.991, 1.011)
Lebanon	1997 to 2006	0.997 (0.981, 1.012)
Lesotho	1942 to 1951	0.977 (0.954, 1.000)
Lesotho	1947 to 1956	0.986 (0.969, 1.004)
Lesotho	1952 to 1961	0.991 (0.976, 1.006)
Lesotho	1957 to 1966	0.995 (0.982, 1.008)
Lesotho	1962 to 1971	1.002 (0.990, 1.014)
Lesotho	1967 to 1976	1.006 (0.994, 1.017)
Lesotho	1972 to 1981	1.008 (0.997, 1.019)
Lesotho	1977 to 1986	1.004 (0.994, 1.015)
Lesotho	1982 to 1991	1.000 (1.000, 1.000)
Lesotho	1987 to 1996	0.992 (0.980, 1.004)
Lesotho	1992 to 2001	0.972 (0.957, 0.987)
Lesotho	1997 to 2006	0.705 (0.688, 0.722)
Liberia	1942 to 1951	0.979 (0.960, 0.998)
Liberia	1947 to 1956	0.984 (0.971, 0.997)
Liberia	1952 to 1961	0.988 (0.977, 0.998)
Liberia	1957 to 1966	0.994 (0.985, 1.003)
Liberia	1962 to 1971	0.998 (0.991, 1.007)
Liberia	1967 to 1976	1.003 (0.996, 1.010)
Liberia	1972 to 1981	1.007 (1.000, 1.013)
Liberia	1977 to 1986	1.006 (0.999, 1.012)
Liberia	1982 to 1991	1.000 (1.000, 1.000)
Liberia	1987 to 1996	0.988 (0.981, 0.995)

Liberia	1992 to 2001	0.966 (0.958, 0.974)
Liberia	1997 to 2006	0.927 (0.916, 0.938)
Libya	1942 to 1951	0.780 (0.764, 0.797)
Libya	1947 to 1956	0.803 (0.791, 0.815)
Libya	1952 to 1961	0.828 (0.818, 0.838)
Libya	1957 to 1966	0.855 (0.846, 0.864)
Libya	1962 to 1971	0.884 (0.876, 0.892)
Libya	1967 to 1976	0.913 (0.905, 0.920)
Libya	1972 to 1981	0.943 (0.936, 0.951)
Libya	1977 to 1986	0.973 (0.965, 0.980)
Libya	1982 to 1991	1.000 (1.000, 1.000)
Libya	1987 to 1996	1.028 (1.018, 1.038)
Libya	1992 to 2001	1.011 (0.998, 1.024)
Libya	1997 to 2006	0.986 (0.966, 1.006)
Lithuania	1942 to 1951	0.954 (0.934, 0.974)
Lithuania	1947 to 1956	0.959 (0.943, 0.975)
Lithuania	1952 to 1961	0.967 (0.953, 0.982)
Lithuania	1957 to 1966	0.976 (0.963, 0.989)
Lithuania	1962 to 1971	0.985 (0.972, 0.998)
Lithuania	1967 to 1976	0.991 (0.979, 1.004)
Lithuania	1972 to 1981	0.998 (0.986, 1.011)
Lithuania	1977 to 1986	1.002 (0.989, 1.015)
Lithuania	1982 to 1991	1.000 (1.000, 1.000)
Lithuania	1987 to 1996	0.993 (0.977, 1.009)

Lithuania	1992 to 2001	0.847 (0.828, 0.866)
Lithuania	1997 to 2006	0.786 (0.759, 0.815)
Luxembourg	1942 to 1951	0.751 (0.704, 0.801)
Luxembourg	1947 to 1956	0.783 (0.745, 0.823)
Luxembourg	1952 to 1961	0.819 (0.784, 0.855)
Luxembourg	1957 to 1966	0.848 (0.816, 0.881)
Luxembourg	1962 to 1971	0.875 (0.844, 0.908)
Luxembourg	1967 to 1976	0.905 (0.875, 0.937)
Luxembourg	1972 to 1981	0.935 (0.903, 0.968)
Luxembourg	1977 to 1986	0.963 (0.930, 0.998)
Luxembourg	1982 to 1991	1.000 (1.000, 1.000)
Luxembourg	1987 to 1996	1.017 (0.975, 1.061)
Luxembourg	1992 to 2001	0.940 (0.888, 0.996)
Luxembourg	1997 to 2006	0.890 (0.812, 0.975)
Madagascar	1942 to 1951	0.978 (0.964, 0.992)
Madagascar	1947 to 1956	0.980 (0.970, 0.989)
Madagascar	1952 to 1961	0.986 (0.978, 0.993)
Madagascar	1957 to 1966	0.991 (0.985, 0.998)
Madagascar	1962 to 1971	0.997 (0.992, 1.003)
Madagascar	1967 to 1976	1.001 (0.996, 1.007)
Madagascar	1972 to 1981	1.004 (0.999, 1.009)
Madagascar	1977 to 1986	1.005 (1.000, 1.010)
Madagascar	1982 to 1991	1.000 (1.000, 1.000)
Madagascar	1987 to 1996	0.988 (0.983, 0.993)

Madagascar	1992 to 2001	0.961 (0.955, 0.967)
Madagascar	1997 to 2006	0.553 (0.547, 0.559)
Malawi	1942 to 1951	1.025 (1.006, 1.045)
Malawi	1947 to 1956	1.028 (1.014, 1.042)
Malawi	1952 to 1961	1.028 (1.016, 1.039)
Malawi	1957 to 1966	1.026 (1.017, 1.036)
Malawi	1962 to 1971	1.025 (1.017, 1.034)
Malawi	1967 to 1976	1.021 (1.014, 1.029)
Malawi	1972 to 1981	1.018 (1.011, 1.025)
Malawi	1977 to 1986	1.011 (1.004, 1.018)
Malawi	1982 to 1991	1.000 (1.000, 1.000)
Malawi	1987 to 1996	0.982 (0.974, 0.989)
Malawi	1992 to 2001	0.950 (0.942, 0.959)
Malawi	1997 to 2006	0.597 (0.588, 0.605)
Malaysia	1942 to 1951	0.943 (0.911, 0.976)
Malaysia	1947 to 1956	0.964 (0.941, 0.988)
Malaysia	1952 to 1961	0.984 (0.964, 1.005)
Malaysia	1957 to 1966	1.003 (0.985, 1.022)
Malaysia	1962 to 1971	1.020 (1.003, 1.038)
Malaysia	1967 to 1976	1.032 (1.016, 1.049)
Malaysia	1972 to 1981	1.049 (1.033, 1.065)
Malaysia	1977 to 1986	1.056 (1.040, 1.073)
Malaysia	1982 to 1991	1.000 (1.000, 1.000)
Malaysia	1987 to 1996	0.964 (0.946, 0.983)

Malaysia	1992 to 2001	0.933 (0.910, 0.957)
Malaysia	1997 to 2006	0.882 (0.847, 0.918)
Maldives	1942 to 1951	1.030 (0.951, 1.115)
Maldives	1947 to 1956	1.046 (0.989, 1.106)
Maldives	1952 to 1961	1.056 (1.012, 1.102)
Maldives	1957 to 1966	1.060 (1.023, 1.099)
Maldives	1962 to 1971	1.056 (1.023, 1.090)
Maldives	1967 to 1976	1.045 (1.015, 1.076)
Maldives	1972 to 1981	1.041 (1.013, 1.070)
Maldives	1977 to 1986	1.024 (0.996, 1.052)
Maldives	1982 to 1991	1.000 (1.000, 1.000)
Maldives	1987 to 1996	0.885 (0.855, 0.915)
Maldives	1992 to 2001	0.758 (0.722, 0.795)
Maldives	1997 to 2006	0.638 (0.589, 0.692)
Mali	1942 to 1951	1.004 (0.991, 1.018)
Mali	1947 to 1956	1.010 (1.001, 1.020)
Mali	1952 to 1961	1.014 (1.006, 1.022)
Mali	1957 to 1966	1.015 (1.008, 1.022)
Mali	1962 to 1971	1.014 (1.008, 1.020)
Mali	1967 to 1976	1.015 (1.009, 1.020)
Mali	1972 to 1981	1.013 (1.008, 1.019)
Mali	1977 to 1986	1.007 (1.002, 1.012)
Mali	1982 to 1991	1.000 (1.000, 1.000)
Mali	1987 to 1996	0.987 (0.982, 0.993)

Mali	1992 to 2001	0.964 (0.958, 0.970)
Mali	1997 to 2006	0.733 (0.726, 0.739)
Malta	1942 to 1951	0.763 (0.720, 0.810)
Malta	1947 to 1956	0.797 (0.759, 0.837)
Malta	1952 to 1961	0.831 (0.795, 0.868)
Malta	1957 to 1966	0.865 (0.830, 0.900)
Malta	1962 to 1971	0.899 (0.865, 0.935)
Malta	1967 to 1976	0.928 (0.894, 0.963)
Malta	1972 to 1981	0.955 (0.922, 0.991)
Malta	1977 to 1986	0.982 (0.946, 1.019)
Malta	1982 to 1991	1.000 (1.000, 1.000)
Malta	1987 to 1996	1.004 (0.958, 1.051)
Malta	1992 to 2001	0.943 (0.884, 1.007)
Malta	1997 to 2006	0.878 (0.783, 0.984)
Marshall Islands	1942 to 1951	0.981 (0.845, 1.140)
Marshall Islands	1947 to 1956	1.001 (0.902, 1.111)
Marshall Islands	1952 to 1961	1.015 (0.931, 1.108)
Marshall Islands	1957 to 1966	1.029 (0.952, 1.111)
Marshall Islands	1962 to 1971	1.038 (0.968, 1.114)
Marshall Islands	1967 to 1976	1.047 (0.980, 1.118)
Marshall Islands	1972 to 1981	1.058 (0.993, 1.127)
Marshall Islands	1977 to 1986	1.057 (0.992, 1.127)
Marshall Islands	1982 to 1991	1.000 (1.000, 1.000)
Marshall Islands	1987 to 1996	0.904 (0.834, 0.980)

Marshall Islands	1992 to 2001	0.834 (0.753, 0.924)
Marshall Islands	1997 to 2006	0.752 (0.646, 0.875)
Mauritania	1942 to 1951	0.998 (0.981, 1.016)
Mauritania	1947 to 1956	1.007 (0.995, 1.019)
Mauritania	1952 to 1961	1.011 (1.001, 1.020)
Mauritania	1957 to 1966	1.015 (1.006, 1.023)
Mauritania	1962 to 1971	1.016 (1.009, 1.024)
Mauritania	1967 to 1976	1.017 (1.010, 1.024)
Mauritania	1972 to 1981	1.017 (1.010, 1.023)
Mauritania	1977 to 1986	1.009 (1.002, 1.016)
Mauritania	1982 to 1991	1.000 (1.000, 1.000)
Mauritania	1987 to 1996	0.985 (0.978, 0.992)
Mauritania	1992 to 2001	0.959 (0.951, 0.968)
Mauritania	1997 to 2006	0.823 (0.813, 0.833)
Mauritius	1942 to 1951	0.886 (0.863, 0.911)
Mauritius	1947 to 1956	0.907 (0.889, 0.925)
Mauritius	1952 to 1961	0.929 (0.913, 0.945)
Mauritius	1957 to 1966	0.947 (0.933, 0.962)
Mauritius	1962 to 1971	0.965 (0.951, 0.980)
Mauritius	1967 to 1976	0.979 (0.965, 0.993)
Mauritius	1972 to 1981	0.992 (0.978, 1.006)
Mauritius	1977 to 1986	0.997 (0.982, 1.011)
Mauritius	1982 to 1991	1.000 (1.000, 1.000)
Mauritius	1987 to 1996	0.999 (0.982, 1.017)

Mauritius	1992 to 2001	0.833 (0.814, 0.853)
Mauritius	1997 to 2006	0.717 (0.690, 0.745)
Mexico	1942 to 1951	0.850 (0.833, 0.867)
Mexico	1947 to 1956	0.868 (0.855, 0.881)
Mexico	1952 to 1961	0.890 (0.879, 0.901)
Mexico	1957 to 1966	0.908 (0.899, 0.918)
Mexico	1962 to 1971	0.927 (0.918, 0.936)
Mexico	1967 to 1976	0.946 (0.937, 0.955)
Mexico	1972 to 1981	0.968 (0.959, 0.977)
Mexico	1977 to 1986	0.985 (0.976, 0.995)
Mexico	1982 to 1991	1.000 (1.000, 1.000)
Mexico	1987 to 1996	1.007 (0.996, 1.019)
Mexico	1992 to 2001	0.957 (0.943, 0.972)
Mexico	1997 to 2006	0.939 (0.917, 0.961)
Micronesia (Federated States of)	1942 to 1951	1.059 (0.959, 1.168)
Micronesia (Federated States of)	1947 to 1956	1.078 (1.003, 1.159)
Micronesia (Federated States of)	1952 to 1961	1.103 (1.037, 1.173)
Micronesia (Federated States of)	1957 to 1966	1.122 (1.062, 1.186)
Micronesia (Federated States of)	1962 to 1971	1.136 (1.079, 1.196)
Micronesia (Federated States of)	1967 to 1976	1.145 (1.090, 1.203)
Micronesia (Federated States of)	1972 to 1981	1.169 (1.114, 1.226)
Micronesia (Federated States of)	1977 to 1986	1.174 (1.117, 1.232)
Micronesia (Federated States of)	1982 to 1991	1.000 (1.000, 1.000)
Micronesia (Federated States of)	1987 to 1996	0.868 (0.816, 0.923)

Micronesia (Federated States of)	1992 to 2001	0.814 (0.753, 0.879)
Micronesia (Federated States of)	1997 to 2006	0.777 (0.694, 0.870)
Monaco	1942 to 1951	0.732 (0.582, 0.919)
Monaco	1947 to 1956	0.758 (0.624, 0.921)
Monaco	1952 to 1961	0.795 (0.666, 0.950)
Monaco	1957 to 1966	0.829 (0.704, 0.978)
Monaco	1962 to 1971	0.865 (0.741, 1.010)
Monaco	1967 to 1976	0.896 (0.770, 1.043)
Monaco	1972 to 1981	0.933 (0.800, 1.089)
Monaco	1977 to 1986	0.968 (0.827, 1.134)
Monaco	1982 to 1991	1.000 (1.000, 1.000)
Monaco	1987 to 1996	0.956 (0.784, 1.166)
Monaco	1992 to 2001	0.944 (0.730, 1.220)
Monaco	1997 to 2006	0.953 (0.645, 1.407)
Mongolia	1942 to 1951	1.008 (0.989, 1.027)
Mongolia	1947 to 1956	1.008 (0.996, 1.021)
Mongolia	1952 to 1961	1.013 (1.003, 1.023)
Mongolia	1957 to 1966	1.015 (1.007, 1.024)
Mongolia	1962 to 1971	1.016 (1.008, 1.023)
Mongolia	1967 to 1976	1.020 (1.013, 1.026)
Mongolia	1972 to 1981	1.023 (1.016, 1.030)
Mongolia	1977 to 1986	1.025 (1.019, 1.032)
Mongolia	1982 to 1991	1.000 (1.000, 1.000)
Mongolia	1987 to 1996	0.894 (0.887, 0.902)

Mongolia	1992 to 2001	0.829 (0.820, 0.838)
Mongolia	1997 to 2006	0.774 (0.761, 0.787)
Montenegro	1942 to 1951	0.798 (0.763, 0.835)
Montenegro	1947 to 1956	0.819 (0.792, 0.848)
Montenegro	1952 to 1961	0.841 (0.816, 0.867)
Montenegro	1957 to 1966	0.868 (0.844, 0.893)
Montenegro	1962 to 1971	0.894 (0.870, 0.918)
Montenegro	1967 to 1976	0.918 (0.895, 0.942)
Montenegro	1972 to 1981	0.945 (0.921, 0.969)
Montenegro	1977 to 1986	0.972 (0.947, 0.997)
Montenegro	1982 to 1991	1.000 (1.000, 1.000)
Montenegro	1987 to 1996	1.025 (0.992, 1.059)
Montenegro	1992 to 2001	1.041 (0.998, 1.086)
Montenegro	1997 to 2006	1.031 (0.964, 1.103)
Morocco	1942 to 1951	0.749 (0.743, 0.756)
Morocco	1947 to 1956	0.775 (0.771, 0.780)
Morocco	1952 to 1961	0.806 (0.802, 0.810)
Morocco	1957 to 1966	0.837 (0.833, 0.840)
Morocco	1962 to 1971	0.870 (0.866, 0.873)
Morocco	1967 to 1976	0.902 (0.899, 0.906)
Morocco	1972 to 1981	0.936 (0.932, 0.939)
Morocco	1977 to 1986	0.969 (0.965, 0.972)
Morocco	1982 to 1991	1.000 (1.000, 1.000)
Morocco	1987 to 1996	1.031 (1.026, 1.035)

Morocco	1992 to 2001	1.040 (1.034, 1.047)
Morocco	1997 to 2006	1.013 (1.004, 1.023)
Mozambique	1942 to 1951	1.050 (1.038, 1.063)
Mozambique	1947 to 1956	1.049 (1.040, 1.057)
Mozambique	1952 to 1961	1.043 (1.036, 1.050)
Mozambique	1957 to 1966	1.039 (1.033, 1.046)
Mozambique	1962 to 1971	1.035 (1.030, 1.040)
Mozambique	1967 to 1976	1.030 (1.025, 1.035)
Mozambique	1972 to 1981	1.023 (1.019, 1.028)
Mozambique	1977 to 1986	1.012 (1.008, 1.017)
Mozambique	1982 to 1991	1.000 (1.000, 1.000)
Mozambique	1987 to 1996	0.983 (0.979, 0.988)
Mozambique	1992 to 2001	0.935 (0.929, 0.940)
Mozambique	1997 to 2006	0.571 (0.566, 0.576)
Myanmar	1942 to 1951	0.929 (0.914, 0.945)
Myanmar	1947 to 1956	0.949 (0.937, 0.960)
Myanmar	1952 to 1961	0.966 (0.956, 0.976)
Myanmar	1957 to 1966	0.979 (0.970, 0.988)
Myanmar	1962 to 1971	0.990 (0.982, 0.998)
Myanmar	1967 to 1976	0.998 (0.991, 1.006)
Myanmar	1972 to 1981	1.006 (0.998, 1.014)
Myanmar	1977 to 1986	1.006 (0.998, 1.014)
Myanmar	1982 to 1991	1.000 (1.000, 1.000)
Myanmar	1987 to 1996	0.991 (0.982, 1.001)

Myanmar	1992 to 2001	0.972 (0.960, 0.983)
Myanmar	1997 to 2006	0.835 (0.820, 0.851)
Namibia	1942 to 1951	1.050 (1.021, 1.079)
Namibia	1947 to 1956	1.051 (1.031, 1.071)
Namibia	1952 to 1961	1.049 (1.033, 1.066)
Namibia	1957 to 1966	1.046 (1.032, 1.060)
Namibia	1962 to 1971	1.041 (1.028, 1.053)
Namibia	1967 to 1976	1.035 (1.023, 1.046)
Namibia	1972 to 1981	1.030 (1.019, 1.040)
Namibia	1977 to 1986	1.017 (1.006, 1.028)
Namibia	1982 to 1991	1.000 (1.000, 1.000)
Namibia	1987 to 1996	0.977 (0.966, 0.989)
Namibia	1992 to 2001	0.944 (0.930, 0.958)
Namibia	1997 to 2006	0.892 (0.873, 0.912)
Nauru	1942 to 1951	1.248 (0.923, 1.687)
Nauru	1947 to 1956	1.271 (1.015, 1.592)
Nauru	1952 to 1961	1.285 (1.058, 1.561)
Nauru	1957 to 1966	1.293 (1.084, 1.543)
Nauru	1962 to 1971	1.307 (1.109, 1.541)
Nauru	1967 to 1976	1.318 (1.126, 1.542)
Nauru	1972 to 1981	1.335 (1.145, 1.558)
Nauru	1977 to 1986	1.101 (0.935, 1.296)
Nauru	1982 to 1991	1.000 (1.000, 1.000)
Nauru	1987 to 1996	1.048 (0.863, 1.273)

Nauru	1992 to 2001	1.014 (0.795, 1.294)
Nauru	1997 to 2006	0.892 (0.618, 1.289)
Nepal	1942 to 1951	0.946 (0.927, 0.966)
Nepal	1947 to 1956	0.958 (0.944, 0.972)
Nepal	1952 to 1961	0.965 (0.953, 0.978)
Nepal	1957 to 1966	0.968 (0.958, 0.979)
Nepal	1962 to 1971	0.974 (0.965, 0.984)
Nepal	1967 to 1976	0.980 (0.972, 0.989)
Nepal	1972 to 1981	0.988 (0.98, 0.997)
Nepal	1977 to 1986	0.994 (0.986, 1.003)
Nepal	1982 to 1991	1.000 (1.000, 1.000)
Nepal	1987 to 1996	1.011 (1.001, 1.022)
Nepal	1992 to 2001	1.021 (1.008, 1.034)
Nepal	1997 to 2006	0.953 (0.934, 0.972)
Netherlands	1942 to 1951	0.757 (0.746, 0.769)
Netherlands	1947 to 1956	0.784 (0.775, 0.794)
Netherlands	1952 to 1961	0.813 (0.804, 0.822)
Netherlands	1957 to 1966	0.843 (0.834, 0.851)
Netherlands	1962 to 1971	0.872 (0.864, 0.880)
Netherlands	1967 to 1976	0.904 (0.896, 0.912)
Netherlands	1972 to 1981	0.939 (0.931, 0.948)
Netherlands	1977 to 1986	0.973 (0.963, 0.982)
Netherlands	1982 to 1991	1.000 (1.000, 1.000)
Netherlands	1987 to 1996	1.022 (1.011, 1.034)

Netherlands	1992 to 2001	1.038 (1.023, 1.053)
Netherlands	1997 to 2006	1.048 (1.024, 1.073)
New Zealand	1942 to 1951	0.881 (0.830, 0.935)
New Zealand	1947 to 1956	0.919 (0.879, 0.962)
New Zealand	1952 to 1961	0.955 (0.918, 0.993)
New Zealand	1957 to 1966	0.987 (0.953, 1.023)
New Zealand	1962 to 1971	1.017 (0.984, 1.051)
New Zealand	1967 to 1976	1.046 (1.014, 1.080)
New Zealand	1972 to 1981	1.086 (1.052, 1.121)
New Zealand	1977 to 1986	1.047 (1.013, 1.082)
New Zealand	1982 to 1991	1.000 (1.000, 1.000)
New Zealand	1987 to 1996	0.940 (0.903, 0.978)
New Zealand	1992 to 2001	0.888 (0.843, 0.936)
New Zealand	1997 to 2006	0.877 (0.809, 0.949)
Nicaragua	1942 to 1951	0.878 (0.851, 0.907)
Nicaragua	1947 to 1956	0.898 (0.878, 0.918)
Nicaragua	1952 to 1961	0.917 (0.901, 0.934)
Nicaragua	1957 to 1966	0.934 (0.920, 0.949)
Nicaragua	1962 to 1971	0.949 (0.936, 0.963)
Nicaragua	1967 to 1976	0.964 (0.952, 0.977)
Nicaragua	1972 to 1981	0.981 (0.969, 0.993)
Nicaragua	1977 to 1986	0.990 (0.978, 1.003)
Nicaragua	1982 to 1991	1.000 (1.000, 1.000)
Nicaragua	1987 to 1996	1.008 (0.993, 1.023)

Nicaragua	1992 to 2001	0.963 (0.945, 0.981)
Nicaragua	1997 to 2006	0.925 (0.898, 0.953)
Niger	1942 to 1951	1.029 (1.017, 1.041)
Niger	1947 to 1956	1.028 (1.020, 1.037)
Niger	1952 to 1961	1.028 (1.022, 1.035)
Niger	1957 to 1966	1.029 (1.024, 1.035)
Niger	1962 to 1971	1.027 (1.023, 1.032)
Niger	1967 to 1976	1.025 (1.020, 1.029)
Niger	1972 to 1981	1.020 (1.016, 1.024)
Niger	1977 to 1986	1.011 (1.007, 1.015)
Niger	1982 to 1991	1.000 (1.000, 1.000)
Niger	1987 to 1996	0.982 (0.978, 0.986)
Niger	1992 to 2001	0.958 (0.953, 0.963)
Niger	1997 to 2006	0.922 (0.916, 0.928)
Nigeria	1942 to 1951	1.108 (1.093, 1.124)
Nigeria	1947 to 1956	1.096 (1.086, 1.107)
Nigeria	1952 to 1961	1.087 (1.079, 1.095)
Nigeria	1957 to 1966	1.079 (1.072, 1.086)
Nigeria	1962 to 1971	1.069 (1.064, 1.075)
Nigeria	1967 to 1976	1.060 (1.055, 1.065)
Nigeria	1972 to 1981	1.047 (1.043, 1.052)
Nigeria	1977 to 1986	1.027 (1.022, 1.032)
Nigeria	1982 to 1991	1.000 (1.000, 1.000)
Nigeria	1987 to 1996	0.968 (0.963, 0.973)

Nigeria	1992 to 2001	0.923 (0.917, 0.928)
Nigeria	1997 to 2006	0.768 (0.762, 0.775)
Niue	1942 to 1951	1.079 (0.557, 2.088)
Niue	1947 to 1956	1.092 (0.632, 1.887)
Niue	1952 to 1961	1.164 (0.706, 1.918)
Niue	1957 to 1966	1.141 (0.714, 1.823)
Niue	1962 to 1971	1.164 (0.745, 1.818)
Niue	1967 to 1976	1.183 (0.767, 1.825)
Niue	1972 to 1981	1.212 (0.786, 1.869)
Niue	1977 to 1986	1.219 (0.780, 1.905)
Niue	1982 to 1991	1.000 (1.000, 1.000)
Niue	1987 to 1996	0.859 (0.475, 1.553)
Niue	1992 to 2001	0.782 (0.361, 1.694)
Niue	1997 to 2006	0.847 (0.279, 2.566)
North Macedonia	1942 to 1951	0.769 (0.752, 0.786)
North Macedonia	1947 to 1956	0.792 (0.779, 0.805)
North Macedonia	1952 to 1961	0.818 (0.806, 0.830)
North Macedonia	1957 to 1966	0.848 (0.837, 0.860)
North Macedonia	1962 to 1971	0.879 (0.868, 0.890)
North Macedonia	1967 to 1976	0.907 (0.896, 0.918)
North Macedonia	1972 to 1981	0.939 (0.928, 0.951)
North Macedonia	1977 to 1986	0.969 (0.958, 0.981)
North Macedonia	1982 to 1991	1.000 (1.000, 1.000)
North Macedonia	1987 to 1996	1.028 (1.013, 1.044)

North Macedonia	1992 to 2001	1.041 (1.020, 1.063)
North Macedonia	1997 to 2006	0.980 (0.945, 1.015)
Northern Mariana Islands	1942 to 1951	1.000 (0.882, 1.133)
Northern Mariana Islands	1947 to 1956	1.003 (0.917, 1.097)
Northern Mariana Islands	1952 to 1961	1.012 (0.936, 1.094)
Northern Mariana Islands	1957 to 1966	1.022 (0.951, 1.099)
Northern Mariana Islands	1962 to 1971	1.027 (0.960, 1.098)
Northern Mariana Islands	1967 to 1976	1.030 (0.962, 1.102)
Northern Mariana Islands	1972 to 1981	1.035 (0.970, 1.105)
Northern Mariana Islands	1977 to 1986	1.037 (0.963, 1.118)
Northern Mariana Islands	1982 to 1991	1.000 (1.000, 1.000)
Northern Mariana Islands	1987 to 1996	0.833 (0.756, 0.917)
Northern Mariana Islands	1992 to 2001	0.731 (0.644, 0.831)
Northern Mariana Islands	1997 to 2006	0.728 (0.607, 0.873)
Norway	1942 to 1951	0.775 (0.758, 0.793)
Norway	1947 to 1956	0.806 (0.792, 0.821)
Norway	1952 to 1961	0.838 (0.825, 0.852)
Norway	1957 to 1966	0.866 (0.854, 0.879)
Norway	1962 to 1971	0.894 (0.882, 0.906)
Norway	1967 to 1976	0.919 (0.908, 0.931)
Norway	1972 to 1981	0.947 (0.935, 0.960)
Norway	1977 to 1986	0.974 (0.961, 0.988)
Norway	1982 to 1991	1.000 (1.000, 1.000)
Norway	1987 to 1996	1.021 (1.005, 1.038)

Norway	1992 to 2001	1.041 (1.020, 1.062)
Norway	1997 to 2006	0.980 (0.950, 1.012)
Oman	1942 to 1951	0.758 (0.710, 0.810)
Oman	1947 to 1956	0.790 (0.755, 0.826)
Oman	1952 to 1961	0.825 (0.796, 0.855)
Oman	1957 to 1966	0.860 (0.834, 0.887)
Oman	1962 to 1971	0.898 (0.874, 0.923)
Oman	1967 to 1976	0.936 (0.913, 0.959)
Oman	1972 to 1981	0.980 (0.959, 1.003)
Oman	1977 to 1986	1.022 (1.000, 1.045)
Oman	1982 to 1991	1.000 (1.000, 1.000)
Oman	1987 to 1996	0.996 (0.969, 1.023)
Oman	1992 to 2001	1.015 (0.978, 1.054)
Oman	1997 to 2006	1.037 (0.977, 1.100)
Pakistan	1942 to 1951	0.931 (0.913, 0.948)
Pakistan	1947 to 1956	0.941 (0.928, 0.953)
Pakistan	1952 to 1961	0.952 (0.941, 0.962)
Pakistan	1957 to 1966	0.962 (0.953, 0.971)
Pakistan	1962 to 1971	0.969 (0.961, 0.977)
Pakistan	1967 to 1976	0.978 (0.971, 0.986)
Pakistan	1972 to 1981	0.986 (0.979, 0.993)
Pakistan	1977 to 1986	0.992 (0.985, 0.999)
Pakistan	1982 to 1991	1.000 (1.000, 1.000)
Pakistan	1987 to 1996	1.011 (1.003, 1.019)

Pakistan	1992 to 2001	1.008 (0.998, 1.018)
Pakistan	1997 to 2006	0.841 (0.828, 0.855)
Palau	1942 to 1951	1.040 (0.843, 1.283)
Palau	1947 to 1956	1.062 (0.903, 1.249)
Palau	1952 to 1961	1.079 (0.934, 1.246)
Palau	1957 to 1966	1.100 (0.963, 1.257)
Palau	1962 to 1971	1.115 (0.982, 1.267)
Palau	1967 to 1976	1.130 (0.999, 1.280)
Palau	1972 to 1981	1.154 (1.019, 1.307)
Palau	1977 to 1986	1.166 (1.024, 1.326)
Palau	1982 to 1991	1.000 (1.000, 1.000)
Palau	1987 to 1996	0.892 (0.747, 1.067)
Palau	1992 to 2001	0.825 (0.650, 1.048)
Palau	1997 to 2006	0.796 (0.551, 1.149)
Palestine	1942 to 1951	0.831 (0.804, 0.859)
Palestine	1947 to 1956	0.850 (0.830, 0.870)
Palestine	1952 to 1961	0.868 (0.852, 0.885)
Palestine	1957 to 1966	0.892 (0.878, 0.906)
Palestine	1962 to 1971	0.915 (0.902, 0.928)
Palestine	1967 to 1976	0.935 (0.924, 0.947)
Palestine	1972 to 1981	0.958 (0.947, 0.970)
Palestine	1977 to 1986	0.980 (0.968, 0.992)
Palestine	1982 to 1991	1.000 (1.000, 1.000)
Palestine	1987 to 1996	0.954 (0.941, 0.967)

Palestine	1992 to 2001	0.938 (0.922, 0.954)
Palestine	1997 to 2006	0.936 (0.912, 0.961)
Panama	1942 to 1951	0.843 (0.827, 0.859)
Panama	1947 to 1956	0.863 (0.851, 0.875)
Panama	1952 to 1961	0.884 (0.874, 0.895)
Panama	1957 to 1966	0.903 (0.894, 0.913)
Panama	1962 to 1971	0.923 (0.914, 0.932)
Panama	1967 to 1976	0.943 (0.935, 0.952)
Panama	1972 to 1981	0.964 (0.956, 0.973)
Panama	1977 to 1986	0.984 (0.975, 0.992)
Panama	1982 to 1991	1.000 (1.000, 1.000)
Panama	1987 to 1996	1.016 (1.005, 1.027)
Panama	1992 to 2001	1.014 (1.000, 1.027)
Panama	1997 to 2006	0.923 (0.904, 0.942)
Papua New Guinea	1942 to 1951	0.999 (0.950, 1.051)
Papua New Guinea	1947 to 1956	1.016 (0.979, 1.054)
Papua New Guinea	1952 to 1961	1.030 (1.000, 1.060)
Papua New Guinea	1957 to 1966	1.039 (1.014, 1.066)
Papua New Guinea	1962 to 1971	1.046 (1.023, 1.069)
Papua New Guinea	1967 to 1976	1.048 (1.027, 1.069)
Papua New Guinea	1972 to 1981	1.052 (1.032, 1.072)
Papua New Guinea	1977 to 1986	1.047 (1.027, 1.067)
Papua New Guinea	1982 to 1991	1.000 (1.000, 1.000)
Papua New Guinea	1987 to 1996	0.901 (0.881, 0.921)

Papua New Guinea	1992 to 2001	0.759 (0.738, 0.781)
Papua New Guinea	1997 to 2006	0.590 (0.565, 0.616)
Paraguay	1942 to 1951	0.868 (0.855, 0.882)
Paraguay	1947 to 1956	0.893 (0.883, 0.903)
Paraguay	1952 to 1961	0.911 (0.902, 0.919)
Paraguay	1957 to 1966	0.929 (0.921, 0.936)
Paraguay	1962 to 1971	0.944 (0.937, 0.952)
Paraguay	1967 to 1976	0.960 (0.953, 0.967)
Paraguay	1972 to 1981	0.976 (0.969, 0.983)
Paraguay	1977 to 1986	0.989 (0.982, 0.996)
Paraguay	1982 to 1991	1.000 (1.000, 1.000)
Paraguay	1987 to 1996	1.015 (1.006, 1.023)
Paraguay	1992 to 2001	1.017 (1.006, 1.027)
Paraguay	1997 to 2006	0.867 (0.852, 0.881)
Peru	1942 to 1951	0.850 (0.834, 0.866)
Peru	1947 to 1956	0.868 (0.857, 0.880)
Peru	1952 to 1961	0.888 (0.878, 0.898)
Peru	1957 to 1966	0.909 (0.900, 0.918)
Peru	1962 to 1971	0.930 (0.922, 0.938)
Peru	1967 to 1976	0.949 (0.941, 0.957)
Peru	1972 to 1981	0.970 (0.962, 0.978)
Peru	1977 to 1986	0.987 (0.979, 0.995)
Peru	1982 to 1991	1.000 (1.000, 1.000)
Peru	1987 to 1996	1.014 (1.004, 1.024)

Peru	1992 to 2001	1.023 (1.010, 1.036)
Peru	1997 to 2006	0.925 (0.906, 0.943)
Philippines	1942 to 1951	1.030 (0.985, 1.077)
Philippines	1947 to 1956	1.036 (1.003, 1.069)
Philippines	1952 to 1961	1.043 (1.017, 1.071)
Philippines	1957 to 1966	1.048 (1.025, 1.072)
Philippines	1962 to 1971	1.045 (1.024, 1.067)
Philippines	1967 to 1976	1.038 (1.018, 1.058)
Philippines	1972 to 1981	1.033 (1.014, 1.052)
Philippines	1977 to 1986	1.019 (1.000, 1.038)
Philippines	1982 to 1991	1.000 (1.000, 1.000)
Philippines	1987 to 1996	0.805 (0.787, 0.824)
Philippines	1992 to 2001	0.730 (0.710, 0.751)
Philippines	1997 to 2006	0.587 (0.562, 0.613)
Poland	1942 to 1951	0.907 (0.898, 0.916)
Poland	1947 to 1956	0.920 (0.913, 0.927)
Poland	1952 to 1961	0.931 (0.924, 0.937)
Poland	1957 to 1966	0.941 (0.935, 0.947)
Poland	1962 to 1971	0.951 (0.945, 0.957)
Poland	1967 to 1976	0.961 (0.955, 0.967)
Poland	1972 to 1981	0.973 (0.968, 0.979)
Poland	1977 to 1986	0.986 (0.980, 0.992)
Poland	1982 to 1991	1.000 (1.000, 1.000)
Poland	1987 to 1996	1.010 (1.003, 1.018)

Poland	1992 to 2001	0.960 (0.950, 0.971)
Poland	1997 to 2006	0.945 (0.929, 0.962)
Portugal	1942 to 1951	0.703 (0.693, 0.714)
Portugal	1947 to 1956	0.743 (0.734, 0.752)
Portugal	1952 to 1961	0.782 (0.774, 0.791)
Portugal	1957 to 1966	0.818 (0.810, 0.826)
Portugal	1962 to 1971	0.853 (0.845, 0.861)
Portugal	1967 to 1976	0.887 (0.879, 0.895)
Portugal	1972 to 1981	0.923 (0.915, 0.931)
Portugal	1977 to 1986	0.960 (0.951, 0.969)
Portugal	1982 to 1991	1.000 (1.000, 1.000)
Portugal	1987 to 1996	1.021 (1.009, 1.033)
Portugal	1992 to 2001	1.005 (0.990, 1.020)
Portugal	1997 to 2006	0.952 (0.930, 0.974)
Puerto Rico	1942 to 1951	0.915 (0.902, 0.928)
Puerto Rico	1947 to 1956	0.927 (0.917, 0.938)
Puerto Rico	1952 to 1961	0.939 (0.929, 0.949)
Puerto Rico	1957 to 1966	0.952 (0.943, 0.962)
Puerto Rico	1962 to 1971	0.967 (0.958, 0.976)
Puerto Rico	1967 to 1976	0.984 (0.975, 0.993)
Puerto Rico	1972 to 1981	1.004 (0.995, 1.013)
Puerto Rico	1977 to 1986	1.007 (0.997, 1.016)
Puerto Rico	1982 to 1991	1.000 (1.000, 1.000)
Puerto Rico	1987 to 1996	0.993 (0.981, 1.004)

Puerto Rico	1992 to 2001	0.992 (0.977, 1.008)
Puerto Rico	1997 to 2006	1.013 (0.987, 1.039)
Qatar	1942 to 1951	0.758 (0.730, 0.787)
Qatar	1947 to 1956	0.784 (0.766, 0.803)
Qatar	1952 to 1961	0.811 (0.797, 0.826)
Qatar	1957 to 1966	0.842 (0.830, 0.855)
Qatar	1962 to 1971	0.876 (0.864, 0.889)
Qatar	1967 to 1976	0.911 (0.900, 0.922)
Qatar	1972 to 1981	0.949 (0.939, 0.959)
Qatar	1977 to 1986	0.988 (0.979, 0.998)
Qatar	1982 to 1991	1.000 (1.000, 1.000)
Qatar	1987 to 1996	1.030 (1.017, 1.043)
Qatar	1992 to 2001	1.071 (1.050, 1.093)
Qatar	1997 to 2006	1.119 (1.084, 1.155)
Republic of Korea	1942 to 1951	1.174 (1.075, 1.281)
Republic of Korea	1947 to 1956	1.205 (1.126, 1.289)
Republic of Korea	1952 to 1961	1.237 (1.167, 1.310)
Republic of Korea	1957 to 1966	1.250 (1.186, 1.318)
Republic of Korea	1962 to 1971	1.272 (1.210, 1.338)
Republic of Korea	1967 to 1976	1.299 (1.238, 1.364)
Republic of Korea	1972 to 1981	1.340 (1.277, 1.407)
Republic of Korea	1977 to 1986	1.091 (1.037, 1.149)
Republic of Korea	1982 to 1991	1.000 (1.000, 1.000)
Republic of Korea	1987 to 1996	0.925 (0.867, 0.987)

Republic of Korea	1992 to 2001	0.896 (0.825, 0.973)
Republic of Korea	1997 to 2006	0.858 (0.750, 0.982)
Republic of Moldova	1942 to 1951	0.970 (0.936, 1.005)
Republic of Moldova	1947 to 1956	0.968 (0.944, 0.993)
Republic of Moldova	1952 to 1961	0.973 (0.952, 0.995)
Republic of Moldova	1957 to 1966	0.979 (0.959, 0.999)
Republic of Moldova	1962 to 1971	0.985 (0.966, 1.004)
Republic of Moldova	1967 to 1976	0.988 (0.970, 1.006)
Republic of Moldova	1972 to 1981	0.993 (0.976, 1.011)
Republic of Moldova	1977 to 1986	0.998 (0.981, 1.017)
Republic of Moldova	1982 to 1991	1.000 (1.000, 1.000)
Republic of Moldova	1987 to 1996	0.911 (0.890, 0.933)
Republic of Moldova	1992 to 2001	0.635 (0.612, 0.659)
Republic of Moldova	1997 to 2006	0.525 (0.493, 0.559)
Romania	1942 to 1951	0.946 (0.880, 1.017)
Romania	1947 to 1956	0.959 (0.907, 1.014)
Romania	1952 to 1961	0.970 (0.923, 1.020)
Romania	1957 to 1966	0.983 (0.937, 1.031)
Romania	1962 to 1971	0.989 (0.946, 1.034)
Romania	1967 to 1976	0.989 (0.949, 1.032)
Romania	1972 to 1981	0.999 (0.959, 1.041)
Romania	1977 to 1986	1.001 (0.958, 1.046)
Romania	1982 to 1991	1.000 (1.000, 1.000)
Romania	1987 to 1996	0.835 (0.787, 0.887)

Romania	1992 to 2001	0.699 (0.643, 0.761)
Romania	1997 to 2006	0.616 (0.539, 0.703)
Russian Federation	1942 to 1951	0.914 (0.899, 0.929)
Russian Federation	1947 to 1956	0.917 (0.905, 0.928)
Russian Federation	1952 to 1961	0.932 (0.922, 0.943)
Russian Federation	1957 to 1966	0.948 (0.939, 0.958)
Russian Federation	1962 to 1971	0.959 (0.950, 0.969)
Russian Federation	1967 to 1976	0.969 (0.960, 0.978)
Russian Federation	1972 to 1981	0.979 (0.970, 0.988)
Russian Federation	1977 to 1986	0.990 (0.981, 0.999)
Russian Federation	1982 to 1991	1.000 (1.000, 1.000)
Russian Federation	1987 to 1996	1.005 (0.993, 1.017)
Russian Federation	1992 to 2001	0.969 (0.952, 0.985)
Russian Federation	1997 to 2006	0.763 (0.742, 0.784)
Rwanda	1942 to 1951	1.083 (1.065, 1.101)
Rwanda	1947 to 1956	1.078 (1.066, 1.090)
Rwanda	1952 to 1961	1.067 (1.057, 1.076)
Rwanda	1957 to 1966	1.059 (1.051, 1.067)
Rwanda	1962 to 1971	1.048 (1.041, 1.056)
Rwanda	1967 to 1976	1.037 (1.030, 1.043)
Rwanda	1972 to 1981	1.028 (1.022, 1.034)
Rwanda	1977 to 1986	1.015 (1.009, 1.021)
Rwanda	1982 to 1991	1.000 (1.000, 1.000)
Rwanda	1987 to 1996	0.977 (0.970, 0.983)

Rwanda	1992 to 2001	0.931 (0.924, 0.939)
Rwanda	1997 to 2006	0.517 (0.511, 0.524)
Saint Kitts and Nevis	1942 to 1951	0.912 (0.776, 1.072)
Saint Kitts and Nevis	1947 to 1956	0.924 (0.828, 1.031)
Saint Kitts and Nevis	1952 to 1961	0.936 (0.856, 1.024)
Saint Kitts and Nevis	1957 to 1966	0.946 (0.872, 1.026)
Saint Kitts and Nevis	1962 to 1971	0.957 (0.887, 1.032)
Saint Kitts and Nevis	1967 to 1976	0.967 (0.900, 1.039)
Saint Kitts and Nevis	1972 to 1981	0.978 (0.911, 1.051)
Saint Kitts and Nevis	1977 to 1986	0.989 (0.919, 1.063)
Saint Kitts and Nevis	1982 to 1991	1.000 (1.000, 1.000)
Saint Kitts and Nevis	1987 to 1996	1.004 (0.920, 1.097)
Saint Kitts and Nevis	1992 to 2001	0.918 (0.816, 1.034)
Saint Kitts and Nevis	1997 to 2006	0.861 (0.709, 1.045)
Saint Lucia	1942 to 1951	0.962 (0.876, 1.056)
Saint Lucia	1947 to 1956	0.969 (0.904, 1.038)
Saint Lucia	1952 to 1961	0.971 (0.916, 1.029)
Saint Lucia	1957 to 1966	0.977 (0.928, 1.028)
Saint Lucia	1962 to 1971	0.984 (0.938, 1.031)
Saint Lucia	1967 to 1976	0.990 (0.946, 1.036)
Saint Lucia	1972 to 1981	0.997 (0.954, 1.043)
Saint Lucia	1977 to 1986	1.000 (0.955, 1.047)
Saint Lucia	1982 to 1991	1.000 (1.000, 1.000)
Saint Lucia	1987 to 1996	0.998 (0.944, 1.055)

Saint Lucia	1992 to 2001	0.997 (0.927, 1.072)
Saint Lucia	1997 to 2006	0.889 (0.787, 1.005)
Saint Vincent and the Grenadines	1942 to 1951	0.899 (0.807, 1.002)
Saint Vincent and the Grenadines	1947 to 1956	0.911 (0.842, 0.987)
Saint Vincent and the Grenadines	1952 to 1961	0.926 (0.866, 0.990)
Saint Vincent and the Grenadines	1957 to 1966	0.937 (0.882, 0.994)
Saint Vincent and the Grenadines	1962 to 1971	0.948 (0.897, 1.002)
Saint Vincent and the Grenadines	1967 to 1976	0.963 (0.913, 1.016)
Saint Vincent and the Grenadines	1972 to 1981	0.977 (0.927, 1.030)
Saint Vincent and the Grenadines	1977 to 1986	0.989 (0.936, 1.044)
Saint Vincent and the Grenadines	1982 to 1991	1.000 (1.000, 1.000)
Saint Vincent and the Grenadines	1987 to 1996	1.008 (0.943, 1.077)
Saint Vincent and the Grenadines	1992 to 2001	1.011 (0.929, 1.100)
Saint Vincent and the Grenadines	1997 to 2006	0.915 (0.797, 1.051)
Samoa	1942 to 1951	1.010 (0.935, 1.091)
Samoa	1947 to 1956	1.020 (0.964, 1.079)
Samoa	1952 to 1961	1.027 (0.979, 1.077)
Samoa	1957 to 1966	1.031 (0.988, 1.075)
Samoa	1962 to 1971	1.031 (0.993, 1.072)
Samoa	1967 to 1976	1.030 (0.993, 1.068)
Samoa	1972 to 1981	1.032 (0.996, 1.069)
Samoa	1977 to 1986	1.026 (0.989, 1.065)
Samoa	1982 to 1991	1.000 (1.000, 1.000)
Samoa	1987 to 1996	0.892 (0.854, 0.932)

Samoa	1992 to 2001	0.850 (0.805, 0.898)
Samoa	1997 to 2006	0.867 (0.802, 0.936)
San Marino	1942 to 1951	0.753 (0.580, 0.977)
San Marino	1947 to 1956	0.779 (0.634, 0.956)
San Marino	1952 to 1961	0.807 (0.673, 0.969)
San Marino	1957 to 1966	0.841 (0.714, 0.990)
San Marino	1962 to 1971	0.871 (0.747, 1.014)
San Marino	1967 to 1976	0.901 (0.778, 1.043)
San Marino	1972 to 1981	0.936 (0.807, 1.085)
San Marino	1977 to 1986	0.968 (0.828, 1.132)
San Marino	1982 to 1991	1.000 (1.000, 1.000)
San Marino	1987 to 1996	0.960 (0.789, 1.169)
San Marino	1992 to 2001	0.921 (0.716, 1.186)
San Marino	1997 to 2006	0.938 (0.639, 1.377)
Sao Tome and Principe	1942 to 1951	1.051 (0.960, 1.150)
Sao Tome and Principe	1947 to 1956	1.049 (0.986, 1.116)
Sao Tome and Principe	1952 to 1961	1.042 (0.991, 1.095)
Sao Tome and Principe	1957 to 1966	1.037 (0.994, 1.082)
Sao Tome and Principe	1962 to 1971	1.031 (0.993, 1.070)
Sao Tome and Principe	1967 to 1976	1.026 (0.992, 1.061)
Sao Tome and Principe	1972 to 1981	1.021 (0.989, 1.053)
Sao Tome and Principe	1977 to 1986	1.011 (0.979, 1.043)
Sao Tome and Principe	1982 to 1991	1.000 (1.000, 1.000)
Sao Tome and Principe	1987 to 1996	0.981 (0.946, 1.017)

Sao Tome and Principe	1992 to 2001	0.950 (0.910, 0.993)
Sao Tome and Principe	1997 to 2006	0.605 (0.565, 0.649)
Saudi Arabia	1942 to 1951	0.794 (0.737, 0.857)
Saudi Arabia	1947 to 1956	0.825 (0.783, 0.869)
Saudi Arabia	1952 to 1961	0.861 (0.826, 0.897)
Saudi Arabia	1957 to 1966	0.897 (0.866, 0.929)
Saudi Arabia	1962 to 1971	0.932 (0.904, 0.961)
Saudi Arabia	1967 to 1976	0.965 (0.939, 0.992)
Saudi Arabia	1972 to 1981	1.004 (0.978, 1.031)
Saudi Arabia	1977 to 1986	1.037 (1.011, 1.064)
Saudi Arabia	1982 to 1991	1.000 (1.000, 1.000)
Saudi Arabia	1987 to 1996	0.973 (0.943, 1.004)
Saudi Arabia	1992 to 2001	0.970 (0.930, 1.012)
Saudi Arabia	1997 to 2006	0.963 (0.898, 1.032)
Senegal	1942 to 1951	0.958 (0.947, 0.969)
Senegal	1947 to 1956	0.968 (0.960, 0.976)
Senegal	1952 to 1961	0.978 (0.971, 0.984)
Senegal	1957 to 1966	0.983 (0.978, 0.989)
Senegal	1962 to 1971	0.987 (0.983, 0.992)
Senegal	1967 to 1976	0.994 (0.990, 0.999)
Senegal	1972 to 1981	1.000 (0.996, 1.004)
Senegal	1977 to 1986	1.000 (0.996, 1.004)
Senegal	1982 to 1991	1.000 (1.000, 1.000)
Senegal	1987 to 1996	0.998 (0.993, 1.002)

Senegal	1992 to 2001	0.982 (0.977, 0.988)
Senegal	1997 to 2006	0.657 (0.651, 0.663)
Serbia	1942 to 1951	0.791 (0.782, 0.800)
Serbia	1947 to 1956	0.813 (0.806, 0.821)
Serbia	1952 to 1961	0.836 (0.829, 0.842)
Serbia	1957 to 1966	0.862 (0.856, 0.868)
Serbia	1962 to 1971	0.889 (0.883, 0.895)
Serbia	1967 to 1976	0.916 (0.910, 0.922)
Serbia	1972 to 1981	0.943 (0.937, 0.950)
Serbia	1977 to 1986	0.971 (0.965, 0.978)
Serbia	1982 to 1991	1.000 (1.000, 1.000)
Serbia	1987 to 1996	1.025 (1.016, 1.034)
Serbia	1992 to 2001	1.046 (1.034, 1.058)
Serbia	1997 to 2006	1.001 (0.982, 1.019)
Seychelles	1942 to 1951	0.925 (0.831, 1.031)
Seychelles	1947 to 1956	0.940 (0.870, 1.017)
Seychelles	1952 to 1961	0.955 (0.897, 1.017)
Seychelles	1957 to 1966	0.970 (0.917, 1.026)
Seychelles	1962 to 1971	0.979 (0.930, 1.032)
Seychelles	1967 to 1976	0.984 (0.937, 1.035)
Seychelles	1972 to 1981	0.996 (0.948, 1.046)
Seychelles	1977 to 1986	0.999 (0.950, 1.052)
Seychelles	1982 to 1991	1.000 (1.000, 1.000)
Seychelles	1987 to 1996	0.876 (0.821, 0.936)

Seychelles	1992 to 2001	0.769 (0.704, 0.841)
Seychelles	1997 to 2006	0.702 (0.608, 0.811)
Sierra Leone	1942 to 1951	1.038 (1.023, 1.054)
Sierra Leone	1947 to 1956	1.041 (1.030, 1.052)
Sierra Leone	1952 to 1961	1.039 (1.030, 1.047)
Sierra Leone	1957 to 1966	1.037 (1.029, 1.044)
Sierra Leone	1962 to 1971	1.033 (1.027, 1.040)
Sierra Leone	1967 to 1976	1.029 (1.024, 1.035)
Sierra Leone	1972 to 1981	1.023 (1.017, 1.028)
Sierra Leone	1977 to 1986	1.012 (1.007, 1.018)
Sierra Leone	1982 to 1991	1.000 (1.000, 1.000)
Sierra Leone	1987 to 1996	0.982 (0.976, 0.987)
Sierra Leone	1992 to 2001	0.956 (0.949, 0.962)
Sierra Leone	1997 to 2006	0.914 (0.906, 0.923)
Singapore	1942 to 1951	1.091 (1.034, 1.151)
Singapore	1947 to 1956	1.101 (1.059, 1.145)
Singapore	1952 to 1961	1.110 (1.074, 1.148)
Singapore	1957 to 1966	1.118 (1.085, 1.152)
Singapore	1962 to 1971	1.121 (1.090, 1.152)
Singapore	1967 to 1976	1.120 (1.091, 1.150)
Singapore	1972 to 1981	1.128 (1.100, 1.157)
Singapore	1977 to 1986	1.128 (1.099, 1.158)
Singapore	1982 to 1991	1.000 (1.000, 1.000)
Singapore	1987 to 1996	0.917 (0.888, 0.948)

Singapore	1992 to 2001	0.874 (0.832, 0.917)
Singapore	1997 to 2006	0.833 (0.765, 0.907)
Slovakia	1942 to 1951	0.851 (0.833, 0.870)
Slovakia	1947 to 1956	0.867 (0.853, 0.881)
Slovakia	1952 to 1961	0.885 (0.872, 0.898)
Slovakia	1957 to 1966	0.901 (0.889, 0.913)
Slovakia	1962 to 1971	0.919 (0.907, 0.930)
Slovakia	1967 to 1976	0.936 (0.925, 0.948)
Slovakia	1972 to 1981	0.958 (0.946, 0.969)
Slovakia	1977 to 1986	0.978 (0.966, 0.990)
Slovakia	1982 to 1991	1.000 (1.000, 1.000)
Slovakia	1987 to 1996	1.015 (0.999, 1.031)
Slovakia	1992 to 2001	0.884 (0.864, 0.904)
Slovakia	1997 to 2006	0.835 (0.805, 0.867)
Slovenia	1942 to 1951	0.826 (0.804, 0.847)
Slovenia	1947 to 1956	0.849 (0.832, 0.866)
Slovenia	1952 to 1961	0.872 (0.856, 0.887)
Slovenia	1957 to 1966	0.895 (0.881, 0.911)
Slovenia	1962 to 1971	0.916 (0.902, 0.931)
Slovenia	1967 to 1976	0.939 (0.924, 0.953)
Slovenia	1972 to 1981	0.961 (0.947, 0.976)
Slovenia	1977 to 1986	0.980 (0.964, 0.995)
Slovenia	1982 to 1991	1.000 (1.000, 1.000)
Slovenia	1987 to 1996	1.012 (0.992, 1.034)

Slovenia	1992 to 2001	0.887 (0.861, 0.914)
Slovenia	1997 to 2006	0.805 (0.765, 0.846)
Solomon Islands	1942 to 1951	1.111 (0.988, 1.248)
Solomon Islands	1947 to 1956	1.126 (1.036, 1.224)
Solomon Islands	1952 to 1961	1.148 (1.073, 1.230)
Solomon Islands	1957 to 1966	1.161 (1.096, 1.231)
Solomon Islands	1962 to 1971	1.171 (1.112, 1.233)
Solomon Islands	1967 to 1976	1.174 (1.120, 1.230)
Solomon Islands	1972 to 1981	1.188 (1.136, 1.243)
Solomon Islands	1977 to 1986	1.187 (1.135, 1.243)
Solomon Islands	1982 to 1991	1.000 (1.000, 1.000)
Solomon Islands	1987 to 1996	0.807 (0.762, 0.855)
Solomon Islands	1992 to 2001	0.722 (0.671, 0.776)
Solomon Islands	1997 to 2006	0.668 (0.602, 0.742)
Somalia	1942 to 1951	0.999 (0.989, 1.009)
Somalia	1947 to 1956	1.001 (0.994, 1.008)
Somalia	1952 to 1961	1.004 (0.998, 1.010)
Somalia	1957 to 1966	1.006 (1.001, 1.011)
Somalia	1962 to 1971	1.006 (1.002, 1.011)
Somalia	1967 to 1976	1.007 (1.002, 1.011)
Somalia	1972 to 1981	1.007 (1.003, 1.010)
Somalia	1977 to 1986	1.005 (1.001, 1.008)
Somalia	1982 to 1991	1.000 (1.000, 1.000)
Somalia	1987 to 1996	0.993 (0.989, 0.997)

Somalia	1992 to 2001	0.985 (0.981, 0.990)
Somalia	1997 to 2006	0.972 (0.966, 0.978)
South Africa	1942 to 1951	0.938 (0.897, 0.980)
South Africa	1947 to 1956	0.949 (0.919, 0.979)
South Africa	1952 to 1961	0.956 (0.932, 0.982)
South Africa	1957 to 1966	0.967 (0.945, 0.990)
South Africa	1962 to 1971	0.977 (0.956, 0.998)
South Africa	1967 to 1976	0.983 (0.964, 1.003)
South Africa	1972 to 1981	0.994 (0.974, 1.013)
South Africa	1977 to 1986	1.001 (0.981, 1.020)
South Africa	1982 to 1991	1.000 (1.000, 1.000)
South Africa	1987 to 1996	0.939 (0.917, 0.961)
South Africa	1992 to 2001	0.877 (0.849, 0.906)
South Africa	1997 to 2006	0.826 (0.784, 0.872)
South Sudan	1942 to 1951	0.935 (0.919, 0.951)
South Sudan	1947 to 1956	0.944 (0.933, 0.956)
South Sudan	1952 to 1961	0.955 (0.945, 0.964)
South Sudan	1957 to 1966	0.966 (0.958, 0.974)
South Sudan	1962 to 1971	0.975 (0.968, 0.983)
South Sudan	1967 to 1976	0.984 (0.977, 0.990)
South Sudan	1972 to 1981	0.992 (0.986, 0.998)
South Sudan	1977 to 1986	0.998 (0.992, 1.005)
South Sudan	1982 to 1991	1.000 (1.000, 1.000)
South Sudan	1987 to 1996	0.996 (0.989, 1.004)

South Sudan	1992 to 2001	0.990 (0.981, 0.999)
South Sudan	1997 to 2006	0.978 (0.966, 0.990)
Spain	1942 to 1951	0.776 (0.754, 0.798)
Spain	1947 to 1956	0.809 (0.790, 0.827)
Spain	1952 to 1961	0.832 (0.815, 0.849)
Spain	1957 to 1966	0.856 (0.841, 0.872)
Spain	1962 to 1971	0.882 (0.867, 0.898)
Spain	1967 to 1976	0.911 (0.897, 0.926)
Spain	1972 to 1981	0.943 (0.928, 0.958)
Spain	1977 to 1986	0.977 (0.961, 0.994)
Spain	1982 to 1991	1.000 (1.000, 1.000)
Spain	1987 to 1996	0.949 (0.928, 0.970)
Spain	1992 to 2001	0.908 (0.881, 0.936)
Spain	1997 to 2006	0.875 (0.835, 0.917)
Sri Lanka	1942 to 1951	0.932 (0.918, 0.945)
Sri Lanka	1947 to 1956	0.940 (0.930, 0.951)
Sri Lanka	1952 to 1961	0.948 (0.938, 0.957)
Sri Lanka	1957 to 1966	0.956 (0.948, 0.965)
Sri Lanka	1962 to 1971	0.968 (0.960, 0.977)
Sri Lanka	1967 to 1976	0.982 (0.974, 0.990)
Sri Lanka	1972 to 1981	0.996 (0.988, 1.004)
Sri Lanka	1977 to 1986	1.000 (0.992, 1.008)
Sri Lanka	1982 to 1991	1.000 (1.000, 1.000)
Sri Lanka	1987 to 1996	1.000 (0.990, 1.010)

Sri Lanka	1992 to 2001	0.989 (0.976, 1.002)
Sri Lanka	1997 to 2006	0.792 (0.774, 0.809)
Sudan	1942 to 1951	0.883 (0.875, 0.891)
Sudan	1947 to 1956	0.905 (0.899, 0.911)
Sudan	1952 to 1961	0.926 (0.921, 0.930)
Sudan	1957 to 1966	0.943 (0.939, 0.947)
Sudan	1962 to 1971	0.959 (0.955, 0.963)
Sudan	1967 to 1976	0.974 (0.970, 0.977)
Sudan	1972 to 1981	0.986 (0.983, 0.989)
Sudan	1977 to 1986	0.996 (0.993, 0.999)
Sudan	1982 to 1991	1.000 (1.000, 1.000)
Sudan	1987 to 1996	0.992 (0.988, 0.995)
Sudan	1992 to 2001	0.967 (0.962, 0.971)
Sudan	1997 to 2006	0.835 (0.830, 0.841)
Suriname	1942 to 1951	0.853 (0.812, 0.895)
Suriname	1947 to 1956	0.870 (0.839, 0.903)
Suriname	1952 to 1961	0.890 (0.863, 0.918)
Suriname	1957 to 1966	0.908 (0.884, 0.934)
Suriname	1962 to 1971	0.925 (0.902, 0.949)
Suriname	1967 to 1976	0.945 (0.923, 0.968)
Suriname	1972 to 1981	0.965 (0.943, 0.988)
Suriname	1977 to 1986	0.984 (0.960, 1.008)
Suriname	1982 to 1991	1.000 (1.000, 1.000)
Suriname	1987 to 1996	1.015 (0.986, 1.046)

Suriname	1992 to 2001	1.021 (0.983, 1.060)
Suriname	1997 to 2006	0.905 (0.851, 0.962)
Sweden	1942 to 1951	0.765 (0.754, 0.777)
Sweden	1947 to 1956	0.792 (0.782, 0.802)
Sweden	1952 to 1961	0.822 (0.813, 0.831)
Sweden	1957 to 1966	0.850 (0.842, 0.859)
Sweden	1962 to 1971	0.878 (0.869, 0.886)
Sweden	1967 to 1976	0.909 (0.901, 0.917)
Sweden	1972 to 1981	0.942 (0.933, 0.950)
Sweden	1977 to 1986	0.971 (0.962, 0.980)
Sweden	1982 to 1991	1.000 (1.000, 1.000)
Sweden	1987 to 1996	1.028 (1.017, 1.040)
Sweden	1992 to 2001	1.054 (1.038, 1.070)
Sweden	1997 to 2006	1.122 (1.097, 1.147)
Switzerland	1942 to 1951	0.759 (0.747, 0.771)
Switzerland	1947 to 1956	0.788 (0.778, 0.798)
Switzerland	1952 to 1961	0.818 (0.809, 0.828)
Switzerland	1957 to 1966	0.849 (0.840, 0.858)
Switzerland	1962 to 1971	0.877 (0.869, 0.886)
Switzerland	1967 to 1976	0.905 (0.897, 0.914)
Switzerland	1972 to 1981	0.939 (0.930, 0.948)
Switzerland	1977 to 1986	0.970 (0.961, 0.979)
Switzerland	1982 to 1991	1.000 (1.000, 1.000)
Switzerland	1987 to 1996	1.030 (1.018, 1.042)

Switzerland	1992 to 2001	1.056 (1.040, 1.072)
Switzerland	1997 to 2006	1.074 (1.049, 1.099)
Syrian Arab Republic	1942 to 1951	0.794 (0.772, 0.817)
Syrian Arab Republic	1947 to 1956	0.819 (0.803, 0.836)
Syrian Arab Republic	1952 to 1961	0.844 (0.830, 0.858)
Syrian Arab Republic	1957 to 1966	0.871 (0.859, 0.883)
Syrian Arab Republic	1962 to 1971	0.898 (0.886, 0.909)
Syrian Arab Republic	1967 to 1976	0.923 (0.912, 0.934)
Syrian Arab Republic	1972 to 1981	0.951 (0.940, 0.962)
Syrian Arab Republic	1977 to 1986	0.977 (0.966, 0.988)
Syrian Arab Republic	1982 to 1991	1.000 (1.000, 1.000)
Syrian Arab Republic	1987 to 1996	0.961 (0.947, 0.976)
Syrian Arab Republic	1992 to 2001	0.940 (0.923, 0.957)
Syrian Arab Republic	1997 to 2006	0.925 (0.900, 0.950)
Taiwan (Province of China)	1942 to 1951	1.220 (1.102, 1.351)
Taiwan (Province of China)	1947 to 1956	1.251 (1.161, 1.347)
Taiwan (Province of China)	1952 to 1961	1.281 (1.199, 1.369)
Taiwan (Province of China)	1957 to 1966	1.305 (1.227, 1.387)
Taiwan (Province of China)	1962 to 1971	1.317 (1.242, 1.396)
Taiwan (Province of China)	1967 to 1976	1.321 (1.248, 1.399)
Taiwan (Province of China)	1972 to 1981	1.331 (1.258, 1.408)
Taiwan (Province of China)	1977 to 1986	1.305 (1.232, 1.383)
Taiwan (Province of China)	1982 to 1991	1.000 (1.000, 1.000)
Taiwan (Province of China)	1987 to 1996	0.853 (0.787, 0.924)

Taiwan (Province of China)	1992 to 2001	0.762 (0.685, 0.847)
Taiwan (Province of China)	1997 to 2006	0.676 (0.565, 0.808)
Tajikistan	1942 to 1951	0.941 (0.925, 0.956)
Tajikistan	1947 to 1956	0.946 (0.936, 0.956)
Tajikistan	1952 to 1961	0.955 (0.948, 0.963)
Tajikistan	1957 to 1966	0.965 (0.958, 0.971)
Tajikistan	1962 to 1971	0.972 (0.966, 0.977)
Tajikistan	1967 to 1976	0.981 (0.975, 0.986)
Tajikistan	1972 to 1981	0.990 (0.984, 0.995)
Tajikistan	1977 to 1986	0.998 (0.993, 1.004)
Tajikistan	1982 to 1991	1.000 (1.000, 1.000)
Tajikistan	1987 to 1996	0.995 (0.990, 1.001)
Tajikistan	1992 to 2001	0.986 (0.978, 0.993)
Tajikistan	1997 to 2006	0.853 (0.844, 0.863)
Thailand	1942 to 1951	0.908 (0.841, 0.979)
Thailand	1947 to 1956	0.936 (0.884, 0.991)
Thailand	1952 to 1961	0.957 (0.911, 1.005)
Thailand	1957 to 1966	0.975 (0.932, 1.019)
Thailand	1962 to 1971	0.987 (0.947, 1.030)
Thailand	1967 to 1976	0.994 (0.955, 1.036)
Thailand	1972 to 1981	1.007 (0.967, 1.049)
Thailand	1977 to 1986	1.007 (0.965, 1.050)
Thailand	1982 to 1991	1.000 (1.000, 1.000)
Thailand	1987 to 1996	0.811 (0.766, 0.858)

Thailand	1992 to 2001	0.685 (0.634, 0.739)
Thailand	1997 to 2006	0.604 (0.532, 0.686)
Timor-Leste	1942 to 1951	1.022 (0.989, 1.057)
Timor-Leste	1947 to 1956	1.026 (1.002, 1.051)
Timor-Leste	1952 to 1961	1.033 (1.013, 1.054)
Timor-Leste	1957 to 1966	1.036 (1.018, 1.054)
Timor-Leste	1962 to 1971	1.036 (1.020, 1.053)
Timor-Leste	1967 to 1976	1.033 (1.017, 1.048)
Timor-Leste	1972 to 1981	1.029 (1.015, 1.045)
Timor-Leste	1977 to 1986	1.017 (1.002, 1.032)
Timor-Leste	1982 to 1991	1.000 (1.000, 1.000)
Timor-Leste	1987 to 1996	0.983 (0.967, 0.999)
Timor-Leste	1992 to 2001	0.958 (0.940, 0.977)
Timor-Leste	1997 to 2006	0.928 (0.903, 0.953)
Togo	1942 to 1951	0.979 (0.965, 0.993)
Togo	1947 to 1956	0.986 (0.976, 0.996)
Togo	1952 to 1961	0.995 (0.987, 1.003)
Togo	1957 to 1966	1.001 (0.994, 1.008)
Togo	1962 to 1971	1.004 (0.998, 1.010)
Togo	1967 to 1976	1.007 (1.002, 1.012)
Togo	1972 to 1981	1.010 (1.004, 1.015)
Togo	1977 to 1986	1.006 (1.001, 1.011)
Togo	1982 to 1991	1.000 (1.000, 1.000)
Togo	1987 to 1996	0.991 (0.985, 0.997)

Togo	1992 to 2001	0.970 (0.964, 0.977)
Togo	1997 to 2006	0.935 (0.926, 0.945)
Tokelau	1942 to 1951	0.889 (0.389, 2.029)
Tokelau	1947 to 1956	0.945 (0.514, 1.735)
Tokelau	1952 to 1961	0.915 (0.515, 1.627)
Tokelau	1957 to 1966	0.938 (0.559, 1.574)
Tokelau	1962 to 1971	0.974 (0.601, 1.579)
Tokelau	1967 to 1976	0.986 (0.620, 1.568)
Tokelau	1972 to 1981	0.994 (0.619, 1.595)
Tokelau	1977 to 1986	1.002 (0.629, 1.594)
Tokelau	1982 to 1991	1.000 (1.000, 1.000)
Tokelau	1987 to 1996	0.883 (0.503, 1.552)
Tokelau	1992 to 2001	0.646 (0.286, 1.462)
Tokelau	1997 to 2006	0.672 (0.219, 2.058)
Tonga	1942 to 1951	1.226 (1.102, 1.364)
Tonga	1947 to 1956	1.240 (1.142, 1.347)
Tonga	1952 to 1961	1.259 (1.171, 1.353)
Tonga	1957 to 1966	1.271 (1.191, 1.355)
Tonga	1962 to 1971	1.274 (1.200, 1.352)
Tonga	1967 to 1976	1.275 (1.205, 1.348)
Tonga	1972 to 1981	1.290 (1.221, 1.363)
Tonga	1977 to 1986	1.281 (1.211, 1.355)
Tonga	1982 to 1991	1.000 (1.000, 1.000)
Tonga	1987 to 1996	0.896 (0.833, 0.963)

Tonga	1992 to 2001	0.827 (0.755, 0.906)
Tonga	1997 to 2006	0.765 (0.668, 0.876)
Trinidad and Tobago	1942 to 1951	0.873 (0.848, 0.898)
Trinidad and Tobago	1947 to 1956	0.881 (0.863, 0.900)
Trinidad and Tobago	1952 to 1961	0.896 (0.879, 0.913)
Trinidad and Tobago	1957 to 1966	0.912 (0.897, 0.927)
Trinidad and Tobago	1962 to 1971	0.927 (0.912, 0.941)
Trinidad and Tobago	1967 to 1976	0.946 (0.931, 0.960)
Trinidad and Tobago	1972 to 1981	0.965 (0.951, 0.979)
Trinidad and Tobago	1977 to 1986	0.983 (0.968, 0.997)
Trinidad and Tobago	1982 to 1991	1.000 (1.000, 1.000)
Trinidad and Tobago	1987 to 1996	1.018 (0.998, 1.037)
Trinidad and Tobago	1992 to 2001	1.027 (1.000, 1.054)
Trinidad and Tobago	1997 to 2006	0.928 (0.888, 0.969)
Tunisia	1942 to 1951	0.799 (0.748, 0.853)
Tunisia	1947 to 1956	0.828 (0.790, 0.867)
Tunisia	1952 to 1961	0.856 (0.824, 0.890)
Tunisia	1957 to 1966	0.885 (0.856, 0.916)
Tunisia	1962 to 1971	0.912 (0.884, 0.941)
Tunisia	1967 to 1976	0.934 (0.907, 0.962)
Tunisia	1972 to 1981	0.961 (0.934, 0.989)
Tunisia	1977 to 1986	0.983 (0.955, 1.012)
Tunisia	1982 to 1991	1.000 (1.000, 1.000)
Tunisia	1987 to 1996	0.919 (0.886, 0.953)

Tunisia	1992 to 2001	0.824 (0.783, 0.868)
Tunisia	1997 to 2006	0.777 (0.716, 0.844)
Turkey	1942 to 1951	0.814 (0.796, 0.833)
Turkey	1947 to 1956	0.839 (0.825, 0.853)
Turkey	1952 to 1961	0.863 (0.851, 0.875)
Turkey	1957 to 1966	0.891 (0.880, 0.903)
Turkey	1962 to 1971	0.916 (0.905, 0.927)
Turkey	1967 to 1976	0.938 (0.927, 0.948)
Turkey	1972 to 1981	0.961 (0.951, 0.972)
Turkey	1977 to 1986	0.983 (0.973, 0.994)
Turkey	1982 to 1991	1.000 (1.000, 1.000)
Turkey	1987 to 1996	1.010 (0.997, 1.024)
Turkey	1992 to 2001	0.953 (0.936, 0.970)
Turkey	1997 to 2006	0.882 (0.858, 0.908)
Turkmenistan	1942 to 1951	0.896 (0.882, 0.911)
Turkmenistan	1947 to 1956	0.904 (0.895, 0.914)
Turkmenistan	1952 to 1961	0.918 (0.910, 0.926)
Turkmenistan	1957 to 1966	0.932 (0.925, 0.939)
Turkmenistan	1962 to 1971	0.943 (0.937, 0.950)
Turkmenistan	1967 to 1976	0.958 (0.952, 0.964)
Turkmenistan	1972 to 1981	0.974 (0.968, 0.980)
Turkmenistan	1977 to 1986	0.986 (0.980, 0.992)
Turkmenistan	1982 to 1991	1.000 (1.000, 1.000)
Turkmenistan	1987 to 1996	1.015 (1.007, 1.022)

Turkmenistan	1992 to 2001	1.021 (1.012, 1.030)
Turkmenistan	1997 to 2006	0.868 (0.856, 0.880)
Tuvalu	1942 to 1951	0.917 (0.699, 1.203)
Tuvalu	1947 to 1956	0.931 (0.757, 1.146)
Tuvalu	1952 to 1961	0.950 (0.792, 1.140)
Tuvalu	1957 to 1966	0.967 (0.819, 1.141)
Tuvalu	1962 to 1971	0.979 (0.838, 1.144)
Tuvalu	1967 to 1976	0.986 (0.849, 1.145)
Tuvalu	1972 to 1981	1.000 (0.864, 1.159)
Tuvalu	1977 to 1986	1.005 (0.865, 1.168)
Tuvalu	1982 to 1991	1.000 (1.000, 1.000)
Tuvalu	1987 to 1996	0.895 (0.750, 1.069)
Tuvalu	1992 to 2001	0.786 (0.627, 0.984)
Tuvalu	1997 to 2006	0.707 (0.506, 0.988)
Uganda	1942 to 1951	1.094 (1.069, 1.121)
Uganda	1947 to 1956	1.089 (1.071, 1.107)
Uganda	1952 to 1961	1.086 (1.072, 1.101)
Uganda	1957 to 1966	1.079 (1.067, 1.091)
Uganda	1962 to 1971	1.071 (1.061, 1.081)
Uganda	1967 to 1976	1.058 (1.049, 1.067)
Uganda	1972 to 1981	1.045 (1.037, 1.054)
Uganda	1977 to 1986	1.026 (1.018, 1.034)
Uganda	1982 to 1991	1.000 (1.000, 1.000)
Uganda	1987 to 1996	0.965 (0.956, 0.973)

Uganda	1992 to 2001	0.916 (0.907, 0.926)
Uganda	1997 to 2006	0.642 (0.632, 0.652)
Ukraine	1942 to 1951	0.831 (0.822, 0.839)
Ukraine	1947 to 1956	0.840 (0.833, 0.847)
Ukraine	1952 to 1961	0.857 (0.851, 0.864)
Ukraine	1957 to 1966	0.878 (0.872, 0.884)
Ukraine	1962 to 1971	0.902 (0.896, 0.907)
Ukraine	1967 to 1976	0.924 (0.918, 0.930)
Ukraine	1972 to 1981	0.947 (0.941, 0.952)
Ukraine	1977 to 1986	0.972 (0.966, 0.978)
Ukraine	1982 to 1991	1.000 (1.000, 1.000)
Ukraine	1987 to 1996	1.029 (1.021, 1.037)
Ukraine	1992 to 2001	1.039 (1.027, 1.050)
Ukraine	1997 to 2006	0.905 (0.888, 0.922)
United Arab Emirates	1942 to 1951	0.697 (0.682, 0.712)
United Arab Emirates	1947 to 1956	0.730 (0.721, 0.740)
United Arab Emirates	1952 to 1961	0.764 (0.756, 0.771)
United Arab Emirates	1957 to 1966	0.798 (0.792, 0.805)
United Arab Emirates	1962 to 1971	0.837 (0.831, 0.843)
United Arab Emirates	1967 to 1976	0.877 (0.871, 0.882)
United Arab Emirates	1972 to 1981	0.923 (0.918, 0.929)
United Arab Emirates	1977 to 1986	0.969 (0.964, 0.974)
United Arab Emirates	1982 to 1991	1.000 (1.000, 1.000)
United Arab Emirates	1987 to 1996	1.041 (1.033, 1.050)

United Arab Emirates	1992 to 2001	1.098 (1.087, 1.110)
United Arab Emirates	1997 to 2006	1.161 (1.142, 1.181)
United Kingdom	1942 to 1951	0.727 (0.710, 0.745)
United Kingdom	1947 to 1956	0.762 (0.747, 0.777)
United Kingdom	1952 to 1961	0.795 (0.781, 0.809)
United Kingdom	1957 to 1966	0.825 (0.812, 0.838)
United Kingdom	1962 to 1971	0.855 (0.843, 0.867)
United Kingdom	1967 to 1976	0.885 (0.873, 0.898)
United Kingdom	1972 to 1981	0.923 (0.910, 0.936)
United Kingdom	1977 to 1986	0.961 (0.948, 0.975)
United Kingdom	1982 to 1991	1.000 (1.000, 1.000)
United Kingdom	1987 to 1996	1.023 (1.005, 1.040)
United Kingdom	1992 to 2001	1.032 (1.009, 1.056)
United Kingdom	1997 to 2006	1.071 (1.033, 1.109)
United Republic of Tanzania	1942 to 1951	0.977 (0.959, 0.995)
United Republic of Tanzania	1947 to 1956	0.988 (0.976, 1.001)
United Republic of Tanzania	1952 to 1961	0.997 (0.987, 1.007)
United Republic of Tanzania	1957 to 1966	1.003 (0.995, 1.012)
United Republic of Tanzania	1962 to 1971	1.006 (0.999, 1.014)
United Republic of Tanzania	1967 to 1976	1.008 (1.001, 1.015)
United Republic of Tanzania	1972 to 1981	1.010 (1.004, 1.016)
United Republic of Tanzania	1977 to 1986	1.007 (1.001, 1.014)
United Republic of Tanzania	1982 to 1991	1.000 (1.000, 1.000)
United Republic of Tanzania	1987 to 1996	0.983 (0.976, 0.990)

United Republic of Tanzania	1992 to 2001	0.952 (0.944, 0.960)
United Republic of Tanzania	1997 to 2006	0.609 (0.601, 0.617)
United States of America	1942 to 1951	0.742 (0.722, 0.763)
United States of America	1947 to 1956	0.758 (0.742, 0.774)
United States of America	1952 to 1961	0.789 (0.775, 0.804)
United States of America	1957 to 1966	0.836 (0.822, 0.851)
United States of America	1962 to 1971	0.873 (0.860, 0.887)
United States of America	1967 to 1976	0.901 (0.887, 0.915)
United States of America	1972 to 1981	0.928 (0.914, 0.942)
United States of America	1977 to 1986	0.966 (0.951, 0.981)
United States of America	1982 to 1991	1.000 (1.000, 1.000)
United States of America	1987 to 1996	0.997 (0.979, 1.016)
United States of America	1992 to 2001	1.022 (0.998, 1.046)
United States of America	1997 to 2006	1.106 (1.067, 1.146)
United States Virgin Islands	1942 to 1951	0.885 (0.805, 0.972)
United States Virgin Islands	1947 to 1956	0.897 (0.829, 0.970)
United States Virgin Islands	1952 to 1961	0.918 (0.854, 0.986)
United States Virgin Islands	1957 to 1966	0.937 (0.876, 1.002)
United States Virgin Islands	1962 to 1971	0.957 (0.897, 1.021)
United States Virgin Islands	1967 to 1976	0.973 (0.913, 1.036)
United States Virgin Islands	1972 to 1981	0.998 (0.937, 1.063)
United States Virgin Islands	1977 to 1986	0.999 (0.936, 1.067)
United States Virgin Islands	1982 to 1991	1.000 (1.000, 1.000)
United States Virgin Islands	1987 to 1996	1.011 (0.931, 1.099)

United States Virgin Islands	1992 to 2001	1.010 (0.903, 1.128)
United States Virgin Islands	1997 to 2006	1.017 (0.855, 1.211)
Uruguay	1942 to 1951	0.770 (0.752, 0.789)
Uruguay	1947 to 1956	0.811 (0.796, 0.826)
Uruguay	1952 to 1961	0.843 (0.829, 0.857)
Uruguay	1957 to 1966	0.879 (0.866, 0.892)
Uruguay	1962 to 1971	0.907 (0.894, 0.920)
Uruguay	1967 to 1976	0.933 (0.920, 0.945)
Uruguay	1972 to 1981	0.955 (0.942, 0.968)
Uruguay	1977 to 1986	0.978 (0.965, 0.992)
Uruguay	1982 to 1991	1.000 (1.000, 1.000)
Uruguay	1987 to 1996	1.022 (1.005, 1.039)
Uruguay	1992 to 2001	0.995 (0.973, 1.017)
Uruguay	1997 to 2006	0.988 (0.952, 1.025)
Uzbekistan	1942 to 1951	0.923 (0.908, 0.937)
Uzbekistan	1947 to 1956	0.930 (0.921, 0.939)
Uzbekistan	1952 to 1961	0.941 (0.934, 0.948)
Uzbekistan	1957 to 1966	0.952 (0.945, 0.958)
Uzbekistan	1962 to 1971	0.961 (0.955, 0.967)
Uzbekistan	1967 to 1976	0.972 (0.966, 0.977)
Uzbekistan	1972 to 1981	0.982 (0.976, 0.987)
Uzbekistan	1977 to 1986	0.991 (0.986, 0.996)
Uzbekistan	1982 to 1991	1.000 (1.000, 1.000)
Uzbekistan	1987 to 1996	1.004 (0.997, 1.010)

Uzbekistan	1992 to 2001	0.987 (0.979, 0.994)
Uzbekistan	1997 to 2006	0.738 (0.728, 0.748)
Vanuatu	1942 to 1951	0.964 (0.892, 1.041)
Vanuatu	1947 to 1956	0.983 (0.931, 1.037)
Vanuatu	1952 to 1961	1.003 (0.959, 1.049)
Vanuatu	1957 to 1966	1.021 (0.982, 1.060)
Vanuatu	1962 to 1971	1.037 (1.002, 1.073)
Vanuatu	1967 to 1976	1.048 (1.015, 1.082)
Vanuatu	1972 to 1981	1.065 (1.032, 1.098)
Vanuatu	1977 to 1986	1.072 (1.040, 1.106)
Vanuatu	1982 to 1991	1.000 (1.000, 1.000)
Vanuatu	1987 to 1996	0.882 (0.850, 0.915)
Vanuatu	1992 to 2001	0.835 (0.797, 0.875)
Vanuatu	1997 to 2006	0.762 (0.710, 0.818)
Venezuela (Bolivarian Republic of)	1942 to 1951	0.859 (0.845, 0.874)
Venezuela (Bolivarian Republic of)	1947 to 1956	0.876 (0.866, 0.886)
Venezuela (Bolivarian Republic of)	1952 to 1961	0.892 (0.883, 0.901)
Venezuela (Bolivarian Republic of)	1957 to 1966	0.910 (0.902, 0.919)
Venezuela (Bolivarian Republic of)	1962 to 1971	0.931 (0.923, 0.938)
Venezuela (Bolivarian Republic of)	1967 to 1976	0.948 (0.940, 0.955)
Venezuela (Bolivarian Republic of)	1972 to 1981	0.968 (0.960, 0.975)
Venezuela (Bolivarian Republic of)	1977 to 1986	0.986 (0.978, 0.993)
Venezuela (Bolivarian Republic of)	1982 to 1991	1.000 (1.000, 1.000)
Venezuela (Bolivarian Republic of)	1987 to 1996	1.009 (0.999, 1.018)

Venezuela (Bolivarian Republic of)	1992 to 2001	0.999 (0.987, 1.012)
Venezuela (Bolivarian Republic of)	1997 to 2006	0.933 (0.915, 0.951)
Viet Nam	1942 to 1951	1.057 (1.031, 1.084)
Viet Nam	1947 to 1956	1.071 (1.053, 1.089)
Viet Nam	1952 to 1961	1.076 (1.061, 1.091)
Viet Nam	1957 to 1966	1.075 (1.062, 1.088)
Viet Nam	1962 to 1971	1.072 (1.060, 1.084)
Viet Nam	1967 to 1976	1.062 (1.051, 1.073)
Viet Nam	1972 to 1981	1.050 (1.040, 1.061)
Viet Nam	1977 to 1986	1.027 (1.017, 1.038)
Viet Nam	1982 to 1991	1.000 (1.000, 1.000)
Viet Nam	1987 to 1996	0.967 (0.956, 0.979)
Viet Nam	1992 to 2001	0.857 (0.842, 0.871)
Viet Nam	1997 to 2006	0.571 (0.554, 0.588)
Yemen	1942 to 1951	0.931 (0.904, 0.959)
Yemen	1947 to 1956	0.952 (0.933, 0.971)
Yemen	1952 to 1961	0.970 (0.955, 0.986)
Yemen	1957 to 1966	0.985 (0.972, 0.999)
Yemen	1962 to 1971	0.996 (0.983, 1.008)
Yemen	1967 to 1976	1.002 (0.991, 1.014)
Yemen	1972 to 1981	1.009 (0.999, 1.019)
Yemen	1977 to 1986	1.009 (0.999, 1.019)
Yemen	1982 to 1991	1.000 (1.000, 1.000)
Yemen	1987 to 1996	0.981 (0.970, 0.993)

Yemen	1992 to 2001	0.924 (0.911, 0.938)
Yemen	1997 to 2006	0.746 (0.730, 0.761)
Zambia	1942 to 1951	1.046 (1.029, 1.063)
Zambia	1947 to 1956	1.042 (1.030, 1.054)
Zambia	1952 to 1961	1.037 (1.028, 1.047)
Zambia	1957 to 1966	1.035 (1.027, 1.043)
Zambia	1962 to 1971	1.032 (1.025, 1.039)
Zambia	1967 to 1976	1.029 (1.022, 1.035)
Zambia	1972 to 1981	1.024 (1.018, 1.030)
Zambia	1977 to 1986	1.015 (1.009, 1.020)
Zambia	1982 to 1991	1.000 (1.000, 1.000)
Zambia	1987 to 1996	0.975 (0.969, 0.982)
Zambia	1992 to 2001	0.933 (0.926, 0.940)
Zambia	1997 to 2006	0.795 (0.787, 0.804)
Zimbabwe	1942 to 1951	0.976 (0.927, 1.027)
Zimbabwe	1947 to 1956	0.977 (0.943, 1.012)
Zimbabwe	1952 to 1961	0.982 (0.954, 1.011)
Zimbabwe	1957 to 1966	0.986 (0.962, 1.011)
Zimbabwe	1962 to 1971	0.993 (0.971, 1.014)
Zimbabwe	1967 to 1976	0.993 (0.974, 1.013)
Zimbabwe	1972 to 1981	0.996 (0.978, 1.015)
Zimbabwe	1977 to 1986	0.999 (0.981, 1.017)
Zimbabwe	1982 to 1991	1.000 (1.000, 1.000)
Zimbabwe	1987 to 1996	0.992 (0.971, 1.012)

Zimbabwe	1992 to 2001	0.766 (0.746, 0.786)
Zimbabwe	1997 to 2006	0.558 (0.536, 0.582)

CLD, chronic liver disease; WCBA, women of childbearing age.
